# ALKBH5 promotes hypopharyngeal squamous cell carcinoma apoptosis by targeting TLR2 in a YTHDF1/IGF2BP2-mediated manner

**DOI:** 10.1038/s41420-023-01589-6

**Published:** 2023-08-23

**Authors:** Jing Ye, Yuting Wu, Yao Chen, Yiyue Ren, Xiaohua Jiang, Zhihuai Dong, Jingna Zhang, Mao Jin, Xiaozhen Chen, Zhanggui Wang, Mang Xiao

**Affiliations:** 1 Department of Otolaryngology Head and Neck Surgery, Sir Run Run Shaw Hospital, College of Medicine, Zhejiang `University, Hangzhou, Zhejiang China; 2 Department of Oncology, Sir Run Run Shaw Hospital, College of Medicine, Zhejiang University, Hangzhou, Zhejiang China; 3 Department of Head and Neck Surgery, Institute of Micro-Invasive Surgery of Zhejiang University, Sir Run Run Shaw Hospital, College of Medicine, Zhejiang University Hangzhou, Zhejiang Province, China; 4 Central Laboratory, Institute of Clinical Science, Sir Run Run Shaw Hospital, College of Medicine, Zhejiang University Hangzhou, Zhejiang Province, China; 5 Department of Radiotherapy, The Second People’s Hospital of Anhui Province, Hefei, Anhui China

**Keywords:** Head and neck cancer, Oncogenesis, Apoptosis, Diagnostic markers

## Abstract

Hypopharyngeal squamous cell carcinoma (HPSCC) is one of the most aggressive cancers and is notorious for its extremely poor prognosis. However, very few molecular biological studies have been performed. As a novel method of epigenetic gene modulation, N6-methyladenosine (m^6^A) RNA modification occurs in HPSCC. The expression of the m^6^A demethylase AlkB homolog 5 (ALKBH5) is frequently downregulated in human HPSCC. Furthermore, we found that ALKBH5 impaired cell proliferation by regulating human Toll-like receptor 2 (TLR2) in an m^6^A-dependent manner in HPSCC cells. ALKBH5 decreased TLR2 m^6^A modification, which could be recognized by the m^6^A readers IGF2BP2 and YTHDF1. IGF2BP2 facilitates TLR2 mRNA stability, whereas YTHDF1 promotes TLR2 mRNA translation. The current work uncovered a critical function of ALKBH5 in TLR2 regulation and provides a novel role for m^6^A demethylation of mRNA in HPSCC. The inhibition of m^6^A modification of ALKBH5 in HPSCC deserves further clinical investigation.

## Introduction

Hypopharyngeal squamous cell carcinoma (HPSCC) is an extremely aggressive malignancy that, in 60% of cases, is detected at an advanced stage, contributing to the generally poor prognosis and the low 5-year overall survival (OS) rate [1–5]. Furthermore, these tumors and associated treatments may have a negative impact on quality of life, and patients’ capacity to speak and swallow and social functions may be severely compromised [1, 6].

N(6)-methyladenosine (m^6^A) is the most abundant RNA modification and is reversibly and dynamically installed by methyltransferases (writers), removed by demethylases (erasers) and recognized by m^6^A-binding proteins (readers) [7]. Methyltransferase-like 3 (METTL3), methyltransferase-like 14 (METTL14), and Wilms tumor 1-associated protein are a classic complex of writers known as WMM (WTAP). The erasers include obesity-associated protein (FTO) and fat-mass and AlkB homolog 5 (ALKBH5) [8]. The readers include YTH domain-containing proteins (YTHDC1/2), YT521-B homology (YTH) domain-containing family proteins (YTHDF1/2/3), the insulin-like growth factor 2 mRNA-binding protein family (IGF2BP1/2/3) and the heterogeneous nuclear ribonucleoprotein family [9, 10]. m^6^A methylation regulates the translation, splicing, nuclear export, and decay of target RNA, thereby playing a critical role in a range of biological processes [9, 11]. Since the advent of methylated RNA immunoprecipitation-sequencing (MeRIP-seq) technology, a growing number of molecular mechanisms underlying m^6^A-mediated RNA regulation of self-renewal, the DNA damage response and tumorigenesis in various cancers have been comprehensively clarified [12, 13]. These studies identified molecular mechanisms associated with carcinogenesis, cell proliferation, cell cycle, migration and invasion, apoptosis, and autophagy [14, 15]. However, few studies have investigated how m^6^A plays a role in HPSCC development and proliferation [10, 16].

ALKBH5, an m^6^A demethylase, belongs to the AlkB family of dioxygenases. ALKBH5 modulates the assembly/modification of mRNA processing components, demethylates m^6^A mRNA, and regulates the export and stabilization of mRNA [17]. As shown in previous reports, ALKBH5 is associated with cisplatin chemosensitivity in bladder cancers through a casein kinase 2 (CK2) alpha-mediated glycolysis pathway in an m^6^A-dependent manner [18]. ALKBH5 acts as a key regulator that protects cells from DNA damage and apoptosis during reactive oxygen species (ROS)-induced stress [19]. ALKBH5 overexpression reduced the in vitro migration and invasion of esophageal squamous cell carcinoma cells, which was partly attributed to G1-phase arrest [20]. ALKBH5 is directly regulated by DDX3, which leads to decreased m^6^A methylation of FOXM1 and NANOG nascent transcripts and contributes to chemoresistance [21]. Our prior research confirmed that AlKBH5 inhibited ferroptosis in HPSCC. This finding suggests that the involvement of ALKBH5 in cancer may be significant but baffling. However, it remains unknown whether ALKBH5 contributes to the development of HPSCC, and its role in HPSCC apoptosis is still unclear.

Toll-like receptors (TLRs) are mainly associated with innate immune cells, and their dysregulation has been reported in multiple inflammation-associated cancers [22]. However, TLR2 is also present in epithelial tissues and is upregulated in proportion to proliferating cells [23]. It has been reported that TLR2 is involved in esophageal cancer in patients with lymph node metastasis, [24]. in laryngeal squamous cell carcinoma, which was closely associated with tumor clinical stage and lymph node metastasis, [23]. and in gastric cancer (GC), [25]. and TLR2 has been reported to be involved in apoptosis. 25-Hydroxycholesterol (25HC), which is a type of oxysterol, promotes GC invasion partly through the upregulation of TLR2/nuclear factor (NF)-kappaB signaling-mediated matrix metalloproteinase (MMP) expression [25]. TLR2 is also upregulated on primitive acute myeloid leukemia (AML) cells, and agoniztic targeting of TLR1/TLR2 forces AML cells to undergo apoptosis by p38 MAPK-dependent activation of Caspase 3 and differentiation by activating NF-kappaB [26] TLR2 knockdown significantly suppressed the proliferation of colorectal cancer cells through NF-kappaB, cyclin D1, and cyclin D3 regulation, leading to G1 phase arrest [27] Exopolysaccharides (EPS) from Lactobacillus plantarum NCU116 (EPS116) suppressed the proliferation of intestinal epithelial cancer cells through c-Jun-dependent Fas/Fasl-mediated apoptosis via TLR2 [28] The growth responsiveness of human GC cells induced by TLR2 corresponds to the upregulation of six antiapoptotic genes (BCL2A1, BCL2, BIRC3, CFLAR, IER3, TNFAIP3) and the downregulation of two tumor suppressor genes (PDCD4, TP53INP1) [22].

However, the identity of specific TLRs and their molecular targets that drive the pathogenesis of HPSCC is currently unclear. Moreover, the transcriptional modulation of TLRs, particularly m6A modification after TLR2 transcription, has not yet been elucidated. In the current study, we demonstrated that the function of TLR2 was regulated by ALKBH5, IGF2BP2 and YTHDF1 and under the control of m^6^A modification to modulate cell proliferation and apoptosis in HPSCC. These findings suggested that m^6^A modification of TLR2 may be a promising therapeutic target for HPSCC.

## Results

### The expression of the m^6^A demethylase ALKBH5 is frequently downregulated and associated with the pathogenesis and progression of human HPSCC

**Fig. 1:**
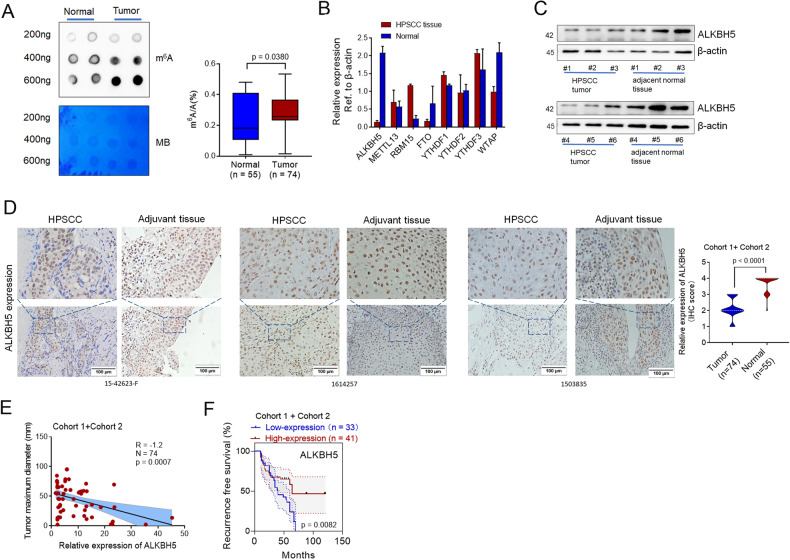
ALKBH5 is frequently downregulated and negatively associated with the pathogenesis and progression of human HPSCC. **A** The m^6^A levels of tumor and paired normal tissues were determined by m^6^A dot blots and m^6^A methylation quantification. **B** The protein levels of m6A de/methylase and readers were analyzed by Western blot analysis of three pairs of normal tissue and the HPSCC tumor tissue. **C** Representative IHC images of ALKBH5 expression in the tumor and adjacent tissue. **D** The correlation between ALKBH5 IHC scores and maximum tumor diameter. **E** The correlation between ALKBH5 expression and recurrence-free survival time in HPSCC patients.

To determine whether the polyadenylated RNA (poly(A) RNA) m^6^A mechanism is involved in HPSCC prognosis, m^6^A methylation was analyzed in 14 primary biopsy tissues from **Cohort 1**. The modification of m^6^A in total RNA from tumor and adjacent tissue was observed and confirmed by m^6^A methylation quantification and m^6^A dot blots. The results showed that the m^6^A level was upregulated in tumor tissue compared with normal tissue (Fig. 1A). Therefore, m^6^A methylation may participate in HPSCC development.

We measured the expression levels of m^6^A-related genes in three paired tumor and adjacent tissues by qPCR. Interestingly, ALKBH5 was the most significantly downregulated gene in tumor compared with normal tissues (Fig. 1B). We measured the expression of ALKBH5 in six paired tumor and adjacent tissues by Western blotting. The expression of ALKBH5 was lower than that in paired adjacent tissue (Fig. 1C). IHC analysis of 74 HPSCC patients in **Cohort 1** and **Cohort 2** confirmed that the expression level of ALKBH5 in cancer tissues was much lower than that in paired normal tissues (Fig. 1D). Furthermore, ALKBH5 gradually decreased with increasing tumor grade in HPSCC tissue (Supplementary Fig. S1A) and was negatively correlated with the maximum tumor diameter and lymph node metastasis (Fig. 1E, Supplementary Fig. S1B). Furthermore, **Cohort 1** and **Cohort 2** datasets were used to determine the prognostic correlation between ALKBH5 expression and HPSCC patient survival. The results showed that the recurrence-free survival time of patients with high ALKBH5 expression (*n* = 33) was longer than that of patients with low ALKBH5 expression (*n* = 41) (*P* = 0.0082) (Fig. 1F). Overall, the results derived from our datasets demonstrated that ALKBH5 was frequently downregulated in HPSCC and negatively associated with the pathogenesis and progression of HPSCC.

### ALKBH5 significantly inhibited cell growth and proliferation and promoted apoptosis in HPSCC

**Fig. 2:**
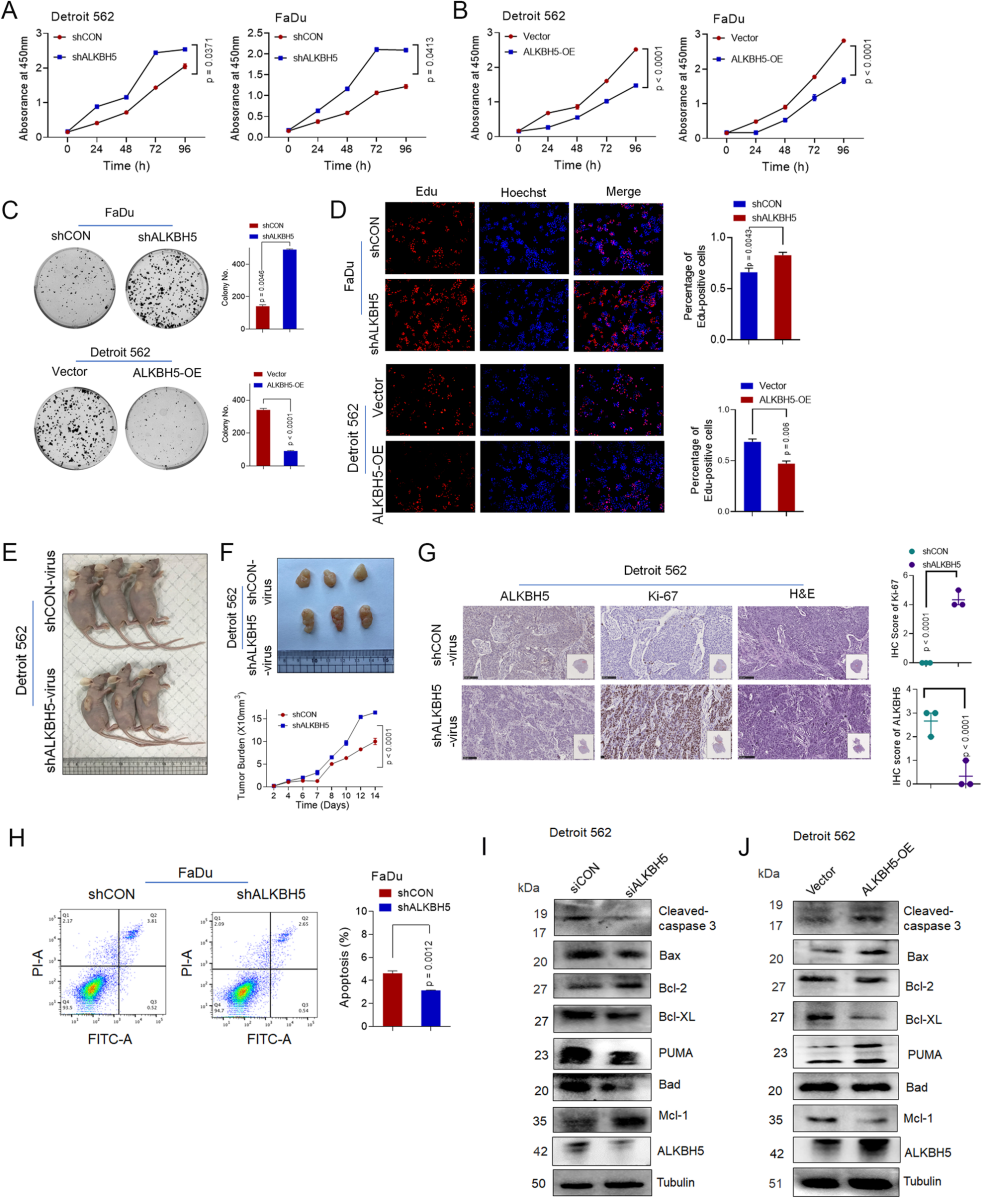
ALKBH5 significantly suppressed proliferation and promoted apoptosis in HPSCC cells in vitro and in vivo. **A** CCK8 assays of Detroit 562 and FaDu cells transfected with control or shALKBH5. **B** CCK8 assays of Detroit 562 and FaDu cells transfected with control or ALKBH5-WT plasmids. Representative images and quantitative analysis of colony formation (**C**) and EdU staining (**D**) of HPSCC cells transfected with control, shALKBH5 or ALKBH5-WT plasmids. Representative images of xenografted tumors (**E**) and statistical analysis of tumor burden (**F**) of the indicated xenografted tumors (L) in nude mice bearing shALKBH5 or shCON cells in indicated groups (*n* = 3). **G** Representative HE and immunohistochemical images and quantification of ALKBH5 and Ki-67 in the indicated xenografted tumor tissues. Scale bars: 100 μm. **H** Representative images and quantification of flow cytometric analysis of apoptosis in Detroit 562 and FaDu cells transfected with control or shALKBH5. **I** Apoptotic markers (cleaved caspase 3, cleaved PARP, Bax, PUMA, and Bad) and antiapoptotic markers (Bcl-2, Bcl-xl, Mcl-1) in Detroit 562 cells transfected with siRNA or ALKBH5 siRNA were analyzed by western blotting. **J** Apoptotic markers (cleaved caspase 3, Bax, PUMA, and Bad) and antiapoptotic markers (Bcl-2, Bcl-xl, Mcl-1) in Detroit 562 cells transfected with WT or ALKBH5-WT were analyzed by Western blotting.

Next, we explored the function of ALKBH5 in tumorigenesis. We used siRNAs and shRNAs to knock down ALKBH5 (siALKBH5 and shALKBH5) or overexpressed these genes by transfecting Detroit 562 and FaDu cells with a plasmid encoding ALKBH5. The transfection efficiency of ALKBH5 shRNA and the plasmid was examined by qPCR and Western blot analysis (Supplementary Fig. S1C). CCK8 assays (Fig. 2A), Clone formation assays (Fig. 2B) and EdU staining (Fig. 2C) confirmed the results. Similar results for cellular migration, invasion and growth were observed in FaDu cells, as detected by wound healing and Transwell assays (Supplementary Fig. S2A, B). Notably, ALKBH5 overexpression decreased the oncogenic behaviors of Detroit 562 cells, as examined by CCK8 assays (Fig. 2B), clone formation assays (Fig. 2C), EdU staining (Fig. 2D), wound healing and Transwell assays (Supplementary Fig. S2C, D).

To determine the effect of ALKBH5 on HPSCC growth in vivo, we developed a xenograft model. Compared to control mice, ALKBH5 knockdown enhanced the susceptibility of xenograft mice, as indicated by increased tumor growth rate and burden (Fig. 2E, F). ALKBH5 and Ki67 staining (as a proliferation indicator) confirmed the results (Fig. 2G). Similar results for bone metastasis in vivo were obtained in Detroit 562 cells (Supplementary Fig. S2E). Furthermore, knocking down ALKBH5 inhibited spontaneous apoptosis in Detroit 562 and FaDu cells, as determined by flow cytometry (Fig. 2H).

We next examined apoptotic and antiapoptotic markers in Detroit 562 and FaDu cells with or without ALKBH5 depletion. Apoptotic markers (cleaved caspase 3, cleaved PARP, PUMA-23 kDa, Bad and Bax) were decreased, and antiapoptotic markers (Bcl-2, Bcl-xL, Mcl-1) were increased in ALKBH5-silenced cells (Fig. 2I). The opposite result was observed in response to ALKBH5 overexpression (Fig. 2J, Supplementary Fig. S2F). Apoptotic markers (cleaved caspase 3, Bax, Bad, PUMA-23 kDa, cleaved PARP) were increased, and antiapoptotic markers (Bcl-2, Mcl-1, Bcl-xL) were decreased in ALKBH5-overexpressing cells. Consequently, ALKBH5 inhibits cell growth and proliferation and promotes apoptosis in HPSCC.

### ALKBH5 mediates the protein expression of TLR2 in an m^6^A-dependent manner in HPSCC

Since ALKBH5 was involved in the development of HPSCC, we next investigated whether ALKBH5-mediated m^6^A modification affected the maintenance of HPSCC oncogenic behavior. To identify and localize m^6^A at the transcriptome-wide level, m^6^A sequencing (m^6^A-seq) was performed on mRNA purified from ALKBH5-knockdown (shALKBH5) and control (shCON) FaDu cells. Quality control, including Reads density, Principal component analysis (PCA), Peak density, DistProfile and Coverage_By Samples, showed that two repeats (shCON: shCON_1 and shCON_2; shALKBH5: shK5_1 and shK5_2) in each sample clustered together, suggesting good repeatability (Additional file 5). By using the HOMER motif discovery tool, the consensus **GGACU’’** was found to be the most enriched among the m^6^A peaks. The peaks were in protein-coding transcripts and enriched in the 5’UTR and 3′UTR, especially proximal stop codons, as described in our previous study [29].

**Fig. 3:**
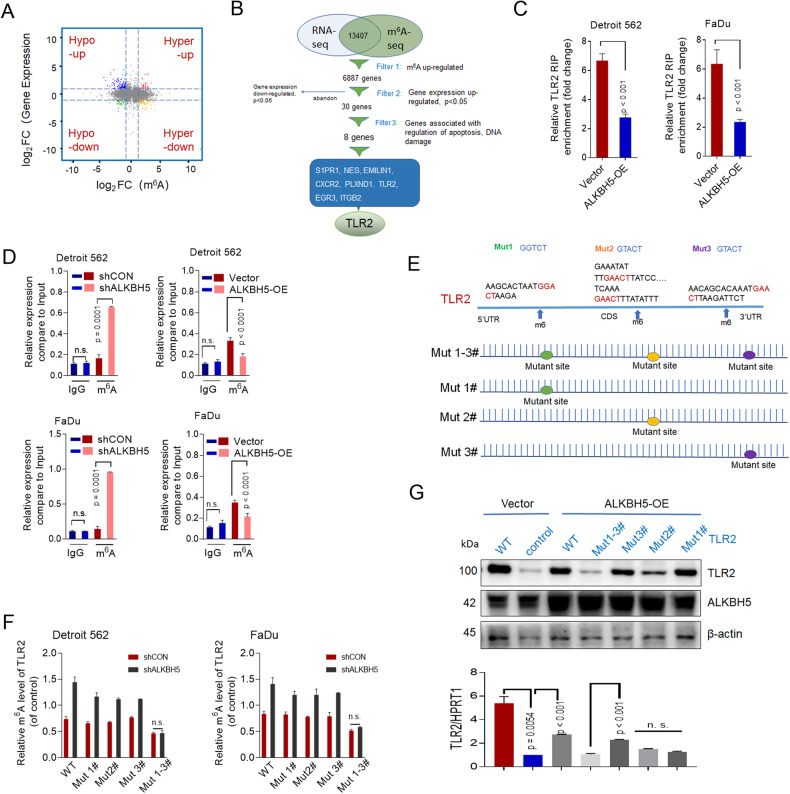
ALKBH5 mediates the expression of TLR2 in an m^6^A-dependent manner in HPSCC. **A** Distribution of genes with significantly different levels of m^6^A (log2FC) and gene expression (log2 FC) between shCON and shALKBH5 FaDu cells. Hyper-up: hypermethylated m6A and increased expression; Hyper-down: hypermethylated m6A and decreased expression. Hypo-down: hypo-methylated m6A and decreased expression; Hypo-up: hypo-methylated m6A and increased expression. **B** Flow chart of the selected candidate ALKBH5 target genes in FaDu cells. **C** The interaction between ALKBH5 and TLR2 pre-mRNA was detected by RIP in FaDu cells. **D** MeRIP-qPCR was used to assess the relative m6A level of TLR2 pre-mRNA in FaDu cells with ALKBH5 knockdown or overexpression. **E** The m^6^A peaks in the 5′UTR, CDS, and 3’UTR sequences of TLR2 and synonymous mutations in the TLR2 5′UTR, CDS, and 3’UTR. **F** The relative level of m^6^A in TLR2 was determined after the coexpression of shALKBH5 and TLR2 WT/Mut#. **G** The relative mRNA and protein levels of TLR2 were analyzed by qPCR and Western blotting.

Based on the RNA-seq data (Additional File 6), we identified 967 m^6^A hypermethylated genes with downregulated mRNA levels (*p* < 0.05, hyper-down) and 49 genes with m^6^A hypermethylation and upregulated mRNA levels (*p* < 0.05, hyper-up) in shALKBH5 cells compared with shCON cells. A total of 11.4% (13407/117471) of m^6^A-modified transcripts overlapped with the RNA-seq data (Additional File 5). The general m^6^A level of transcripts was upregulated, 48.6% (6518 of 13407) of the genes were downregulated, and 51.4% (6887 of 13407) of the genes were upregulated (Fig. 3A). Considering the role of ALKBH5 in the m^6^A methyltransferase complex, mRNA with hypermethylated m^6^A peaks in shALKBH5-transfected FaDu cells were identified as possible targets. Additionally, we found that the hyper-up transcripts (*p* < 0.05) were mostly enriched in genes involved in “cell response to DNA damage”, “DNA damage stimuli” and “apoptosis phases” according to GO analysis (Supplementary Fig. S3A). However, those hypo-up transcripts were significantly enriched in “oxidation reduction” and “DNA binding”, which are not associated with apoptosis (Supplementary Fig. S3B). Then, related genes with hypermethylated m^6^A and increased expression were selected for further validation.

We identified 30 genes as potential targets involved in regulation of apoptosis and DNA damage (Fig. 3B). qPCR showed that the expression of eight target genes (S1PR1, NES, EMILIN1, CXCR2, PLXND1. TLR2, EGR3, GRK5, and ITGB2) was significantly increased in ALKBH5-knockdown FaDu cells (Supplementary Fig. S3C). Further methylated RNA immunoprecipitation combined with qPCR (MeRIP-qPCR) showed that the m^6^A levels in six genes were significantly increased in ALKBH5-knockdown FaDu cells (Supplementary Fig. S3D). We then validated these six candidate target genes using a cell viability assay. Cell viability was significantly reduced in Detroit 562 and FaDu cells after TLR2 knockdown (Supplementary Fig. S3E, F).

To determine whether the methyltransferase activity of ALKBH5 is needed to control the expression of TLR2, RNA immunoprecipitation (RIP) assays and MeRIP-qPCR were performed to assess TLR2 m^6^A methylation levels after ALKBH5 knockdown. The RIP assay showed that in Detroit 562 and FaDu cells, TLR2 pre-mRNA interacted with ALKBH5 (Fig. 3C). Additionally, MeRIP-qPCR demonstrated that when ALKBH5 was overexpressed or knocked down, the amount of m^6^A-modified TLR2 pre-mRNA in Detroit 562 and FaDu cells decreased or increased, respectively (Fig. 3D). Combined with m^6^A profiling datasets and data from the m^6^A2Target Database, the potential consensus motif GGAC in the 5′UTR, 3′UTR, and CDS of TLR2 were identified (Supplementary Fig. S3G).

To ascertain whether TLR2 transcripts are substrates for ALKBH5, we introduced a synonymous mutation at the putative m^6^A sites in the TLR2 mRNA m^6^A coding region as follows: Mut1#, Mut2#, and Mut3# (fragments containing three individual potential m^6^A sites of 3’UTR, CDS and 5’UTR, respectively) and and Mut1–3# (fragments with three potential mutated m^6^A sites) *[The specific method is described in the paper]* [30]. (Fig. 3E). The RIP assay revealed that TLR2-WT and Mut1#, Mut2#, and Mut3# plasmids did not significantly interact with ALKBH5, suggesting that the interaction between TLR2 and ALKBH5 was unaffected by the TLR2-Mut# series (Supplementary Fig. S3H). We performed MeRIP-qPCR to determine TLR2 m^6^A methylation levels after ALKBH5 knockdown. Our investigation proved that m^6^A modification of TLR2 Mut#, but not TLR2 Mut1–3#, was increased in ALKBH5-knockdown Detroit 562 and FaDu cells compared to their respective controls (Fig. 3F). The opposite results were found in ALKBH5-overexpressing Detroit 562 and FaDu cells compared to cells expressing the ALKBH5 control vector (Supplementary Fig. S3I). This finding suggested that the predicted sites in the 3’UTR, CDS and 5’UTR were m^6^A consensus sites. The mRNA and protein levels of TLR2 were measured in Detroit 562 and FaDu cells with different m^6^A modifications to show that m^6^A modification increased TLR2 expression. We noticed that the mRNA and protein levels of TLR2 were lower in ALKBH5-overexpressing cells that were transfected with the TLR2-Mut1#, Mut2#, and Mut3# plasmids than in cells transfected with the TLR2-WT plasmid (Fig. 3G). Moreover, the mRNA and protein levels of TLR2 were lower in ALKBH5-overexpressing cells transfected with TLR2-Mut1-3# than in cells transfected with Mut1#, Mut2#, and Mut3# (Fig. 3G). Collectively, these results showed that TLR2 pre-mRNA was a target of ALKBH5-mediated demethylation. ALKBH5 directly interacted with the 5′/3’UTR and CDS regions of TLR2.

### ALKBH5 impairs cell proliferation and promotes spontaneous apoptosis by regulating TLR2 in HPSCC cells

**Fig. 4:**
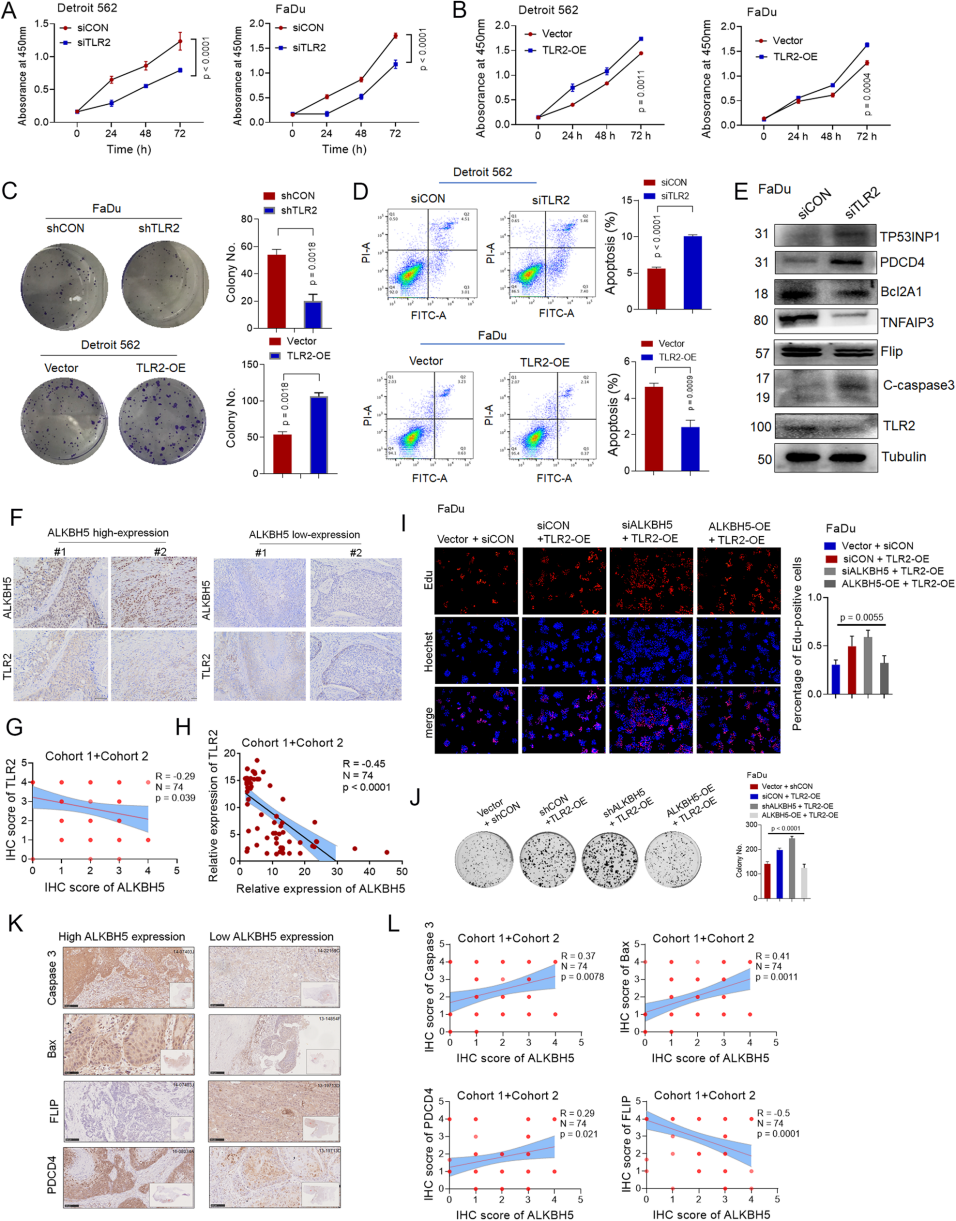
ALKBH5 impairs HPSCC proliferation and promotes apoptosis by regulating TLR2. **A**, **B** CCK8 analysis of Detroit 562 and FaDu cells transfected with control siRNA or a TLR2 siRNA (Aor Vector or TLR2-WT). **C** Representative images and quantification of colony formation by HPSCC transfected with control shRNA or a TLR2 shRNA or with Vector or TLR2-WT. **D** Representative images and quantification of flow cytometric analysis of apoptosis in FaDu cells transfected with control siRNA or TLR2 siRNA or with Vector or TLR2-WT. **E** The protein levels of previously described TLR2-targeted proteins were detected in FaDu cells transfected with control or TLR2 siRNA. Representative IHC images (**F**) and quantification (**G**) of the protein expression levels of ALKBH5 and TLR2 in patients in cohort 1 and cohort 2. Scale bars: 100 μm. **H** The correlation of ALKBH5 mRNA and TLR2 mRNA levels were determined by qPCR in patients in Cohort 1 and Cohort 2. Representative images and quantification of EdU assays (**I**). Colony formation (**J**) by FaDu cells transfected with TLR2-WT plasmids or cotransfected with siALKBH5/shALKBH5 and AlKBH5-WT plasmids. Representative IHC images (**K**) and quantification (**L**) of the expression levels of ALKBH5 and the indicated proteins in patients in cohort 1 and cohort 2. Scale bars: 100 μm. Figure 4I, Figs. 5I and 6J share the same group (Vector + CON siRNA).

We speculated that ALKBH5 exerts its effects via TLR2 regulation. We first used siRNAs and shRNAs to knock down TLR2 (siTLR2 and shTLR2) or overexpressed these genes by transfecting Detroit 562 and FaDu cells with a plasmid encoding TLR2 (Supplementary Fig. S4A). The CCK8 results showed that silencing TLR2 inhibited cell growth in HPSCC cells, while overexpressing TLR2 increased cell growth (Fig. 4A. B). Similar results were observed in colony formation (Fig. 4C) and Transwell assays (Supplementary Fig. S4B, C). Data analysis of **cohort 1** and **cohort 2** showed that TLR2 levels were higher in malignant HPSCC tissues than in the corresponding adjacent normal tissues (Supplementary Fig. S4D).

We assessed the role of TLR2 in apoptosis. As expected, silencing TLR2 increased the spontaneous apoptosis rate in HPSCC cells (Fig. 4D). TLR2 target genes, which are described in previous studies, [22]. including the antiapoptotic markers TNFAIP3 and Bcl-2A1, were also decreased, while the apoptotic markers PDCD4, TP53INP1 and C-caspase 3 were increased in TLR2-silenced FaDu cells (Fig. 4E). Notably, TLR2 overexpression upregulated the levels of antiapoptotic markers (BCL-xl and Bcl-2) and decreased C-caspase 3 (Supplementary Fig. S4E). These results confirm that TLR2 plays an oncogenic role by inhibiting apoptosis in HPSCC cells.

We investigated whether ALKBH5 impacted the expression of TLR2. qPCR and Western blotting showed that the mRNA and protein levels of TLR2 were increased in response to ALKBH5 knockdown, and the opposite effect was found when ALKBH5 was overexpressed (Supplementary Fig. S4F, J). The amounts of TLR2 protein were increased in cells that were cotransfected with shALKBH5 and TLR2-WT plasmids compared with those that were cotransfected with ALKBH5-WT and TLR2-WT plasmids (Supplementary Fig. S4G). Moreover, IHC staining of HPSCC samples from **cohort 1** and **cohort 2** indicated that the protein levels of TLR2 were reduced in tumor tissues when the expression of ALKBH5 was increased (Fig. 4F, G). Consistently with, qPCR identified an inverse relationship between ALKBH5 expression and TLR2 expression (Fig. 4H). Therefore, ALKBH5 suppressed TLR2 mRNA and protein levels.

We investigated whether ALKBH5 suppressed cellular proliferation and spontaneous apoptosis by regulating TLR2. As expected, cell viability and colony formation were increased in cells that were cotransfected with TLR2-WT and shALKBH5 compared to cells that were transfected with TLR2-WT alone, while ALKBH5 overexpression impaired TLR2-induced cell proliferation and colony formation, as determined by EdU staining (Fig. 4I) and colony formation assays (Fig. 4J).

Additionally, the antiapoptotic markers FLIP, TNFAIP3, Bcl-2, and Birc3 were significantly increased, while the apoptotic markers PDCD4, TP53INP1, cleaved caspase 3, and Bax were decreased in TLR2-overexpressing FaDu cells transfected with shALKBH5; the opposite effect was observed in TLR2-overexpressing FaDu cells in response to ALKBH5 overexpression (Supplementary Fig. S4H). Therefore, ALKBH5 promotes spontaneous apoptosis by suppressing the expression of TLR2 in HPSCC in vitro. Consistently, IHC staining revealed that the expression of FLIP was increased in tissues with high ALKBH5 expression; in contrast, PDCD4, Caspase 3 and Bax were decreased in HPSCC with low ALKBH5 expression (Fig. 4K, L). Overall, these results suggested that ALKBH5 impaired cell proliferation and promoted spontaneous apoptosis by regulating TLR2 in HPSCC cells.

### Overexpression of ALKBH5 impairs YTHDF1-mediated translation of TLR2

As shown previously, ALKBH5 decreased the mRNA and protein levels of TLR2, and we then explored whether m^6^A promoted the translation or decay of mRNA [11, 31]. YTH m^6^A RNA-binding protein 1 (YTHDF1) can enhance m^6^A-methylated mRNA translation and recruit translation initiation factors, substantially promoting translation efficiency [32, 33].

**Fig. 5:**
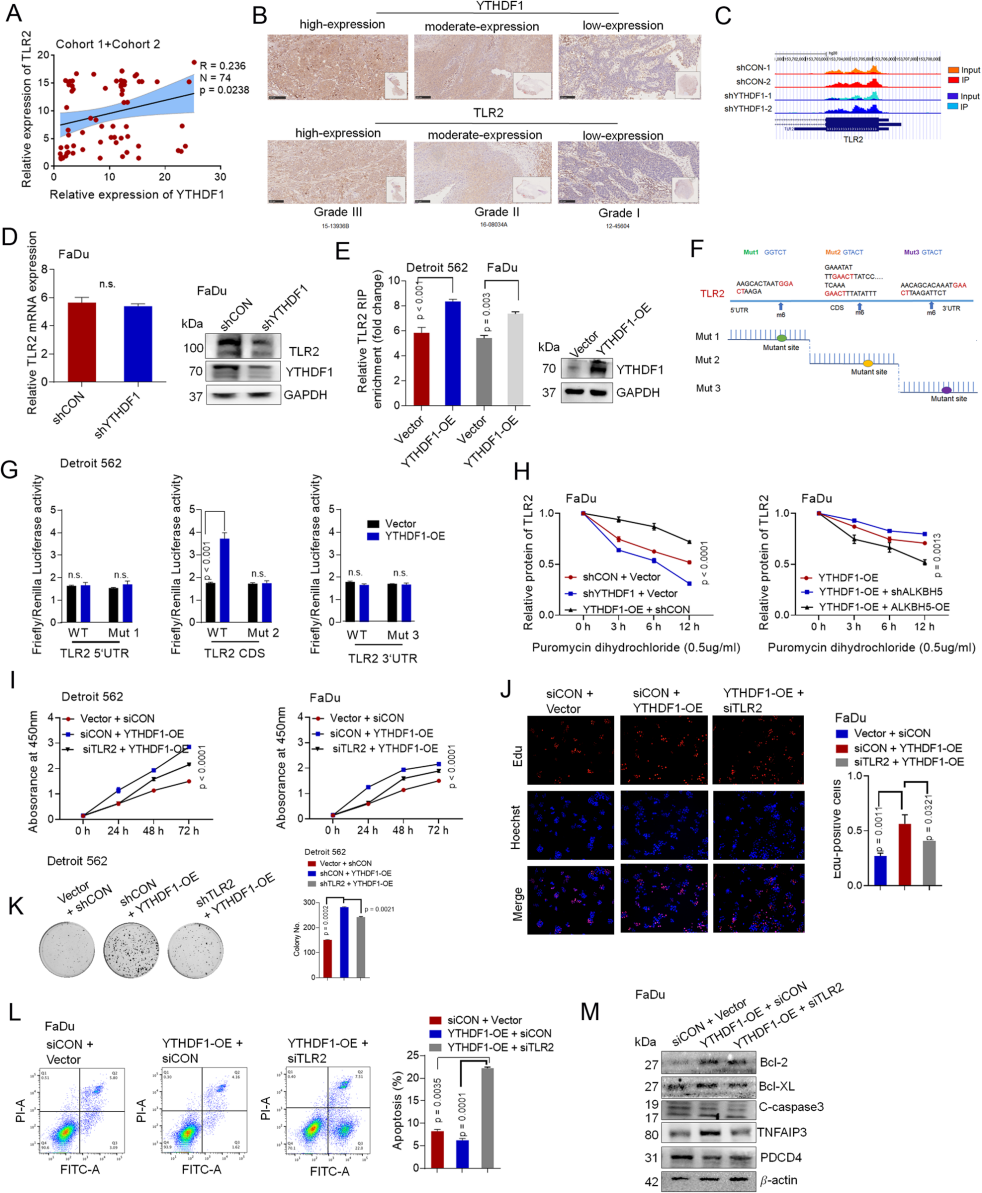
Overexpression of ALKBH5 impairs YTHDF1-mediated translation of TLR2 in m^6^A-dependent manner. **A** The correlation between the mRNA expression of YTHDF1 and TLR2 was analyzed in cohort 1+ cohort 2 patients. **B** The expression of YTHDF1 and TLR2 was determined by IHC in the different grades of HPSCC tissues. **C** IGV tracks displaying the distribution of m^6^A peaks and YTHDF1-binding peaks of TLR2 according to m6A-seq and YTHDF1 RIP-seq analysis of FaDu cells. The specific method is described in the paper [31]. **D** The relative mRNA and protein levels of TLR2 in response to YTHDF1 knockdown were analyzed by qPCR and western blotting. **E** The interaction between YTHDF1 and TLR2 mRNA was determined by RIP assay. **F** m^6^A peaks in the 5′UTR, CDS, and 3’UTR of TLR2 and synonymous mutations. **G** Relative luciferase activity of the WT or TLR2-Mut1/2/3 luciferase reporter in Detroit 562 cells transfected with control or YTHDF1-WT plasmids. Firefly luciferase activity was measured and normalized to Renilla luciferase activity. **H** FaDu cells treated with puromycin and transfected with the corresponding genes were examined by ELISA to determine TLR2 protein levels. Viability was determined by CCK8 assay (**I**). Representative images and quantification of EdU assays (**J**) and colony formation (**K**) in HPSCC cells transfected with YTHDF1-WT plasmids or cotransfected with TLR2 siRNA and YTHDF1-WT plasmids. **L** Representative images and quantification of flow cytometric analysis of apoptosis in FaDu cells transfected with the YTHDF1-WT plasmid or cotransfected with TLR2 siRNA and the YTHDF1-WT plasmid. **M** The relative expression of TLR2-targeted proteins was analyzed by western blotting in FaDu cells transfected with the YTHDF1-WT plasmid or cotransfected with TLR2 siRNA and the YTHDF1-WT plasmid. Figures 4I, 5I and 6J share the same group (Vector + CON siRNA).

Data analysis of **Cohort 1** and **Cohort 2** using the Spearman rank correlation coefficient demonstrated a positive association between YTHDF1 and TLR2 levels (Fig. 5A). In addition, YTHDF1 protein levels increased steadily with increasing HPSCC tumor grade (Fig. 5B). High YTHDF1 levels were related to a poor outcome in patients who received adjuvant platinum-based chemoradiotherapy (CCT) or radiation therapy (RT) (Supplementary Fig. S5A) [34].

To investigate whether TLR2 was the target of YTHDF1 and promoted by m^6^A methylation, we reanalyzed the previous MeRIP-seq and RIP-seq data to identify YTHDF1-bound mRNAs marked with m^6^A [34]. (Additional File 5). Notably, TLR2 was among the overlapping genes identified in the m^6^A-seq and RIP-seq data analyses (Supplementary Fig. S5B). Significant m^6^A peaks in the TLR2 mRNA CDS and YTHDF1 binding enrichment in TLR2 were observed (Fig. 5C). Therefore, YTHDF1 may be the functional m^6^A reader in the current study. We found that knockdown of YTHDF1 did not affect the mRNA level but significantly decreased the protein expression of TLR2 in HPSCC (Fig. 5D). Overexpression of ALKBH5 had no effect on YTHDF1 expression, and overexpression of YTHDF1 had no effect on ALKBH5 expression (Supplementary Fig. S5C). Moreover, RIP using an antibody against FLAG followed by qPCR (RIP-qPCR) confirmed that TLR2 mRNA bound to YTHDF1 (Fig. 5E).

We explored the function of the methylation pattern mediated by YTHDF1. Another synonymous mutation was induced at the putative m^6^A locations in the TLR2 coding domain as follows: TLR2 Mut1, TLR2 Mut2, and TLR2 Mut3 (fragments containing solely one m^6^A site in the 5’UTR, CDS and 3’UTR, respectively) (Fig. 5F). Only reporters containing the wild-type CDS segment of YTHDF1 but not the 3’UTR or the 5’UTR of TLR2 showed substantially increased luciferase activity. This increase was eliminated when the CDS m^6^A consensus sites were mutated (Fig. 5G). Puromycin dihydrochloride (a protein synthesis inhibitor) was used to evaluate YTHDF1-associated TLR2 mRNA translation. The ELISA results showed that TLR2 protein levels were increased or decreased in puromycin-treated FaDu cells in response to YTHDF1 overexpression or knockdown, respectively (Fig. 5H). Additionally, overexpression of ALKBH5 impaired YTHDF1-mediated translation of TLR2 (Fig. 5H). Consistently, previously reported RIP-seq data (GSE130171) from the m^6^A2 Target Database (http://m6a2target.canceromics.org) suggested that TLR2 mRNA could be regulated through m^6^A-dependent translation. These findings indicate that m^6^A modification controls TLR2 mRNA translation associated with YTHDF1.

Finally, we investigated whether YTHDF1 maintained HPSCC tumor progression via TLR2. TLR2 knockdown suppressed the YTHDF1-mediated changes in cell viability, as determined by the EdU assay and CCK8 assay (Fig. 5I, J); proliferation, as determined by colony formation (Fig. 5K); migration, as determined by wound healing assays (Supplementary Fig. S5D)**;** and YTHDF1-mediated inhibition of apoptosis, as determined by flow cytometry and TLR2-targeted gene expression (Fig. 5L, M). This finding revealed that YTHDF1 stimulates TLR2 mRNA translation, promoting cellular growth and migration and inhibiting apoptosis in HPSCC cells.

### Knockdown of ALKBH5 improves TLR2 mRNA stability via an IGF2BP2-dependent pathway

The impact of m^6^A alterations on TLR2 mRNA was then evaluated. The m^6^A readers YTHDF2, YTHDF3 and YTHDC2 are responsible for mRNA decay. However, knockdown of YTHDF2, YTHDF3 or YTHDC2 did not affect the mRNA levels of TLR2 (Supplementary Fig. S6A). Insulin-like growth factor 2 mRNA binding protein (IGF2BP1/2/3) has been reported to recognize and decay m^6^A-modified mRNA. qPCR showed that knockdown of IGF2BP2 but not IGF2BP1/3 significantly reduced the mRNA level of TLR2 (Supplementary Fig. S6B).

**Fig. 6:**
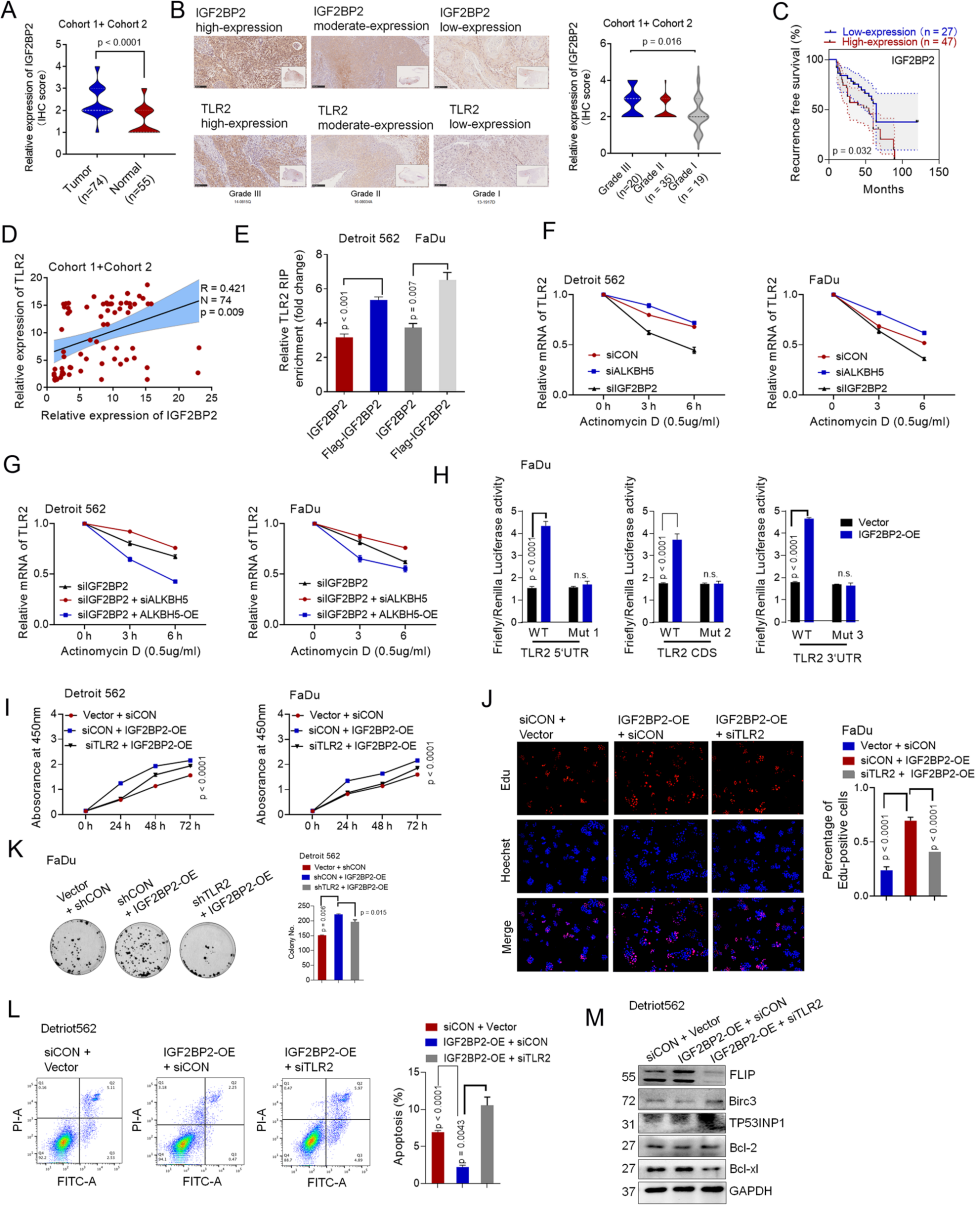
Knockdown of ALKBH5 improves TLR2 mRNA stability via an IGF2BP2-dependent pathway. **A** The expression of IGF2BP2 was analyzed by IHC in HPSCC tumor and paired normal tissue. **B** The expression of IGF2BP2 was evaluated in various grades of HPSCC tissues by IHC. **C** High IGF2BP2 expression was associated with poorer overall survival for HPSCC patients (*p* = 0.0166). **D** The correlation between IGF2BP2 and TLR2 was positive in HPSCC patients in cohort 1 + cohort 2. **E** The interaction between IGF2BP2 and TLR2 mRNA was determined by RIP assays. **F** TLR2 mRNA degradation was detected by qPCR in Detroit 562 and FaDu cells treated with actinomycin D and transfected with control or siRNA ALKBH5 and siIGF2BP2. **G** TLR2 mRNA degradation was detected by qPCR in Detroit 562 and FaDu cells treated with actinomycin D and transfected with IGF2BP2 siRNA, cotransfected with siIGF2BP2 and ALKBH5-WT plasmid, or cotransfected with IGF2BP2 siRNA and ALKBH5 siRNA. **H** Relative luciferase activity of WT or TLR2-Mut1/2/3 luciferase reporters in Detroit 562 cells transfected with control or IGF2BP2-WT plasmids. The results are shown in the form of firefly luciferase activity relative to Renilla luciferase activity. Cellular growth was analyzed by CCK8 assay (**I**), EdU staining (**J**) and colony formation assays (**K**) in Detroit 562 and FaDu cells transfected with IGF2BP2-WT plasmids or cotransfected with TLR2 siRNA and IGF2BP2-WT plasmids. **L** Representative images and quantification of flow cytometric analysis of apoptosis in Detroit 562 cells transfected with IGF2BP2-WT plasmids or cotransfected with TLR2 siRNA and IGF2BP2-WT plasmids. **M** The relative expression of TLR2-targeted genes was analyzed by western blotting in Detroit 562 cells transfected with IGF2BP2-WT plasmids or cotransfected with TLR2 siRNA and IGF2BP2-WT plasmids. Figures 4I, 5I and 6J share the same group, Vector + CON siRNA.

The protein levels of IGF2BP2 were higher in the HPSCC tumors in **Cohort 1** and **Cohort 2** than in the paired normal tissues, as determined by IHC assays (Fig. 6A). IGF2BP2 also gradually increased with increasing HPSCC tumor grade (Fig. 6B). The prognosis of patients with low IGF2BP2 expression was better than that of patients with high IGF2BP2 expression who received adjuvant platinum-based CCT or RT treatments (Fig. 6C). Spearman rank correlation analysis showed a positive correlation between IGF2BP2 and TLR2 mRNA expression (Fig. 6D). Moreover, this positive correlation with the expression of TLR2 was not observed in IGF2BP1 or IGF2BP3 (Supplementary Fig. S6C, D). Furthermore, RIP-qPCR assays validated that IGF2BP2 directly bound to TLR2 mRNA (Fig. 6E, Supplementary Fig. S6E).

To explore whether TLR2 mRNA might be subsequently degraded by IGF2BP2, we performed an RNA synthesis assay with actinomycin D. With increasing actinomycin D treatment time, TLR2 mRNA degradation was significantly increased or decreased in cells transfected with siRNA IGF2BP2 or ALKBH5, respectively (Fig. 6F). Furthermore, TLR2 mRNA degradation was increased in HPSCC cells that were cotransfected with ALKBH5-WT but were decreased in cells that were transfected with shALKBH5 (Fig. 6G).

In addition, dual-luciferase assays demonstrated that ectopic IGF2BP2 strongly inhibited luciferase activity in reporters containing a wild-type 5’UTR, CDS, and 3′UTR segment of TLR2. Mutating the m^6^A consensus sites of the 5’UTR, CDS, and 3′UTR abrogated the increased expression induced by IGF2BP2, suggesting m^6^A-dependent regulation of TLR2 expression by IGF2BP2 (Fig. 6H). Consistent with previously reported RIP-seq and CLIP-seq data (GSE157052, GSE168565) from the m6A2 Target Database (http://m6a2target.canceromics.org), we also identified that TLR2 mRNA stability could be regulated by m^6^A readers on the 3’UTR, exons or TSS sites.

We then investigated whether IGF2BP2 affects HPSCC cells via TLR2. As expected, knockdown of TLR2 inhibited IGF2BP2-induced cell growth, proliferation (Fig. 6I, J) and migration (Supplementary Fig. S6F). Silencing TLR2 increased the apoptosis rate, which was suppressed by IGF2BP2 overexpression (Fig. 6L). Overall, we demonstrated that ALKBH5 reduced the growth and proliferation of tumors and promoted apoptosis by decreasing the expression and function of TLR2 in a YTHDF1/IGFBP2–dependent manner.

## Discussion

HPSCC is an extremely aggressive malignancy that has a poor prognosis. Strategies to sensitize cells to apoptosis are frequently requested. Generating therapeutic agents with variable mechanisms of action to induce apoptosis in HPSCC cells are urgently required [35]. The induction of apoptosis remains a potential therapeutic option in the presence of strategies to overcome redundant antiapoptotic signals [35].

Recent studies have indicated the importance of m^6^A in treatments linked to DNA damage responses, such as radiotherapy, chemotherapy and therapies associated with DNA damage repair [16]. First, we found that m^6^A methylation was upregulated in tumor tissue. Correspondingly, the protein levels of the m^6^A demethylase ALKBH5 were reduced in HPSCC patients. Multiple biological functions become disordered when ALKBH5 loses or gains function [17]. ALKBH5 is dysregulated and plays biological and pharmacological roles in human cancers [36–38]. Biochemical analyses demonstrated that ALKBH5 deficiency increased cell growth and proliferation in vitro and in vivo. Additionally, we found that knocking down ALKBH5 increased migration and invasion, as determined by wound healing and Transwell assays and bone metastasis in vivo. We suggested that ALKBH5 may participate in apoptosis and EMT inhibition. Previous reports have shown that ALKBH5 has weak DNA repair activity to demethylate DNA 3-methylcytosine [39]. Similarly, ALKBH5 overexpression promoted spontaneous apoptosis, as shown by flow cytometry. Western blotting showed that knocking down ALKBH5 downregulated the protein levels of apoptotic markers and upregulated those of antiapoptotic markers. Apoptosis is a form of programmed cell death that is regulated by the Bcl-2 and caspase families of proteins [40]. Detection of BCL2 and cleaved caspase-3 is therefore considered a reliable marker for cells that are dying or have died via apoptosis [41]. Hypertonicity-induced functional loss of MCL-1 renders BCL-XL a synthetically lethal target in head and neck squamous cell carcinoma (HNSCC), and inhibiting BCL-XL efficiently kills HNSCC cells that respond poorly to conventional therapies [42]. Our results show that apoptotic markers (cleaved caspase 3, cleaved PARP, PUMA-23 kDa, Bad and Bax) were decreased and antiapoptotic markers (Bcl-2, Bcl-xL, Mcl-1) were increased in ALKBH5-silenced cells. We believe that ALKBH5, which is an important m^6^A demethylase, exerts additional effects on pathways other than apoptosis, and these pathways contain multiple cell signal molecules that coordinate to regulate oncogenic behaviors. However, in the current study, we did not clarify the mechanism of migration, invasion and metastasis in depth.

The transcriptome-wide m^6^A-seq analysis, subsequent validation and functional studies suggested that m^6^A of TLR2 mRNA was a target of ALKBH5; its expression is upregulated in HPSCC. Previous studies have highlighted the importance of TLRs in the pathogenesis of certain cancers, including head and neck cancers. Studies have shown that microbial pattern recognition by TLR2 in various epithelial cells is upregulated in premalignant lesions and in malignant cells and can activate oncogenic pathways [43]. TLR2 is also a functional receptor that plays a direct protumorigenic role in head and neck cancers [44, 45]. Here, we demonstrated that TLR2 could inhibit apoptosis in HPSCC, which is one of most lethal subgroups of head and neck cancer. In our study, the absence of TLR2 impaired the proliferation and invasion of HPSCC cells, while TLR2 was elevated in HPSCC tumor tissue, and a high TLR2 level was indicative of poor clinical outcomes for HPSCC. In vitro and in vivo experiments confirmed that ALKBH5 inhibited TLR2-induced cell proliferation and promoted TLR2-induced apoptosis. Indeed, the mechanism should be examined on bulk and single-cell RNA-seq data [46]. Single-cell RNA-seq data may expand the relevance of our findings on the role of cell populations and provide an opportunity to examine cell-specific expression profiles, which further complicates the examination of true cell-to-cell expression variation. In the future, we can use deconvolution approaches to analyze bulk RNA-seq data and use single-cell RNA-seq measurements to infer sample-level cell-type-specific gene expression profiles.

**Fig. 7:**
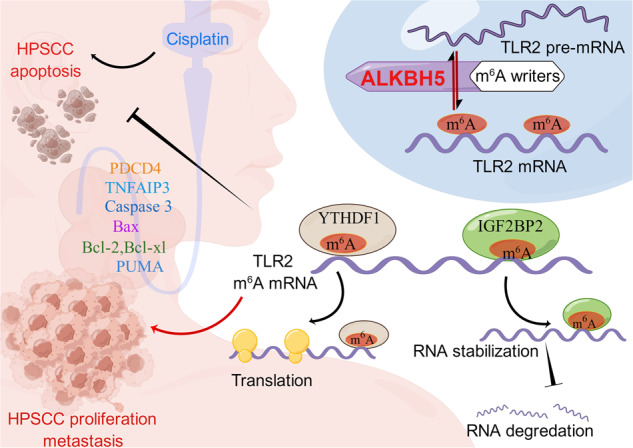
. A working model (by Figdraw IDTIOUU31893) summarizing the mechanism by which the demethylase ALKBH5 induces posttranscriptional activation of TLR2 mRNA to inhibit proliferation and promote apoptosis in HPSCC cells in an m^6^A-YTHDF1/IGF2BP2-dependent manner.

Mechanistically, ALKBH5 was downregulated in HPSCC, which increased m^6^A modification of TLR2 and initially promoted HPSCC tumorigenesis and development. ALKBH5 decreases the m^6^A level of TLR2 at the CDS and 3’ and 5′ untranslated regions (UTRs), which in turn is recognized by the readers IGF2BP2 and YTHDF1 and leads to the upregulation of mRNA and protein levels. Previous reports have shown that human neutrophils (PMNs), which are engaged in the early phase of the antitumor response, express TLR2 and TLR6, which can modulate Bcl-2 family proteins, regulating the intrinsic apoptotic pathway in these cells [45]. Correspondingly, we found that TLR2 modulated the expression of the Bcl-2 family, cleaved caspase 3, Bax, and PUMA. Biochemical analysis showed that TLR2-targeted apoptotic markers and antiapoptotic markers were influenced by expression of the m^6^A demethylase ALKBH5 and the m^6^A readers YTHDF1/IGF2BP2. Due to recognition by different m^6^A readers, m^6^A-modified mRNA have different fates. m^6^A methylation directs mRNAs to distinct fates by affecting mRNA stability, the splicing of transcripts to preserve the cell type-specific proteome, translation and decay in processes such as cell differentiation, embryonic development and stress responses [8]. To achieve its biological effects, m^6^A alteration requires preferential recognition by certain binding proteins [31, 47, 48]. In this study, we showed that m^6^A modification is mediated by YTHDF1/IGF2BP2 to modulate TLR2. The m^6^A reader YTHDF1 recognizes m^6^A located in the CDS, while IGF2BP2 recognizes m^6^A in the 5/3’ UTR and CDS. Our luciferase reporter/mutagenesis assays indicated that the m^6^A sites in the CDS, 3’UTR and 5’UTR of TLR2 were essential for IGF2BP2, and the CDS of TLR2 was essential for YTHDF1. IGF2BP2 facilitated TLR2 mRNA stabilization, whereas YTHDF1 promoted TLR2 mRNA translation; both of these activities were regulated by m^6^A methylation (Fig. 7). In contrast to ALKBH5, YTHDF1 and IGF2BP2 were consistently expressed in the presence of TLR2 in HPSCC tumors. IGF2BP2 and YTHDF1 exerted oncogenic effects on tumor growth and apoptosis inhibition. IGF2BP2 and YTHDF1 overexpression inhibited apoptosis induced by TLR2 silencing. Therefore, from a therapeutic perspective, profiling the m^6^A modification pattern, particularly in ALKBH5-deficient HPSCC, may allow the development of treatments targeting RNA methyltransferases in cancer.

These results indicate m^6^A regulation of TLR2-induced apoptosis by balancing the functions of ALKBH5, YTHDF1 and IGF2BP2 in HPSCC tumors. There are still several limitations. The underlying mechanisms of TLR2 downstream partners in the context of apoptosis-based modulation are not fully characterized. Further research is needed to investigate the direct correlation between TLR2 and downstream apoptosis signaling, as well as whether the ALKBH5/TLR2 axis can alter the susceptibility of HPSCC cells to apoptotic promoters through m^6^A modification in vitro and in vivo. Further mechanistic understanding of TLR2-induced cancer cell biology and apoptosis inhibition in cellular regulation will enable the identification of novel therapeutic targets. Furthermore, other TLR signaling pathways might be controlled by m^6^A methylation and numerous m^6^A readers and affect a variety of other biological processes.

## Conclusion

Here, we demonstrate that ALKBH5 reduces the m^6^A modification of TLR2, which can be recognized by the m^6^A readers IGF2BP2 and YTHDF1. IGF2BP2 facilitates TLR2 mRNA decay, whereas YTHDF1 promotes TLR2 mRNA translation. In addition, ALKBH5 suppresses the development and proliferation of HPSCC while promoting apoptosis by inhibiting the expression and function of TLR2. This study suggests the crucial involvement of TLR2 and a novel function of m^6^A demethylation of RNA in HPSCC. In HPSCC, ALKBH5 inhibitors warrant more clinical research.

## Materials and methods

### Patient specimens

Two cohorts of patients with HPSCC from separate medical centers (32 cases from The Second Affiliated Hospital of Nanchang University Medical College + 42 cases from Sir Run Run Shaw Hospital) who received surgical treatment and regular medical surveillance between 2012 and 2020 were included (Additional file 1).

**Cohort 1** contained 14 HPSCC patients who received cisplatin-based induced chemotherapy followed by radical radiation chemotherapy or cisplatin-based induced chemotherapy followed by curative surgery and CRT/RT [34]. In this study, m^6^A levels in preoperative biopsy tissue was examined.

**Cohort 2** contained 60 HPSCC patients who underwent CRT, definitive RT or surgery followed by CRT/RT. Patient tissue specimens included freshly resected malignant and normal tissues, as well as paraffin-embedded tissues acquired from Sir Run Run Shaw Hospital, Zhejiang University College of Medicine (Hangzhou, China). The preoperative biopsy tissue and surgical specimens, including cancerous and normal tissues, were stored and numbered in the Sir Run Run Shaw Hospital biological specimen bank.

Each patient provided written informed consent consistent with the criteria of the Declaration of Helsinki (No. 20200522-39).

### Cell culture

FaDu (ATCC@ HTB-43™) cells and HPSCC Detroit562 (ATCC@CCL138™) cells were purchased from the American Type Culture Collection (Manassas, VA) in 2020. Detriot562 cells are a metastatic pharyngeal SCC cell line acquired from the hydrothorax [49]. FaDu cells are a primary SCC cell line from the hypopharynx [49]. In vivo, both cells lines are highly invasive. Both cell lines were cultured in Dulbecco’s modified Eagle’s medium (DMEM, Gibco, cat. 10565-018, Life Technologies) supplemented with 10% fetal bovine serum and antibiotics (100 U/ml penicillin and 100 mg/ml streptomycin). The cells were collected with 0.25% trypsin/0.1% EDTA (Wisent) when they reached 80% confluence. The cells were kept in a humidified 37 °C and 5% CO_2_ environment, and the medium was changed every three days.

### Global m^6^A measurement

Global m^6^A levels in mRNA were measured with an EpiQuik m^6^A RNA Methylation Quantification Kit (Colorimetric) (Epigentek, Farmingdale, NY) according to the manufacturer’s protocols [29].

### m^6^A sequencing (m^6^A-seq, MeRIP-seq) and RNA sequencing (RNA-seq)

Total RNA was extracted with TRIzol reagent (Invitrogen, CA, USA) according to the manufacturer’s instructions. Using a Bioanalyzer 2100 and RNA 6000 Nano LabChip Kit (Agilent, CA, USA), the quality and quantity of total RNA were determined to have an RIN > 7.0. To separate poly(A) mRNA, approximately 200 µg of total RNA was mixed with poly(T) oligo-coupled magnetic beads (Invitrogen). After the poly(A) mRNA fractions were purified, they were fragmented into ~100-nt-long oligonucleotide sequences by heating with divalent cations. The cleaved RNA fragments were then incubated for 2 h at 4 °C with an m^6^A-specific antibody (No. 202003, Synaptic Systems, Germany) in IP buffer (50 mM Tris-HCl, 750 mM NaCl and 0.5% Igepal CA-630) supplemented with BSA (0.5 μg μl−1). The mixture was then incubated with protein A beads and eluted with elution buffer (1 × IP buffer and 6.7 mM m^6^A). The eluted RNA was precipitated with 75% ethanol, and the eluted m^6^A-containing fragments (IP) and untreated input control fragments were used to form the final cDNA library according to a strand-specific library preparation strategy using the dUTP method. The average size of the inserts in the paired-end libraries was ~100 ± 50 bp. Then, paired-end 2 × 150 bp sequencing was performed on an Illumina NovaSeq™ 6000 platform at LC-BIO Biotech Ltd. (Hangzhou, China) according to the manufacturer’s suggested procedure. *[The specific method was described in the paper]* [29, 34].

### Anti-m^6^A immunoprecipitation and RNA immunoprecipitation (RIP)

The cell pellet was resuspended in IP lysis buffer (150 mM KCl, 25 mM Tris (pH 7.4), 5 mM EDTA, 0.5 mM DTT, 0.5% NP40, 1× protease inhibitor, and 1 U/μl RNase inhibitor) after being rinsed twice with PBS. After 30 min of incubation, the lysate was extracted by centrifugation at 12,000 × g for 10 min. Before overnight incubation at 4 °C, antibodies and 40 μl of protein G beads (Invitrogen, USA) were added to the lysates. The coprecipitated RNAs were extracted using TRIzol reagent and ethanol-precipitated with glycogen (Invitrogen, USA) after three washes with wash buffer (150 mM KCl, 25 mM Tris (pH 7.4), 5 mM EDTA, 0.5 mM DTT, and 0.5% NP40). The enriched RNA was normalized to that of the IgG immunoprecipitate. *[The specific method is described in the paper]* [29, 34].

### Lentivirus packaging and generation of stable cell lines

The lentiviruses were produced in accordance with the manufacturer’s instructions *[The specific method is described in the paper]* [29, 34].

### Cell transfection, plasmids, and RNA knockdown

shRNA against ALKBH5 was constructed using the GV344 vector (hU6-MCS-Ubiquitin-firefly_Luciferase-IRES-puromycin) and GV493 (hU6-MCS-CBh-gcGFP-IRES-puromycin); shRNA against YTHDF1 was constructed using the GV493 vector. The TLR2-WT plasmid and TLR2-Mut (5’UTR, 3’UTR, CDS) were amplified by qPCR and cloned into GV272 vectors. The IGF2BP1/2/3- and YTHDF1-overexpressing plasmids were cloned into the CMV6-MCS-3flag-SV40-Neomycin vector. The ALKBH5-overexpressing plasmid was cloned into GV492 (Ubi-MCS-3FLAG-CBh-gcGFP-IRES-puromycin). TLR2-overexpressing plasmids were cloned into GV712 (CMV enhancer-MCS-SV40-puromycin).

Gene Chemistry (Shanghai, China) generated the recombinant plasmids. All constructs were sequence-verified, and comprehensive cloning information is available upon request. Overexpression plasmid, shRNA and siRNA of the indicated genes were transfected into cells with Lipofectamine 2000 (Invitrogen, Carlsbad, CA, USA) for plasmids and Lipofectamine RNAiMAX (Invitrogen, Carlsbad, CA, USA) for siRNAs [10]. The cells were analyzed at 48–72 h.

### Cell growth, apoptosis, cell proliferation, migration, and invasion assays

Cell viability was analyzed by adding 10% CCK8 (Dojindo, Japan) to infected cells (5 × 10^3^–1 × 10^4^ per well) in 96-well plates and incubation at 37 °C for 30–45 min as indicated. The absorbance of each well was measured using a microplate reader set to 450 nm. For cells treated with temporal transfection, we first used siRNA to knockdown TLR expression and plasmids to overexpress the target genes temporally in 12-well or 6-well plates. The cells were analyzed at 48–72 h. After 6 h, we digested the cells and transferred them into 96-well plates. After 12–24 h, cell viability was detected by adding 10% CCK8 (Dojindo, Japan) into the infected cells and incubating the cells at 37 °C for 30 min-45 min or 0, 24, 48, or 72 h. Therefore, “Time 0 h” is 18–30 h after transfection and “Time 72 h” is 90–102 h after transfection. *[The specific method is described in the paper]* [29, 34].

Apoptosis was measured by pooling the adherent and floating cells after the indicated treatments and staining the cells with FITC-Annexin V and PI. The cells were sorted using FACS. The cells in the third and fourth quadrants were counted.

### Histology and immunohistochemical staining

The slides were washed with PBS and treated for 60 min with labeled polymer-HRP (GTVison TMIII, GK500710), followed by incubation with dilutions of ALKBH5 (1:100), TLR2 (1:100), Caspase 3 (1:200), Bax (1:150), FLIP (1:150), PDCD4 (1:150), and IGF2BP2 (1:100) at 4 °C overnight. The staining level was rated as 0 (negative), 1 (weak), 2 (moderate), or 3 (strong). The extent of staining was scored as follows: 0 (0%), 1 (1–25%), 2 (26–50%), 3 (51–75%), and 4 (76–100%). The results were quantified by ImageJ IHC profiler [50]. at 5X and 40X magnification *[The specific method is described in the paper]* [29, 34].

### Luciferase assay

The methods were described in our previous studies [29, 34]. Gene Chemistry (Shanghai, China) generated portions of the TLR2-5’UTR, CDS, and 3’UTR, which included both wild-type and mutant m6A motifs (m6A was substituted with T). Using a dual-luciferase reporter assay system (GV272), the firefly luciferase and Renilla luciferase activities in each well were measured.

### Mutagenesis assays

The TLR2-3’UTR, CDS, and 5’UTR with wild-type or mutant sequences (m^6^A was replaced by T) were inserted downstream of the GV272 vector. The methods were described in our previous studies [29, 34].

TLR2 5’UTR with wild-type m^6^A sites

AAGCACTAATGGACTAAGACAAAAAGATTCCATTATGAAAGTGAAAAGGCAACCGCAGAG

TGGCAGAAGCTTTTGGAGATGATGAATATGTTTACTTCCTGGATTGCGGTGATGATACCA

TLR2 5’UTR with mutant m^6^A sites

AAGCACTAATGGATTAAGACAAAAAGATTCCATTATGAAAGTGAAAAGGCAACCGCAGAG

TGGCAGAAGCTTTTGGAGATGATGAATATGTTTACTTCCTGGATTGCGGTGATGATACCA

TLR2 3’UTR with wild-type m^6^A sites

AGAACCCATGGATATAGAGGGCCAACTGTAATCTGTAGCAACTGGCTTAGTTCATTAGGA

AACAGCACAAATGAACTTAAGATTCTCAATGACTGTGTCATTCTTTCTTCCTGCTAAGAG

TLR2 3’UTR with mutant m^6^A sites

AGAACCCATGGATATAGAGGGCCAACTGTAATCTGTAGCAACTGGCTTAGTTCATTAGGA

AACAGCACAAATGATCTTAAGATTCTCAATGACTGTGTCATTCTTTCTTCCTGCTAAGAG

TLR2 CDS with wild-type m^6^A sites

AGTAAGAATAGTTTTCATTCTATGCCTGAAACTTGTCAGTGGCCAGAAAAGATGAAATAT

TTGAACTTATCCAGCACACGAATACACAGTGTAACAGGCTGCATTCCCAAGACACTGGAA

ATTTTAGATGTTAGCAACAACAATCTCAATTTATTTTCTTTGAATTTGCCGCAACTCAAA

GAACTTTATATTTCCAGAAATAAGTTGATGACTCTACCAGATGCCTCCCTCTTACCCATG

TLR2 CDS with mutant m^6^A sites

AGTAAGAATAGTTTTCATTCTATGCCTGAAACTTGTCAGTGGCCAGAAAAGATGAAATAT

TTGTACTTATCCAGCACACGAATACACAGTGTAACAGGCTGCATTCCCAAGACACTGGAA

ATTTTAGATGTTAGCAACAACAATCTCAATTTATTTTCTTTGAATTTGCCGCAACTCAAA

GTACTTTATATTTCCAGAAATAAGTTGATGACTCTACCAGATGCCTCCCTCTTACCCATG

### Analysis of mRNA stability

To measure mRNA stability, the cells were treated with 5 g/mL actinomycin D (Sigma, St. Louis, MO, USA) at a final concentration of 5 μg/mL for 0, 3, or 6 h. The cells were collected, and the RNA was reverse transcribed. qPCR was performed to measure the target mRNA levels.

### Enzyme -linked immunosorbent assay (ELISA) analysis of protein stability

Transfection of the appropriate shRNAs and subsequent culture of FaDu and Detroit 562 cells for the specified times yielded the expected results. TLR2 protein expression was measured with a Human TLR-2 ELISA Kit (Thermo Fisher, EH459RB) according to the manufacturer’s instructions.

### Western blot analysis

Western blot analysis was carried out as previously described. The antibodies used in this work are presented in the supplementary information (Additional file 2).

### Total RNA extraction and quantitative real-time PCR (qPCR)

We used the 2 − ΔΔCt method to quantify the differences in expression levels. HPRT1 was used as an internal control. The primers for qPCR are as follows *[The specific method is described in the paper]* [29, 34]. (Additional file 3)**:**

HPRT1-FTGACACTGGCAAAACAATGCA

HPRT1-RGGTCCTTTTCACCAGCAAGCT

ALKBH5-FCGGCGAAGGCTACACTTACG

ALKBH5-RCCACCAGCTTTTGGATCACCA

YTHDF1-FATACCTCACCACCTACGGACA

YTHDF1-RGTGCTGATAGATGTTGTTCCCC

IGF2BP2-FAGCTAAGCGGGCATCAGTTTG

IGF2BP2-RCCGCAGCGGGAAATCAATCT

IGF2BP3-FTATATCGGAAACCTCAGCGAGA

IGF2BP3-RGGACCGAGTGCTCAACTTCT

IGF2BP1-FCAAAGGAGCCGGAAAATTCAAAT

IGF2BP1-RCGTCTCACTCTCGGTGTTCA

TLR2-F ATCCTCCAATCAGGCTTCTCT

TLR2-R GGACAGGTCAAGGCTTTTTACA

### Mouse treatment and tumor biology studies

The methods were described in our previous studies [29, 34]. HPSCC FaDu or Detroit 562 cells (1 × 10^6^ cells) expressing vector control and construct lentiviruses were subcutaneously injected into the right flanks of 4-week-old male nude mice. Tumor diameters and body weight were recorded every 3 days for 1–6 weeks. Detroit 562 and FaDu cells (1 × 10^6^ cells) were subcutaneously injected into the right flanks of 4-week-old male nude mice. The specific method is described in the paper [29, 34].

The mice were housed in standard cages and fed standard food and water in a room with regulated lighting and temperature. All animal experiments were conducted in compliance with the Institute of Laboratory Animal Resources guidelines with the approval of the University Committee on the Use and Care of Animals at Zhejiang University (SRRSH20200567).

### Statistical analysis

OmicStudio tools at https://www.omicstudio.cn/tool were used for bioinformatic analysis. All statistical analyses were performed with GraphPad Prism version 9 (GraphPad Software, CA) for Windows. Unpaired two-tailed Student’s *t* tests, analysis of variance (ANOVA) or Spearman rank correlation were used to determine statistical significance. The Kaplan–Meier method was used to evaluate recurrence-free survival. The data are presented as the means ± SD. Each experiment was repeated independently at least three times.

### Supplementary information

Additional file 1

Additional file 2

Additional file 3

Additional file 4

Additional file 5

Additional file 6

original data

## Data Availability

The data generated in this study are available upon reasonable request from the corresponding author.
