# Supplementary material for: ALKBH5 promotes hypopharyngeal squamous cell carcinoma apoptosis by targeting TLR2 in a YTHDF1/IGF2BP2-mediated manner
Source: Cell Death Discov. 2023 Aug 23;9:308. doi: 10.1038/s41420-023-01589-6 (PMC10447508; doi:10.1038/s41420-023-01589-6)
Supplement: Supplementary file 1 — Additional file 1 [file 41420_2023_1589_MOESM1_ESM.docx]

| **Characteristic** | **Cohort 1 (N=14)** |  | **Cohort 2 (N=60)** |  |
| --- | --- | --- | --- | --- |
|  | **No. of patients** | **%** | **No. of patients** | **%** |
| **Sex** |  |  |  |  |
| Female | 0 | 0.00% | 3 | 5.00% |
| Male | 14 | 100.00% | 57 | 95.00% |
| **Age** |  |  |  |  |
| Median (Range) | 60.2 |  | 62.3 |  |
| **Clinical T stage** |  |  |  |  |
| T1 | 0 | 0.00% | 0 | 0.00% |
| T2 | 2 | 14.28% | 15 | 25.00% |
| T3 | 6 | 42.86% | 15 | 25.00% |
| T4 | 6 | 42.86% | 30 | 50.00% |
| **Clinical N stage** |  |  |  |  |
| N0 | 0 | 0.00% | 19 | 31.67% |
| N1 | 2 | 14.29% | 22 | 36.67% |
| N2 | 6 | 42.86% | 7 | 11.67% |
| N3 | 6 | 42.86% | 12 | 20.00% |
| **Smoking History at Diagnosis** |  |  |  |  |
| Never | 0 | 0.00% | 4 | 6.67% |
| Smoker (Current/Former) | 14 | 100.00% | 56 | 93.33% |
| **Disease site** |  |  |  |  |
| piriformis | 6 | 42.86% | 10 | 16.67% |
| pharyngeal wall | 6 | 42.86% | 22 | 36.67% |
| aryepiglottic | 2 | 14.29% | 28 | 46.67% |
| **Cisplatin-based ICT** |  |  |  |  |
| response |  |  |  |  |
| PR/CR | 9 | 64.29% | - |  |
| SD | 5 | 35.71% | - |  |
| Following treatments |  |  |  |  |
| + definitive CRT | 10 | 71.43% | 20 | 33.33% |
| +curative surgery + CRT | 4 | 28.57% | 40 | 66.67% |
| **Differentiation** |  |  |  |  |
| Grade I | 2 | 14.29% | 18 | 30.00% |
| Grade II | 4 | 28.57% | 31 | 51.67% |
| Grade III | 8 | 57.14% | 11 | 18.33% |
| **Expression of ALKBH5 mRNA level** |  |  |  |  |
| low | 8 | 57.14% | 25 | 41.67% |
| high | 6 | 42.86% | 35 | 58.33% |
| **Expression of TLR2 mRNA level** |  |  |  |  |
| low | 4 | 28.57% | 33 | 55.00% |
| high | 10 | 71.43% | 27 | 45.00% |
| **Expression of YTHDF1 mRNA level** |  |  |  |  |
| low | 6 | 42.86% | 26 | 43.33% |
| high | 8 | 57.14% | 34 | 56.67% |
| **Expression of IGF2BP2 mRNA level** |  |  |  |  |
| low | 7 | 50.00% | 20 | 33.33% |
| high | 7 |  | 40 | 66.67% |

**Additional file 1:**

**Table 1: Characteristics of HPSCC patients from Cohort1 and Cohort2.**

**ICT: induction chemotherapy**

**CRT/RT: (chemo)radiation**
