# Supplementary material for: ALKBH5 promotes hypopharyngeal squamous cell carcinoma apoptosis by targeting TLR2 in a YTHDF1/IGF2BP2-mediated manner
Source: Cell Death Discov. 2023 Aug 23;9:308. doi: 10.1038/s41420-023-01589-6 (PMC10447508; doi:10.1038/s41420-023-01589-6)
Supplement: Supplementary file 4 — Additional file 4 [file 41420_2023_1589_MOESM4_ESM.docx]

## Additional file 4

Supplementary Figures


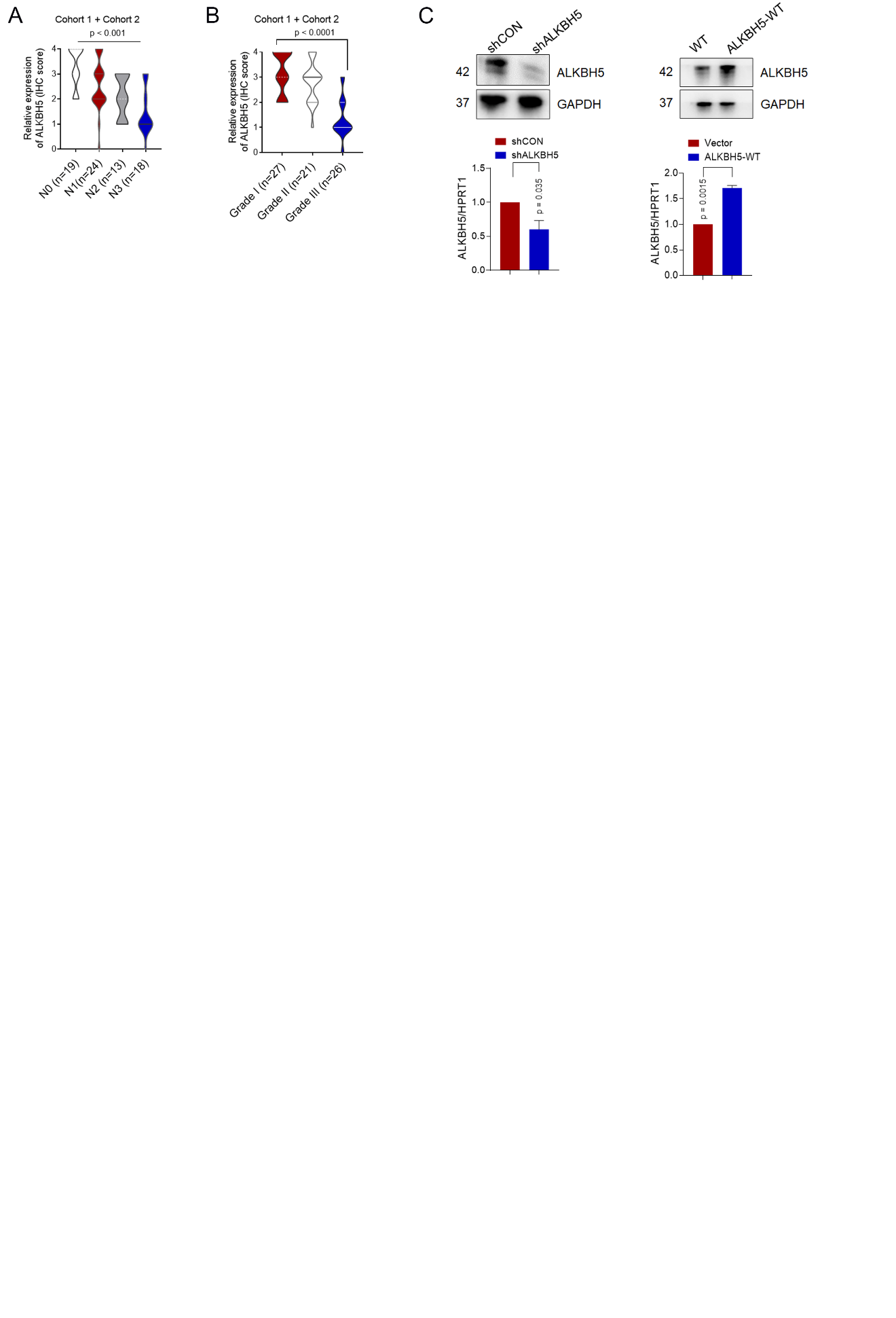


### Supplemental_Fig_S1 ALKBH5 correlated with lymph node metastasis and pathological grades

1. The expression of ALKBH5 was analyzed by IHC assay in the different grades of HPSCC.
2. The correlation of ALKBH5 IHC score and lymph mode stage (AJCC 8th) determined by IHC assay.
3. The transfection efficiency of ALKBH5 shRNA and plasmid was examined by qPCR and Western blot analysis


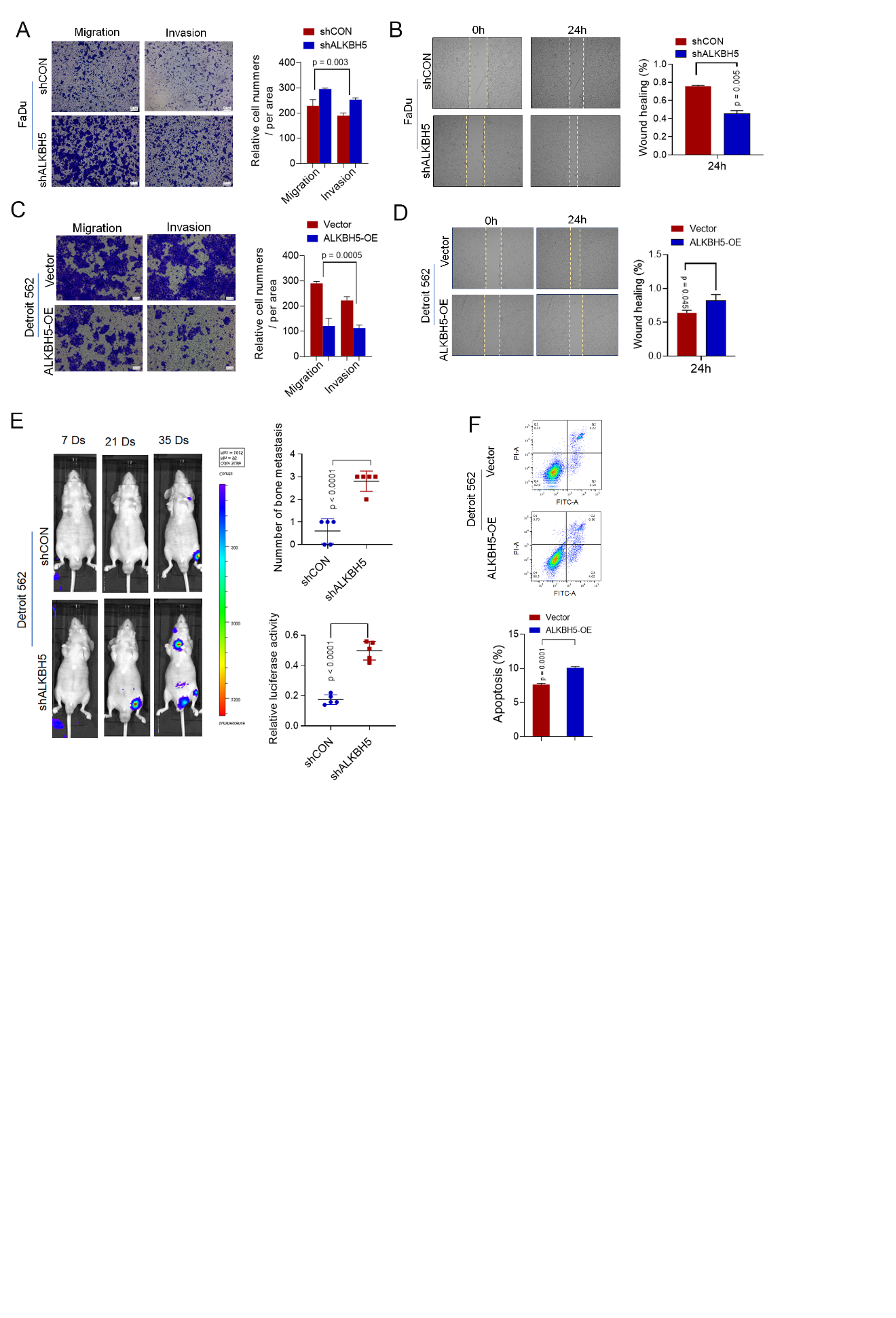


### Supplemental_Fig_S2 Overexpression of ALKBH5 decreased FaDu cells growth

A. B. Representative images and its quantitative analysis of Transwell assays (A) and Wound healing (B) of FaDu cells transfected with control or shALKBH5.

1. D. Representative images and its quantitative analysis of Transwell assays (C) and Wound healing (D) of Detroit 562 cells transfected with control or ALKBH5-WT plasmid.
2. Detroit 562 cells transfected with control or shALKBH5 were injected into mice via tail vain to establish bone metastasis models (n = 3). Representative in vivo images of mice were taken with quantification of luciferase activity and metastasis numbers in the bone region.
3. Representative images and its quantification of Flow cytometry for apoptosis assay of Detroit 562 cells transfected with control or ALKBH5-WT plasmid.


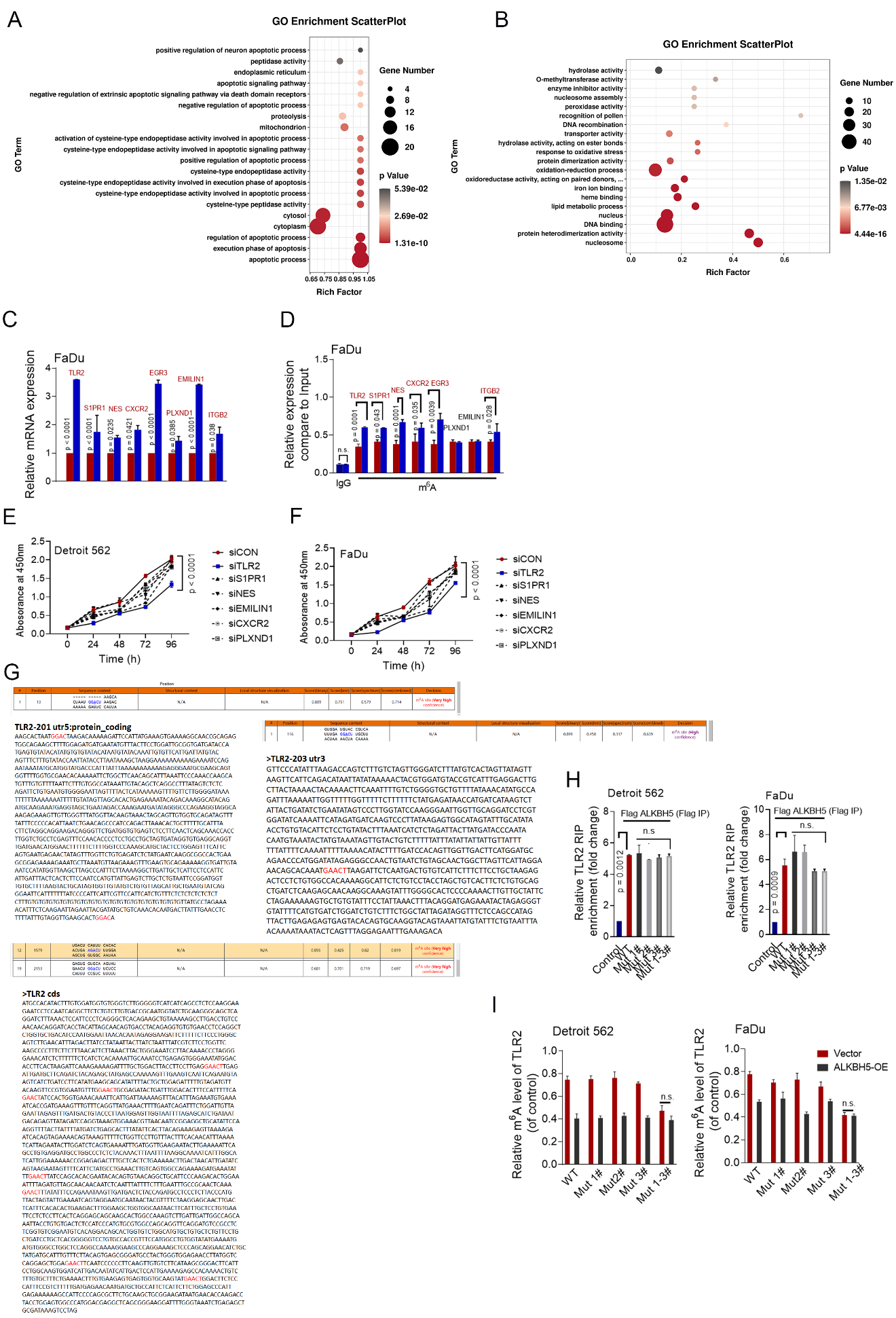


### Supplemental_Fig_S3 ALKBH5 regulated the expression of TLR2 through m^6^A-dependent mechanism

1. B. Gene ontology (GO) term analyses of the significantly hyper-up transcripts(A) and hyper-down transcripts(B) (p <0.05).
2. D. QPCR and MeRIP-qPCR of the levels of indicated potential targets m^6^A modification in control and shALKBH5 FaDu cells.
3. F. The cellular growth of indicated cells was analyzed by CCK8 assay.
4. Sequence analysis of the TLR2 5’-UTR, CDS, 3’-UTR revealed several matches to the 5’-RRACA-3’ (R=G or A) m^6^A consensus sequence.
5. The interaction between ALKBH5 and TLR2 pre-mRNA was detected by RIP.
6. The relative of m^6^A level in TLR2 from co-expression of ALKBH5-WT and TLR2-WT plasmid/Muts# in Detroit 562 and FaDu cells


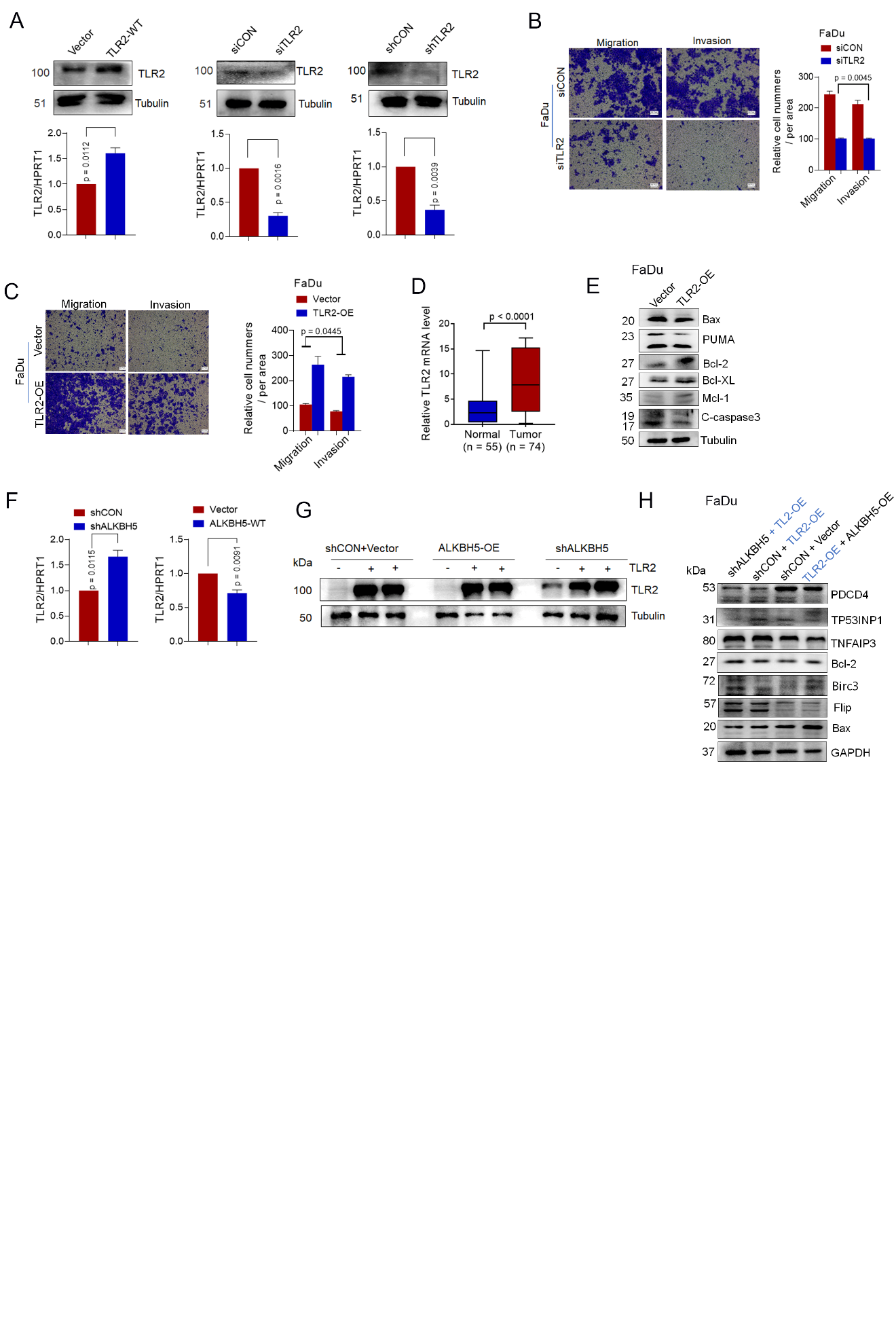


### Supplemental_Fig_S4 Overexpression of TLR2 in ALKBH5-overexpressed and ALKBH5-knockdown HPSCC cells and its function.

1. The transfection efficiency of TLR2 overexpressing plasmid, siRNA TLR2 and shTLR2 were analyzed by Western blot and QPCR assays.
2. Transwell assays of FaDu cells transfected with either a control siRNA or a TLR2 siRNA.
3. Transwell assays of FaDu cells transfected with either control or TLR2-WT plasmid.
4. The mRNA levels of TLR2 were analyzed by QPCR in tumor and their adjacent tissue.
5. The protein levels of apoptosis markers detected in FaDu cells transfected with either control or TLR2-WT plasmid.
6. The expression of TLR2 analyzed by QPCR assays, in FaDu cells transfected with either control or ALKBH5-WT plasmid, either shCON or shALKBH5.
7. The expression of TLR2 analyzed by western blot, in cells transfect with vector+shCON, solely TLR2-WT, shALKBH5+TLR2-WT, ALKBH5-WT+TLR2-WT plasmids. The efficiency of TLR2-WT transfection was duplicated here.
8. The protein levels of previously described TLR2-targeted proteins were detected in indicated FaDu cells


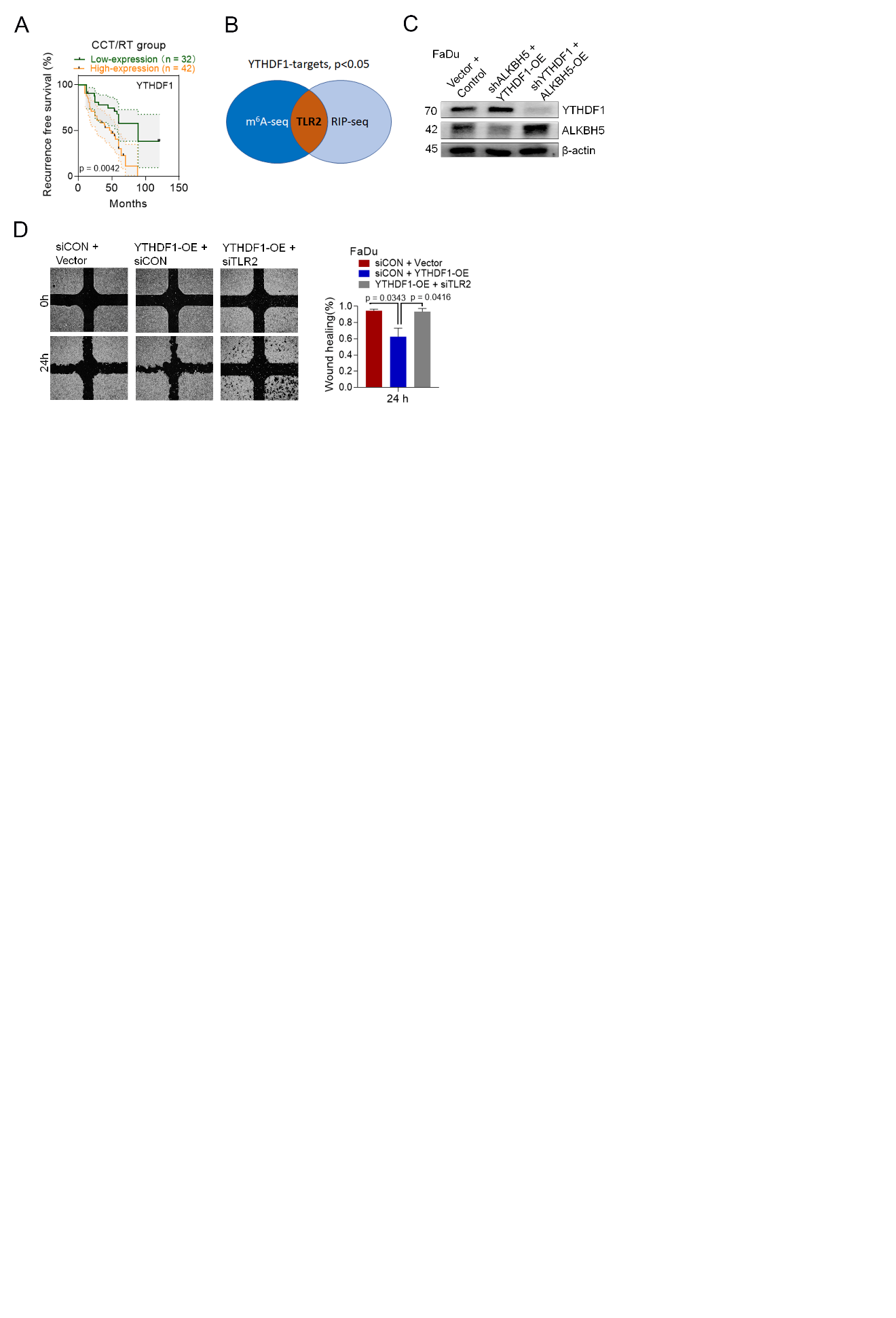


### Supplemental_Fig_S5 YTHDF1 regulated the expression of TLR2 in HPSCC

1. Low expression of YTHDF1 is associated with better prognosis for HPSCC patients who had radiation/ chemoradiation therapy
2. Venn diagram illustrating the overlapping genes identified by m6A-seq and RIP-seq
3. The protein levels of ALKBH5 and YTHDF1 detected in co-expression of ALKBH5-OE and shYTHDF1, YTHDF1-OE and shALKBH5 FaDu cells.
4. The cellular immigration analyzed by wound healing assay.


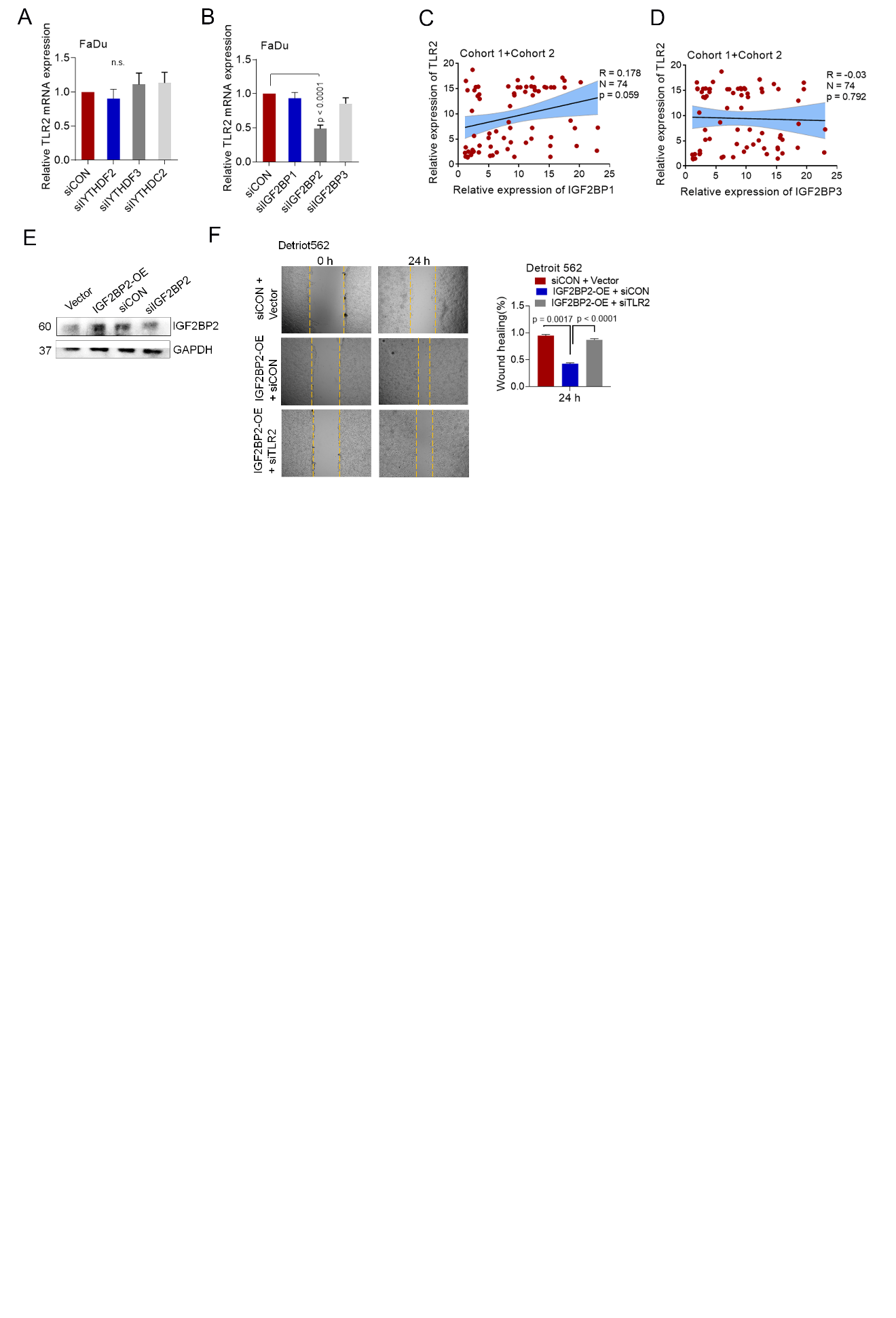


### [Supplemental_Fig_S6](file:///C:\Users\ybjin\Documents\WeChat%20Files\wxid_5905389053622\FileStorage\File\2022-12\figure%20S6.tif) IGF2BP2 regulated the expression of TLR2 in HPSCC.

1. B. The mRNA level of TLR2 detected by QPCR in indicated FaDu cells

C, D. The correlation between IGF2BP1/3 and TLR2 expression analyzed from Cohort 1+ Cohort 2.

1. The transfection efficiency of siRNA IGF1BP2 and IGF2BP2 overexpression confirmed by Western blot
2. The cellular migration analyzed by wound healing assay in Detroit 562 and FaDu cells transfected with IGF2BP2-WT plasmid or co-transfected with TLR2 siRNA and IGF2BP2-WT plasmid.
