# Supplementary material for: ALKBH5 promotes hypopharyngeal squamous cell carcinoma apoptosis by targeting TLR2 in a YTHDF1/IGF2BP2-mediated manner
Source: Cell Death Discov. 2023 Aug 23;9:308. doi: 10.1038/s41420-023-01589-6 (PMC10447508; doi:10.1038/s41420-023-01589-6)
Supplement: Supplementary file 5 — Additional file 5 [file 41420_2023_1589_MOESM5_ESM.docx]

1. Reads density


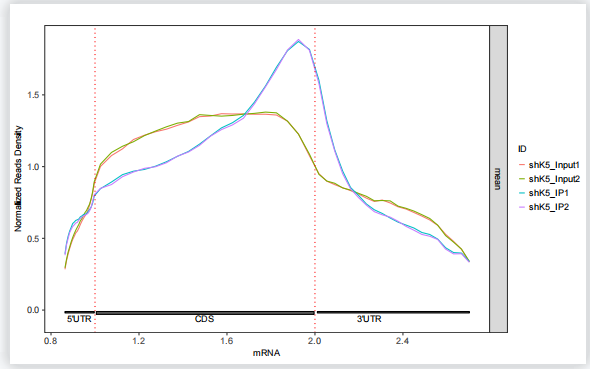


Distribution of reads in mRNAs. Figure shows that the reads are strongly enriched near stop codon in the IP sample compared with the input sample.

1. Peak density


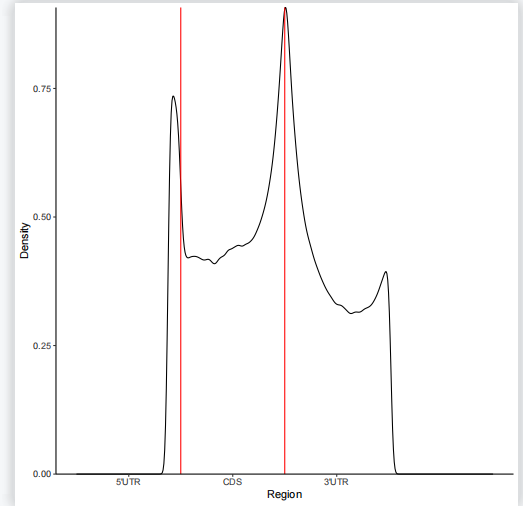


The frequency distribution of m^6^A peaks across the length of mRNA transcripts shown by metagene in samples.

1. principal component analysis (PCA)


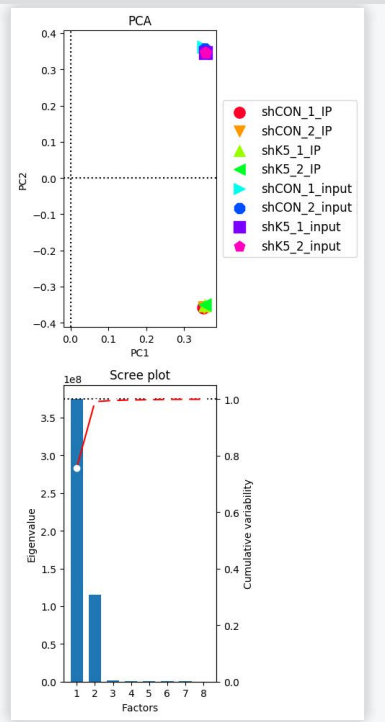


the eigenvalues of the top two principal components;
the Scree plot for the top eight principal components where the bars represent the amount of variability explained by the individual factors and the red line traces the amount of variability is explained by the individual components in a cumulative manner.

1. DistProfile


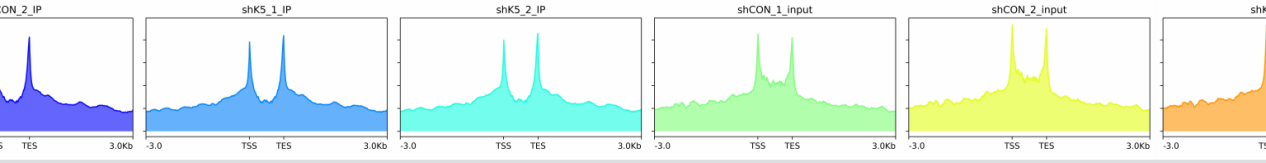


Visualized distribution of reads from 3.0 kb upstream of the transcription start site (TSS) to the transcription end site (TES) on the genome transcript.

1. coverage_By Samples


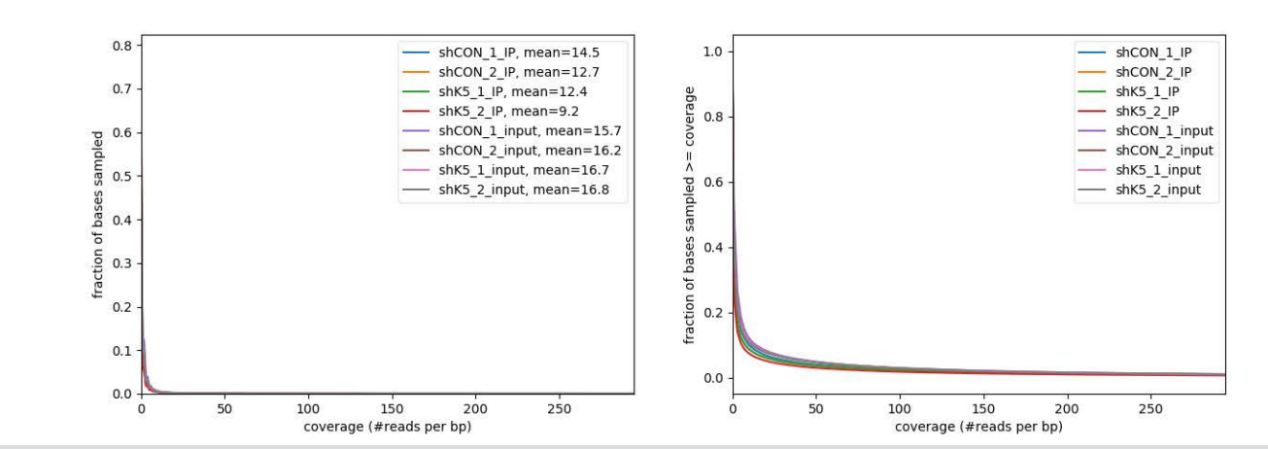
 Frequency distribution of reads coverage in each sample
