# Supplementary material for: ALKBH5 promotes hypopharyngeal squamous cell carcinoma apoptosis by targeting TLR2 in a YTHDF1/IGF2BP2-mediated manner
Source: Cell Death Discov. 2023 Aug 23;9:308. doi: 10.1038/s41420-023-01589-6 (PMC10447508; doi:10.1038/s41420-023-01589-6)
Supplement: Supplementary file 7 — original data [file 41420_2023_1589_MOESM7_ESM.zip › S1C-WB/New Microsoft PowerPoint Presentation.pptx]

## Slide 1
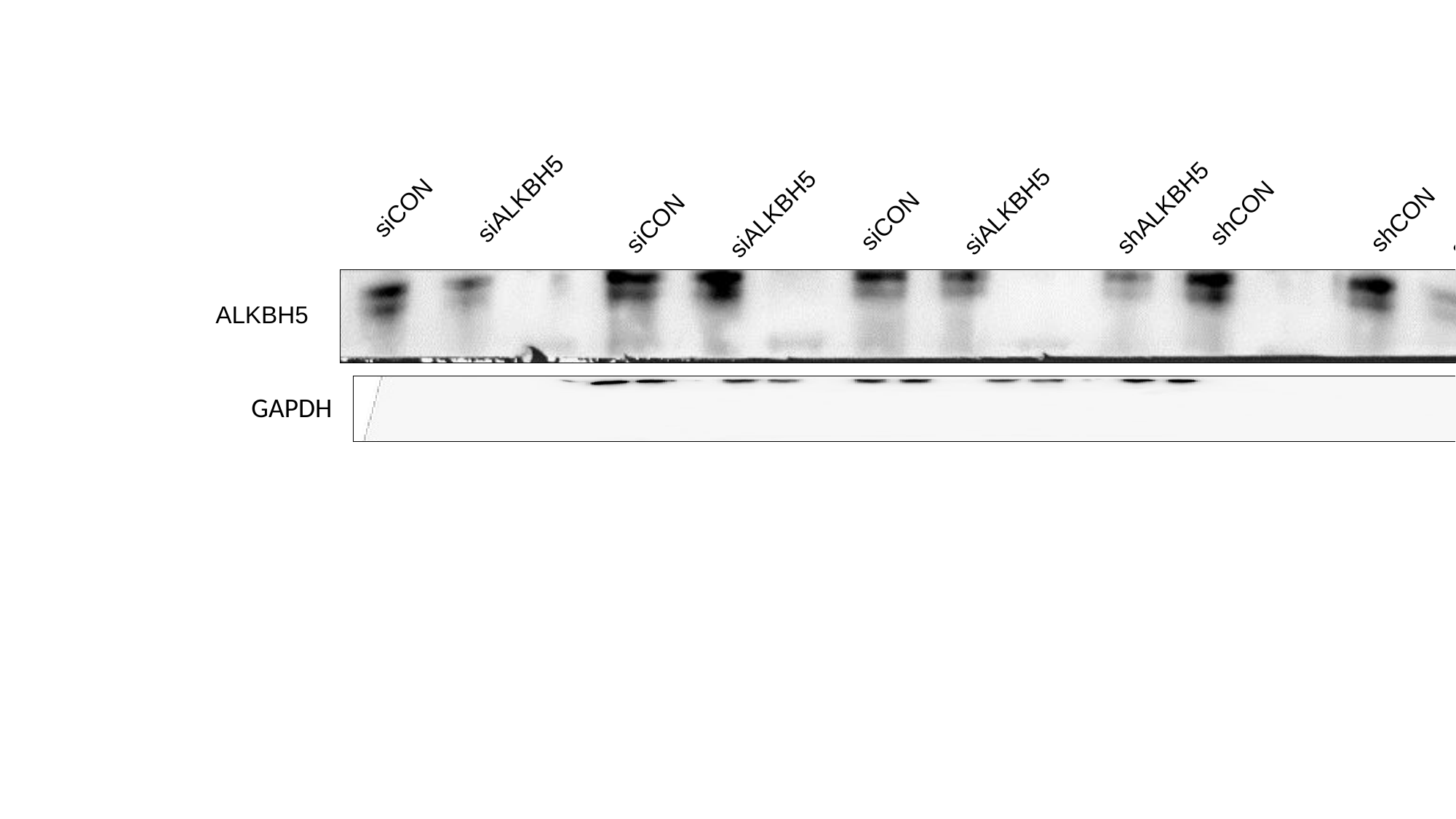

siALKBH5
siCON
shCON
shALKBH5
siALKBH5
shALKBH5
siCON
siALKBH5
shCON
siCON
ALKBH5
GAPDH

## Slide 2
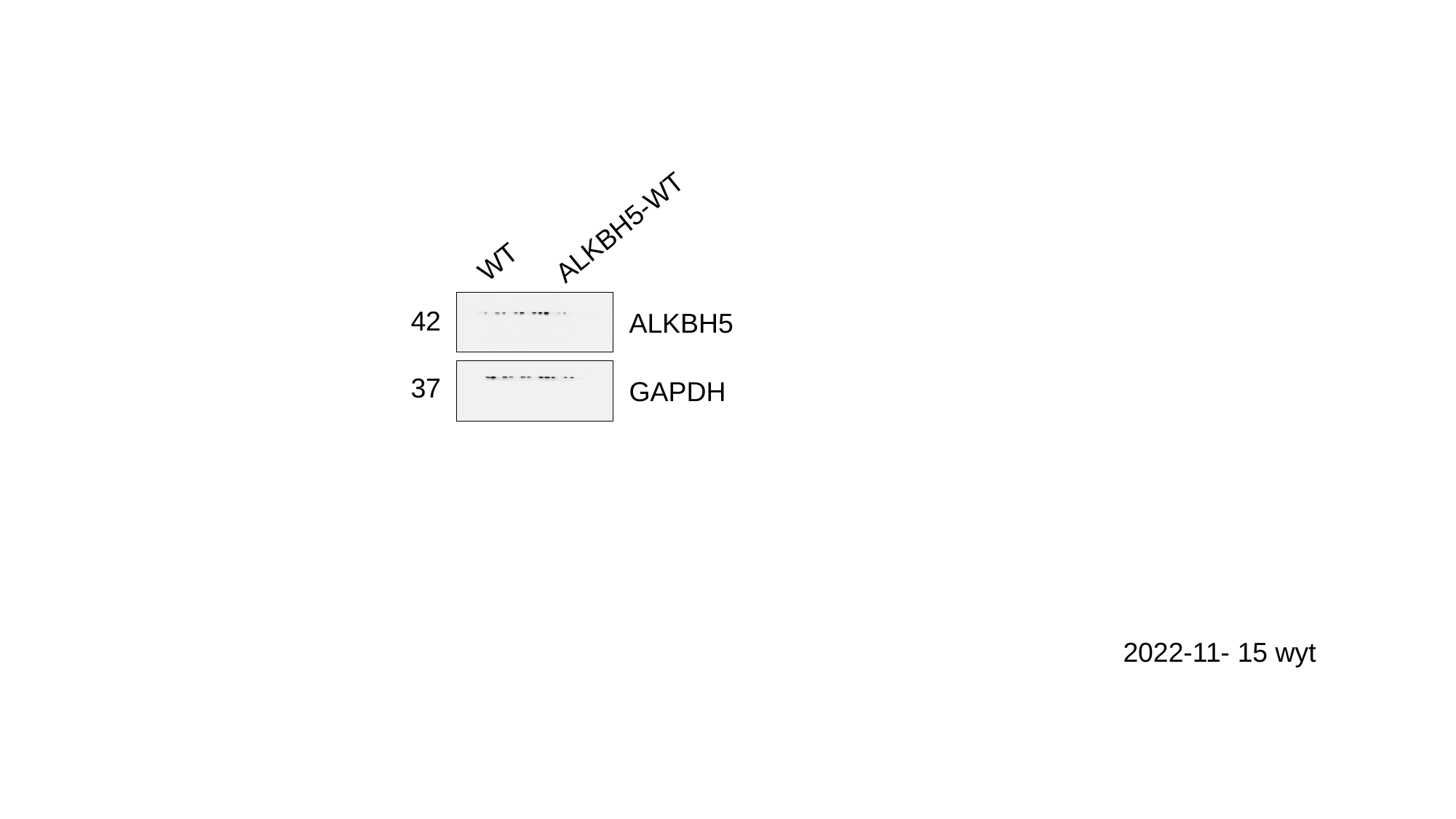

ALKBH5-WT
WT
42
ALKBH5
37
GAPDH
2022-11- 15 wyt

## Slide 3
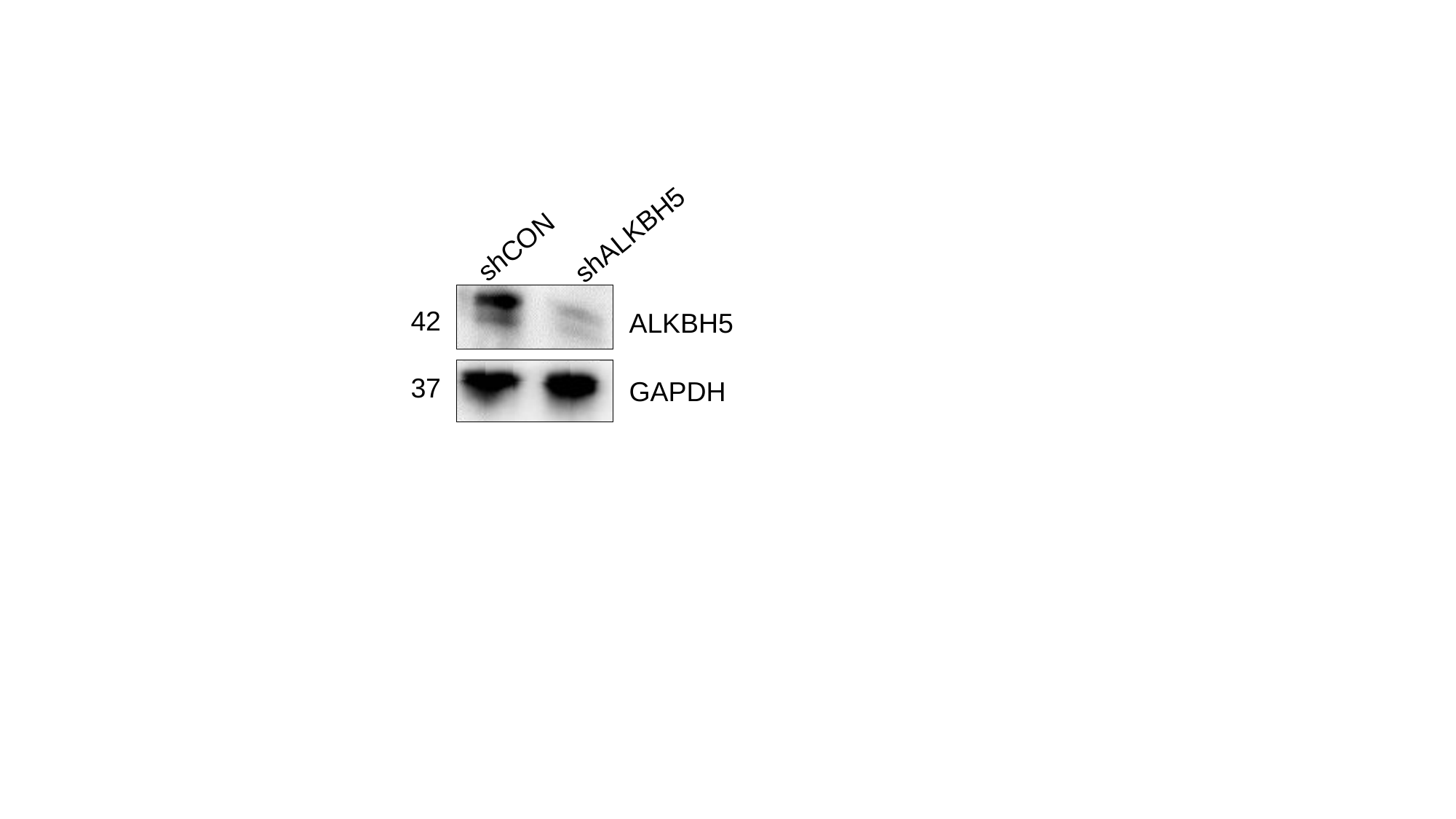

shALKBH5
shCON
42
ALKBH5
37
GAPDH
