# Supplementary material for: ALKBH5 promotes hypopharyngeal squamous cell carcinoma apoptosis by targeting TLR2 in a YTHDF1/IGF2BP2-mediated manner
Source: Cell Death Discov. 2023 Aug 23;9:308. doi: 10.1038/s41420-023-01589-6 (PMC10447508; doi:10.1038/s41420-023-01589-6)
Supplement: Supplementary file 7 — original data [file 41420_2023_1589_MOESM7_ESM.zip › 5M-WB 完成/New Microsoft PowerPoint Presentation.pptx]

## Slide 1
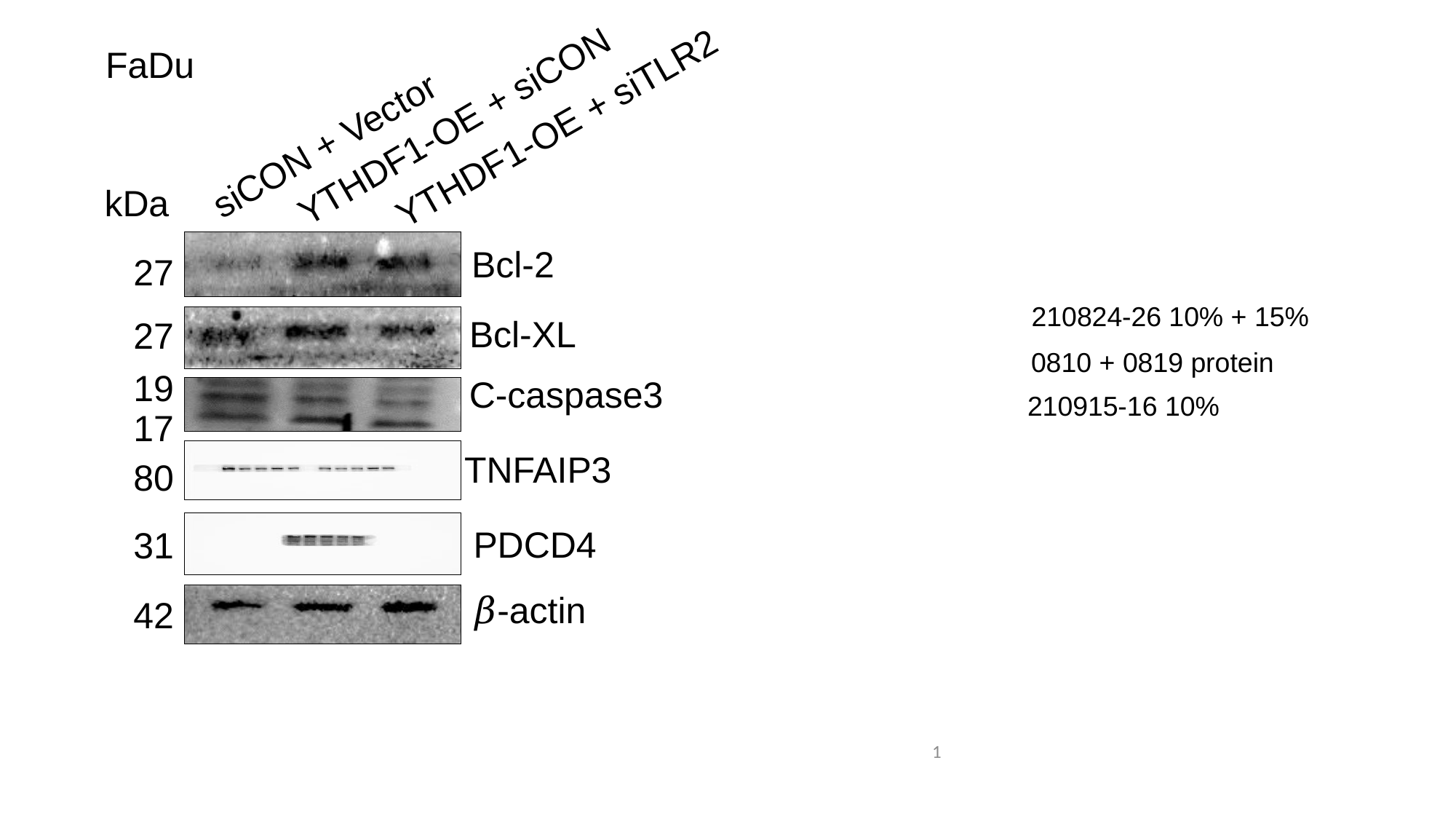

YTHDF1-OE + siCON
YTHDF1-OE + siTLR2
siCON + Vector
kDa
Bcl-2
27
Bcl-XL
27
19
C-caspase3
17
TNFAIP3
80
PDCD4
31
𝛽-actin
42
FaDu
210824-26 10% + 15%
0810 + 0819 protein
210915-16 10%
1

## Slide 2
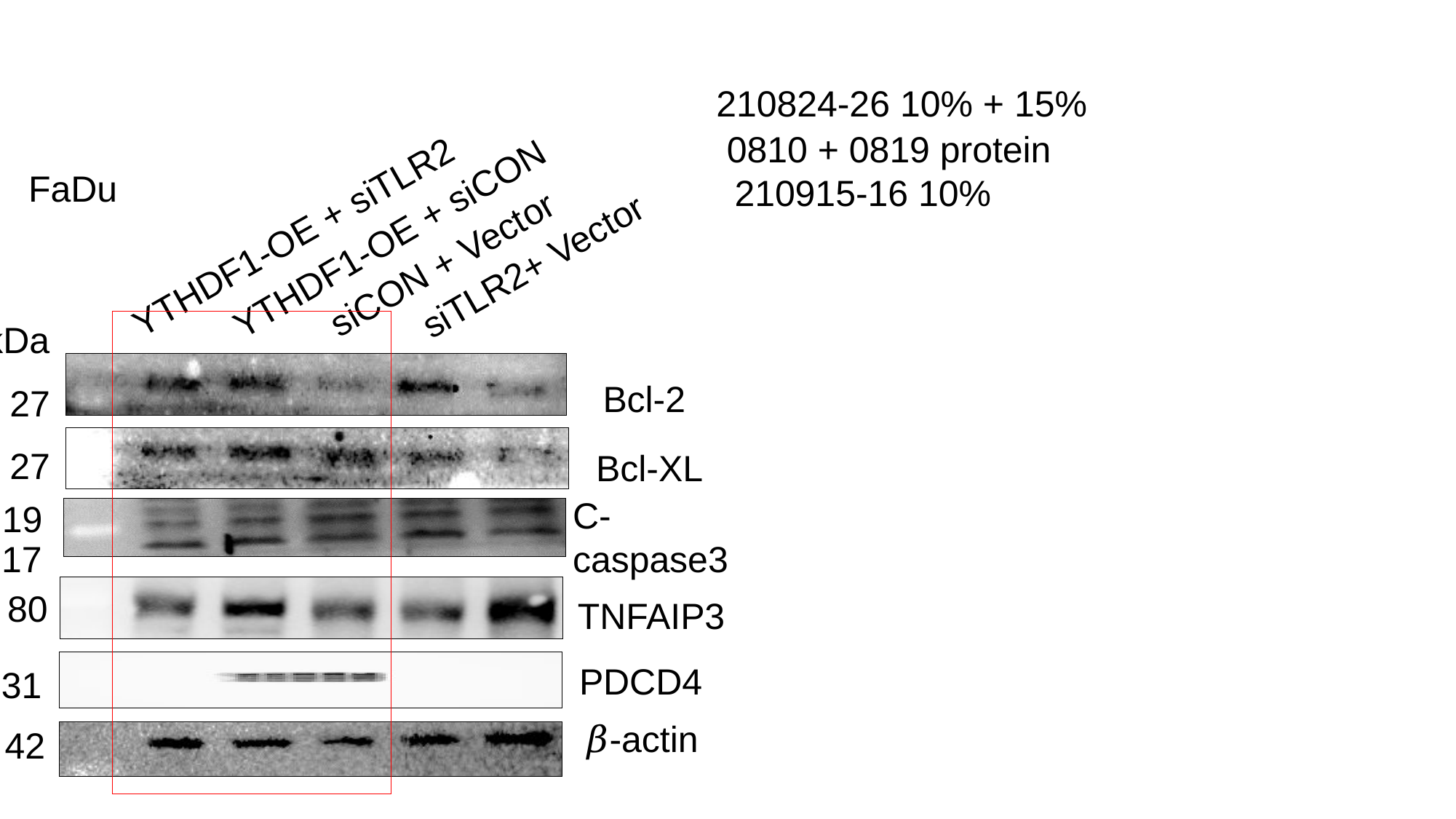

210824-26 10% + 15%
0810 + 0819 protein
210915-16 10%
YTHDF1-OE + siCON
YTHDF1-OE + siTLR2
siCON + Vector
kDa
Bcl-2
27
27
Bcl-XL
C-caspase3
19
17
80
TNFAIP3
PDCD4
31
𝛽-actin
42
FaDu
siTLR2+ Vector
2
