# Supplementary material for: ALKBH5 promotes hypopharyngeal squamous cell carcinoma apoptosis by targeting TLR2 in a YTHDF1/IGF2BP2-mediated manner
Source: Cell Death Discov. 2023 Aug 23;9:308. doi: 10.1038/s41420-023-01589-6 (PMC10447508; doi:10.1038/s41420-023-01589-6)
Supplement: Supplementary file 7 — original data [file 41420_2023_1589_MOESM7_ESM.zip › 5D-WB 完成/New Microsoft PowerPoint Presentation.pptx]

## Slide 1
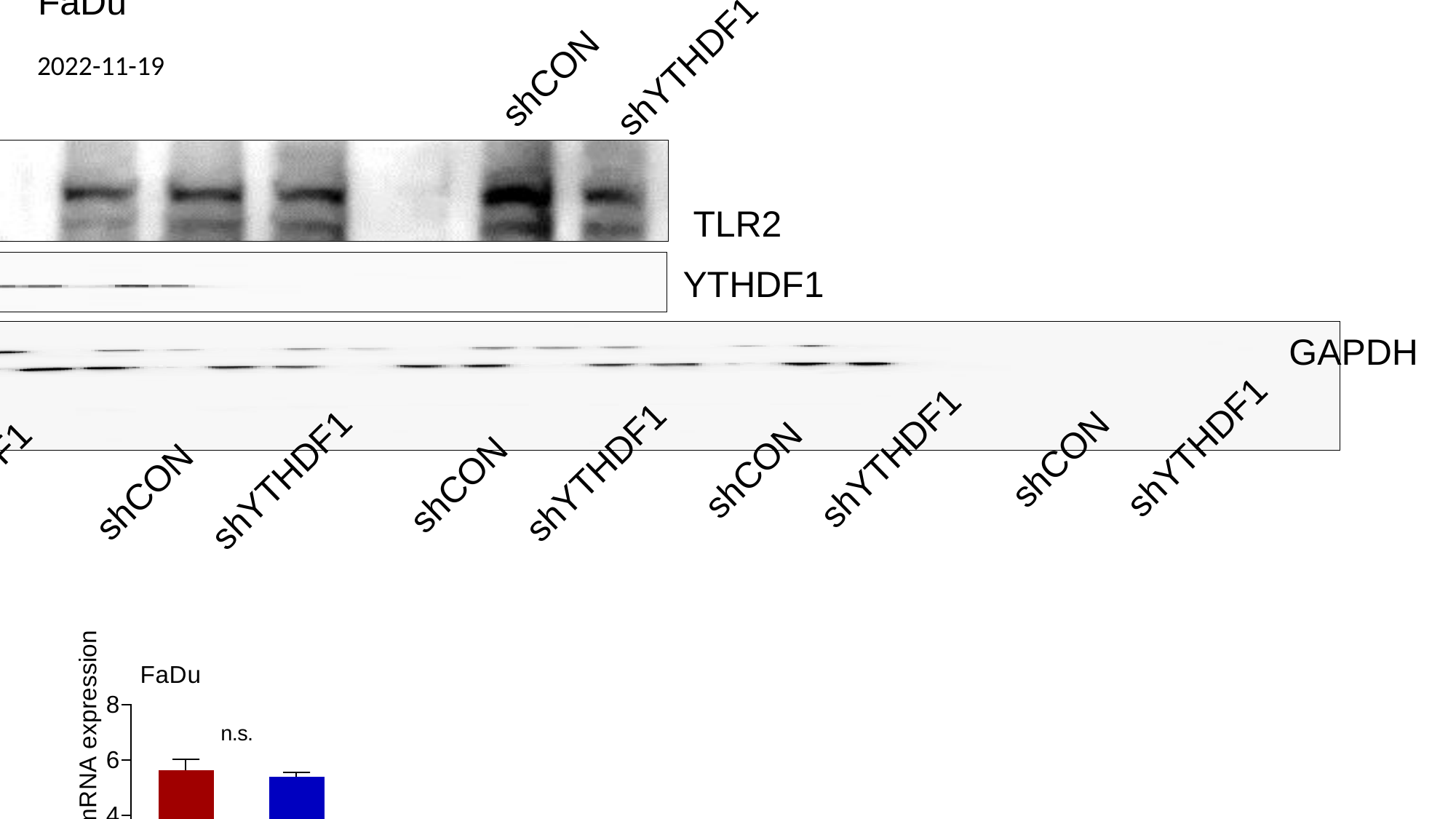

FaDu
shYTHDF1
shCON
2022-11-19
100
TLR2
42
YTHDF1
GAPDH
GAPDH
37
shYTHDF1
shCON
shYTHDF1
shCON
shYTHDF1
shYTHDF1
shCON
shCON
shYTHDF1
shCON

## Slide 2
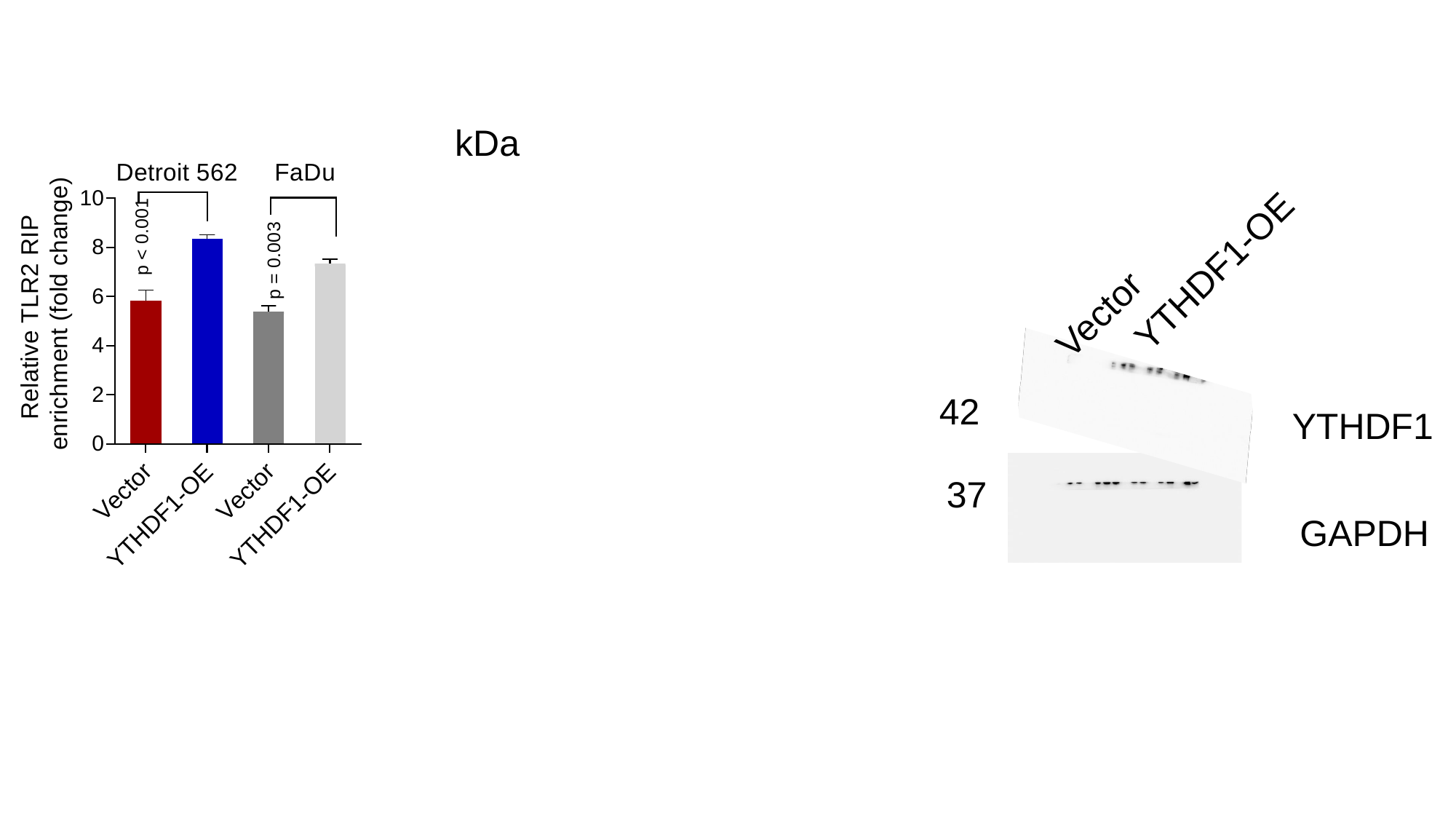

kDa
YTHDF1-OE
Vector
42
YTHDF1
GAPDH
37
