# Supplementary material for: ALKBH5 promotes hypopharyngeal squamous cell carcinoma apoptosis by targeting TLR2 in a YTHDF1/IGF2BP2-mediated manner
Source: Cell Death Discov. 2023 Aug 23;9:308. doi: 10.1038/s41420-023-01589-6 (PMC10447508; doi:10.1038/s41420-023-01589-6)
Supplement: Supplementary file 7 — original data [file 41420_2023_1589_MOESM7_ESM.zip › 4E-WB 完成/New Microsoft PowerPoint Presentation.pptx]

## Slide 1
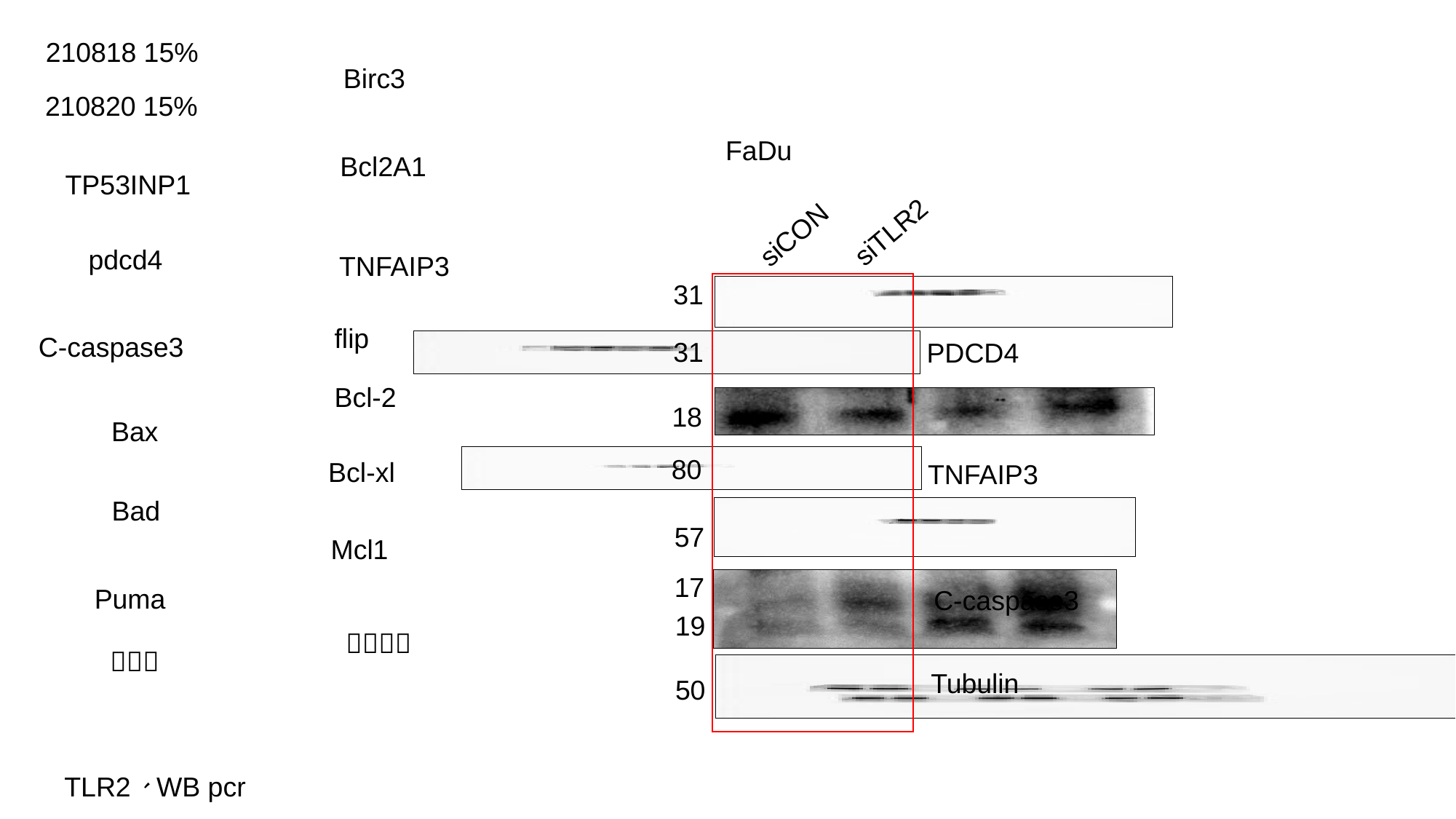

210818 15%
Birc3
210820 15%
FaDu
siTLR2
siCON
31
TP53INP1
31
PDCD4
Bcl2A1
18
80
TNFAIP3
Flip
57
Tubulin
50
17
C-caspase3
19
Bcl2A1
TP53INP1
pdcd4
TNFAIP3
flip
C-caspase3
Bcl-2
Bax
Bcl-xl
Bad
Mcl1
Puma
抑制凋亡
促凋亡
TLR2下游基因、WB pcr
