# Supplementary material for: ALKBH5 promotes hypopharyngeal squamous cell carcinoma apoptosis by targeting TLR2 in a YTHDF1/IGF2BP2-mediated manner
Source: Cell Death Discov. 2023 Aug 23;9:308. doi: 10.1038/s41420-023-01589-6 (PMC10447508; doi:10.1038/s41420-023-01589-6)

## Slide 1
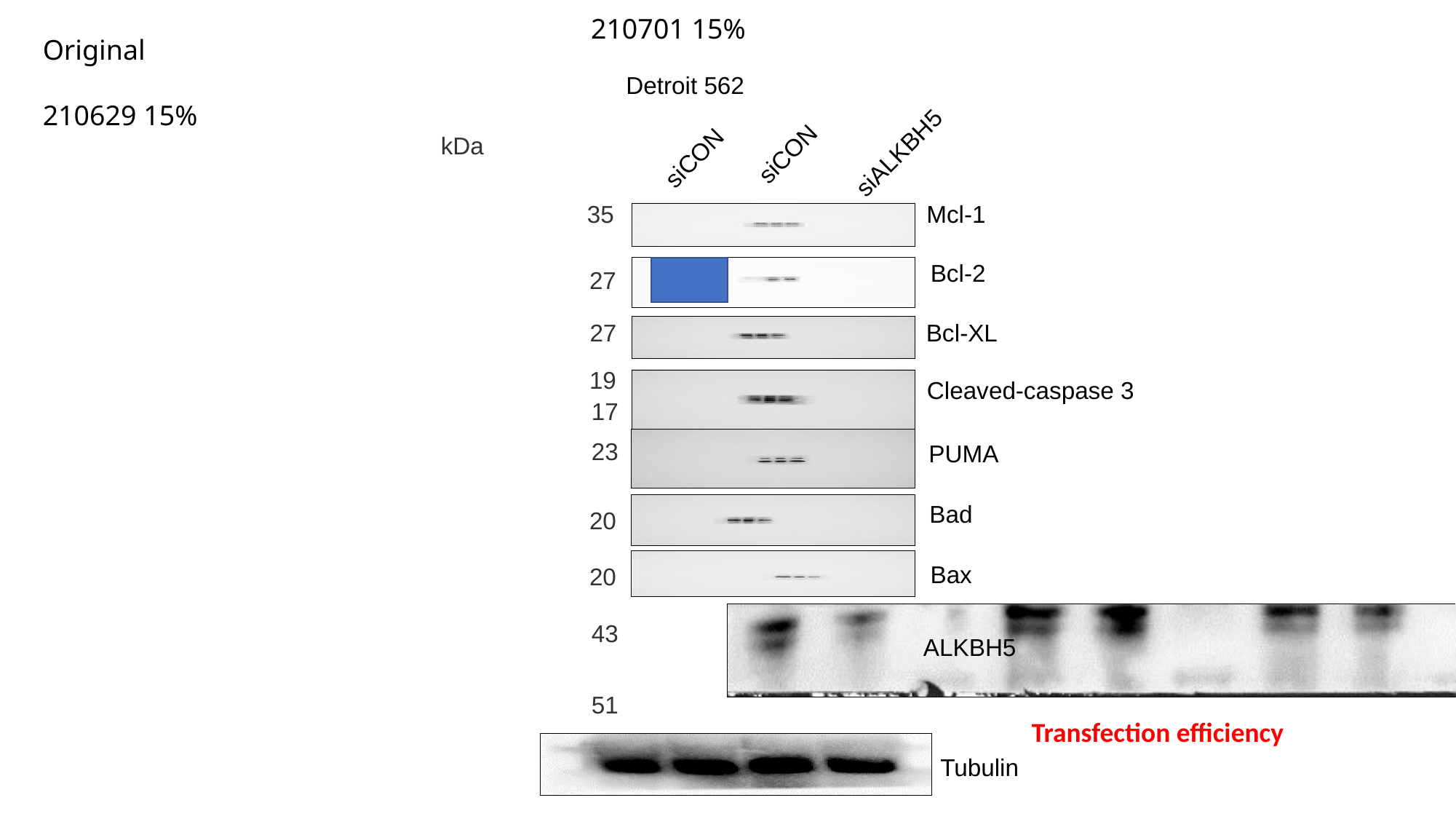

210701 15%
Original
210629 15%
Detroit 562
siCON
kDa
35
Mcl-1
Bcl-2
27
27
Bcl-XL
19
Cleaved-caspase 3
17
23
PUMA
Bad
20
Bax
20
43
ALKBH5
51
Tubulin
siCON
siALKBH5
Transfection efficiency

## Slide 2
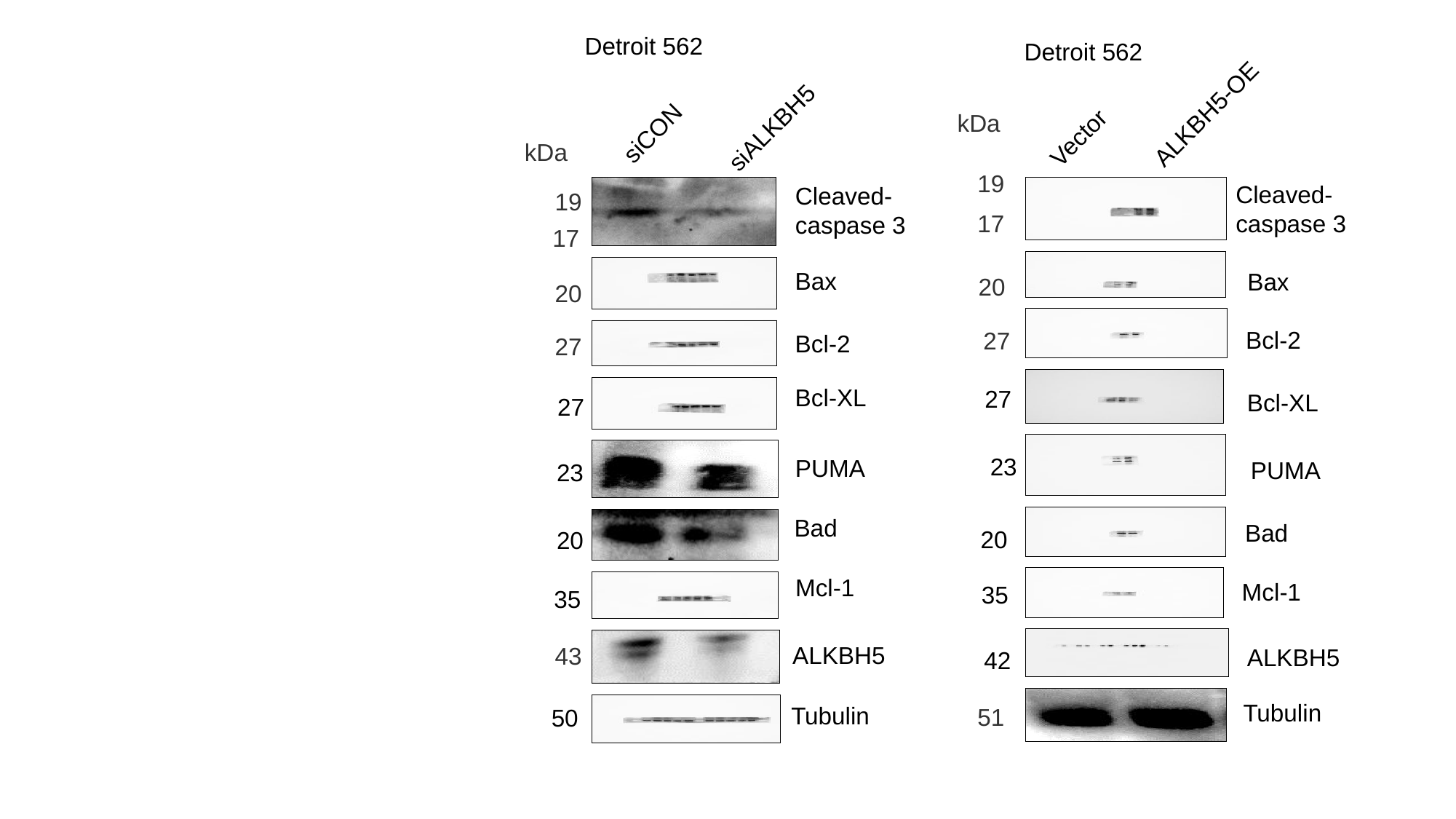

Detroit 562
Detroit 562
Vector
ALKBH5-OE
kDa
19
Cleaved-
caspase 3
17
Bax
20
Bcl-2
27
27
Bcl-XL
23
PUMA
Bad
20
Mcl-1
35
ALKBH5
42
Tubulin
51
siCON
siALKBH5
Cleaved-
caspase 3
19
17
20
27
27
23
20
35
50
Bax
Bcl-2
Bcl-XL
PUMA
Bad
Mcl-1
Tubulin
kDa
ALKBH5
43

## Slide 3
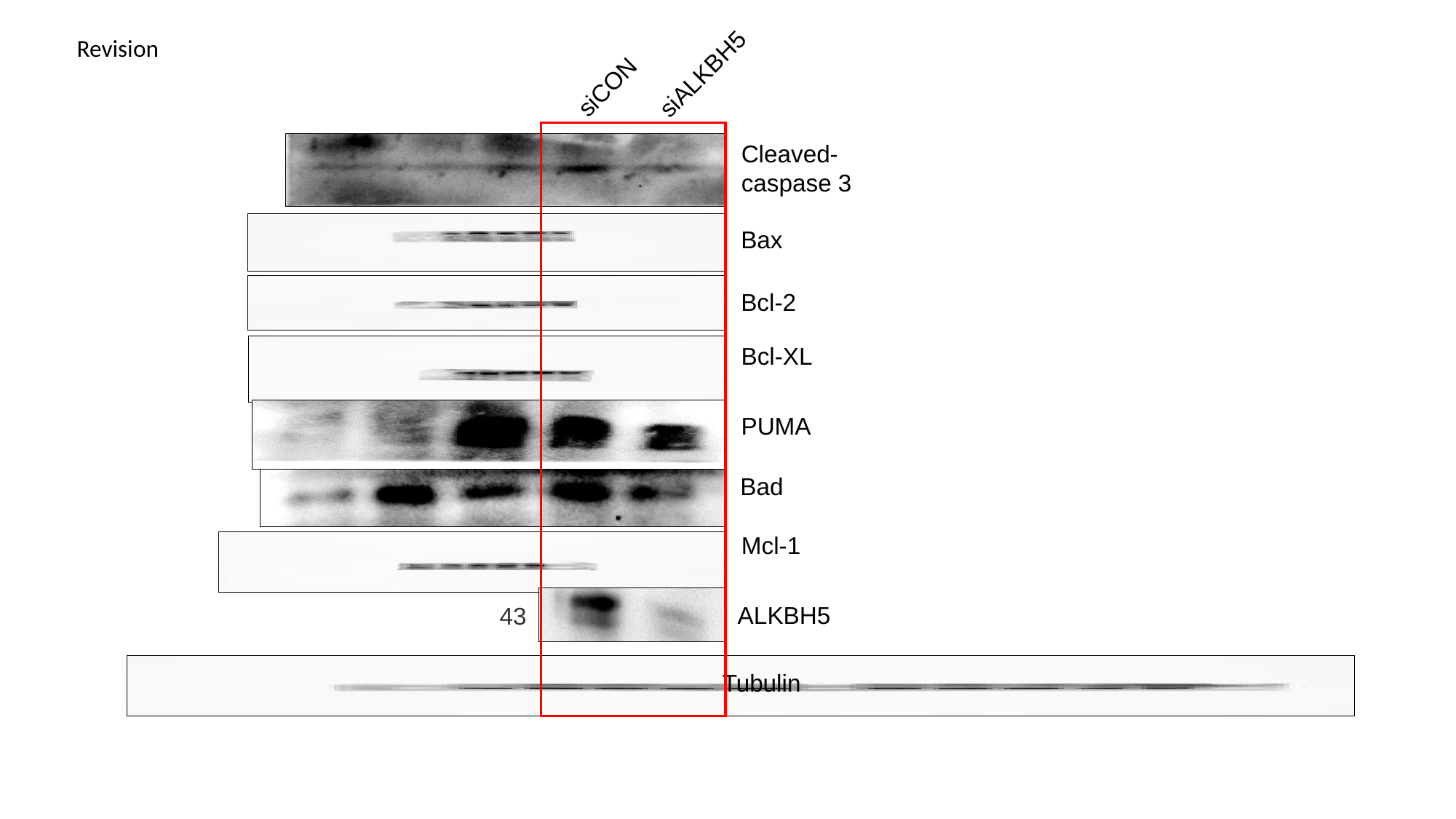

siALKBH5
siCON
Revision
Cleaved-
caspase 3
19
17
20
27
27
23
20
35
50
Bax
Bcl-2
Bcl-XL
PUMA
Bad
Mcl-1
Tubulin
ALKBH5
43
Tubulin

## Slide 4
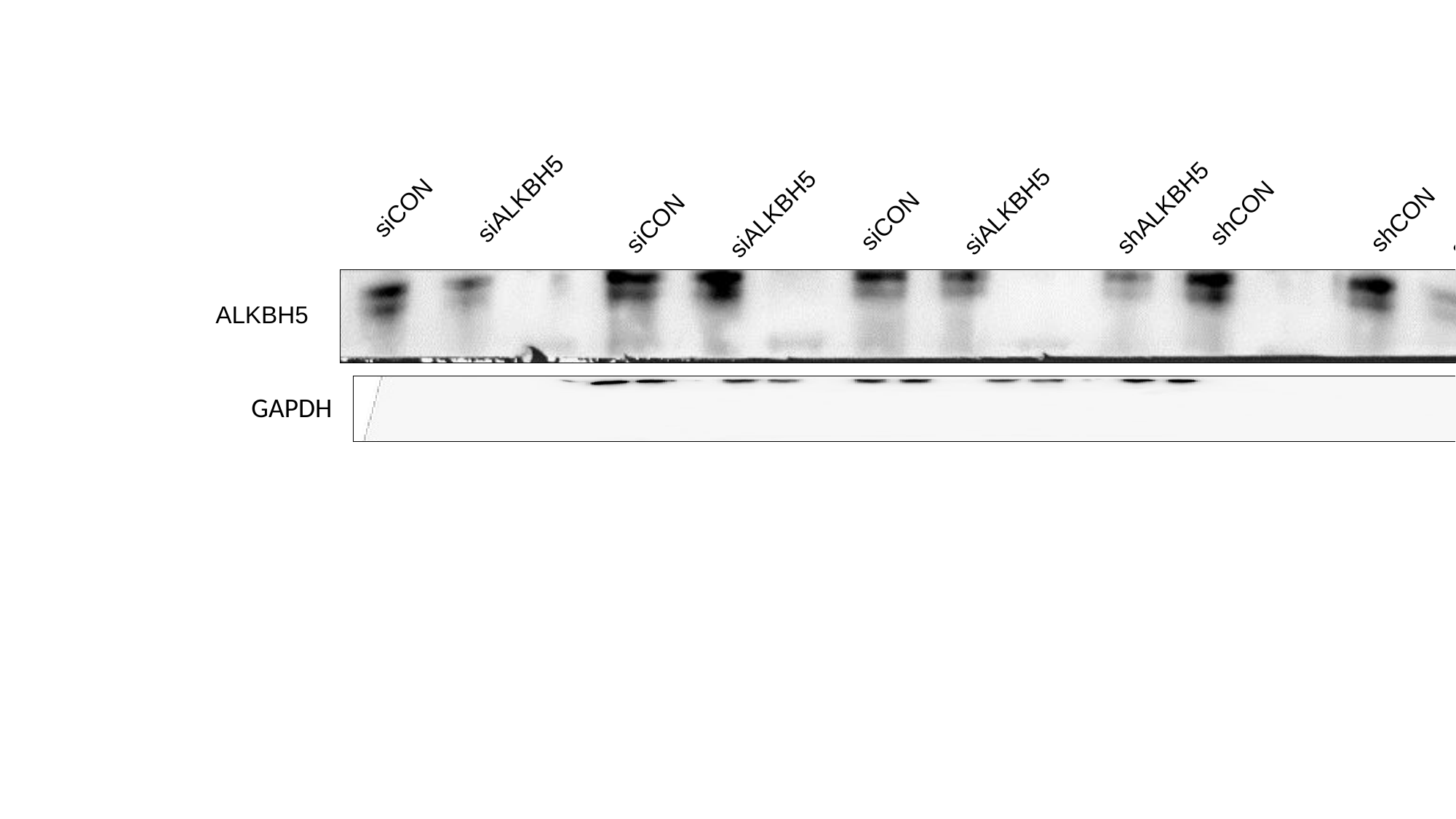

siALKBH5
siCON
shCON
shALKBH5
siALKBH5
shALKBH5
siCON
siALKBH5
shCON
siCON
ALKBH5
GAPDH

## Slide 5
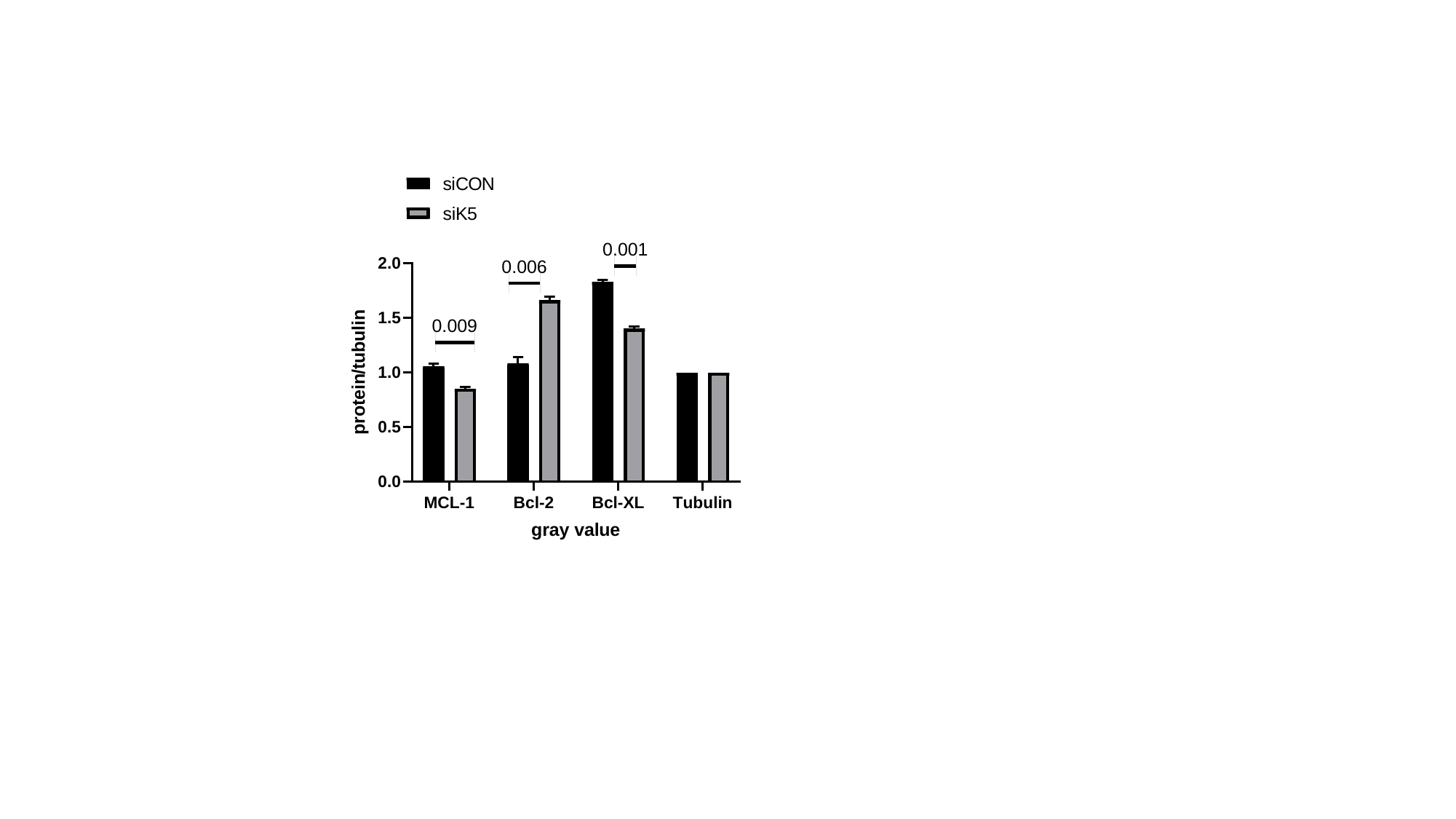

Supplement: Supplementary file 7 — original data [file 41420_2023_1589_MOESM7_ESM.zip › 2I-WB/Blots and analysis.pptx]
