# Supplementary material for: ALKBH5 promotes hypopharyngeal squamous cell carcinoma apoptosis by targeting TLR2 in a YTHDF1/IGF2BP2-mediated manner
Source: Cell Death Discov. 2023 Aug 23;9:308. doi: 10.1038/s41420-023-01589-6 (PMC10447508; doi:10.1038/s41420-023-01589-6)
Supplement: Supplementary file 7 — original data [file 41420_2023_1589_MOESM7_ESM.zip › 3G-WB/New Microsoft PowerPoint Presentation.pptx]

## Slide 1
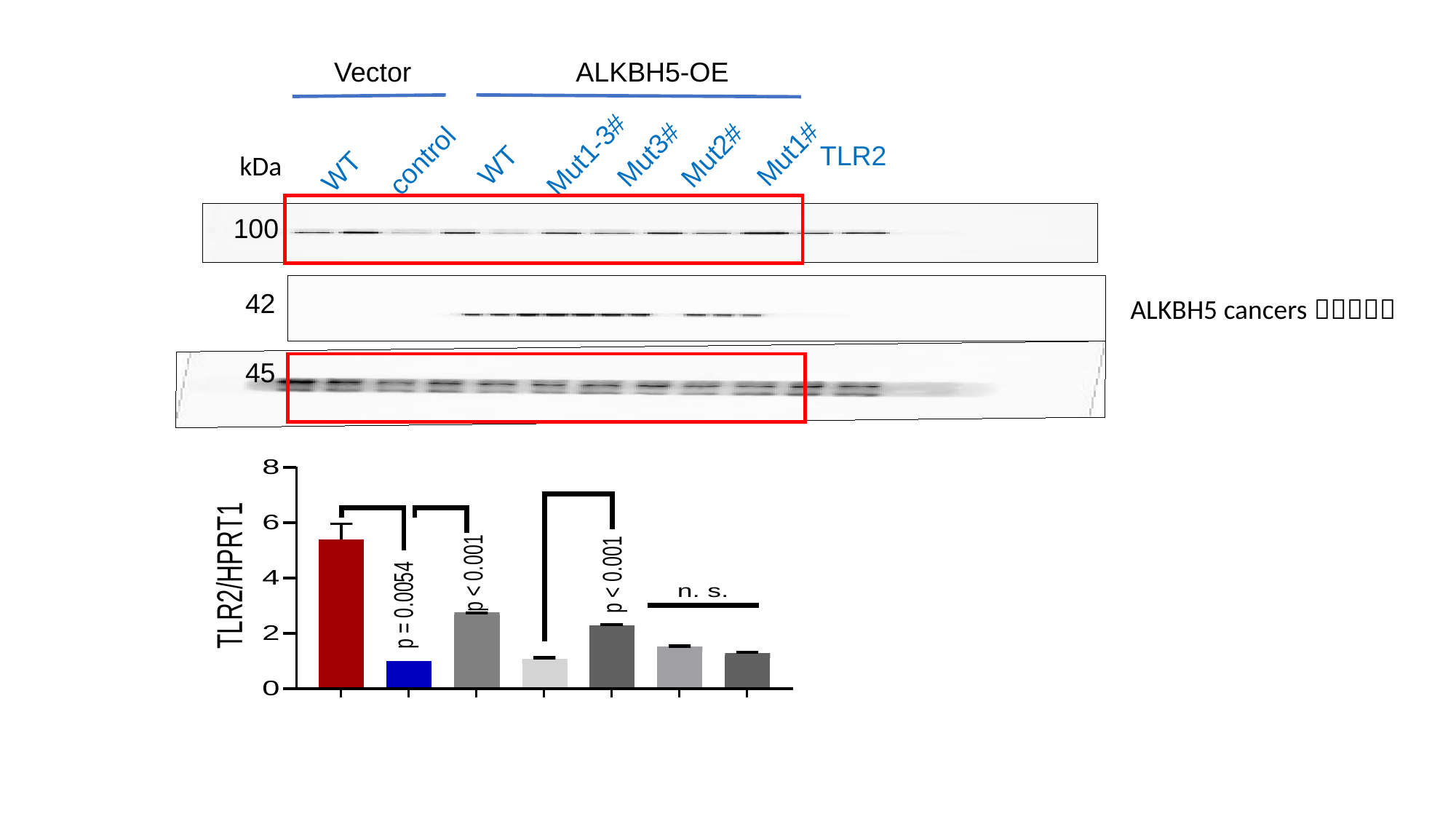

ALKBH5-OE
Vector
WT
Mut1#
Mut3#
Mut2#
WT
control
Mut1-3#
TLR2
kDa
100
TLR2
ALKBH5
42
ALKBH5 cancers补实验做的
45
β-actin

## Slide 2
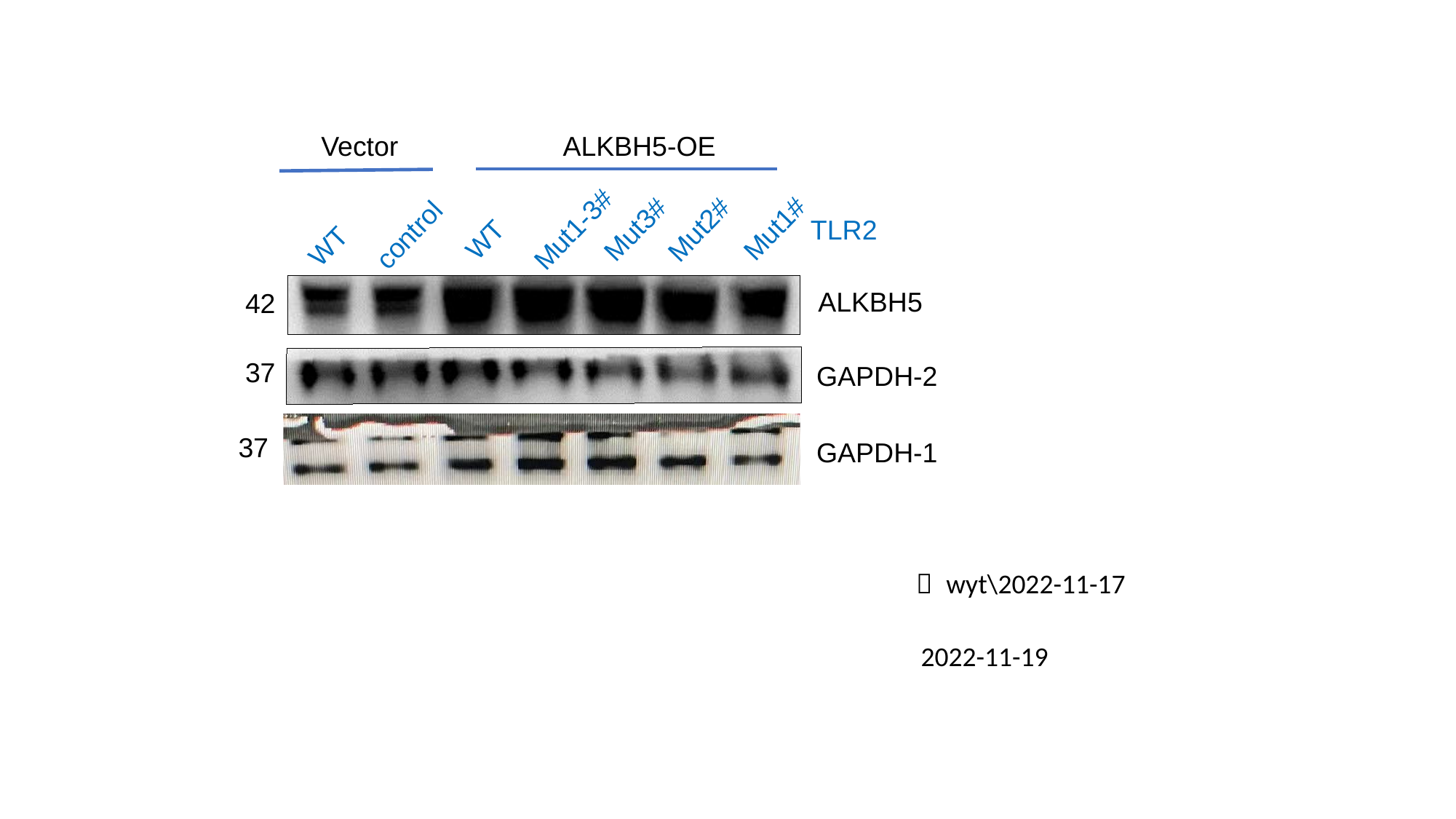

ALKBH5-OE
Vector
WT
Mut1#
Mut3#
Mut2#
WT
control
Mut1-3#
TLR2
ALKBH5
42
37
GAPDH-2
37
GAPDH-1
补 wyt\2022-11-17
2022-11-19
