# Supplementary material for: ALKBH5 promotes hypopharyngeal squamous cell carcinoma apoptosis by targeting TLR2 in a YTHDF1/IGF2BP2-mediated manner
Source: Cell Death Discov. 2023 Aug 23;9:308. doi: 10.1038/s41420-023-01589-6 (PMC10447508; doi:10.1038/s41420-023-01589-6)
Supplement: Supplementary file 7 — original data [file 41420_2023_1589_MOESM7_ESM.zip › 2J-WB/New Microsoft PowerPoint Presentation.pptx]

## Slide 1
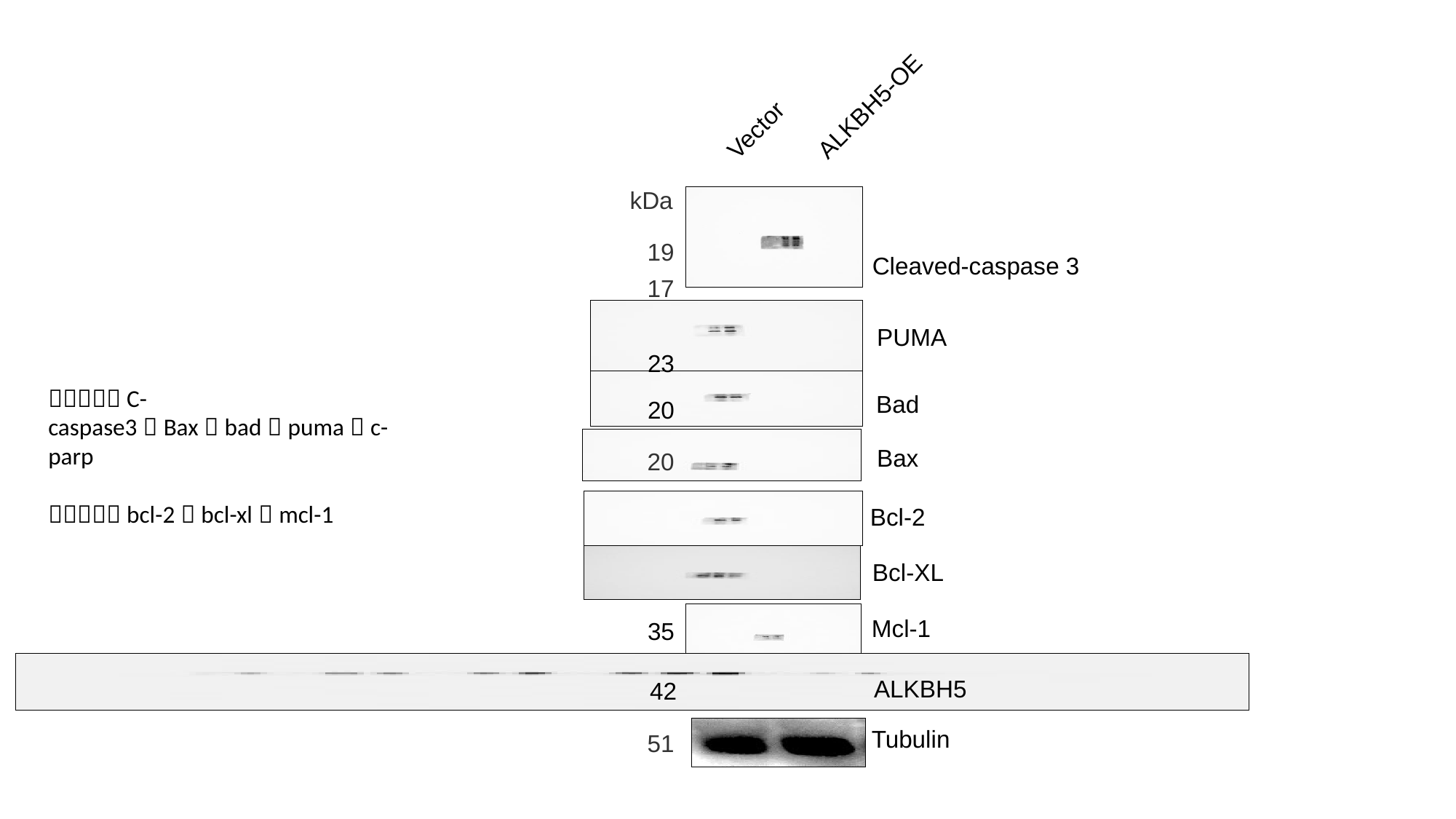

Vector
ALKBH5-OE
kDa
19
Cleaved-caspase 3
17
PUMA
23
促进凋亡：C-caspase3，Bax，bad，puma，c-parp
抑制凋亡：bcl-2，bcl-xl，mcl-1
Bad
20
Bax
20
Bcl-2
27
27
Bcl-XL
Mcl-1
35
ALKBH5
42
Tubulin
51
