# Supplementary figures and images for: ALKBH5 promotes hypopharyngeal squamous cell carcinoma apoptosis by targeting TLR2 in a YTHDF1/IGF2BP2-mediated manner
Source: Cell Death Discov. 2023 Aug 23;9:308. doi: 10.1038/s41420-023-01589-6 (PMC10447508; doi:10.1038/s41420-023-01589-6)

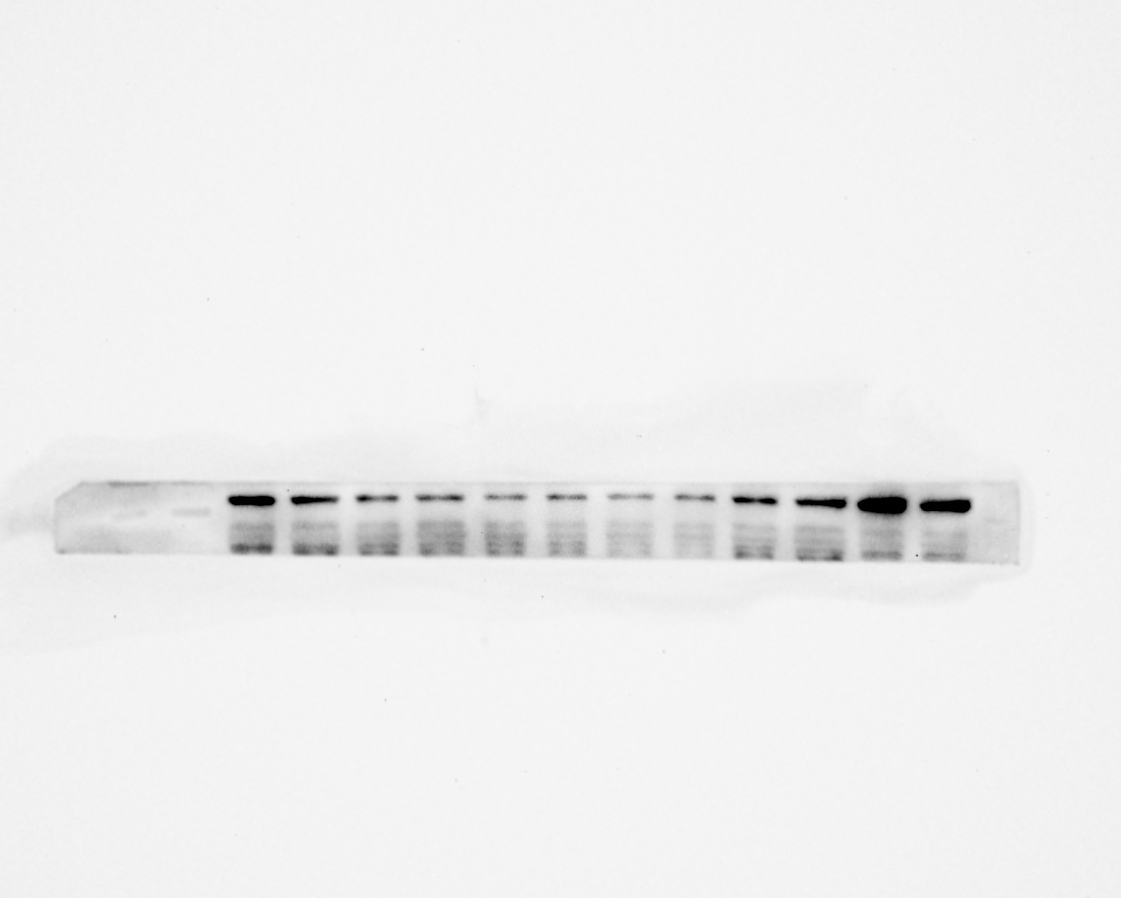

Supplement: Supplementary file 7 — original data [file 41420_2023_1589_MOESM7_ESM.zip › 1B-WB/ALKBH5.tif]

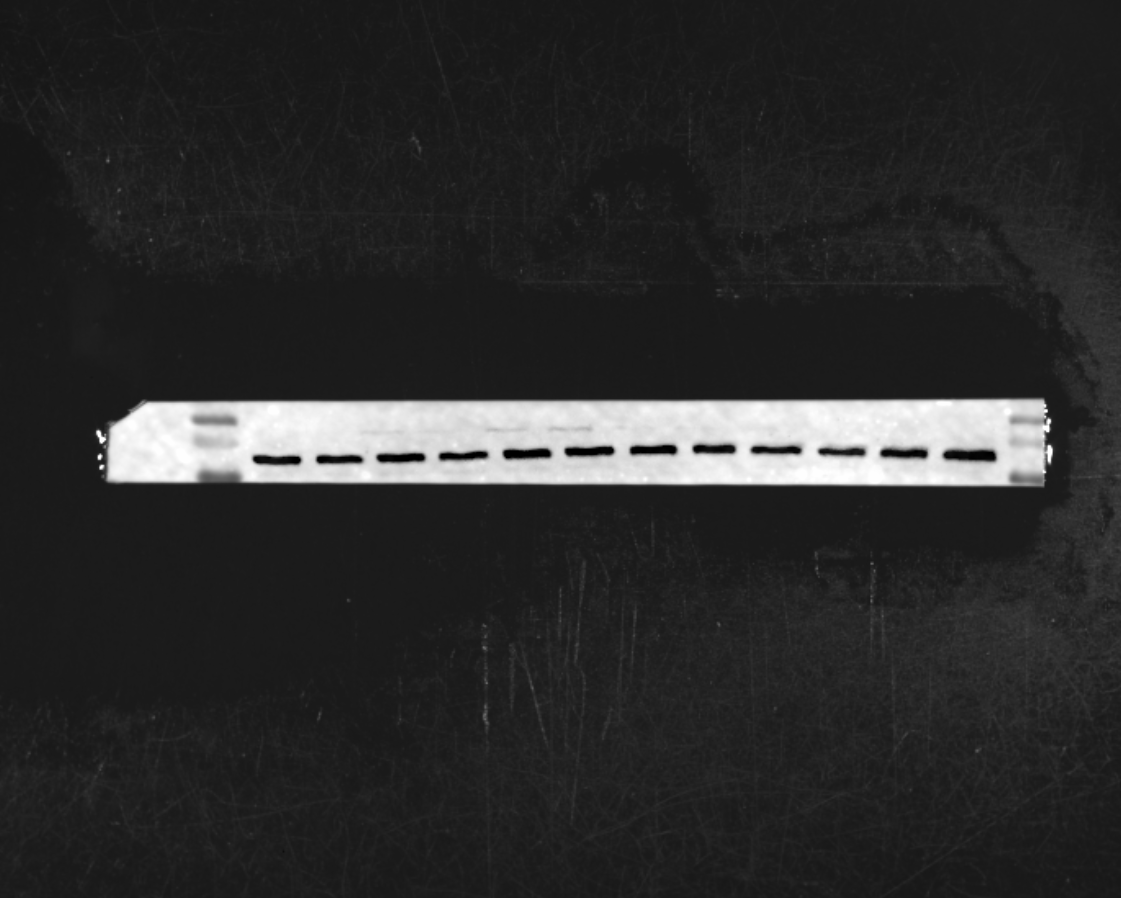

Supplement: Supplementary file 7 — original data [file 41420_2023_1589_MOESM7_ESM.zip › 1B-WB/bactin 211215 cxz 15%.tif]

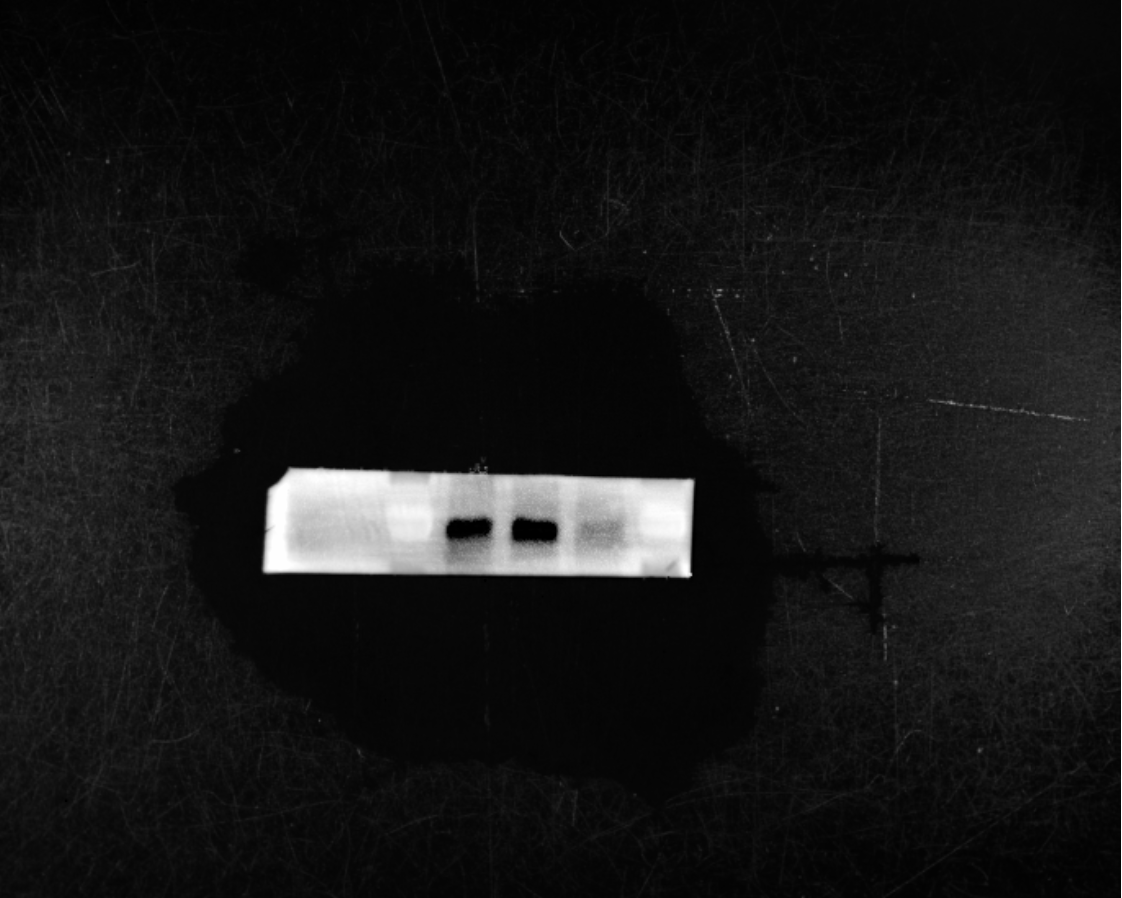

Supplement: Supplementary file 7 — original data [file 41420_2023_1589_MOESM7_ESM.zip › S5C-WB/page18-2 ythdf1 210602 cxz 10% y1_2已用.tif]

## Slide 1
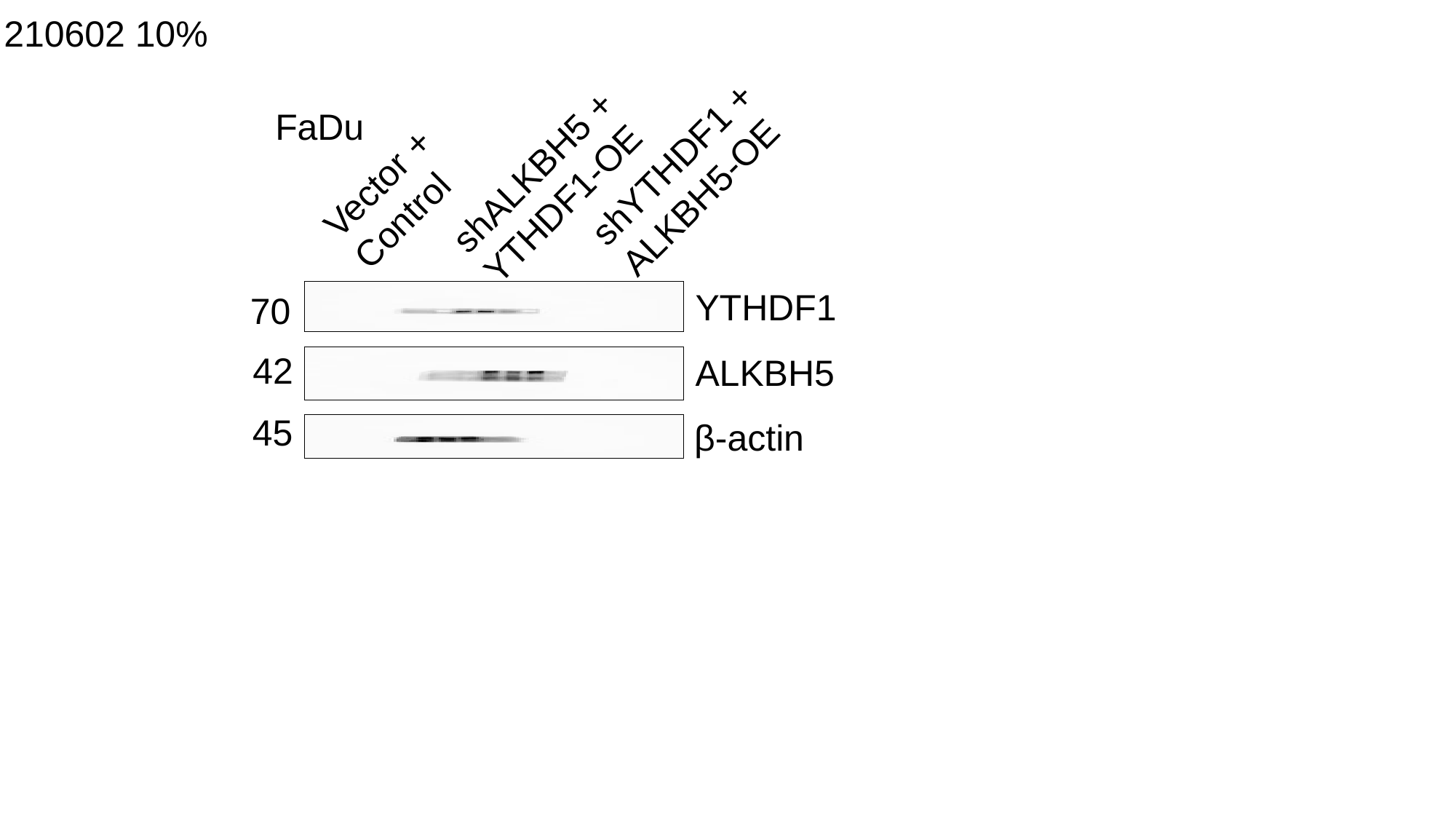

210602 10%
shYTHDF1 +
ALKBH5-OE
FaDu
Vector +
Control
shALKBH5 + YTHDF1-OE
YTHDF1
70
42
ALKBH5
45
β-actin

Supplement: Supplementary file 7 — original data [file 41420_2023_1589_MOESM7_ESM.zip › S5C-WB/New Microsoft PowerPoint Presentation.pptx]

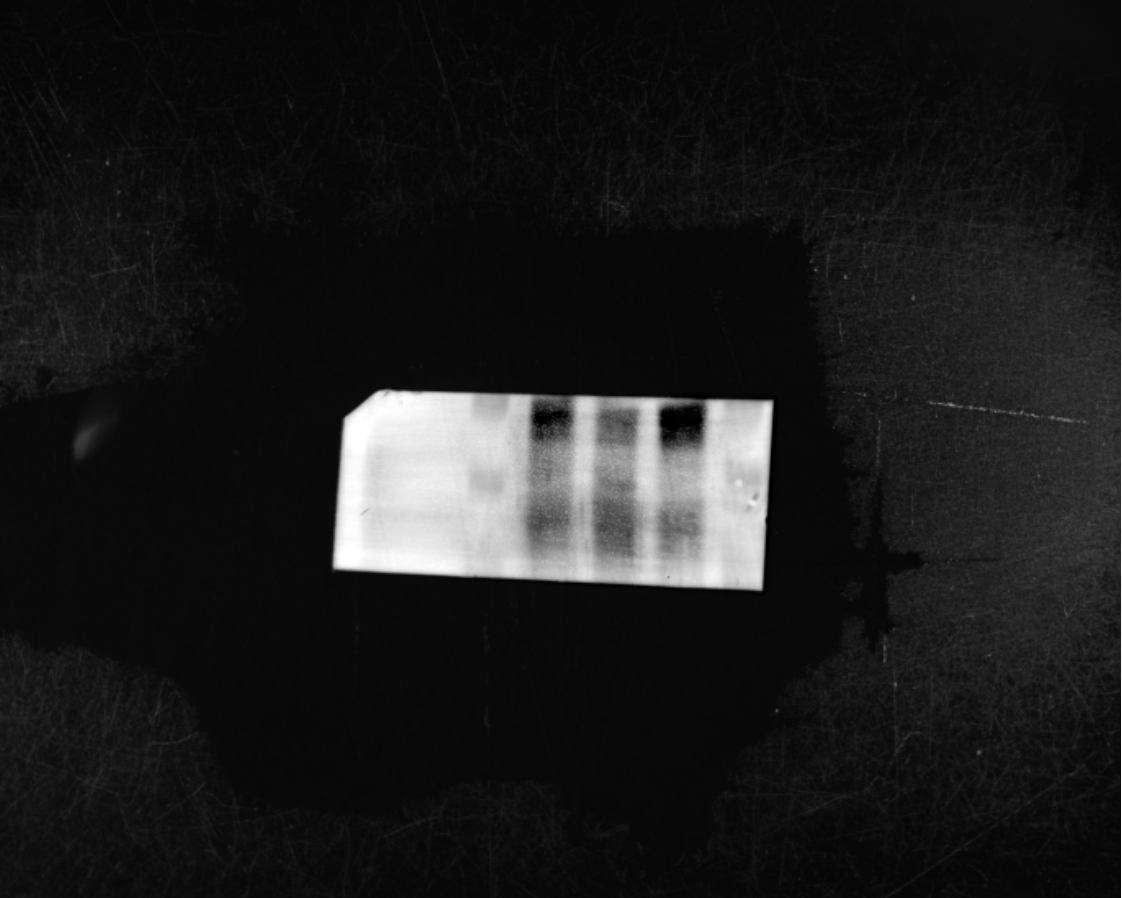

Supplement: Supplementary file 7 — original data [file 41420_2023_1589_MOESM7_ESM.zip › S5C-WB/page18-3 alkbh5 210602 cxz 10% k5_2已用.tif]

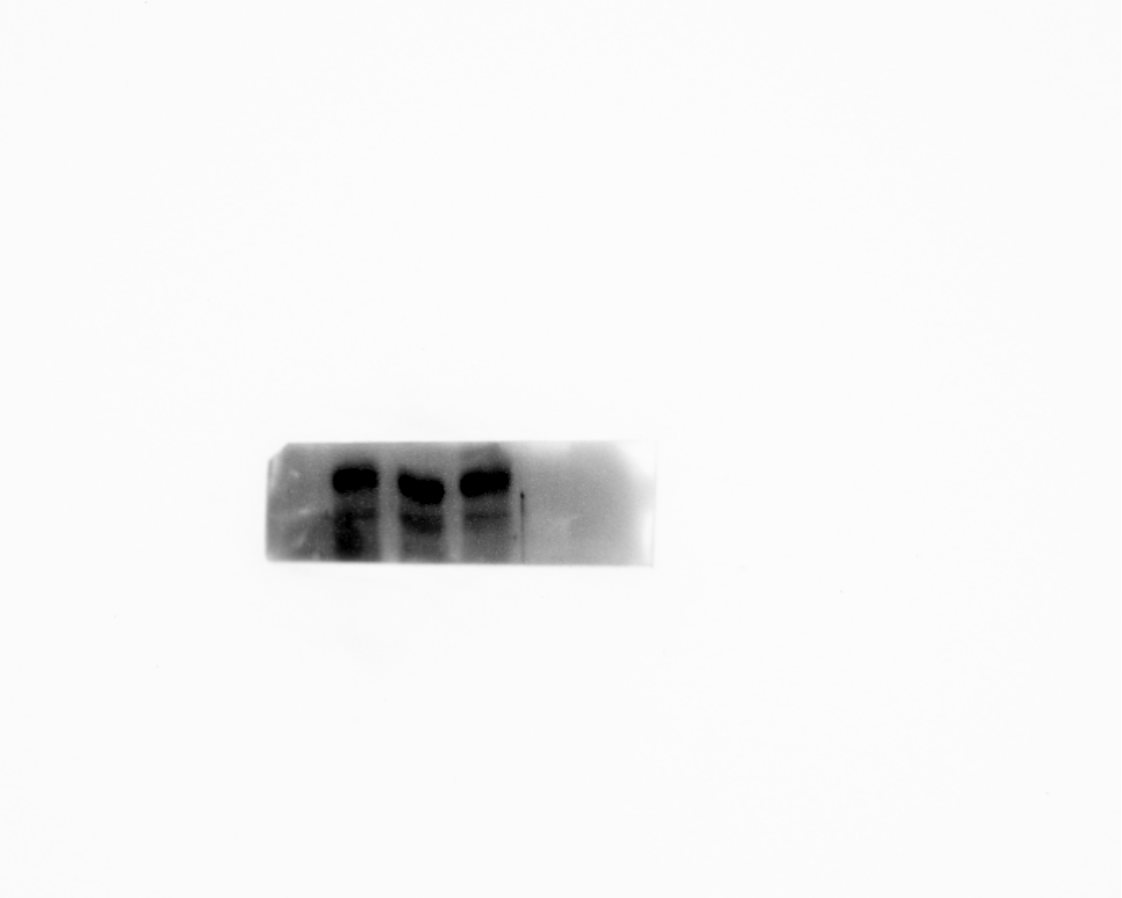

Supplement: Supplementary file 7 — original data [file 41420_2023_1589_MOESM7_ESM.zip › S5C-WB/page18-4 bactin 没有merge 210602 cxz 10% bactin已用.tif]

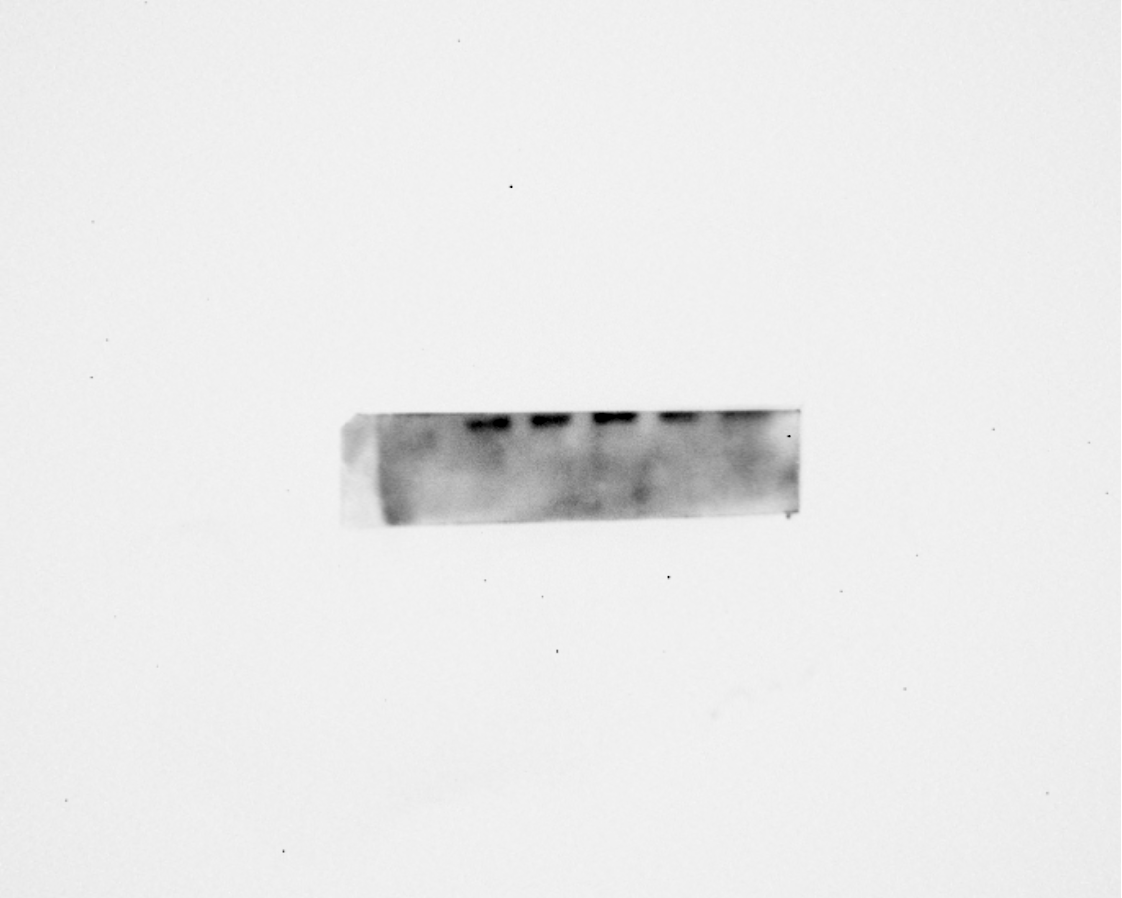

Supplement: Supplementary file 7 — original data [file 41420_2023_1589_MOESM7_ESM.zip › S6E-WB/page14-2 gapdh 没有merge 210901 cxz 10% mcl1已用.tif]

## Slide 1
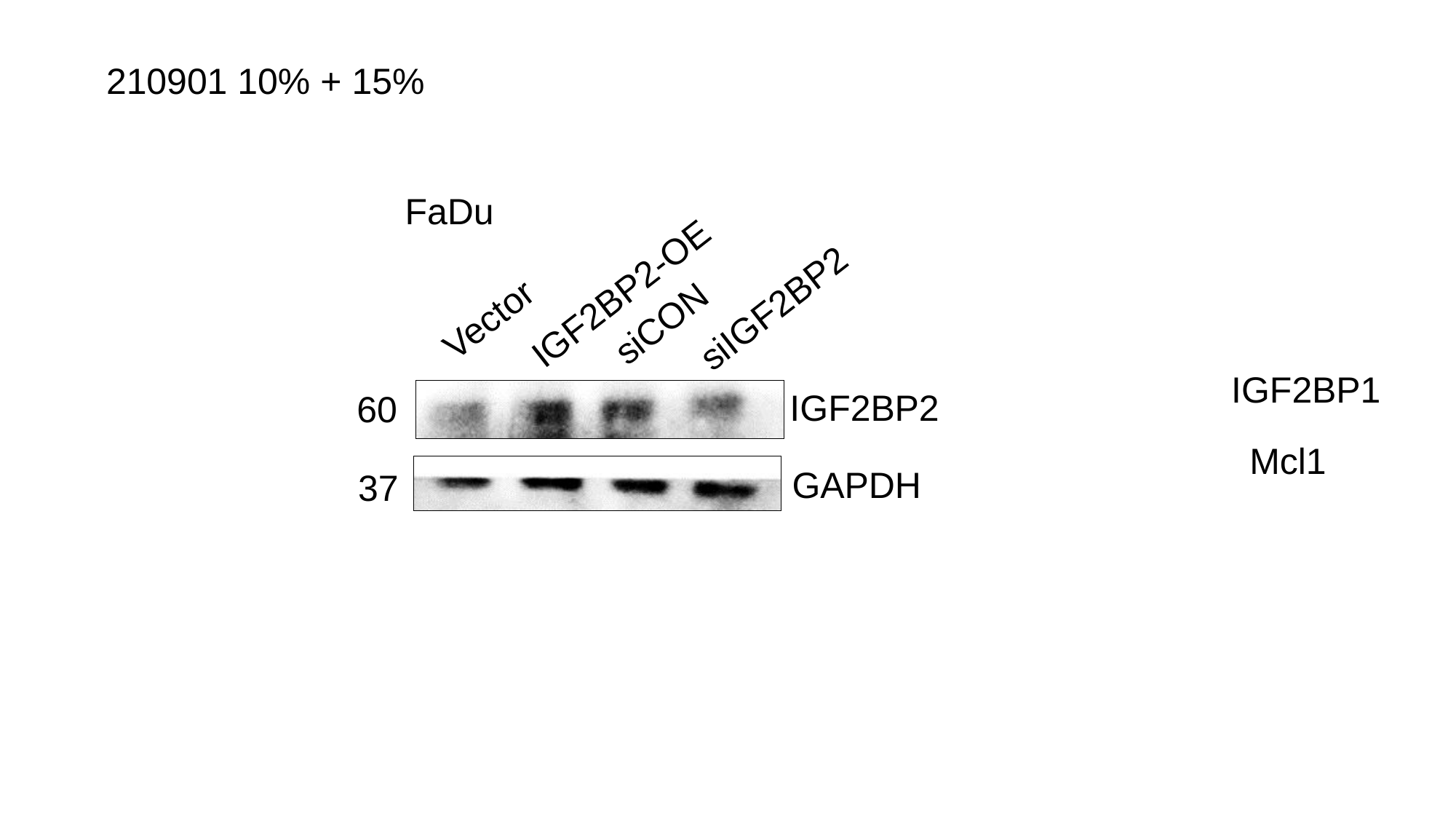

210901 10% + 15%
FaDu
IGF2BP2-OE
siIGF2BP2
Vector
siCON
IGF2BP2
60
GAPDH
37
IGF2BP1
Mcl1

Supplement: Supplementary file 7 — original data [file 41420_2023_1589_MOESM7_ESM.zip › S6E-WB/New Microsoft PowerPoint Presentation.pptx]

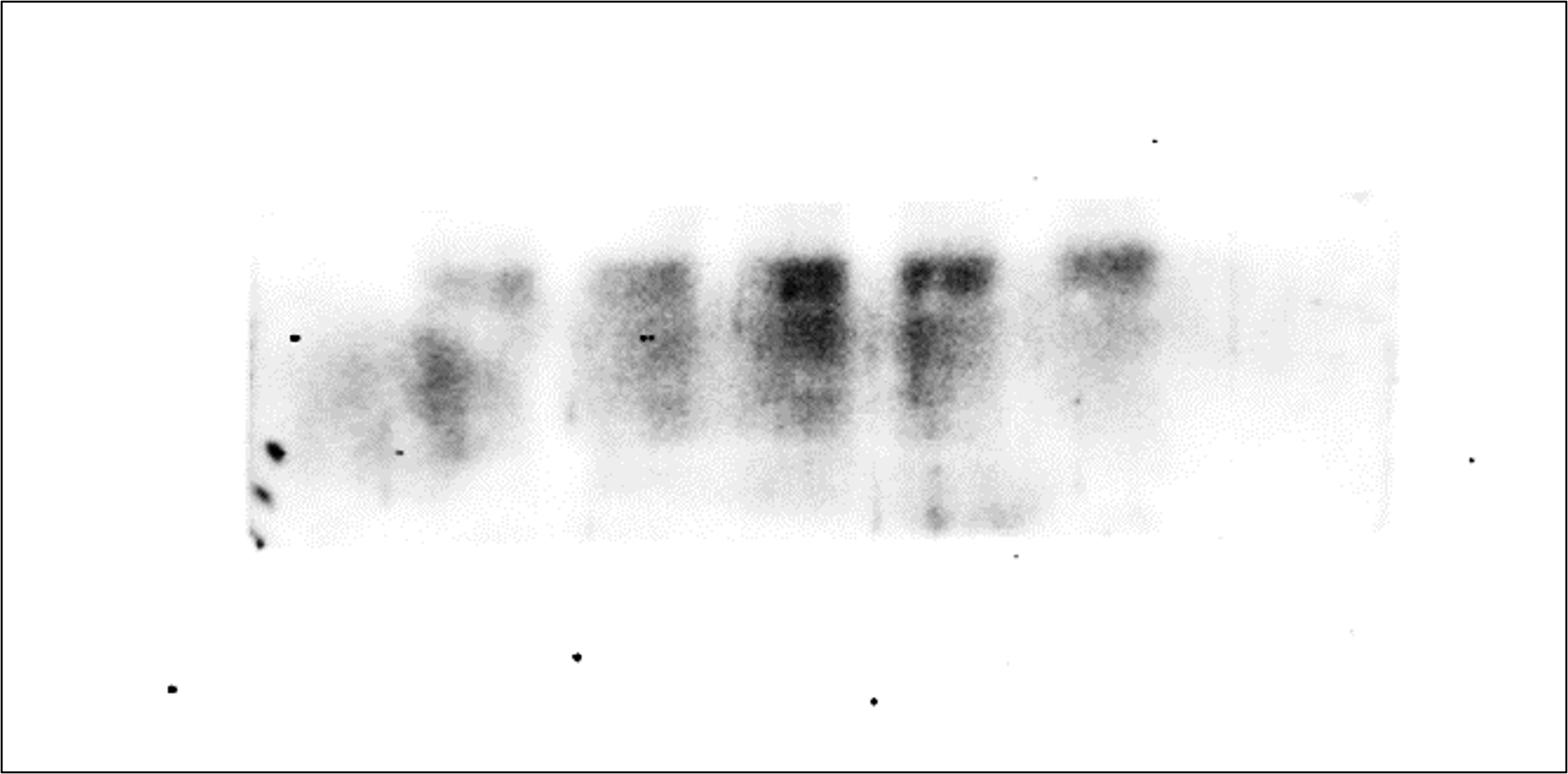

Supplement: Supplementary file 7 — original data [file 41420_2023_1589_MOESM7_ESM.zip › S6E-WB/igf2bp2.tif]

## Slide 1
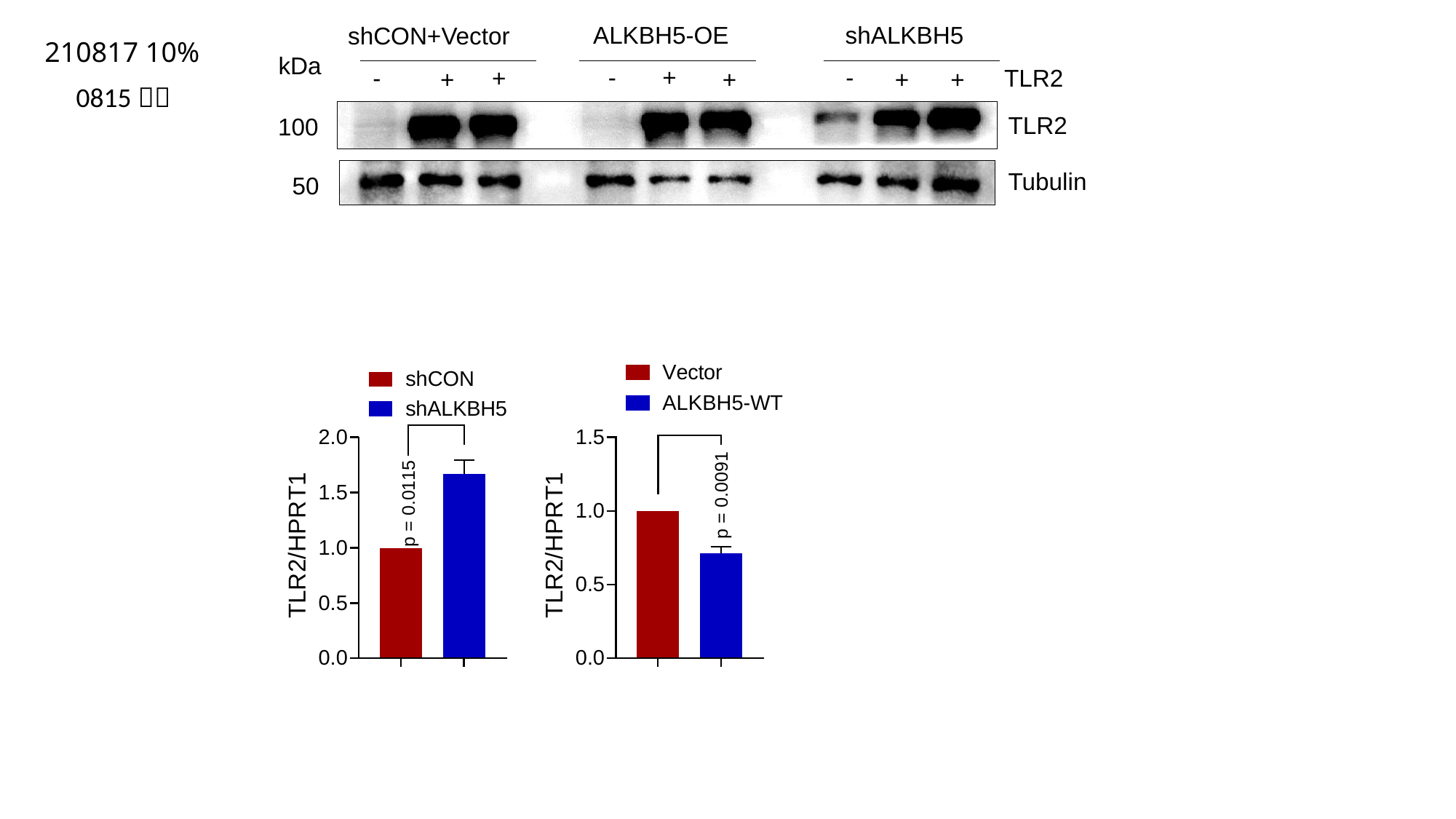

ALKBH5-OE
shALKBH5
shCON+Vector
kDa
 +
-
 +
-
 +
 +
TLR2
-
 +
 +
TLR2
100
Tubulin
50
210817 10%
0815蛋白

Supplement: Supplementary file 7 — original data [file 41420_2023_1589_MOESM7_ESM.zip › S4H-WB/New Microsoft PowerPoint Presentation.pptx]

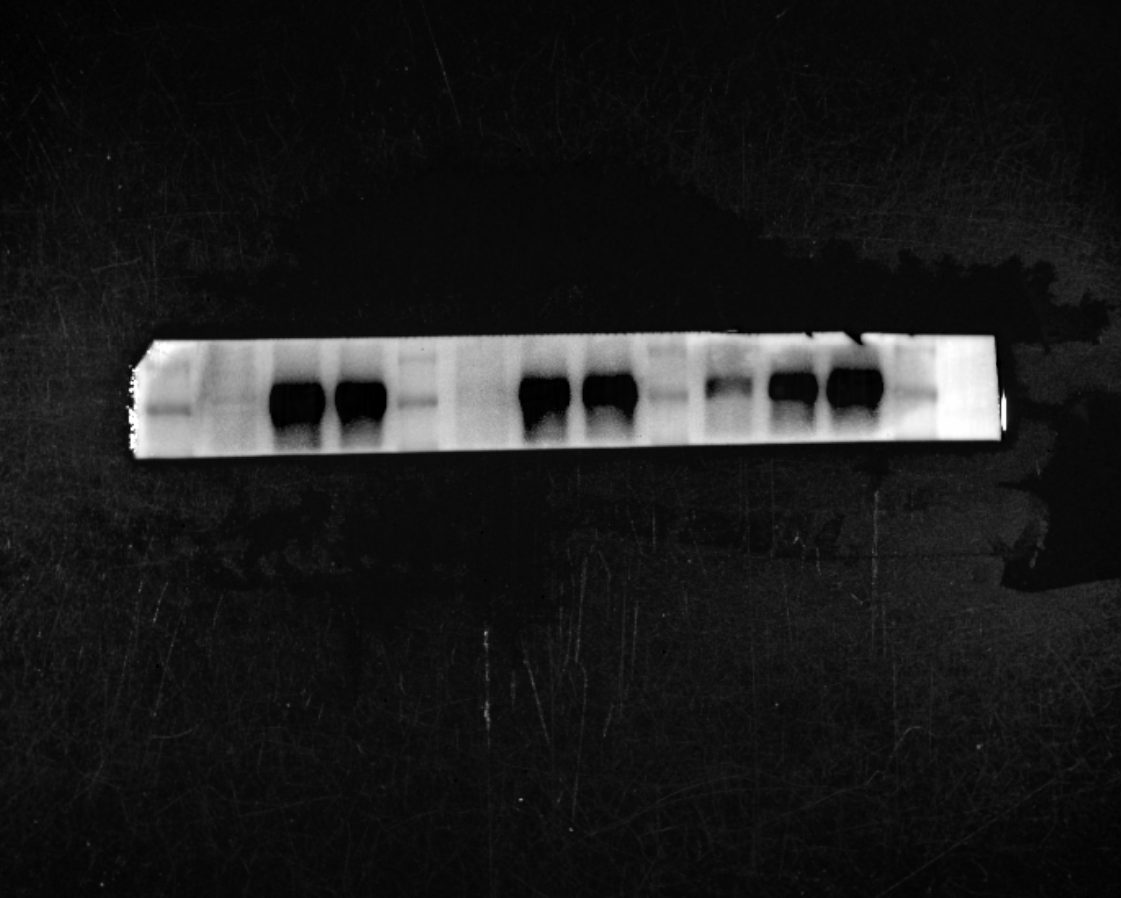

Supplement: Supplementary file 7 — original data [file 41420_2023_1589_MOESM7_ESM.zip › S4H-WB/page6-1 tlr2 210817 cxz 10% tlr2_2已用.tif]

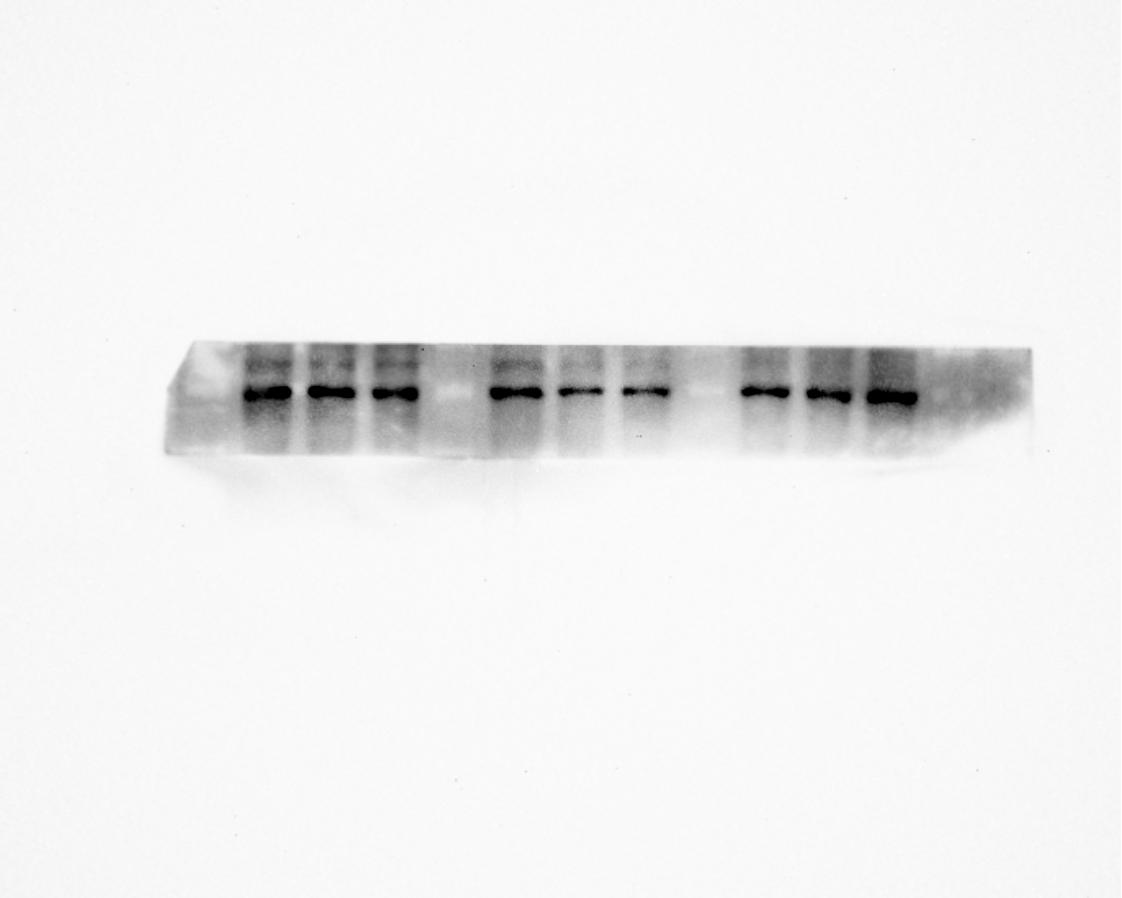

Supplement: Supplementary file 7 — original data [file 41420_2023_1589_MOESM7_ESM.zip › S4H-WB/page6-2 tubulin 没有merge 210817 cxz 10% tubulin已用.tif]

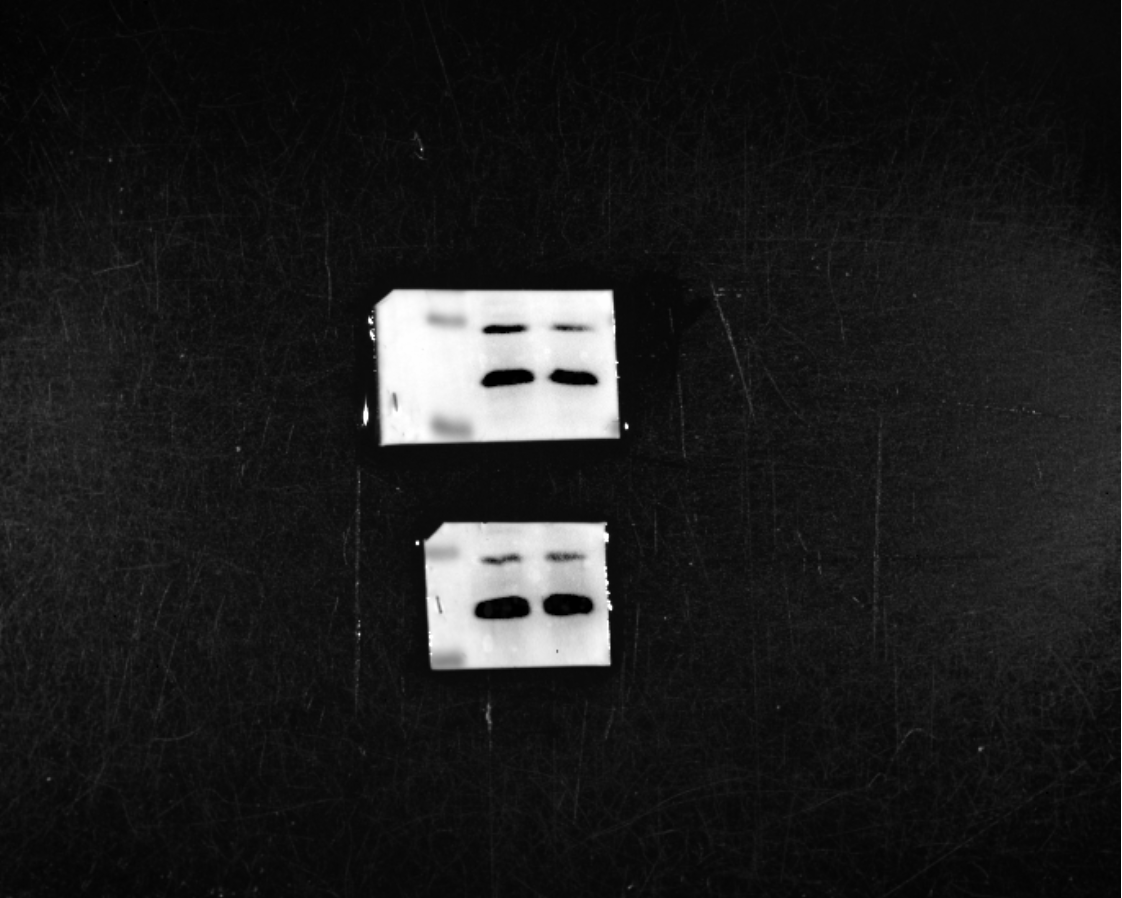

Supplement: Supplementary file 7 — original data [file 41420_2023_1589_MOESM7_ESM.zip › S4E-WB/page5-2 puma 210804 cxz 15% puma_2已用.tif]

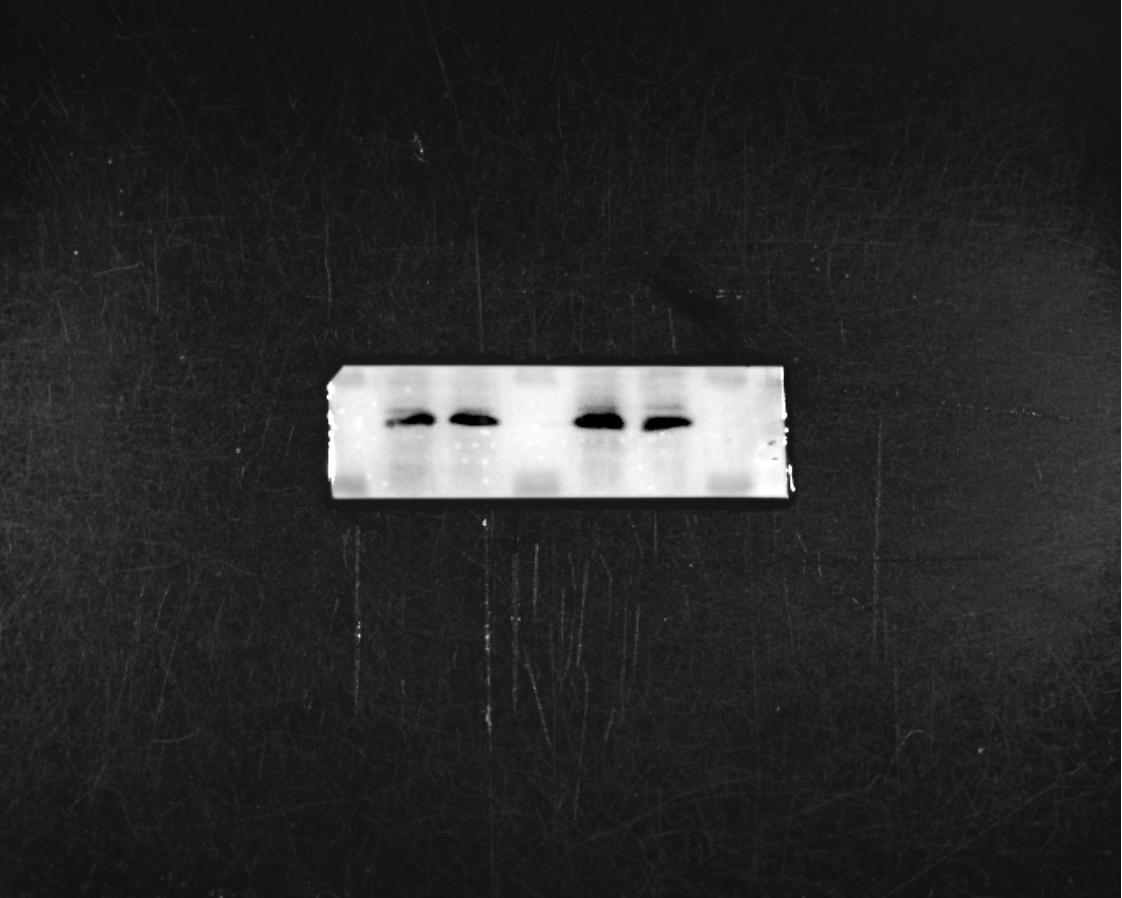

Supplement: Supplementary file 7 — original data [file 41420_2023_1589_MOESM7_ESM.zip › S4E-WB/page5-1 bax 210804 cxz 15% bax_2已用.tif]

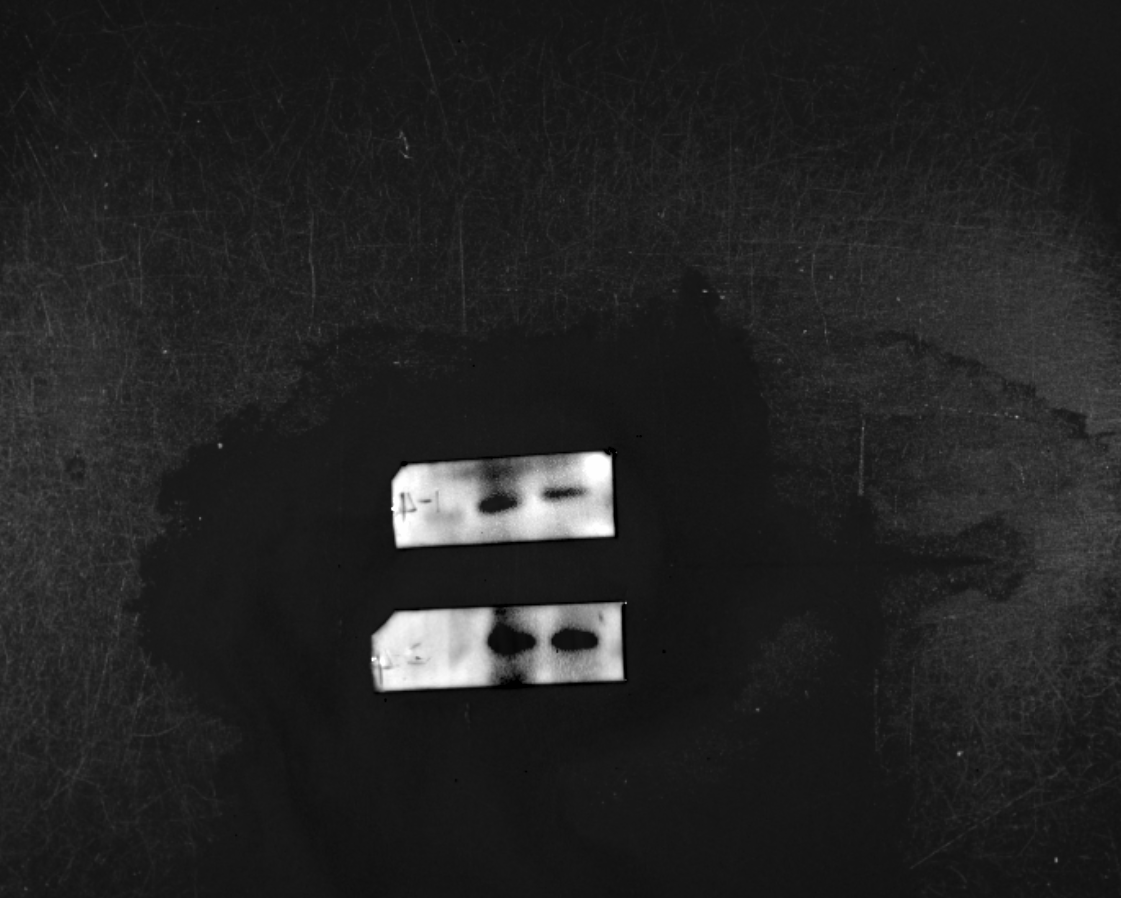

Supplement: Supplementary file 7 — original data [file 41420_2023_1589_MOESM7_ESM.zip › S4E-WB/page5-3 bcl-2 210803 cxz 10% bcl-2_2已用.tif]

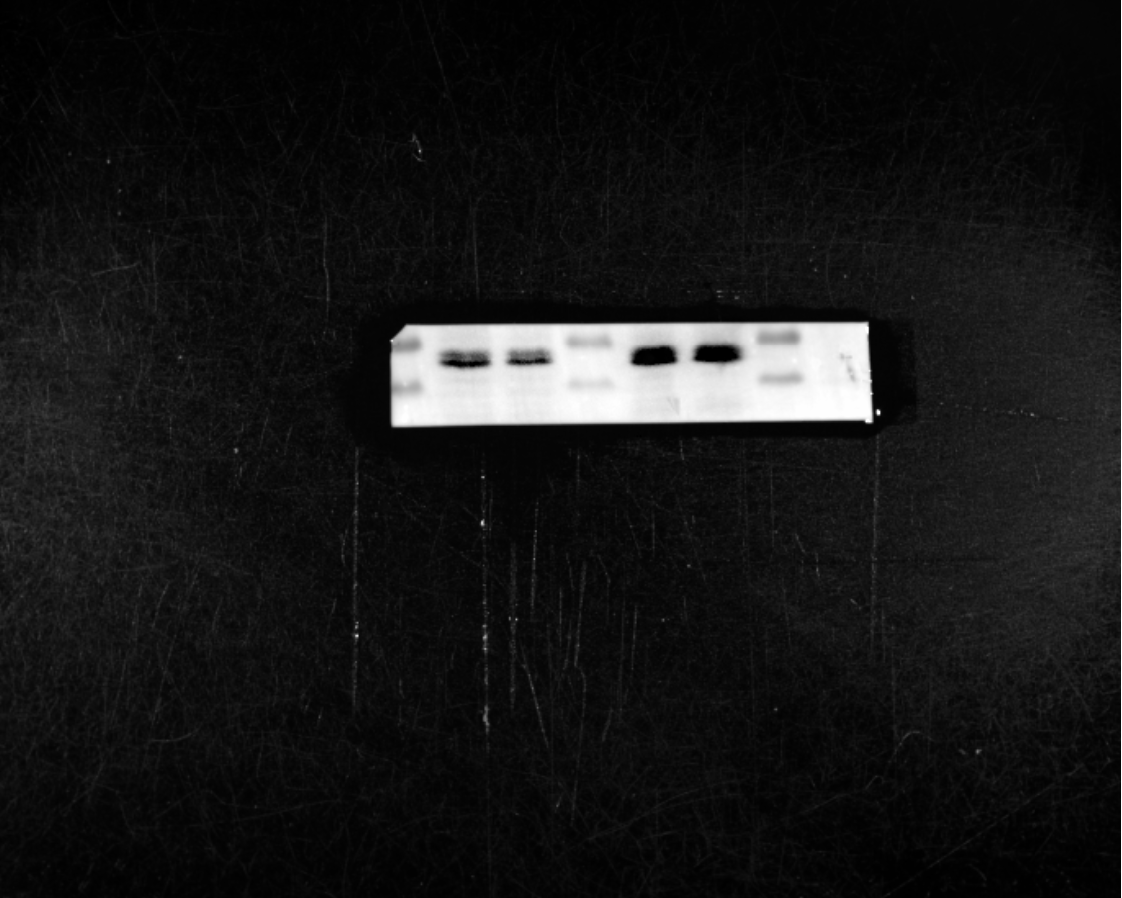

Supplement: Supplementary file 7 — original data [file 41420_2023_1589_MOESM7_ESM.zip › S4E-WB/page5-4 bcl-xl 210804 cxz 15% bcl-xl_2已用.tif]

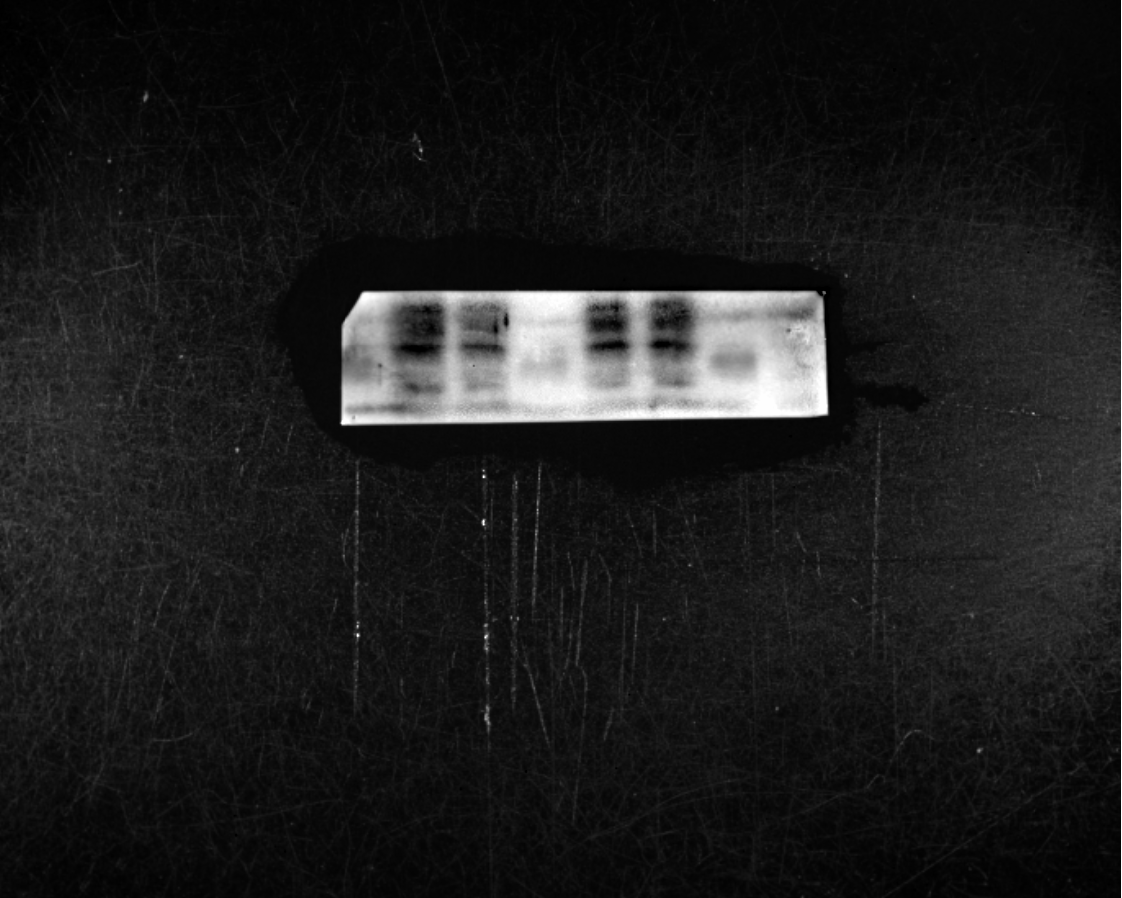

Supplement: Supplementary file 7 — original data [file 41420_2023_1589_MOESM7_ESM.zip › S4E-WB/page5-6 c-cas3 210804 cxz 15% c-caspase3_2已用.tif]

## Slide 1
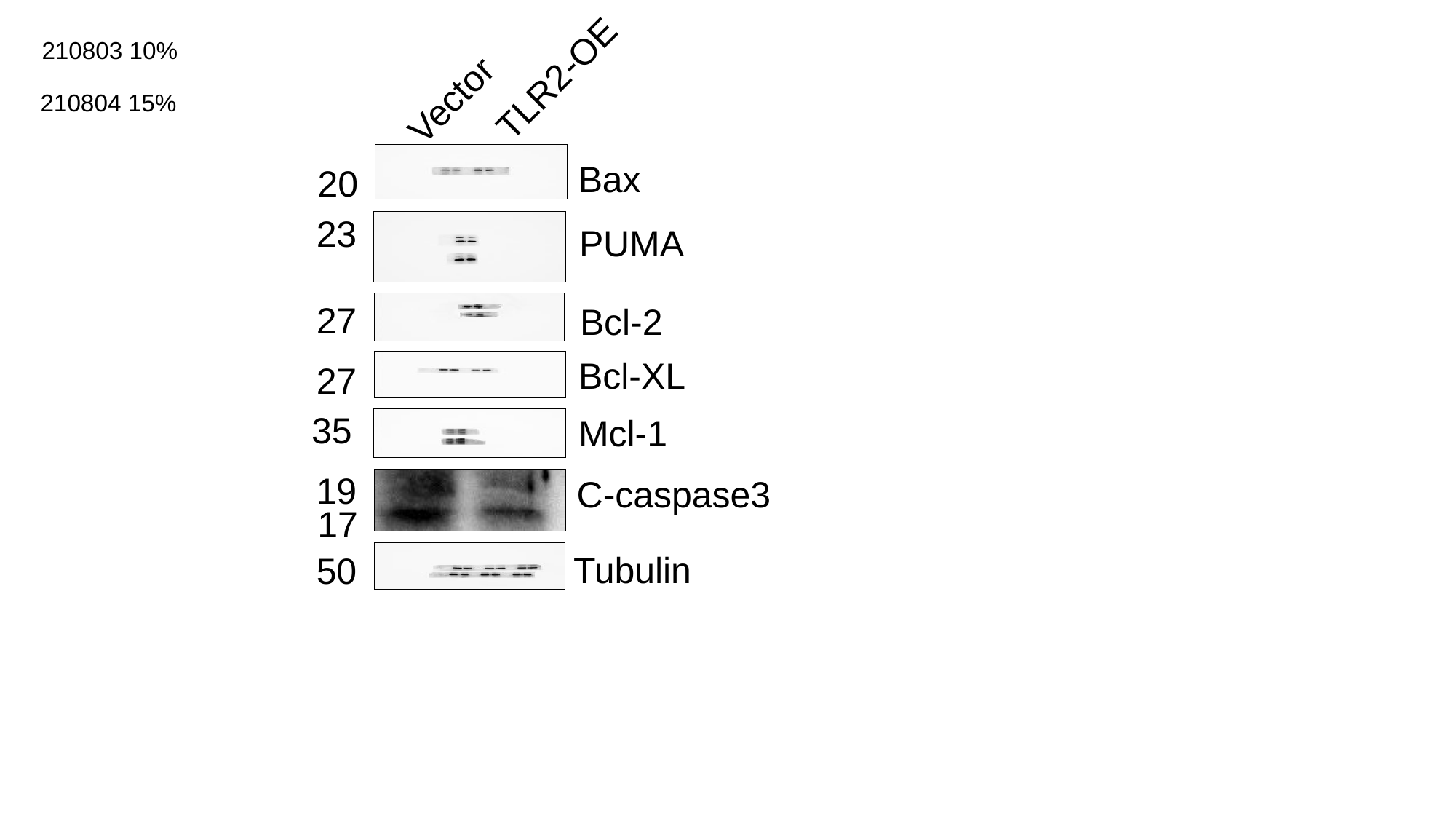

210803 10%
Vector
TLR2-OE
210804 15%
Bax
20
23
PUMA
27
Bcl-2
Bcl-XL
27
35
Mcl-1
19
C-caspase3
17
Tubulin
50

Supplement: Supplementary file 7 — original data [file 41420_2023_1589_MOESM7_ESM.zip › S4E-WB/New Microsoft PowerPoint Presentation.pptx]

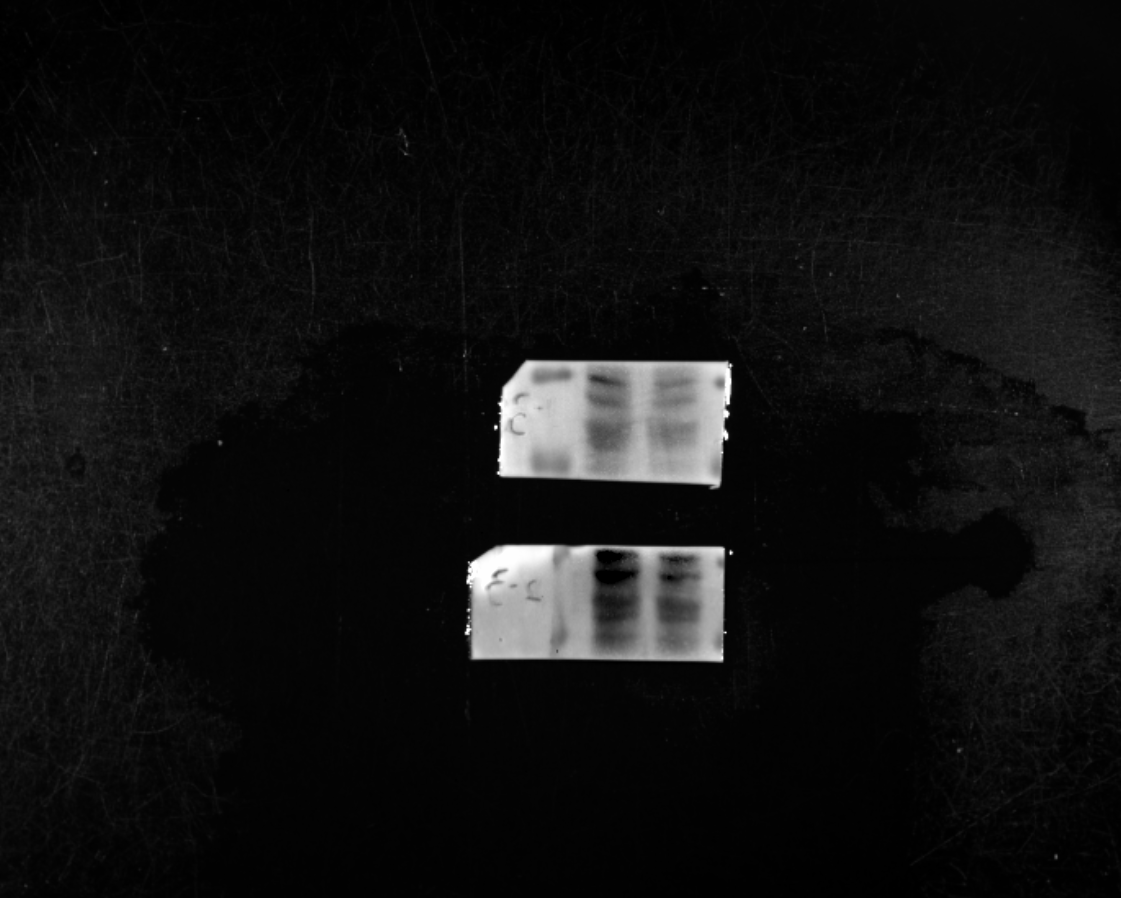

Supplement: Supplementary file 7 — original data [file 41420_2023_1589_MOESM7_ESM.zip › S4E-WB/page5-5 mcl-1 210803 cxz 10% mcl-1_2已用.tif]

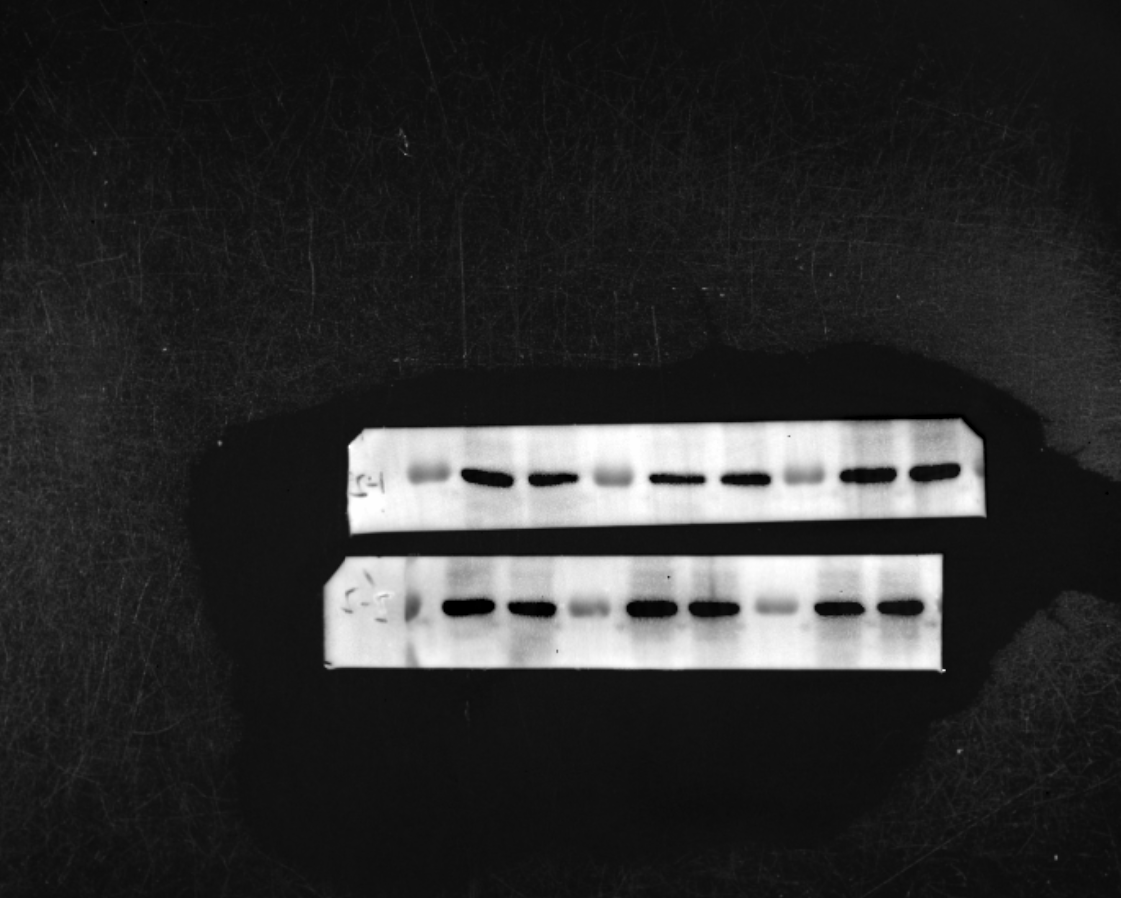

Supplement: Supplementary file 7 — original data [file 41420_2023_1589_MOESM7_ESM.zip › S4E-WB/page10-8=5-7 tubulin 210803 cxz 10% tubulin_2已用.tif]

## Slide 1
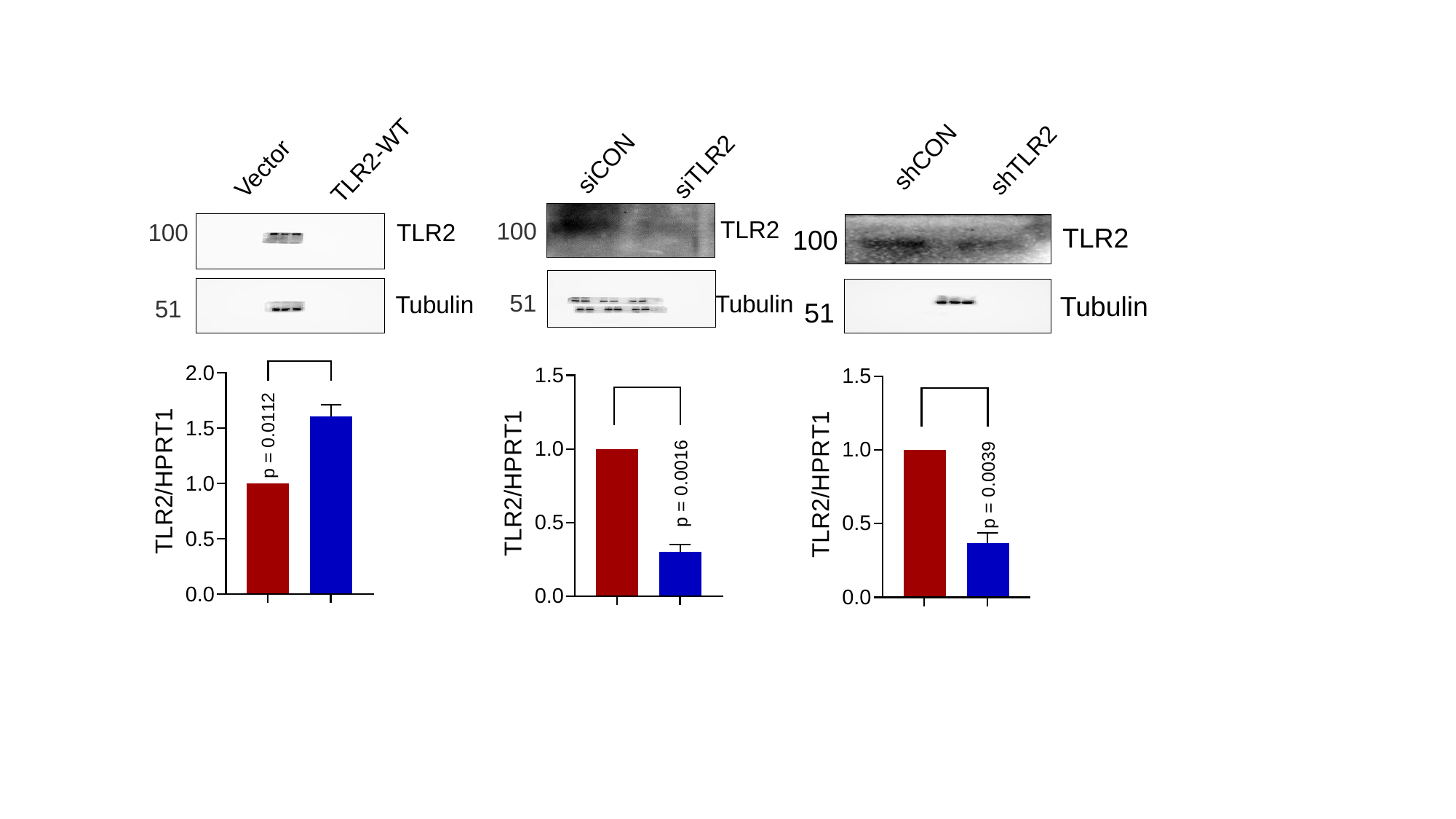

siTLR2
siCON
TLR2
100
51
Tubulin
TLR2-WT
Vector
100
TLR2
Tubulin
51
shTLR2
shCON
TLR2
100
Tubulin
51

Supplement: Supplementary file 7 — original data [file 41420_2023_1589_MOESM7_ESM.zip › S4A-WB/New Microsoft PowerPoint Presentation.pptx]

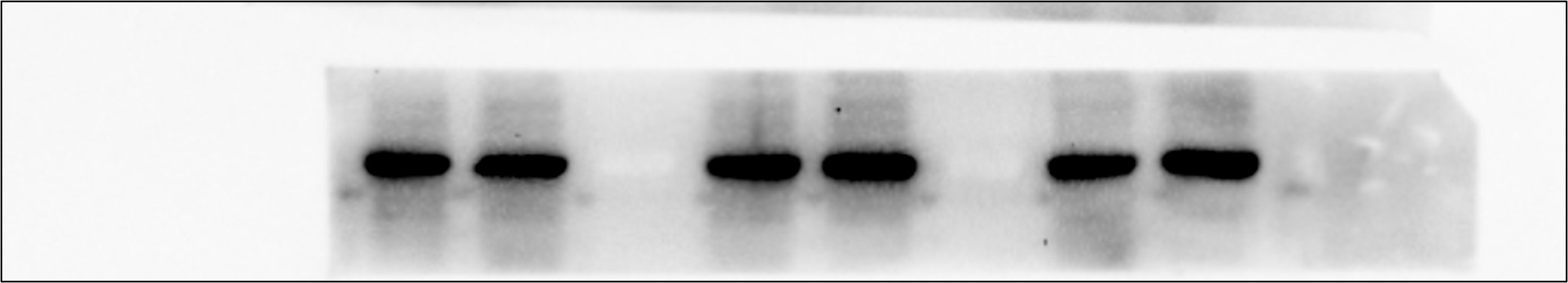

Supplement: Supplementary file 7 — original data [file 41420_2023_1589_MOESM7_ESM.zip › S4A-WB/tubulin -中间.tif]

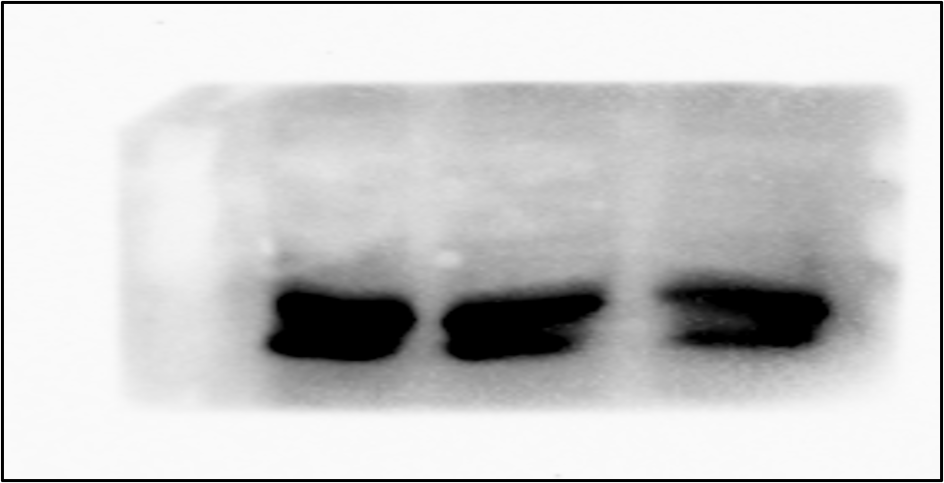

Supplement: Supplementary file 7 — original data [file 41420_2023_1589_MOESM7_ESM.zip › S4A-WB/tubulin左边.tif]

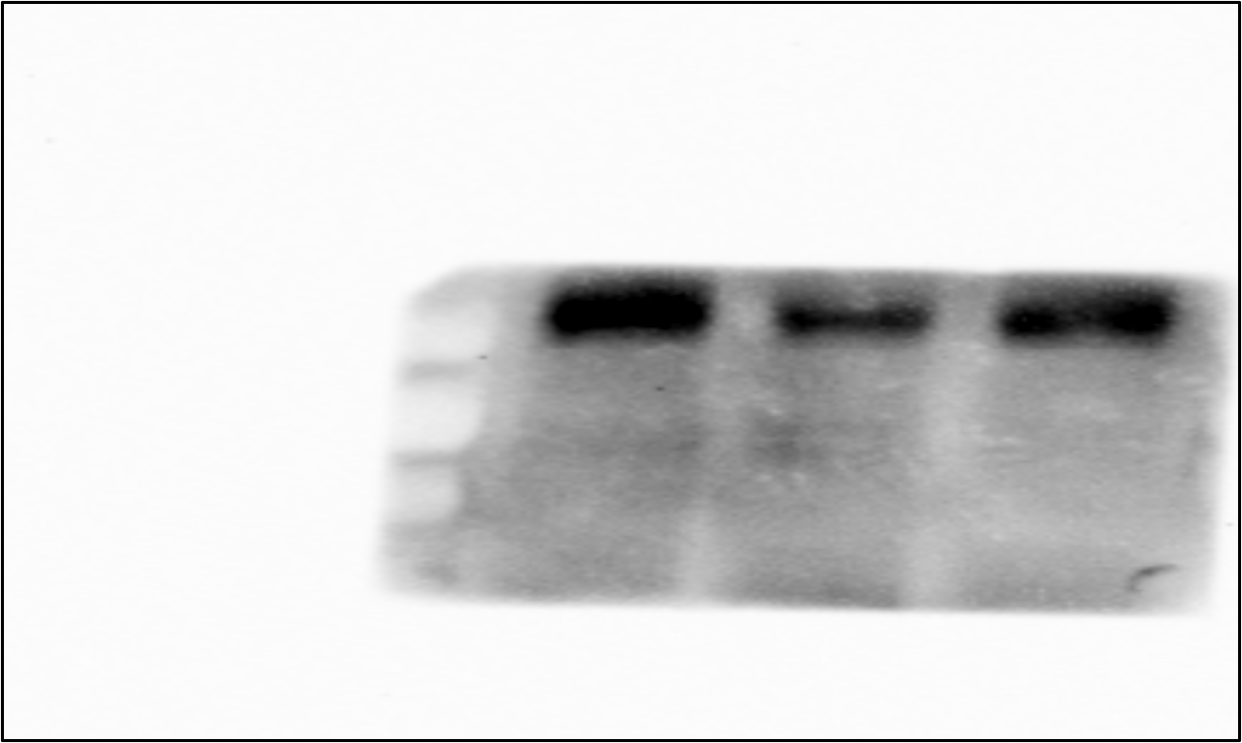

Supplement: Supplementary file 7 — original data [file 41420_2023_1589_MOESM7_ESM.zip › S4A-WB/TLR2左边.tif]

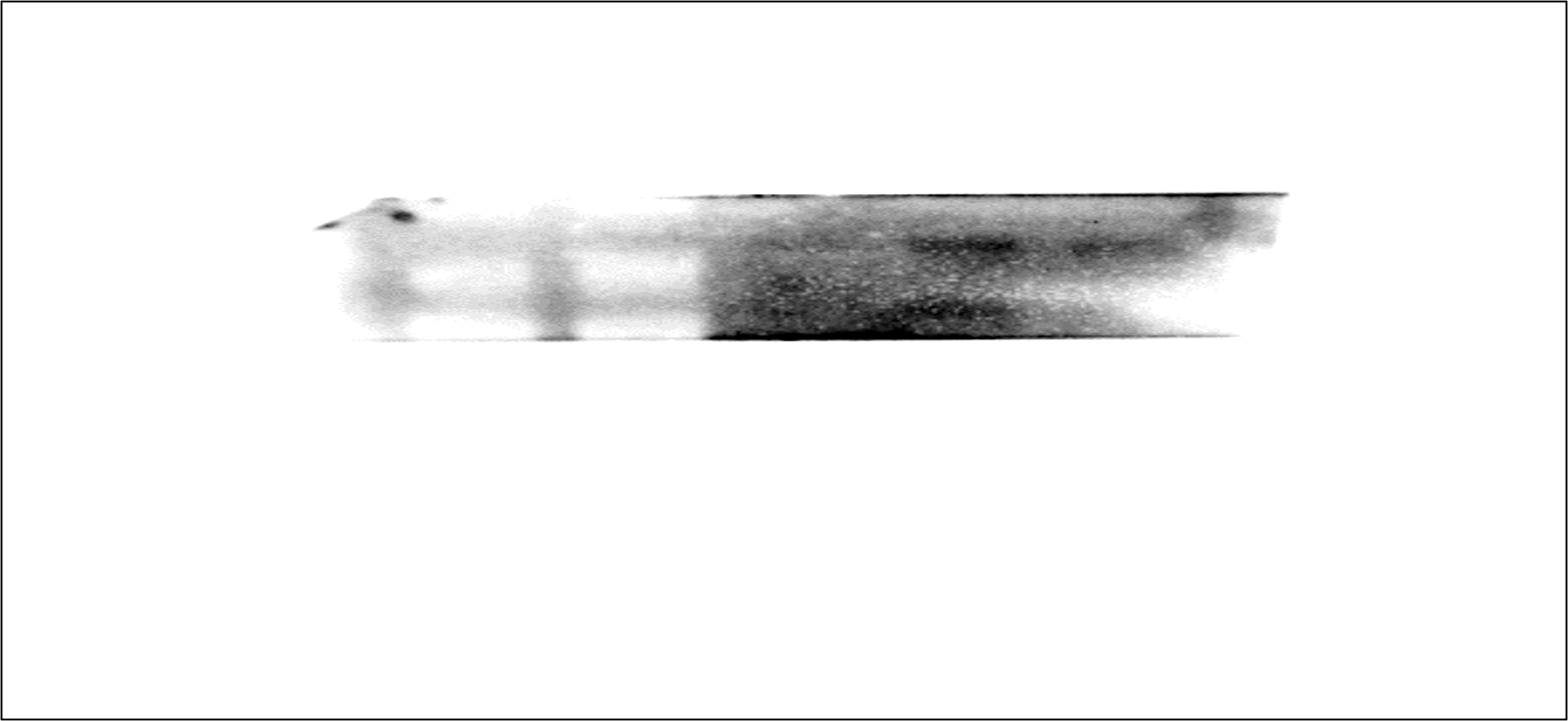

Supplement: Supplementary file 7 — original data [file 41420_2023_1589_MOESM7_ESM.zip › S4A-WB/TLR2右边.tif]

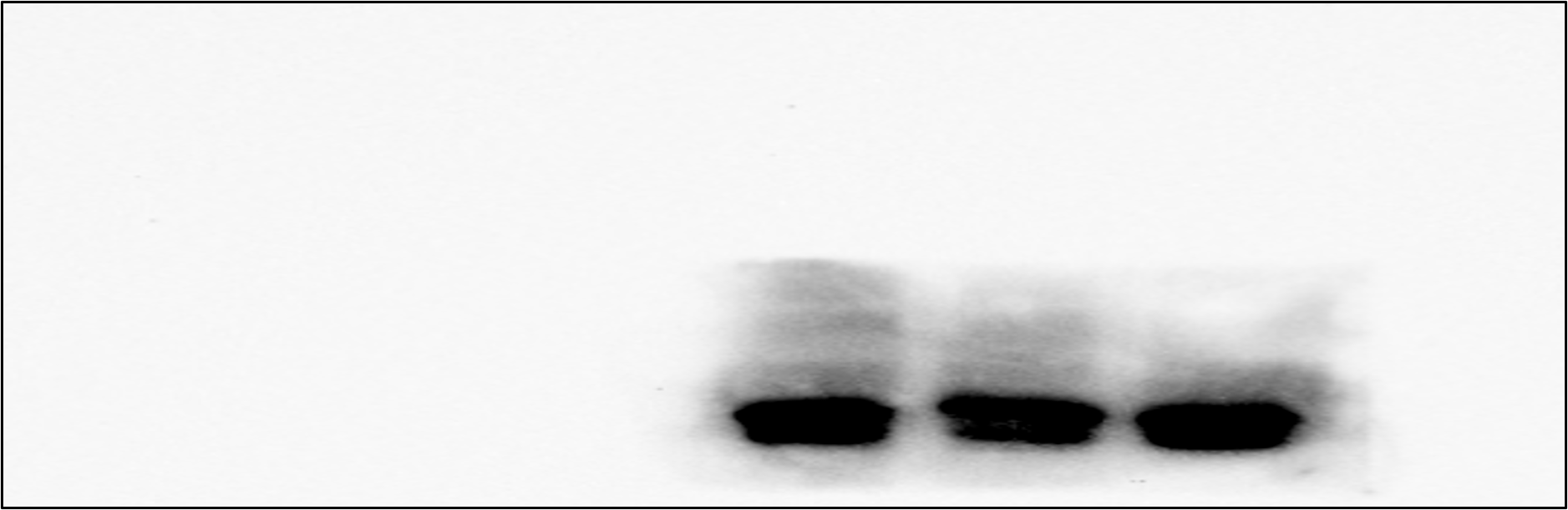

Supplement: Supplementary file 7 — original data [file 41420_2023_1589_MOESM7_ESM.zip › S4A-WB/tubulin右边.tif]

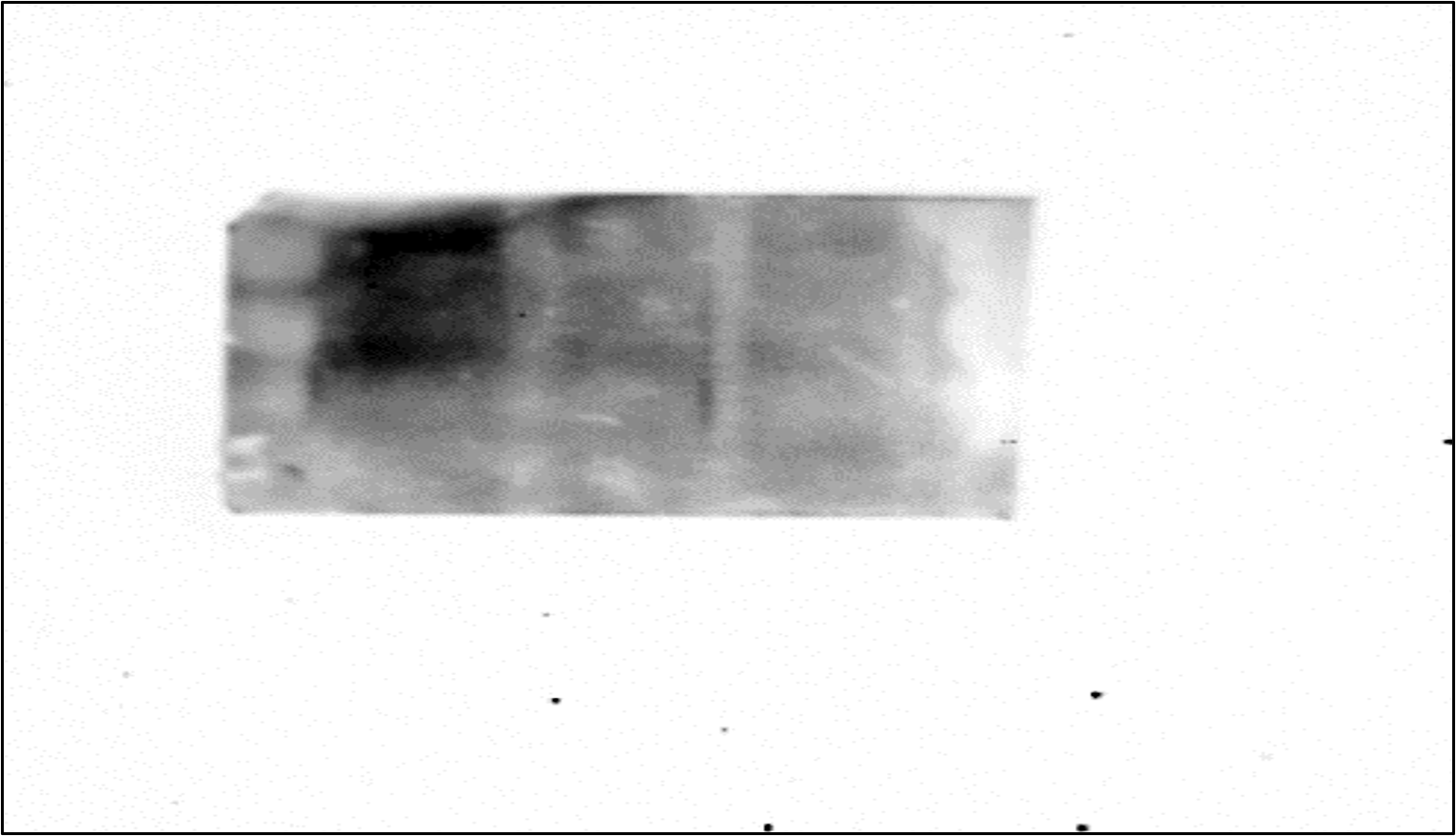

Supplement: Supplementary file 7 — original data [file 41420_2023_1589_MOESM7_ESM.zip › S4A-WB/TLR2 -中间.tif]

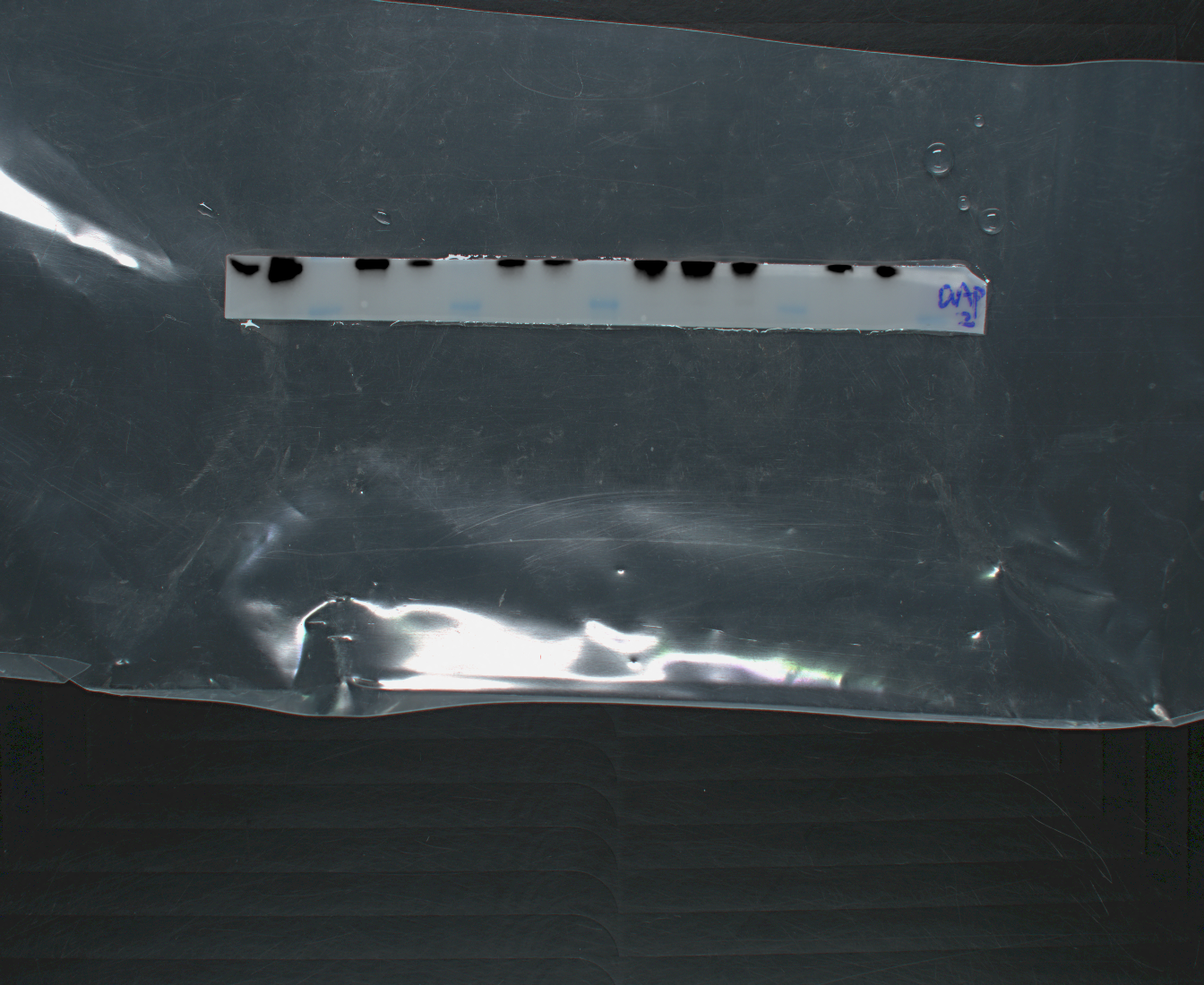

Supplement: Supplementary file 7 — original data [file 41420_2023_1589_MOESM7_ESM.zip › S1C-WB/2022-11-15 GAPDH (2).Tif]

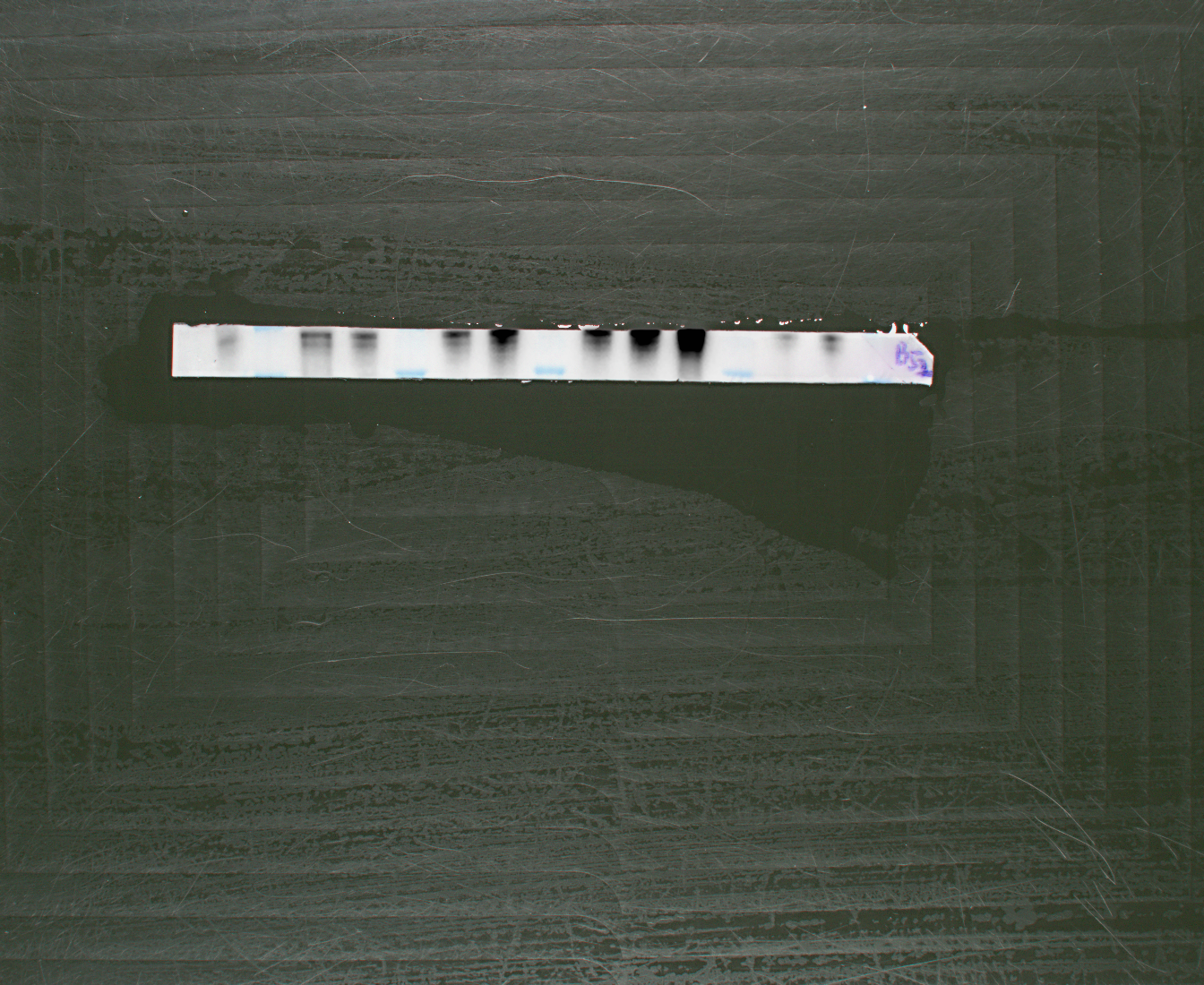

Supplement: Supplementary file 7 — original data [file 41420_2023_1589_MOESM7_ESM.zip › S1C-WB/2022-11-15 ALKBH5(2) .Tif]

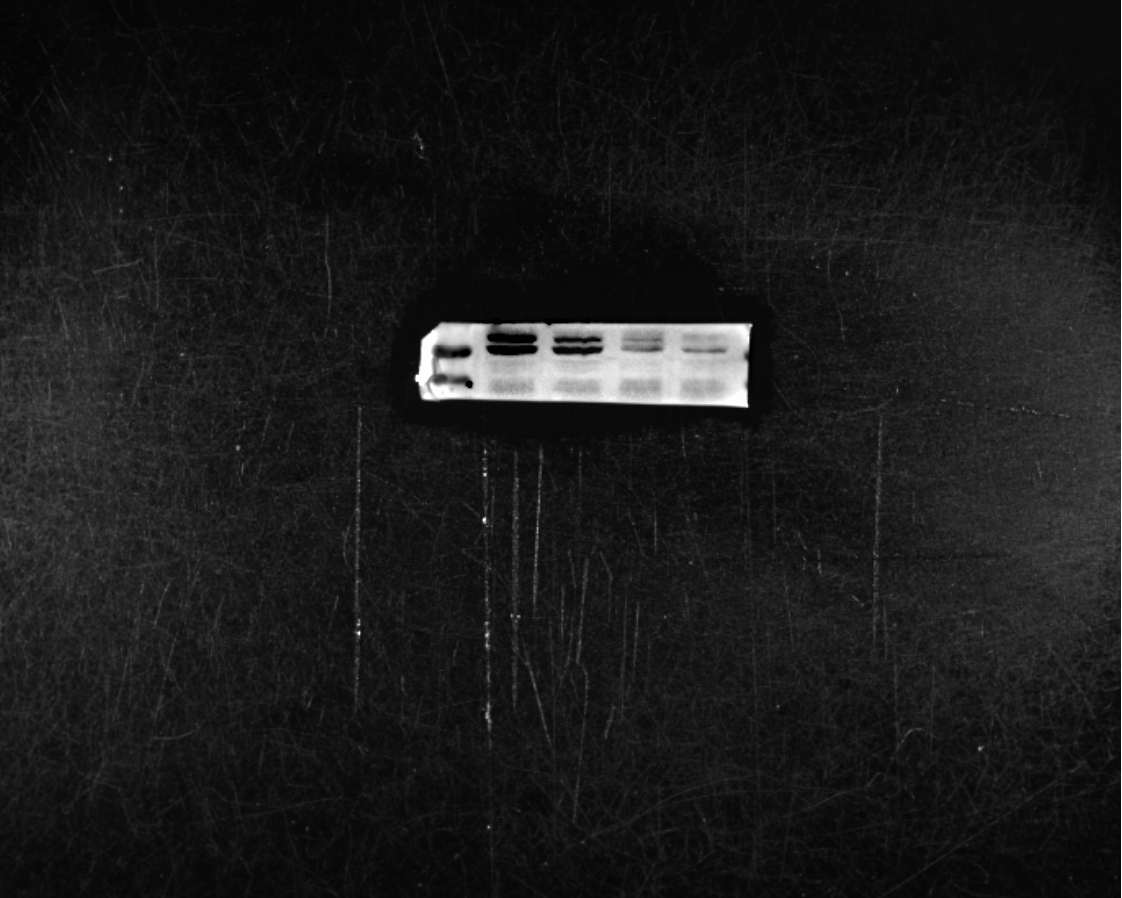

Supplement: Supplementary file 7 — original data [file 41420_2023_1589_MOESM7_ESM.zip › 6M-WB/page10-6 flip 210820 cxz 15% flip_2已用.tif]

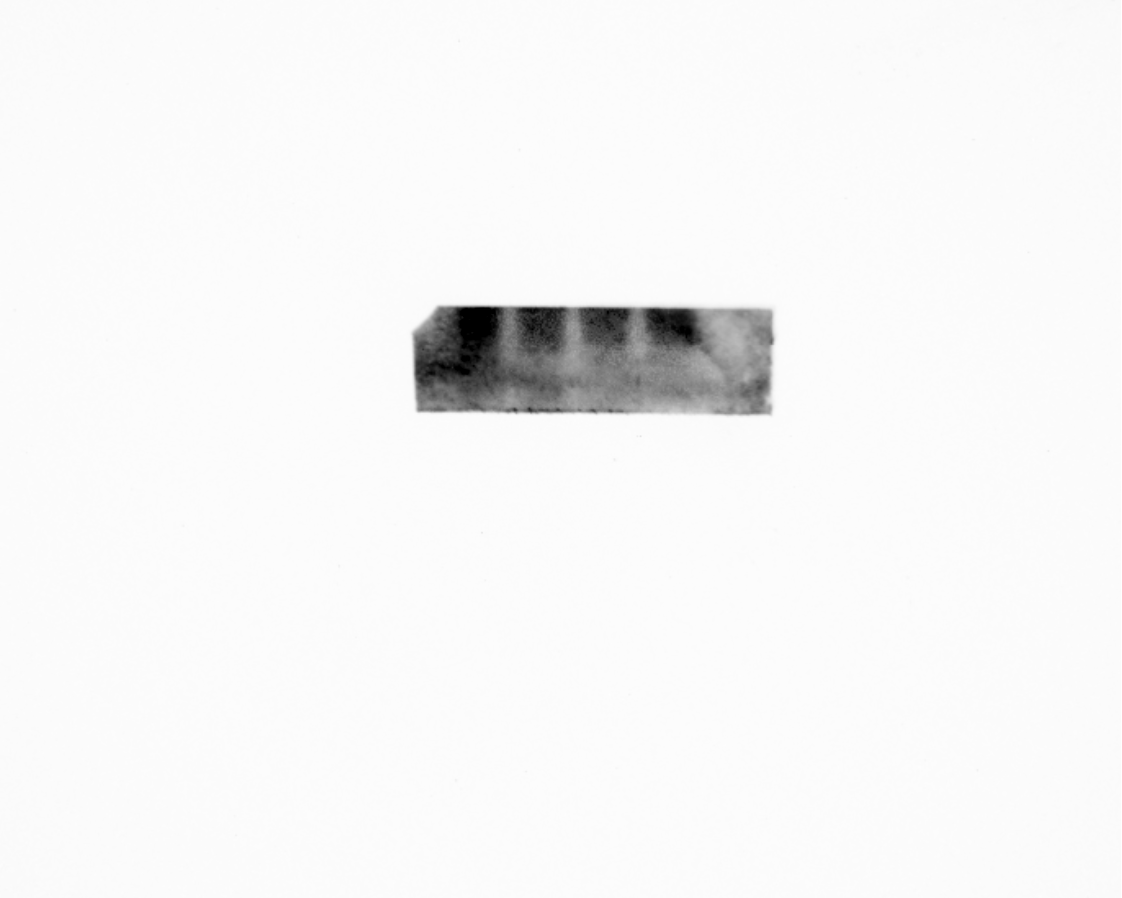

Supplement: Supplementary file 7 — original data [file 41420_2023_1589_MOESM7_ESM.zip › 6M-WB/page16-4 tp53inp1 没有merge 210730 cxz 10% tp53inp1_4已用.tif]

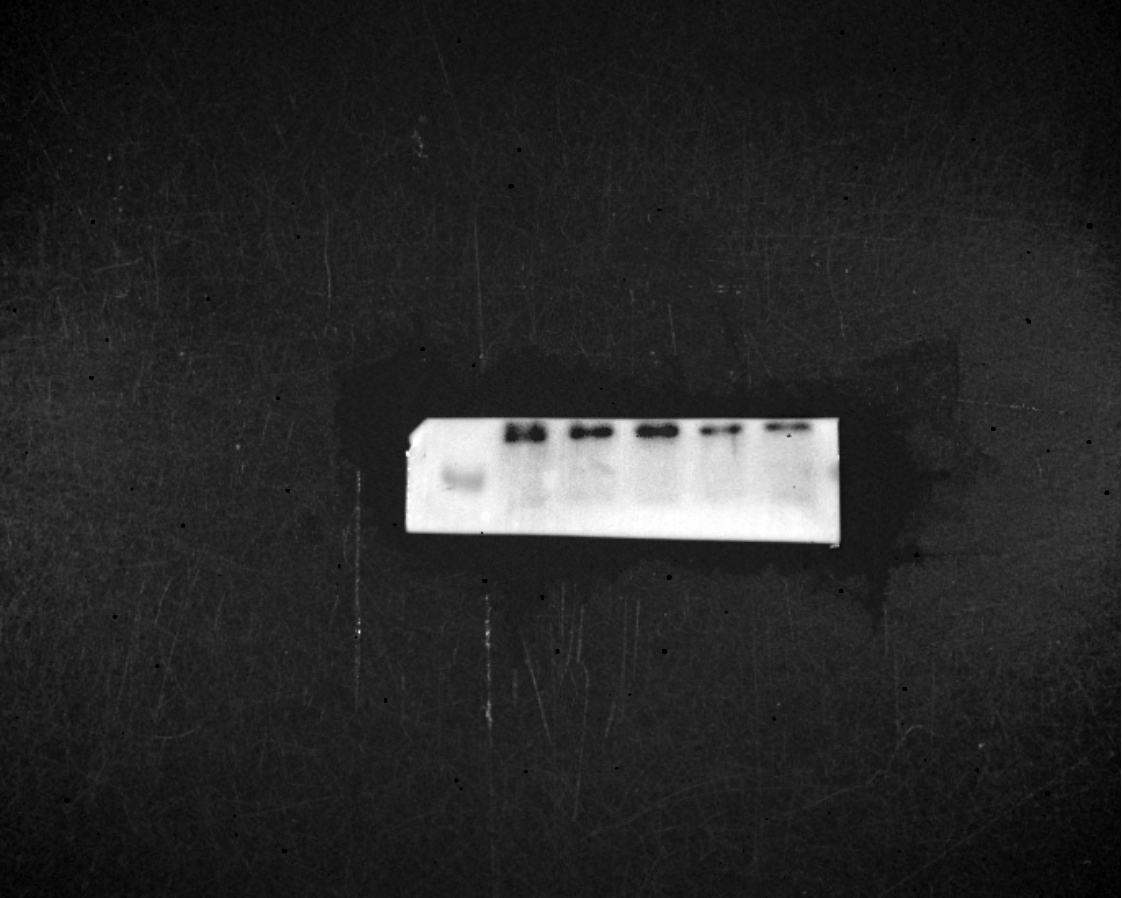

Supplement: Supplementary file 7 — original data [file 41420_2023_1589_MOESM7_ESM.zip › 6M-WB/page16-6 bcl-xl 210826 cxz 10% bcl-xl_2已用.tif]

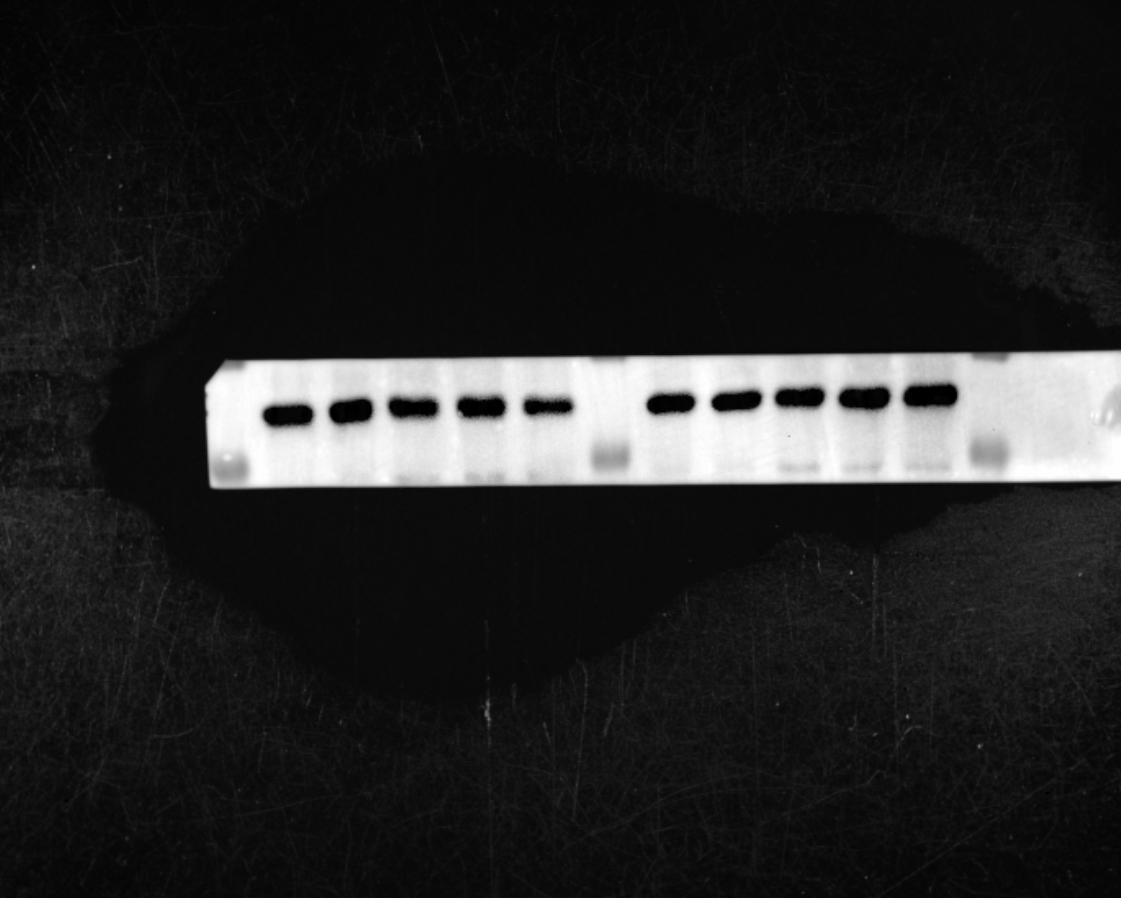

Supplement: Supplementary file 7 — original data [file 41420_2023_1589_MOESM7_ESM.zip › 6M-WB/page16-7 gapdh 210824 cxz 10% gapdh_2已用.tif]

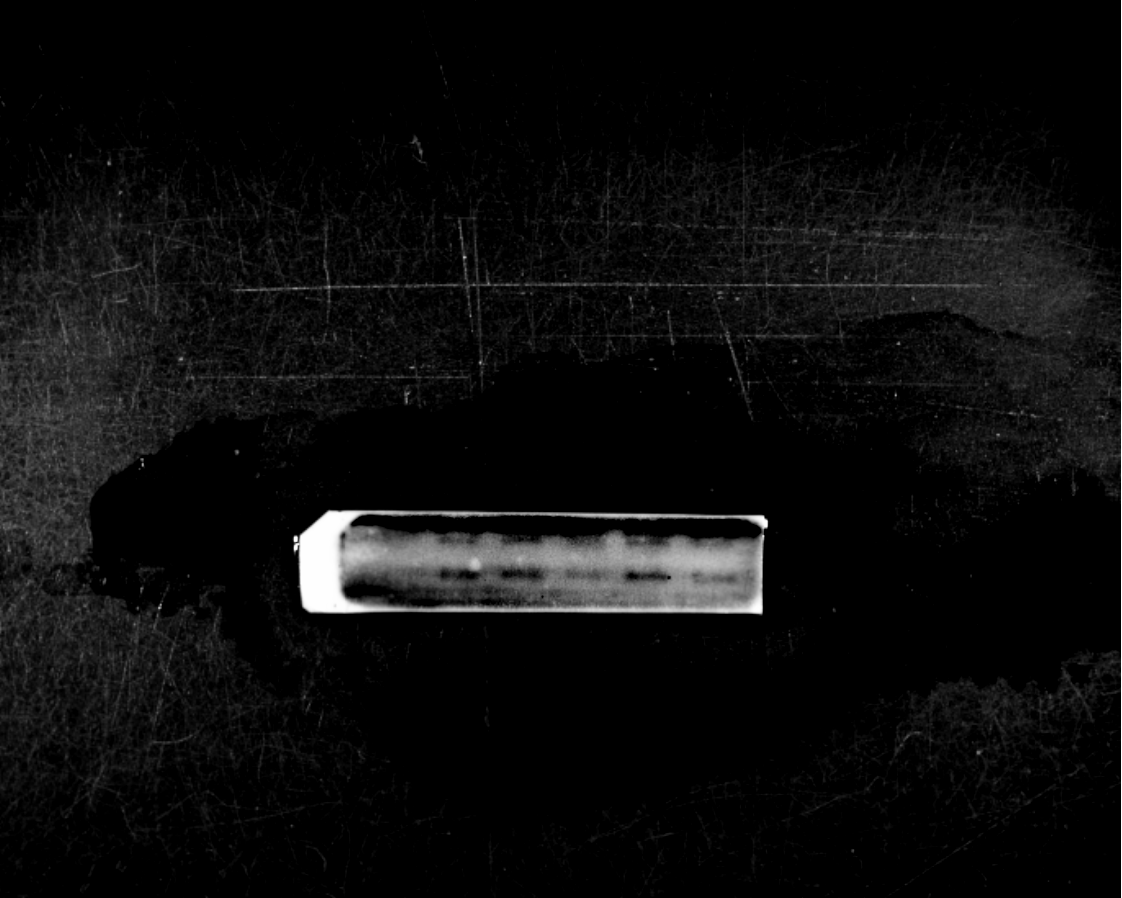

Supplement: Supplementary file 7 — original data [file 41420_2023_1589_MOESM7_ESM.zip › 5M-WB 完成/page19-1 bcl-2 210915 cxz 10% itgfbp2_2已用.tif]

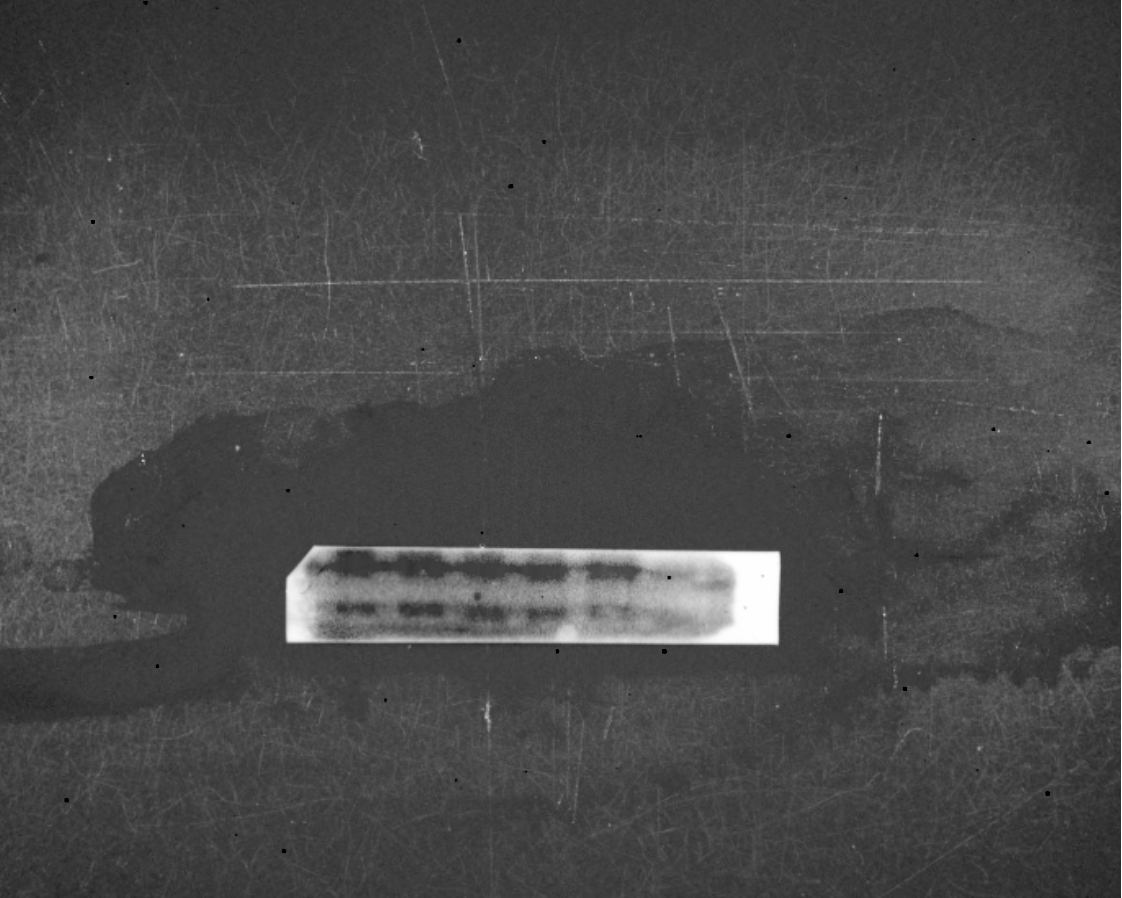

Supplement: Supplementary file 7 — original data [file 41420_2023_1589_MOESM7_ESM.zip › 5M-WB 完成/page19-2 bcl-xl 210915 cxz 10%itgfbp3_2已用.tif]

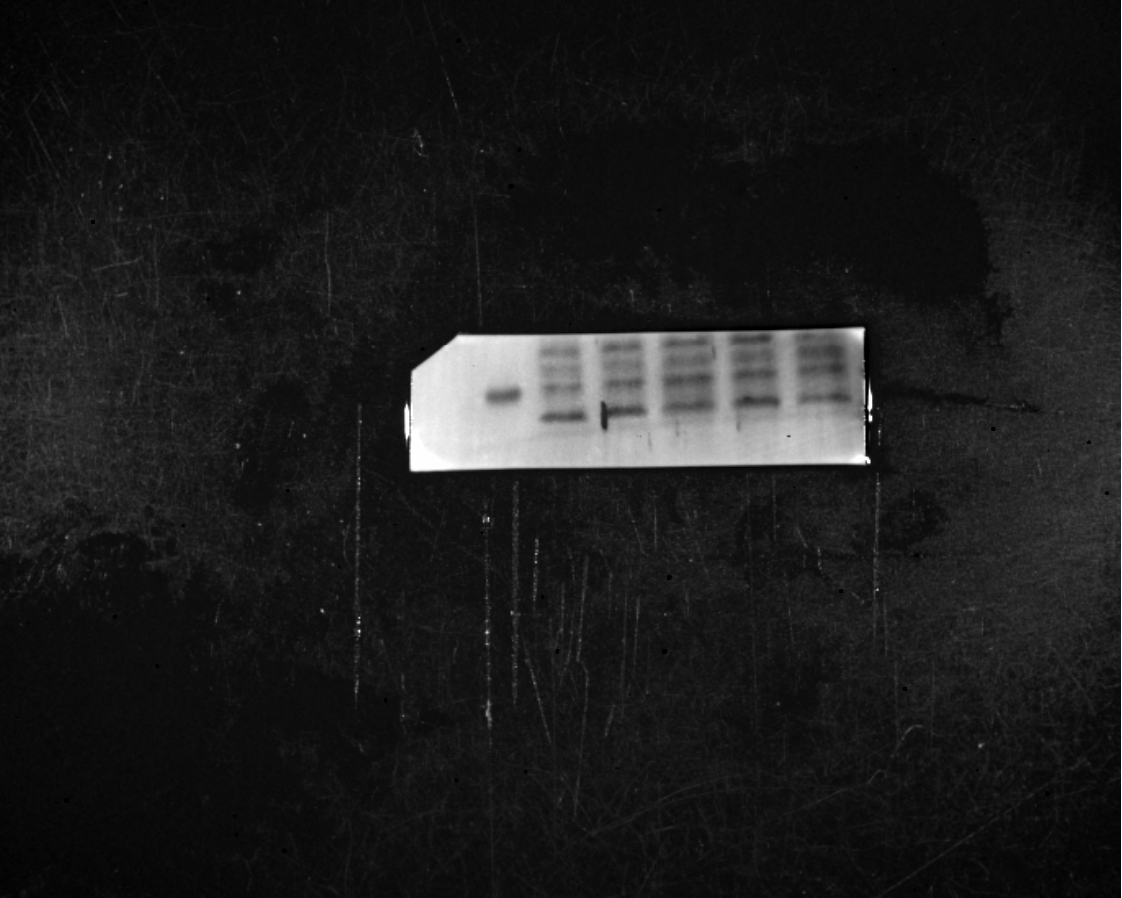

Supplement: Supplementary file 7 — original data [file 41420_2023_1589_MOESM7_ESM.zip › 5M-WB 完成/page19-4 c-cas3 210825 cxz 15% c-csapase3_2已用.tif]

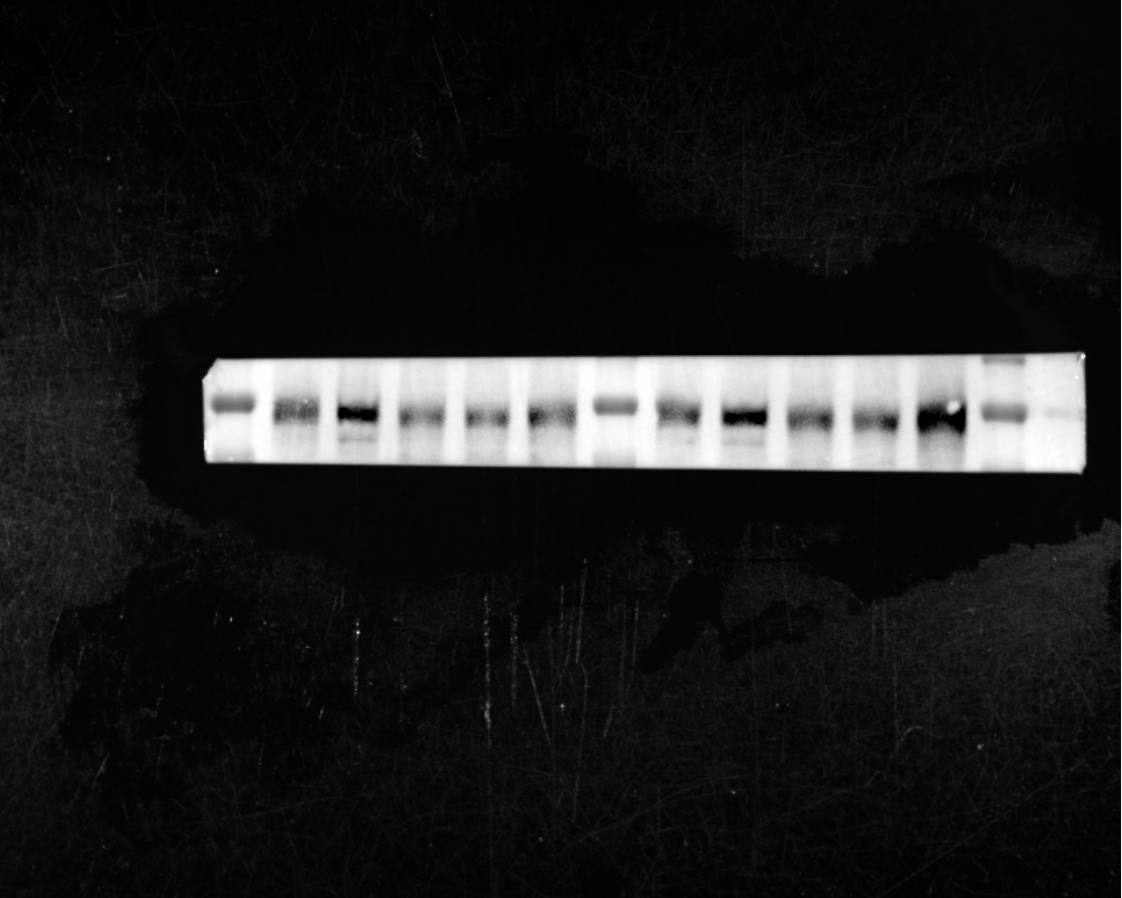

Supplement: Supplementary file 7 — original data [file 41420_2023_1589_MOESM7_ESM.zip › 5M-WB 完成/page19-5 tnfaip3210824 cxz 10% tnfaip3_2已用.tif]

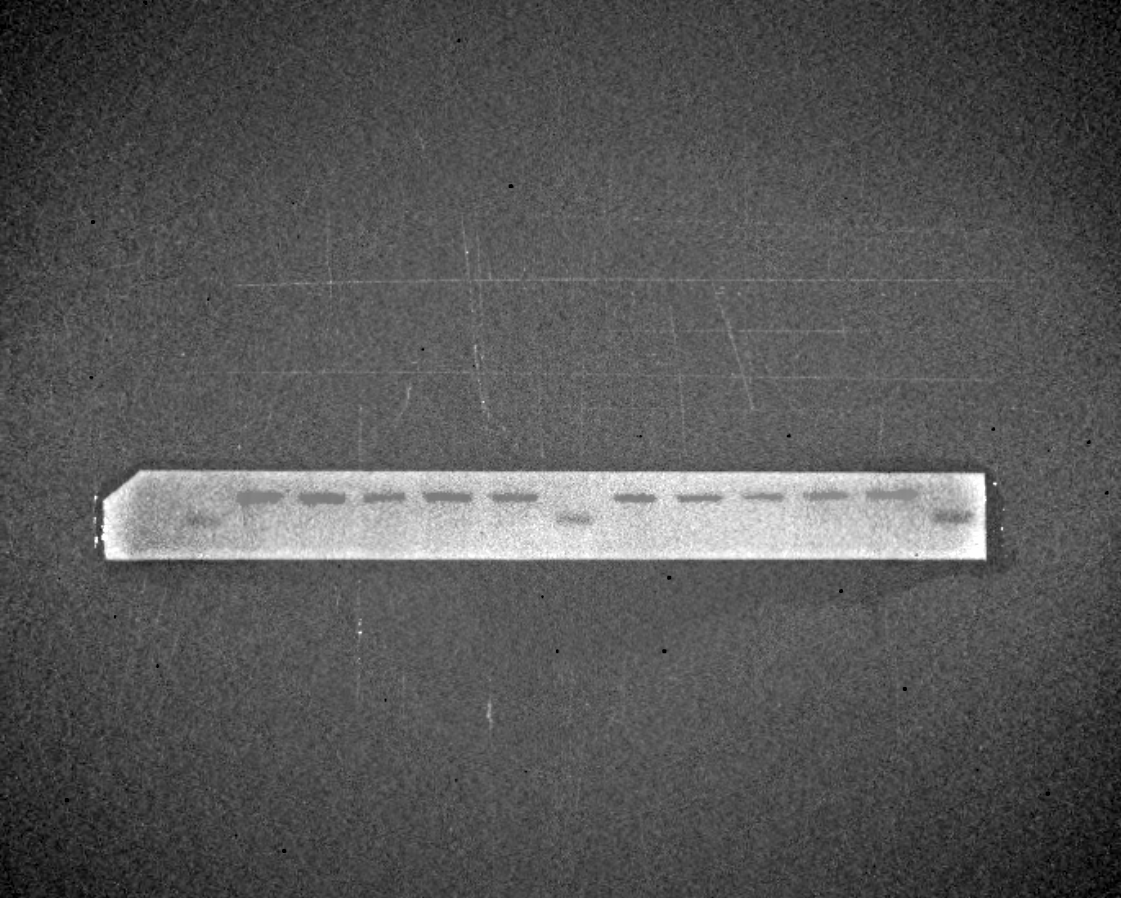

Supplement: Supplementary file 7 — original data [file 41420_2023_1589_MOESM7_ESM.zip › 5M-WB 完成/page19-7 bactin 210915 cxz 10% bactin_2已用.tif]

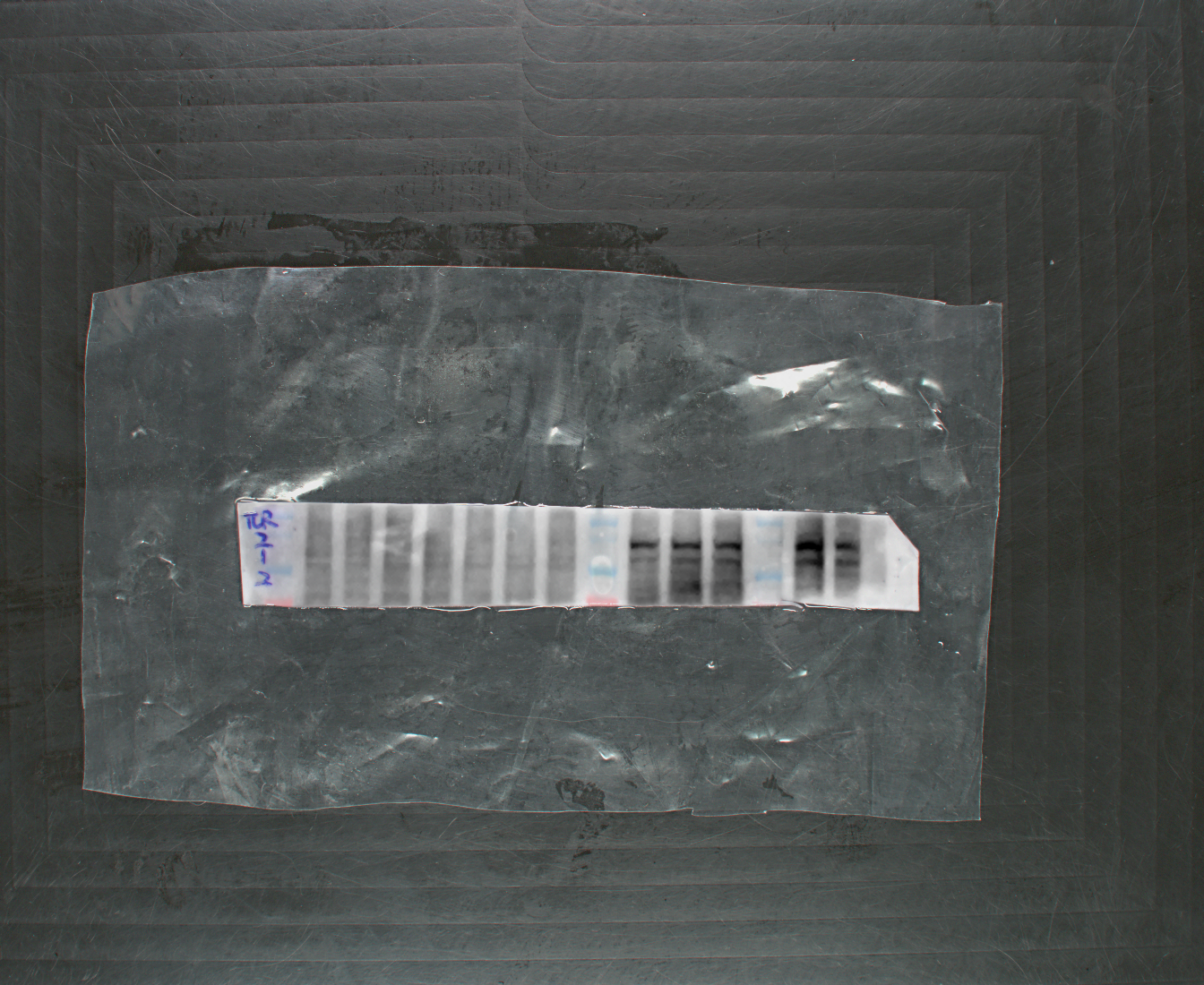

Supplement: Supplementary file 7 — original data [file 41420_2023_1589_MOESM7_ESM.zip › 5D-WB 完成/2022-11-19 TLR2.Tif]

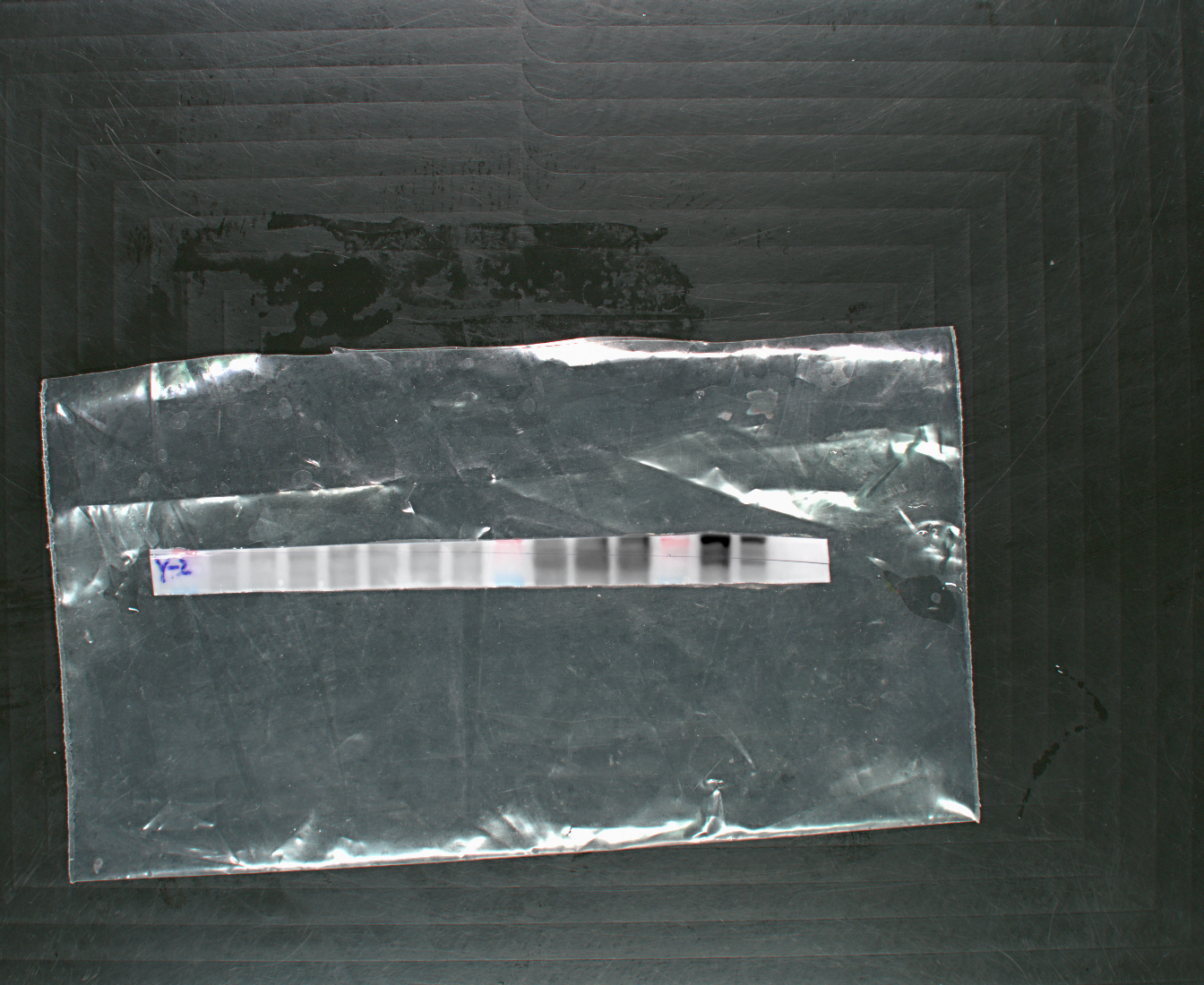

Supplement: Supplementary file 7 — original data [file 41420_2023_1589_MOESM7_ESM.zip › 5D-WB 完成/2022-11-19 YTHDF1 .Tif]

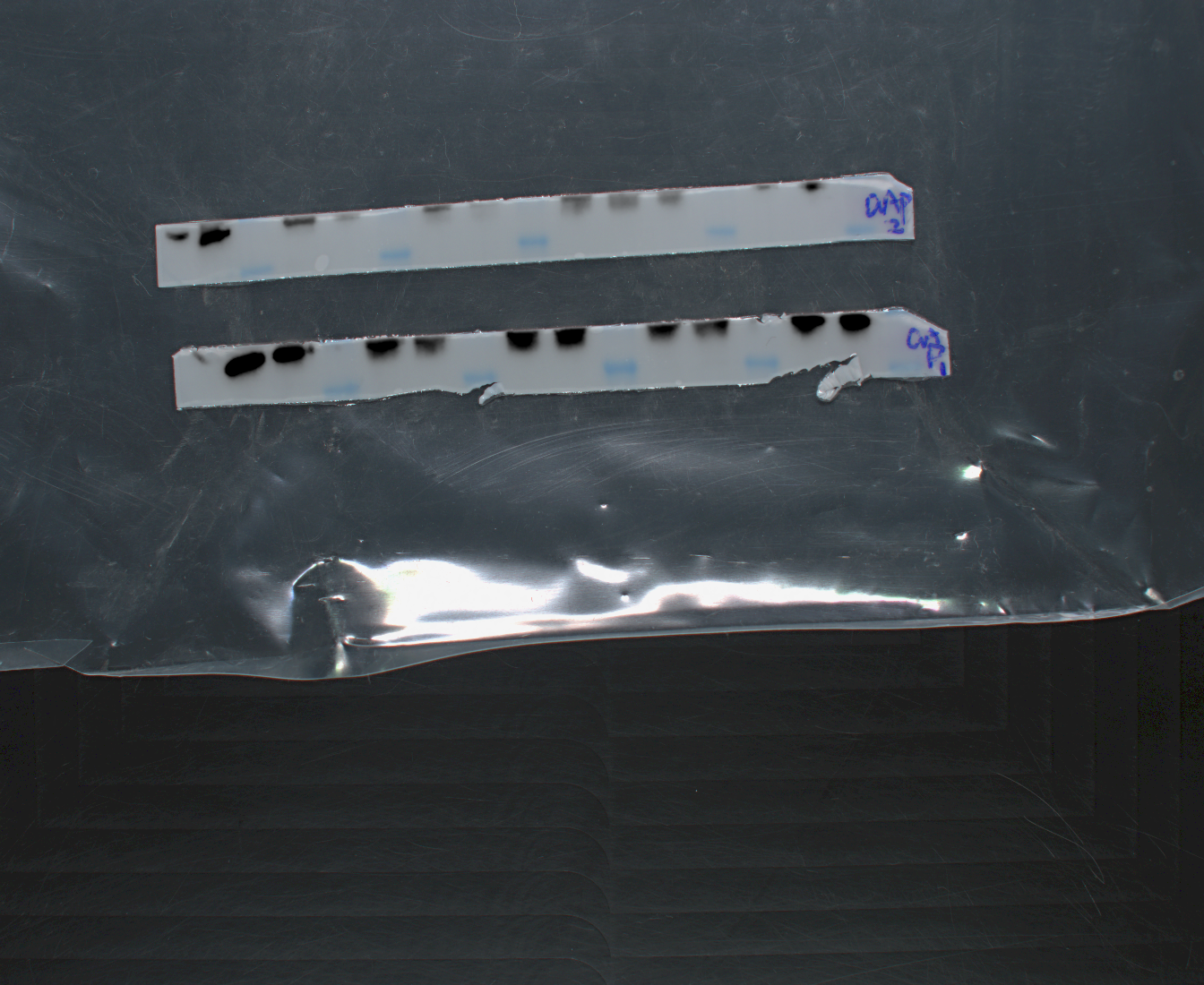

Supplement: Supplementary file 7 — original data [file 41420_2023_1589_MOESM7_ESM.zip › 5D-WB 完成/2022-11-15 GAPDH.Tif]

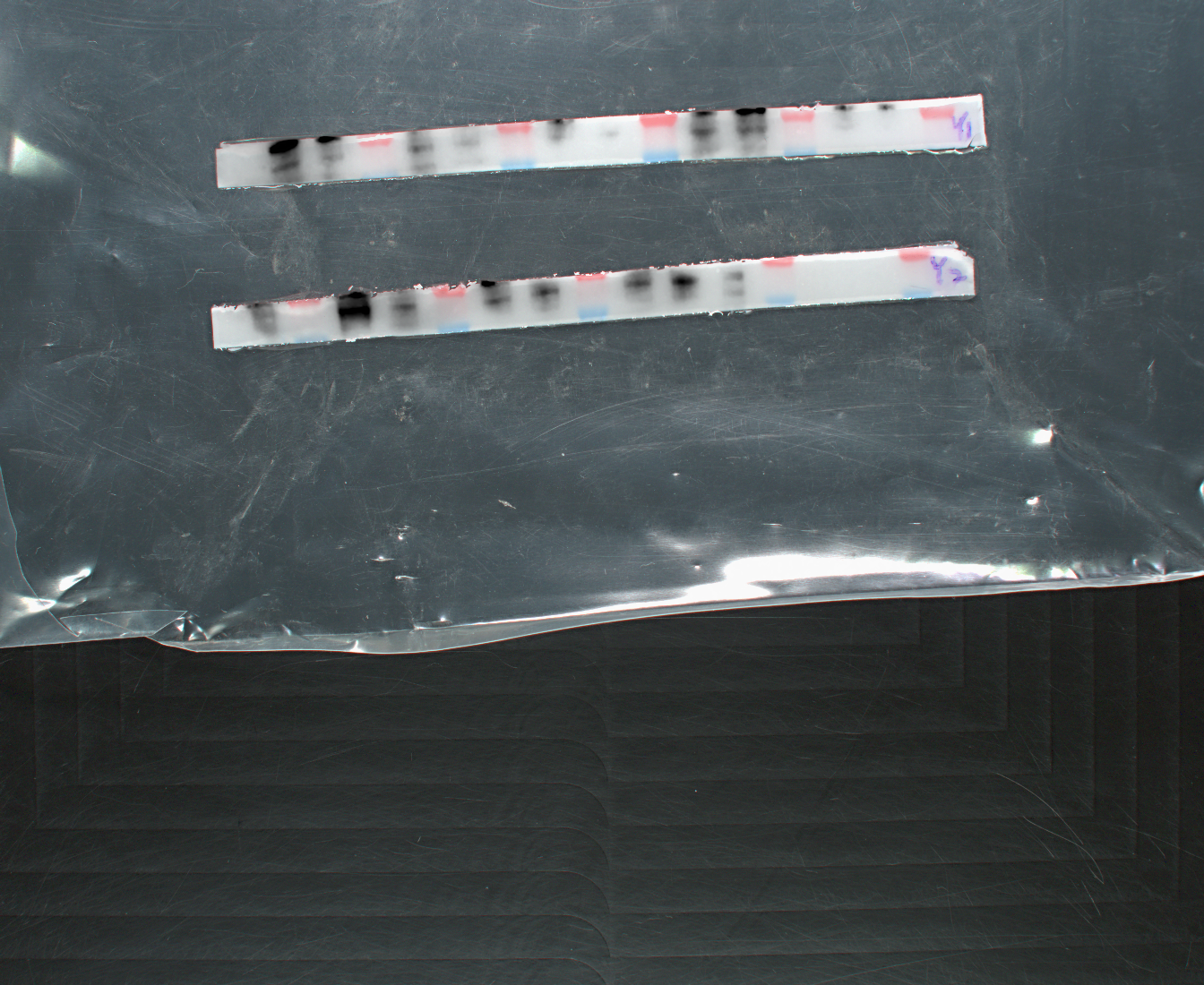

Supplement: Supplementary file 7 — original data [file 41420_2023_1589_MOESM7_ESM.zip › 5D-WB 完成/2022-11-15 YTHDF1 .Tif]

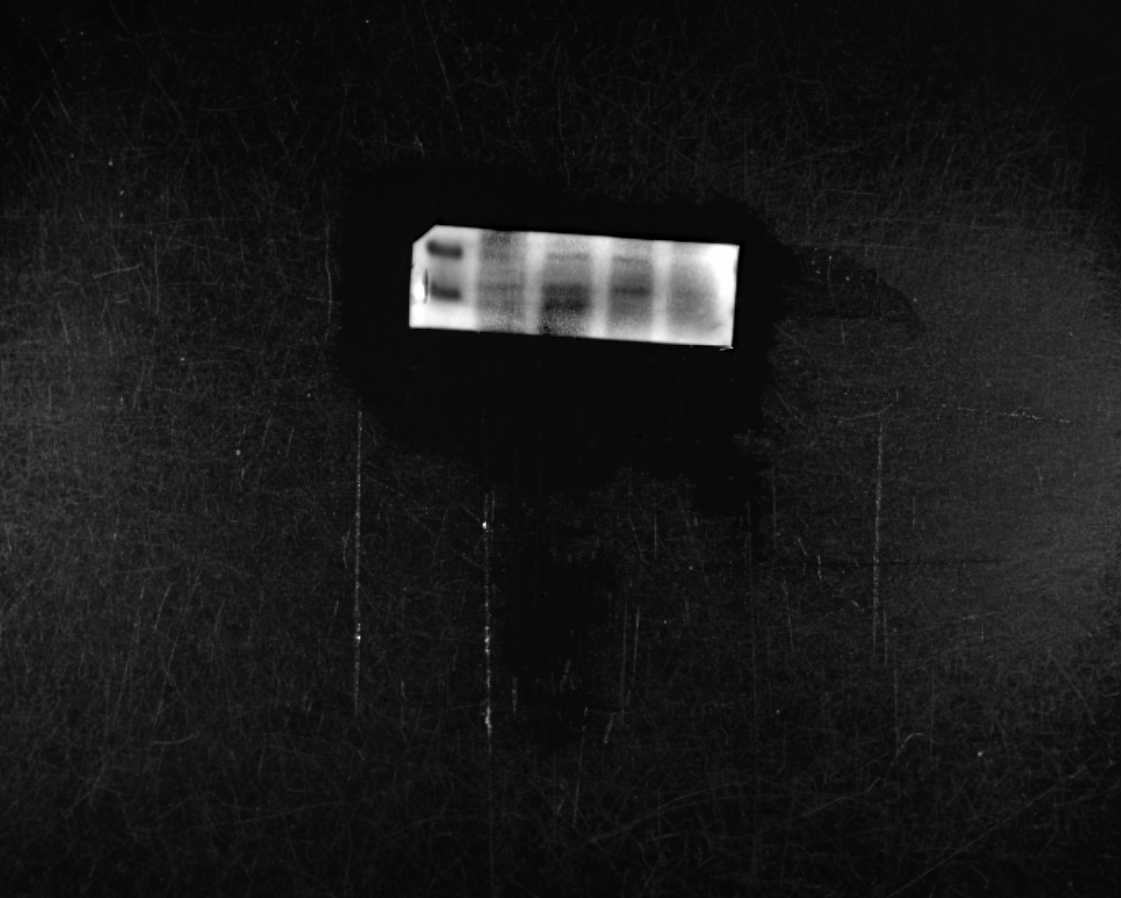

Supplement: Supplementary file 7 — original data [file 41420_2023_1589_MOESM7_ESM.zip › 4E-WB 完成/page10-1 tp53inp1 210820 cxz 15% tp53inp1_2已用.tif]

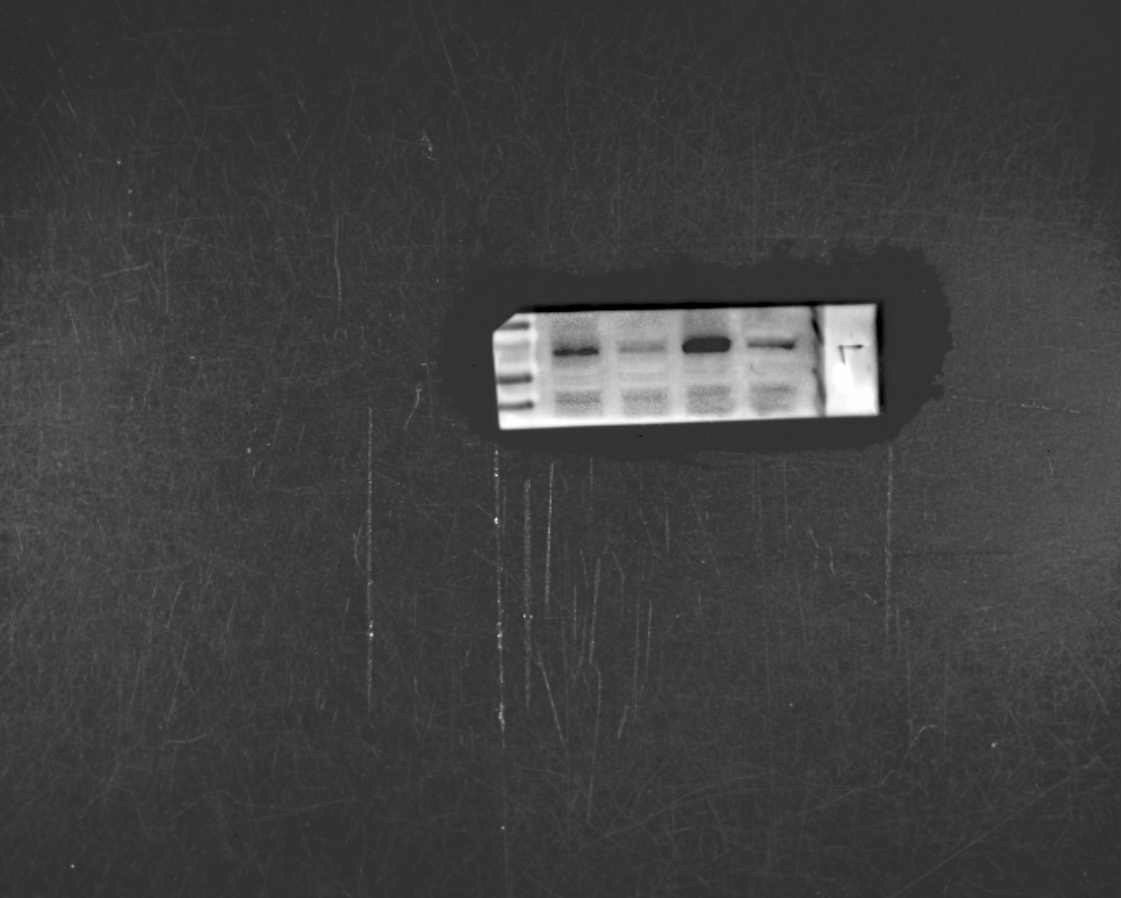

Supplement: Supplementary file 7 — original data [file 41420_2023_1589_MOESM7_ESM.zip › 4E-WB 完成/page10-2 pdcd4 210820 cxz 15% pdcd4_2已用.tif]

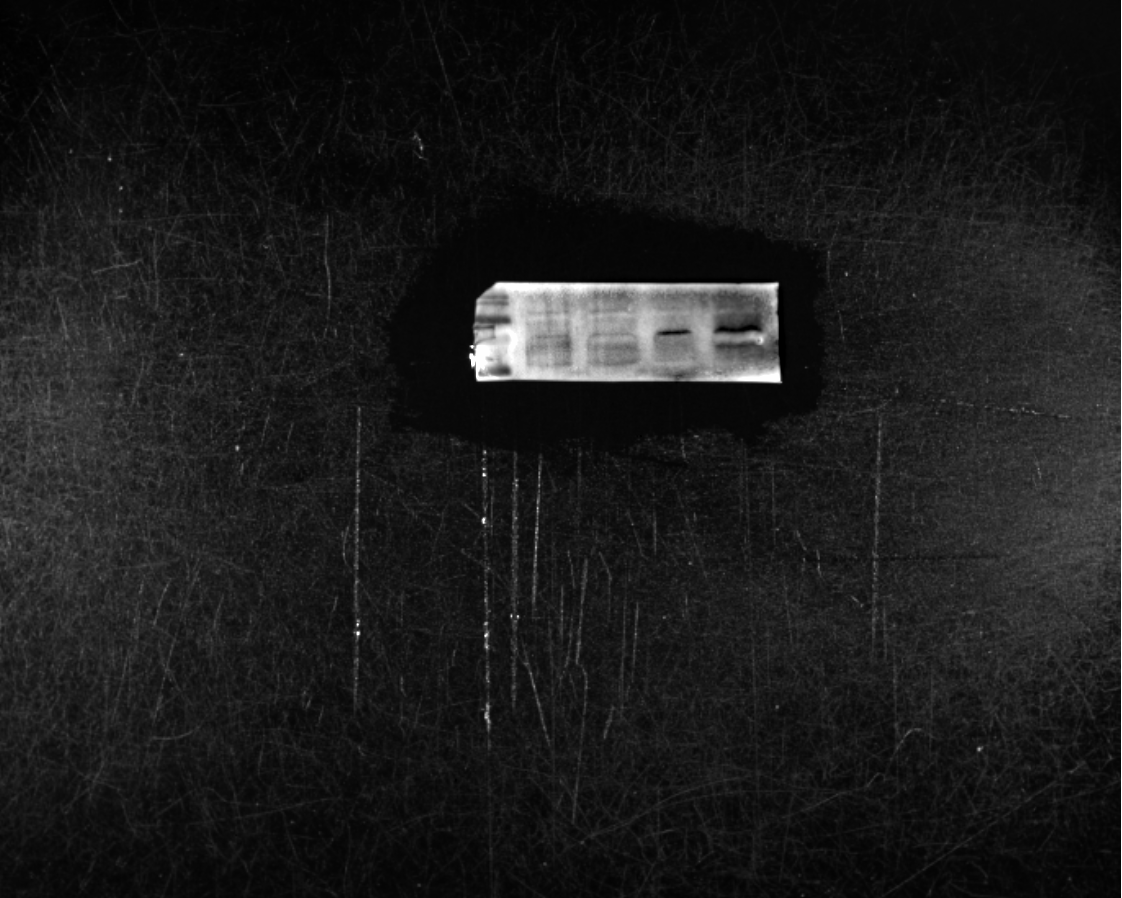

Supplement: Supplementary file 7 — original data [file 41420_2023_1589_MOESM7_ESM.zip › 4E-WB 完成/page10-3 birc3 210820 cxz 15% clap2_2已用.tif]

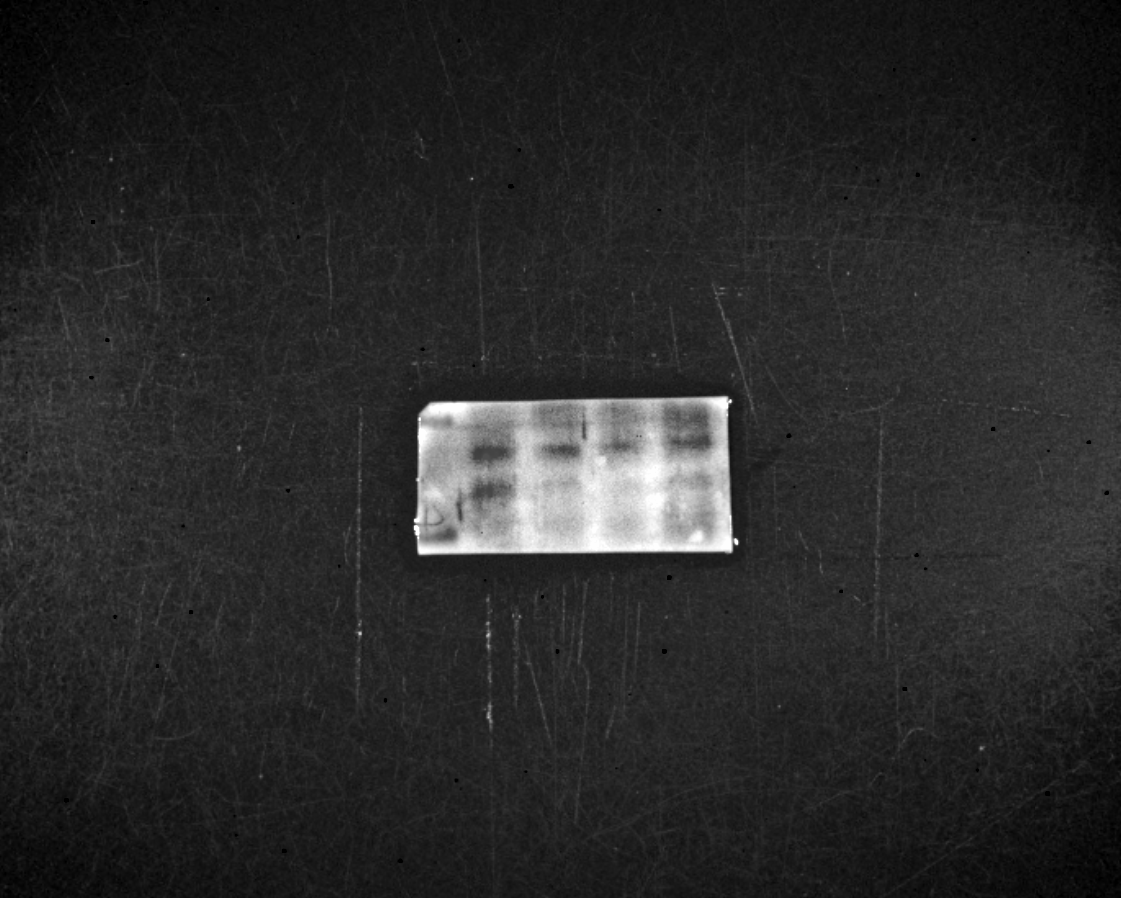

Supplement: Supplementary file 7 — original data [file 41420_2023_1589_MOESM7_ESM.zip › 4E-WB 完成/page10-4 bcl2a1 210820 cxz 15% bcl2a1_2已用.tif]

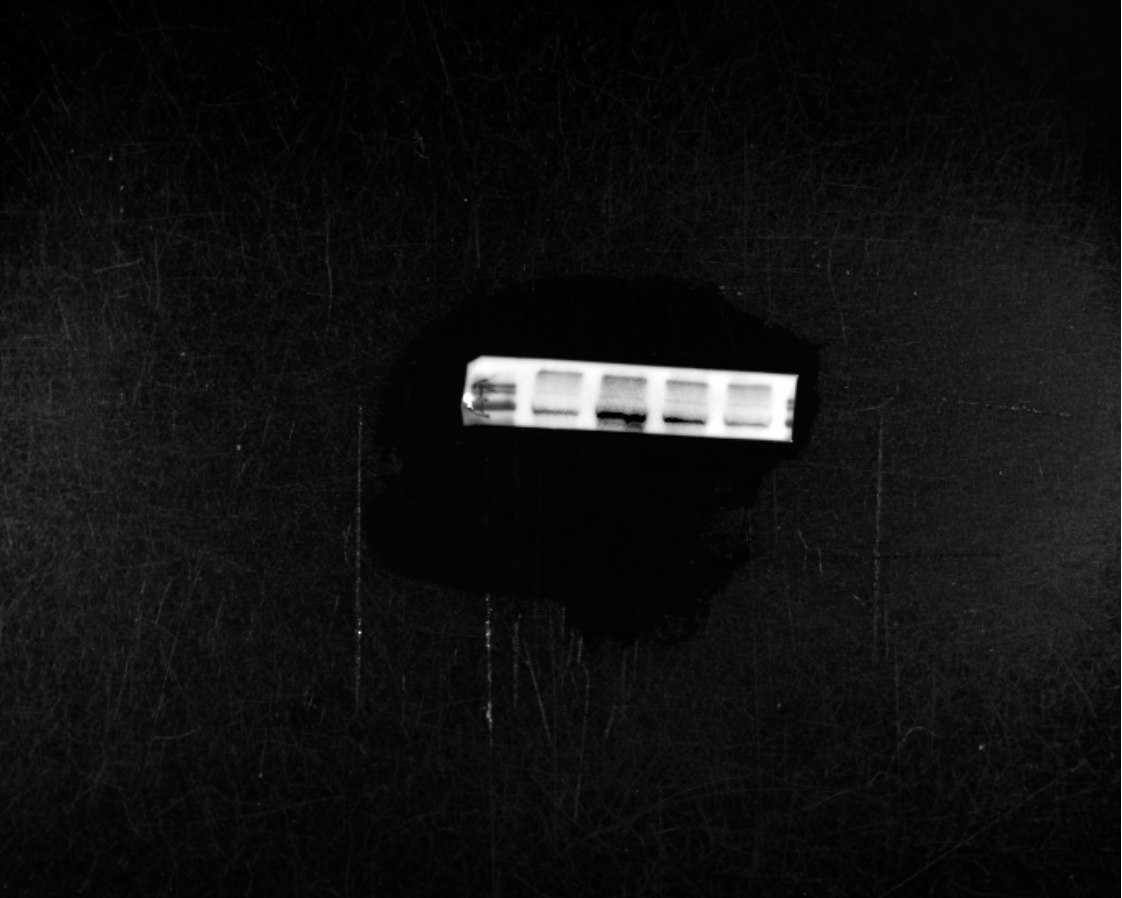

Supplement: Supplementary file 7 — original data [file 41420_2023_1589_MOESM7_ESM.zip › 4E-WB 完成/page10-5 tnfaip3 210820 cxz 15% tnfaip3_2已用.tif]

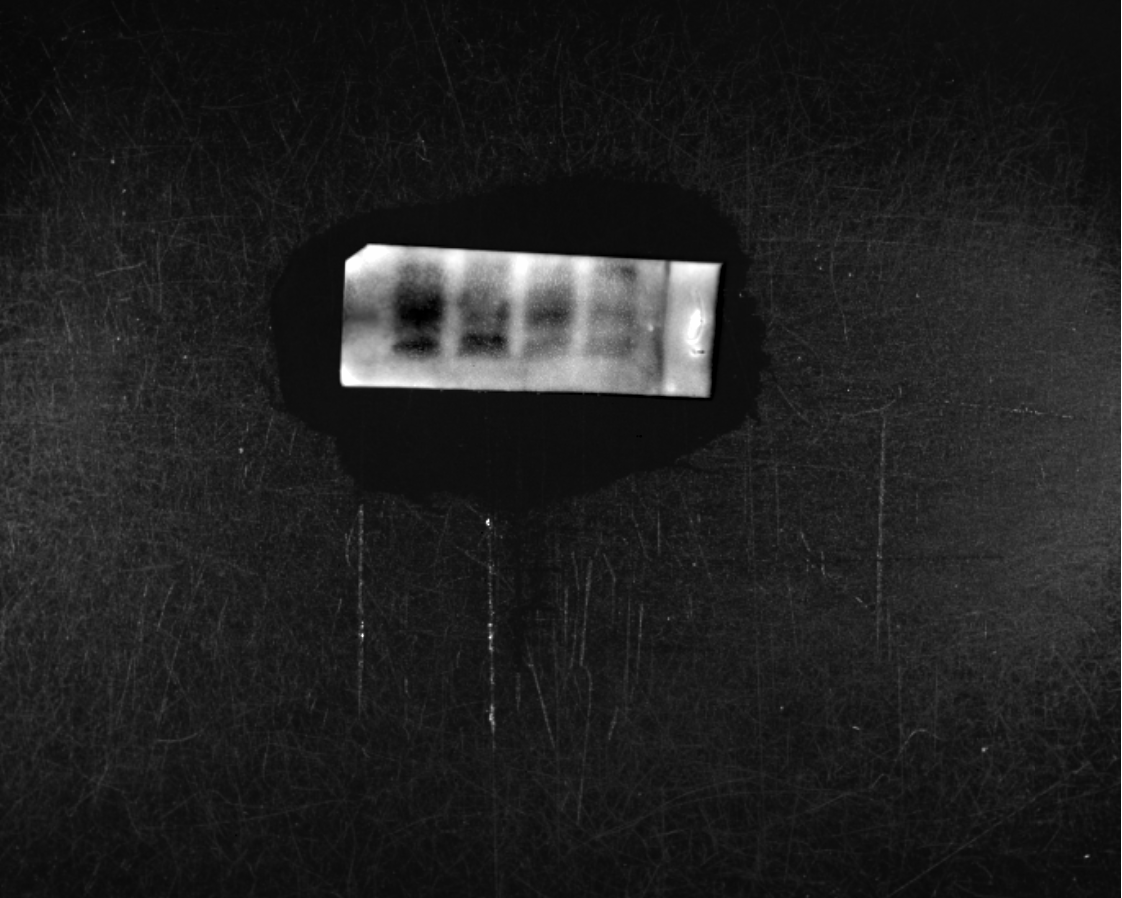

Supplement: Supplementary file 7 — original data [file 41420_2023_1589_MOESM7_ESM.zip › 4E-WB 完成/page7-1 c-cas3=10-7 210820 cxz 15% c-caspase3_2已用.tif]

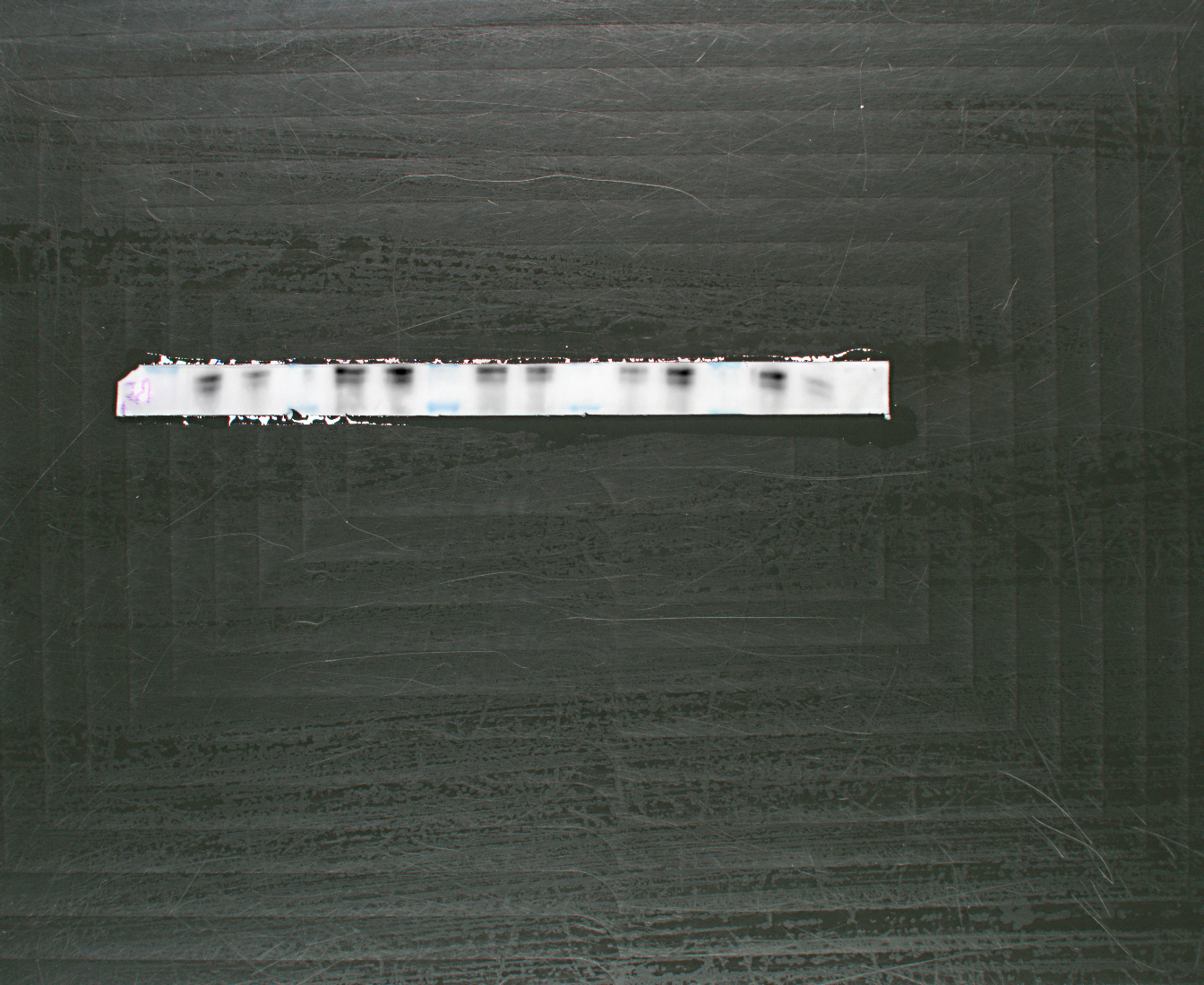

Supplement: Supplementary file 7 — original data [file 41420_2023_1589_MOESM7_ESM.zip › 2I-WB/primary submission/2022-115-15 ALKBH5 .Tif]

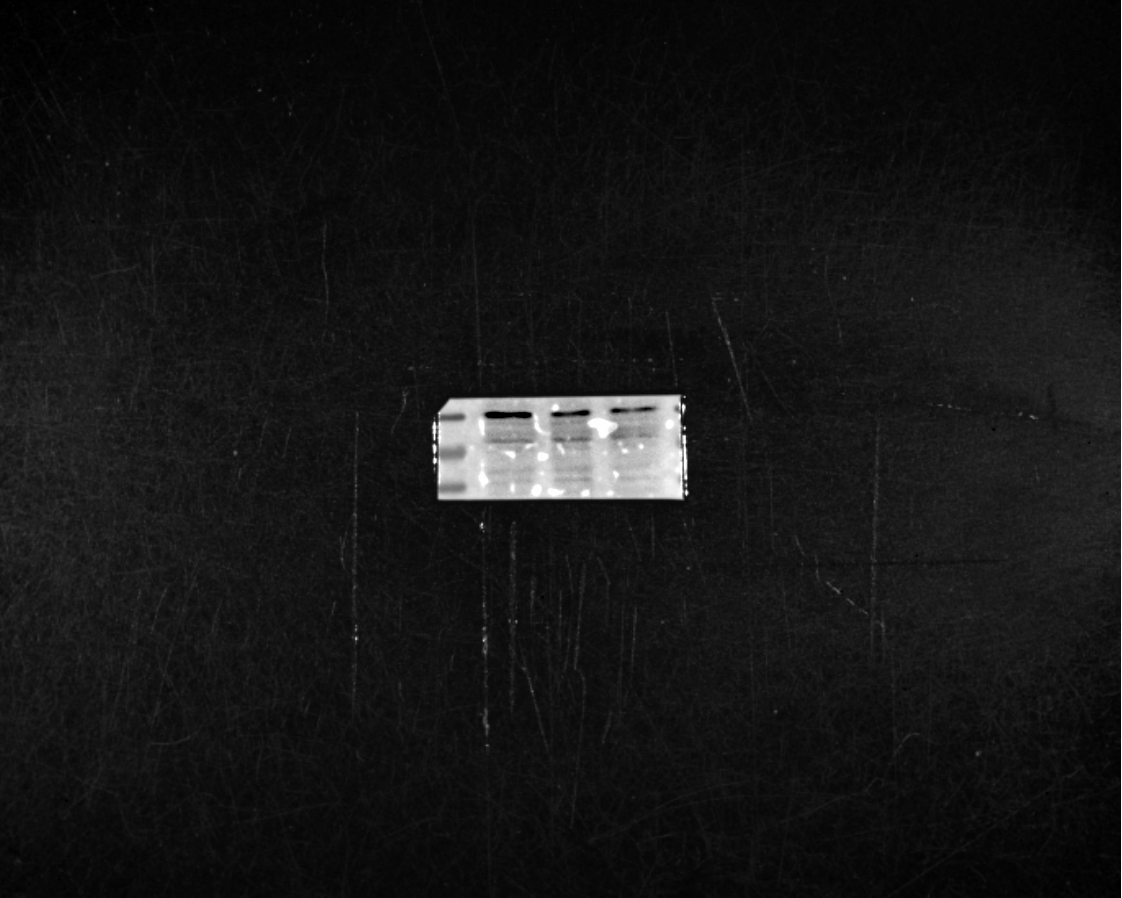

Supplement: Supplementary file 7 — original data [file 41420_2023_1589_MOESM7_ESM.zip › 2I-WB/primary submission/page3-1 mcl-1 210629 cxz 15% mcl-1_2已用.tif]

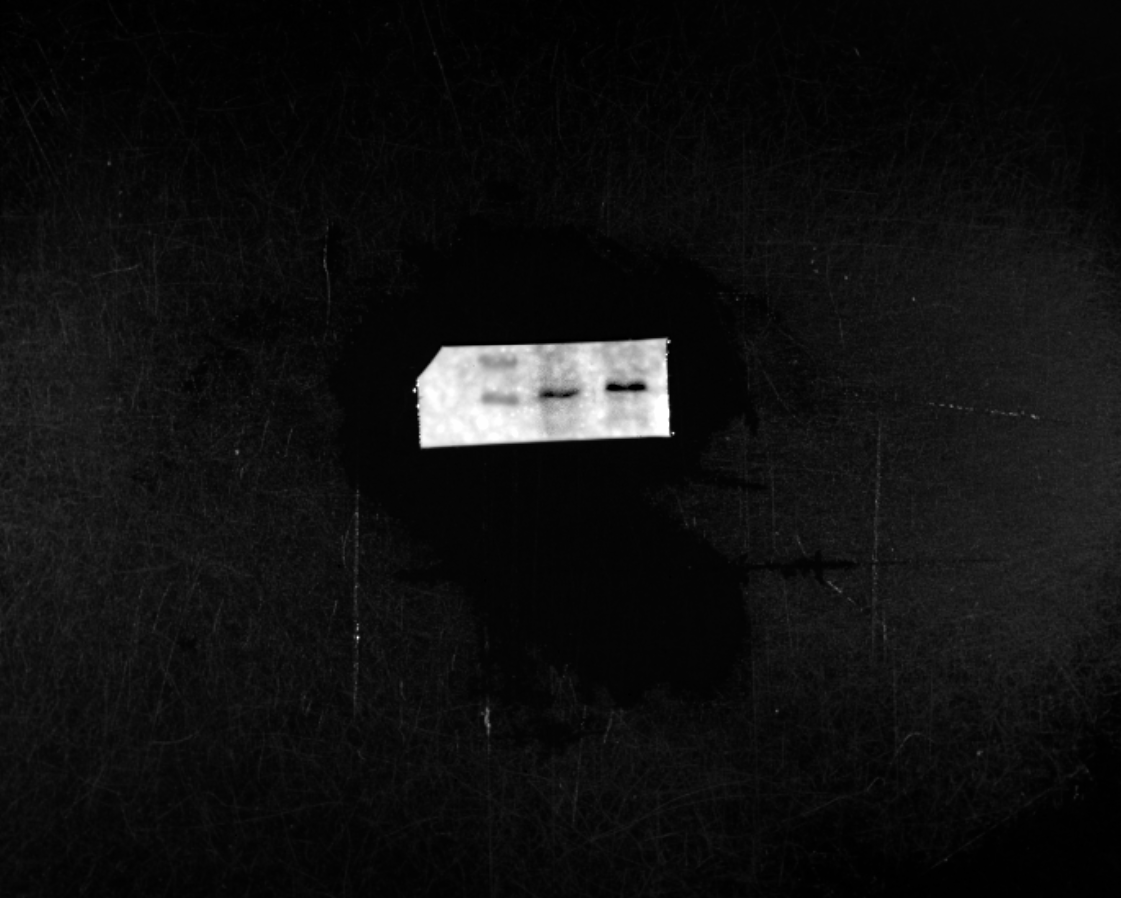

Supplement: Supplementary file 7 — original data [file 41420_2023_1589_MOESM7_ESM.zip › 2I-WB/primary submission/page3-2 bcl-2 210701 cxz 15% bcl2_2已用.tif]

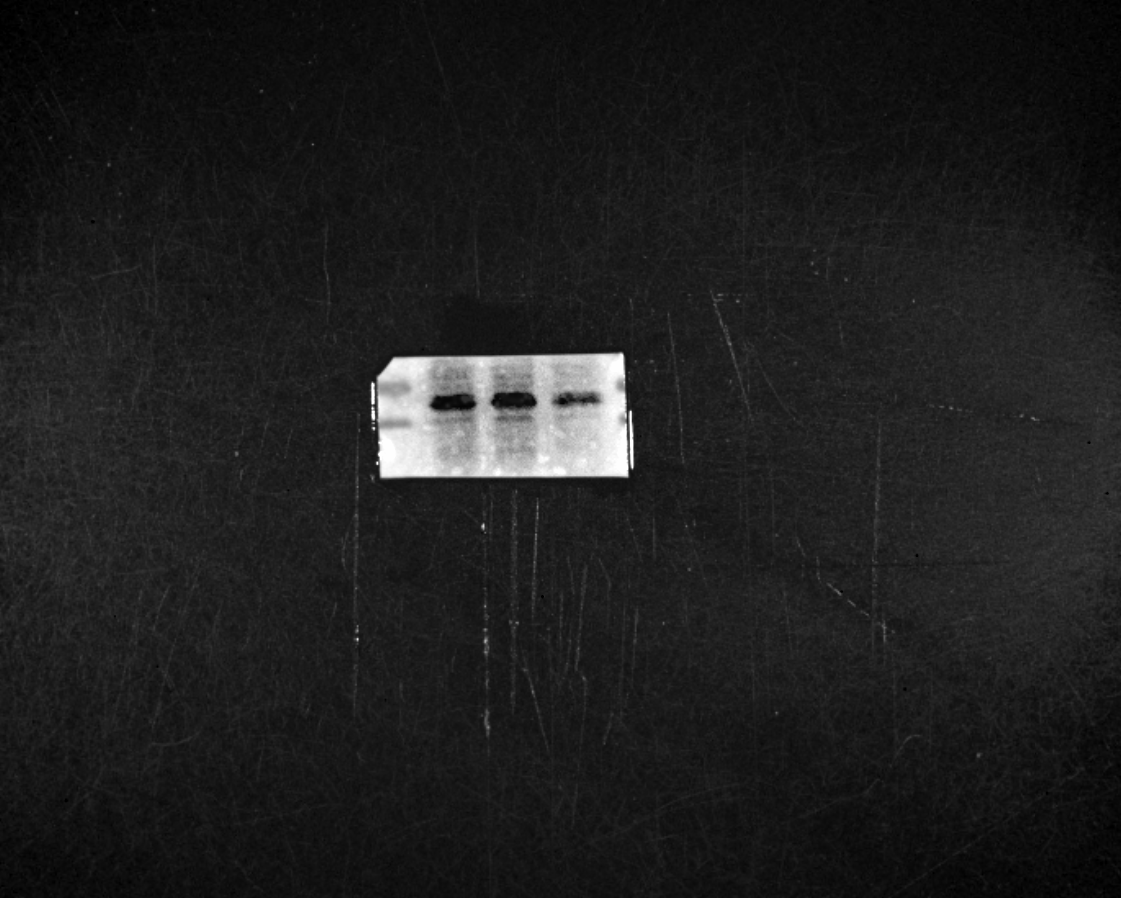

Supplement: Supplementary file 7 — original data [file 41420_2023_1589_MOESM7_ESM.zip › 2I-WB/primary submission/page3-3 bcl-xl 210629 cxz 15% bcl-xl_2已用.tif]

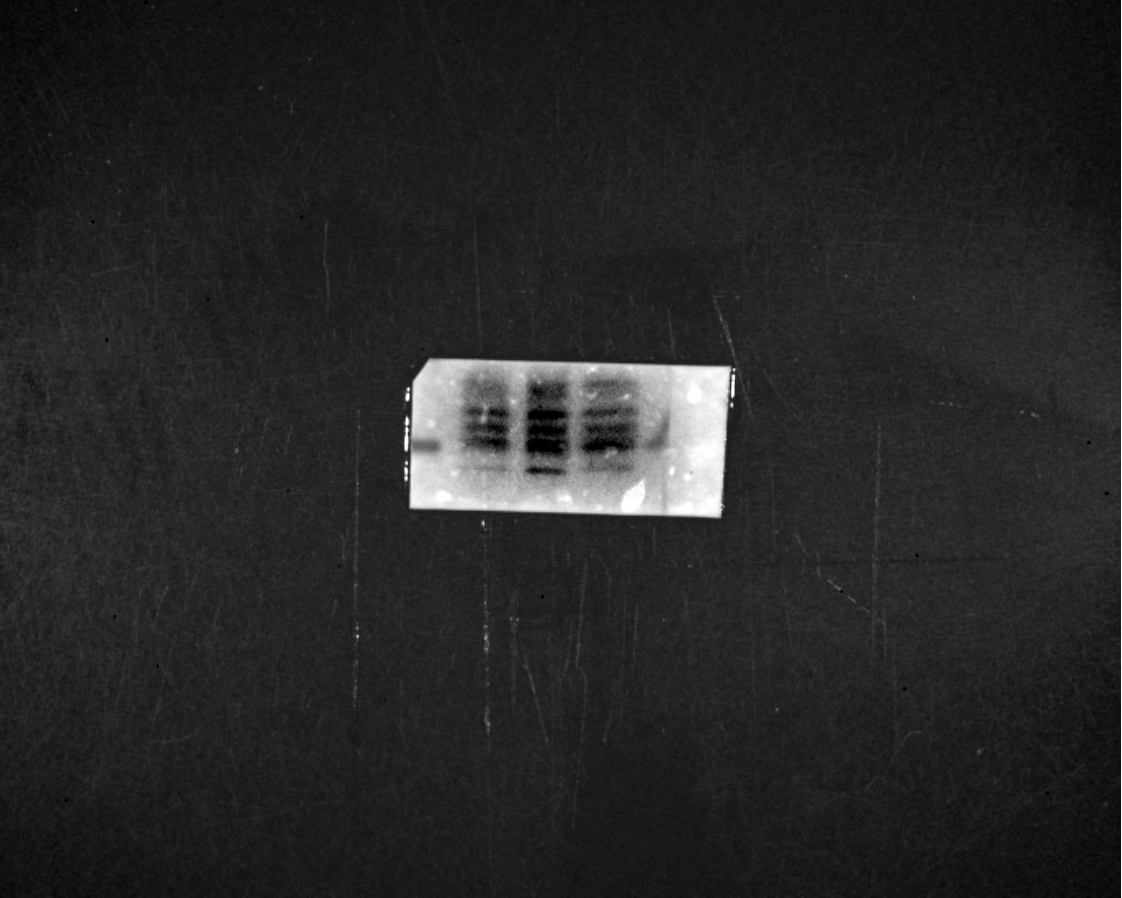

Supplement: Supplementary file 7 — original data [file 41420_2023_1589_MOESM7_ESM.zip › 2I-WB/primary submission/page3-4 c-cas3 210629 cxz 15% c-casp3_2已用.tif]

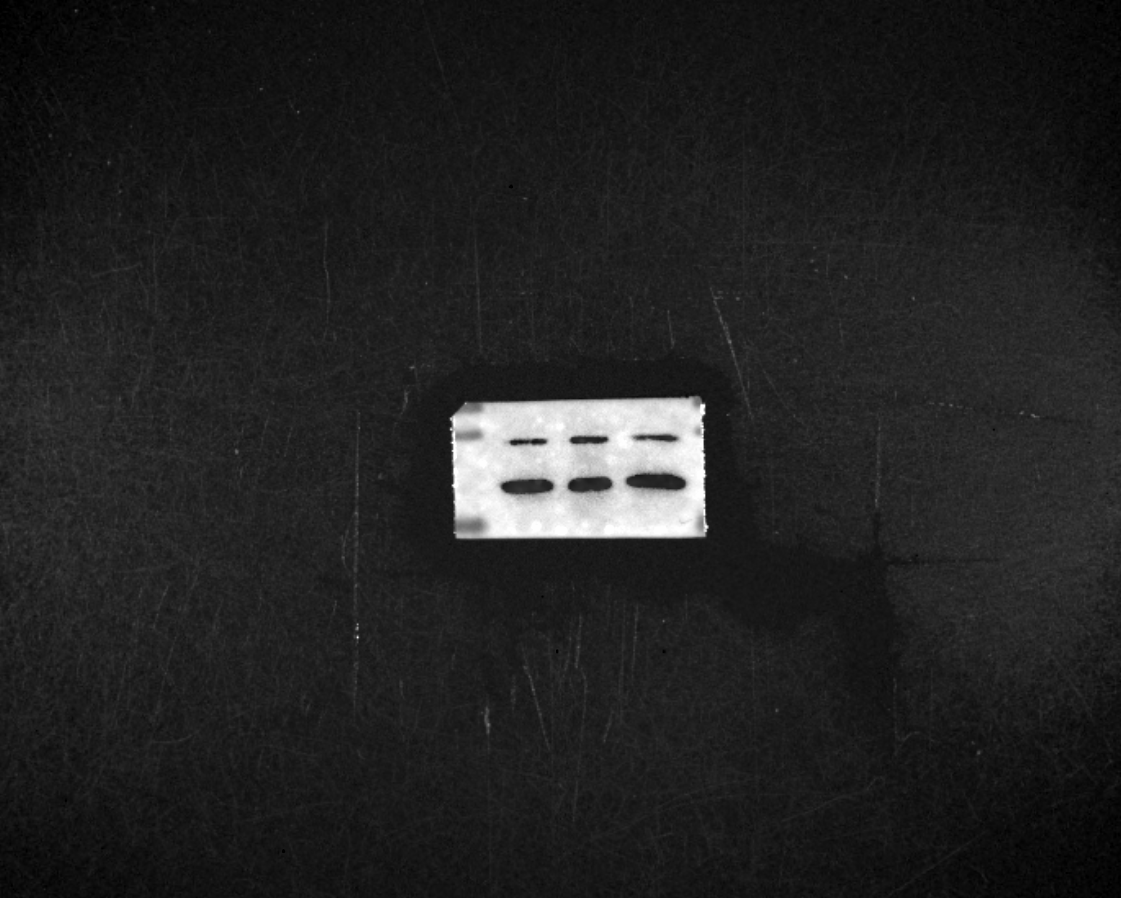

Supplement: Supplementary file 7 — original data [file 41420_2023_1589_MOESM7_ESM.zip › 2I-WB/primary submission/page3-6 puma 210629 cxz 15% puma_2已用.tif]

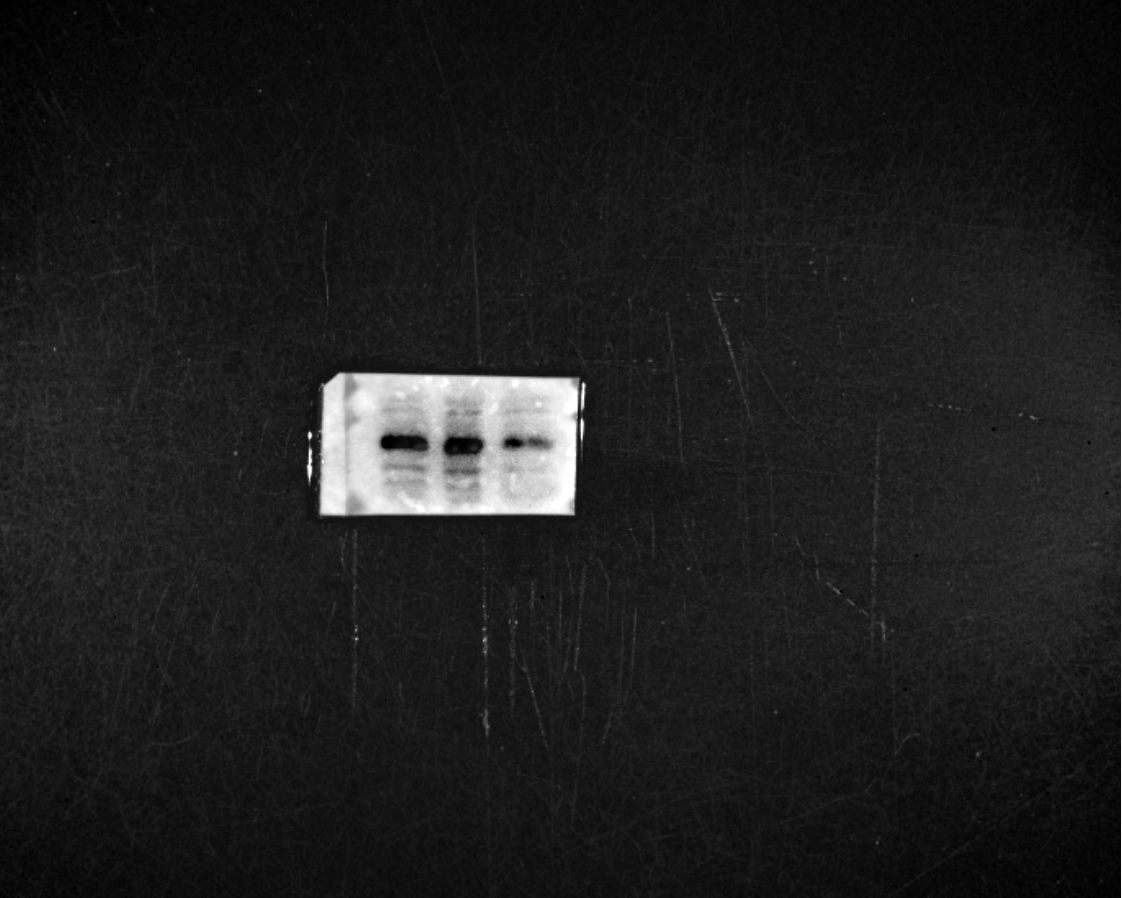

Supplement: Supplementary file 7 — original data [file 41420_2023_1589_MOESM7_ESM.zip › 2I-WB/primary submission/page3-7 bad 210629 cxz 15% bad_2已用.tif]

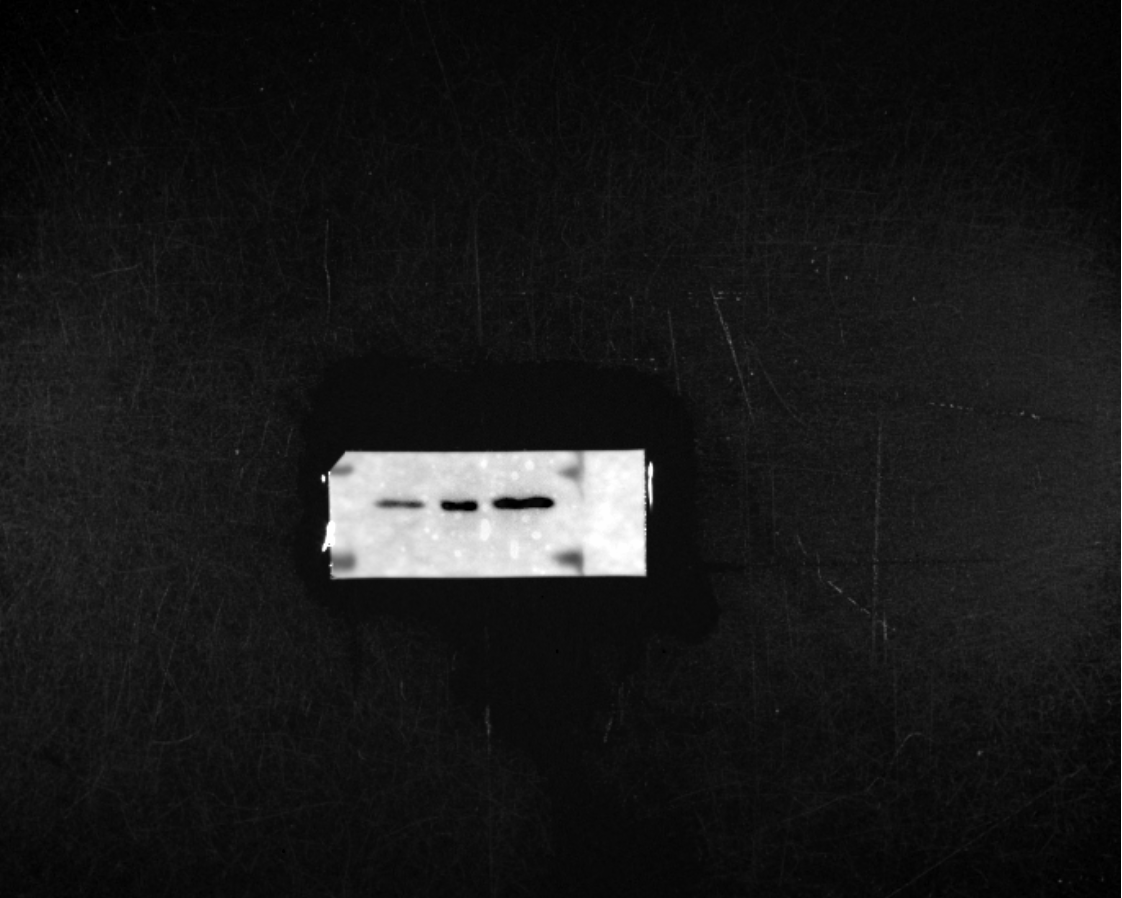

Supplement: Supplementary file 7 — original data [file 41420_2023_1589_MOESM7_ESM.zip › 2I-WB/primary submission/page3-8 bax 210629 cxz 15% bax_2已用.tif]

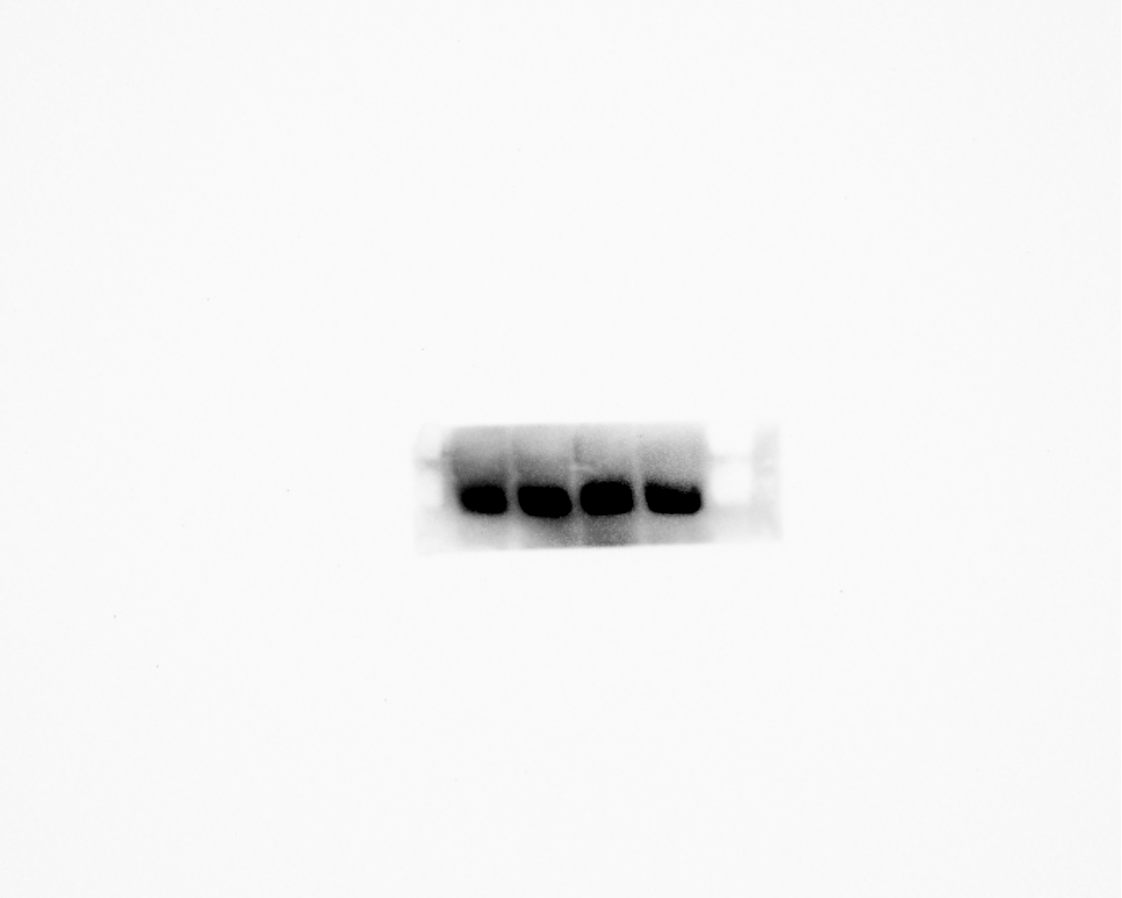

Supplement: Supplementary file 7 — original data [file 41420_2023_1589_MOESM7_ESM.zip › 2I-WB/primary submission/page3-9 tubulin =page4-8 没有merge 210730 cxz 10% tubulin_1已用.tif]

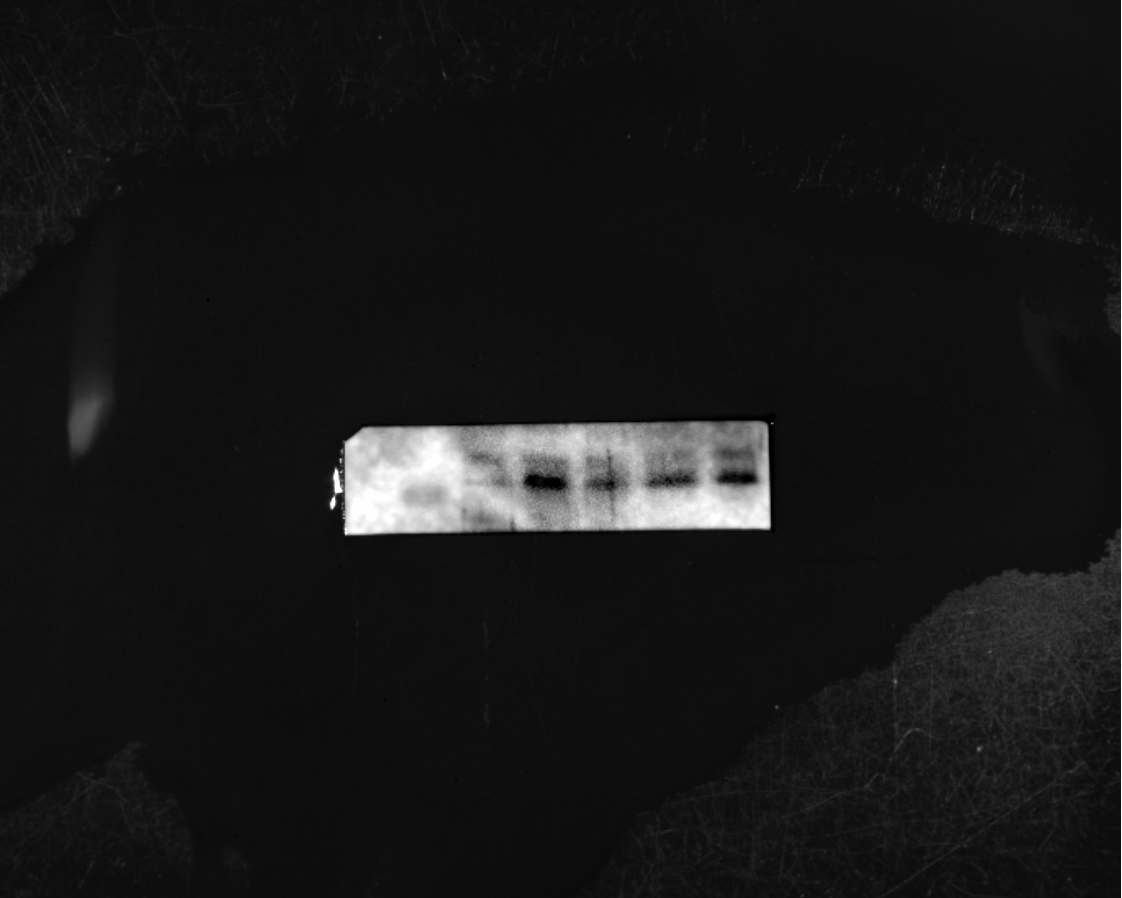

Supplement: Supplementary file 7 — original data [file 41420_2023_1589_MOESM7_ESM.zip › 2I-WB/revision/page2-3左 bcl-2 21064 cxz 12% bcl2_2已用.tif]

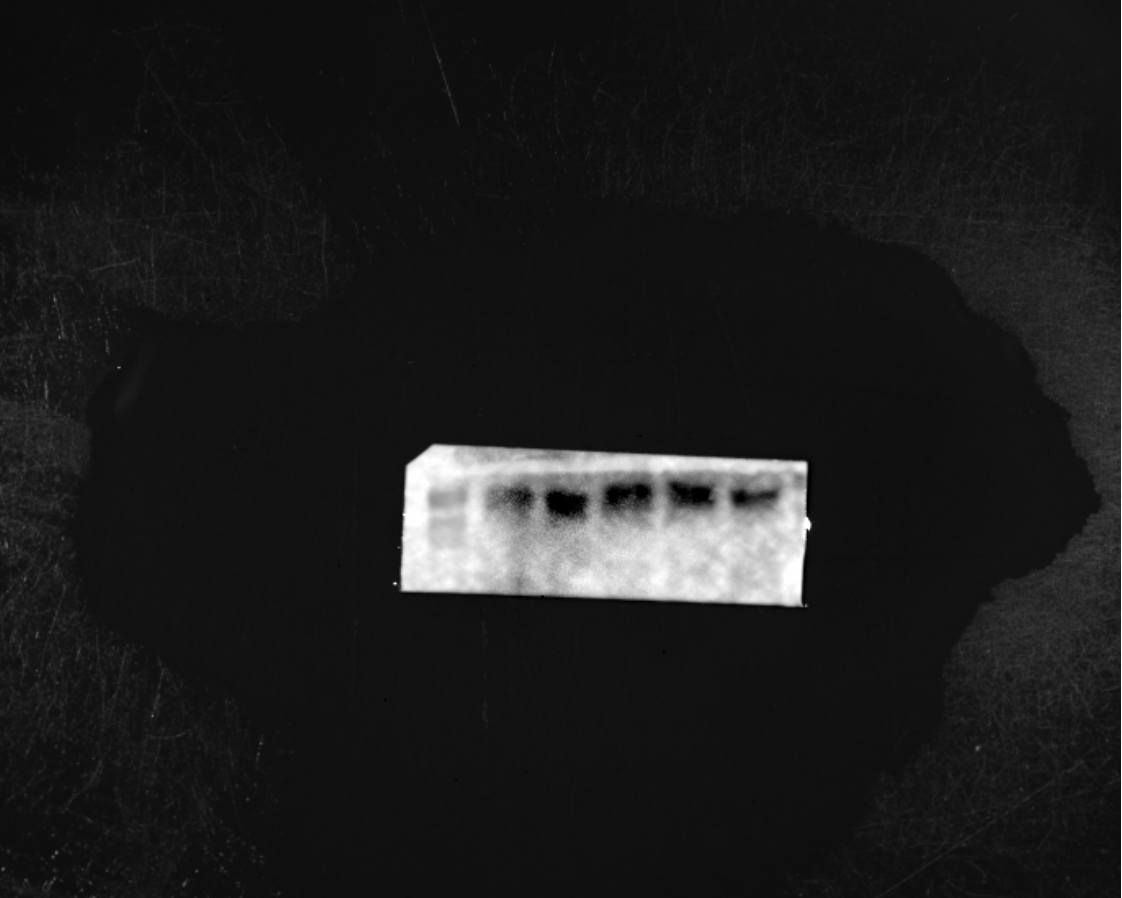

Supplement: Supplementary file 7 — original data [file 41420_2023_1589_MOESM7_ESM.zip › 2I-WB/revision/page2-4左 bcl-xl 210625 cxz 15% bcl-xl_2已用.tif]

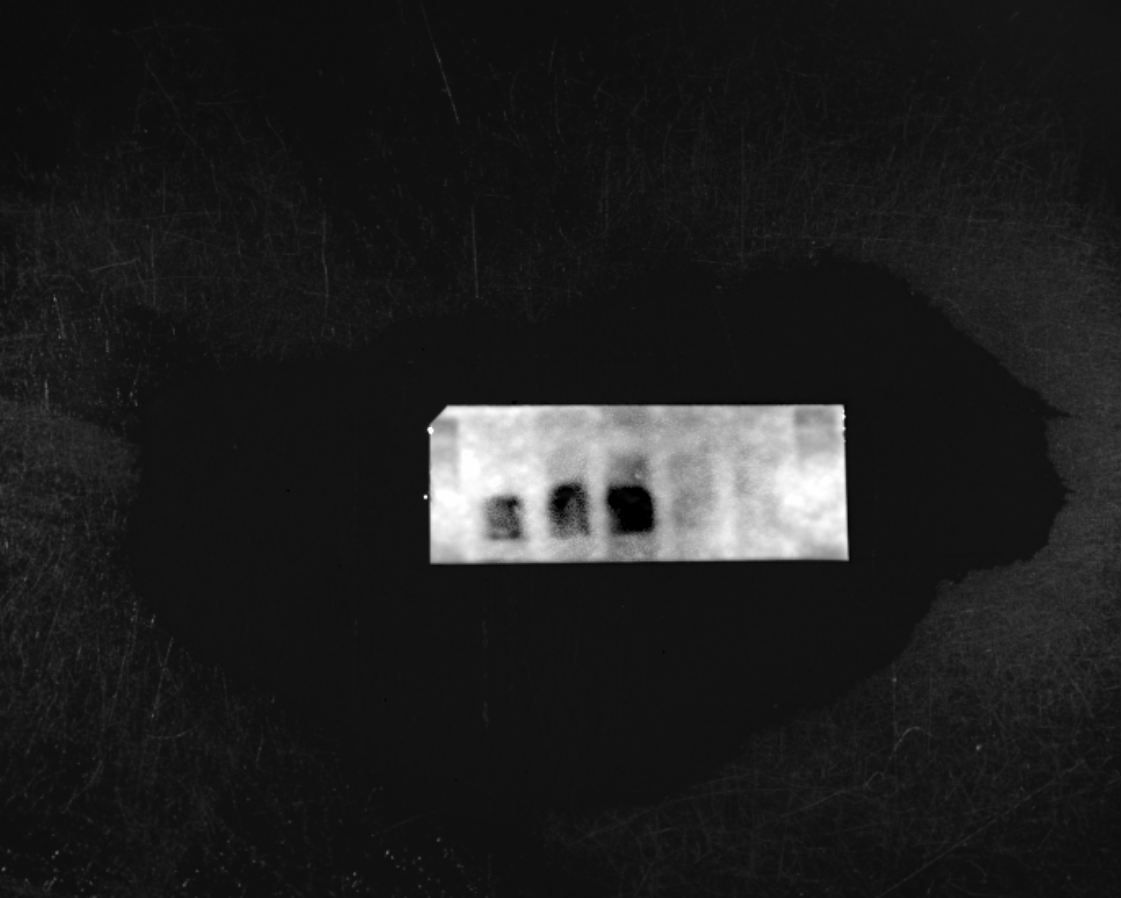

Supplement: Supplementary file 7 — original data [file 41420_2023_1589_MOESM7_ESM.zip › 2I-WB/revision/page2-5左 puma 210625 cxz 15% puma_2已用.tif]

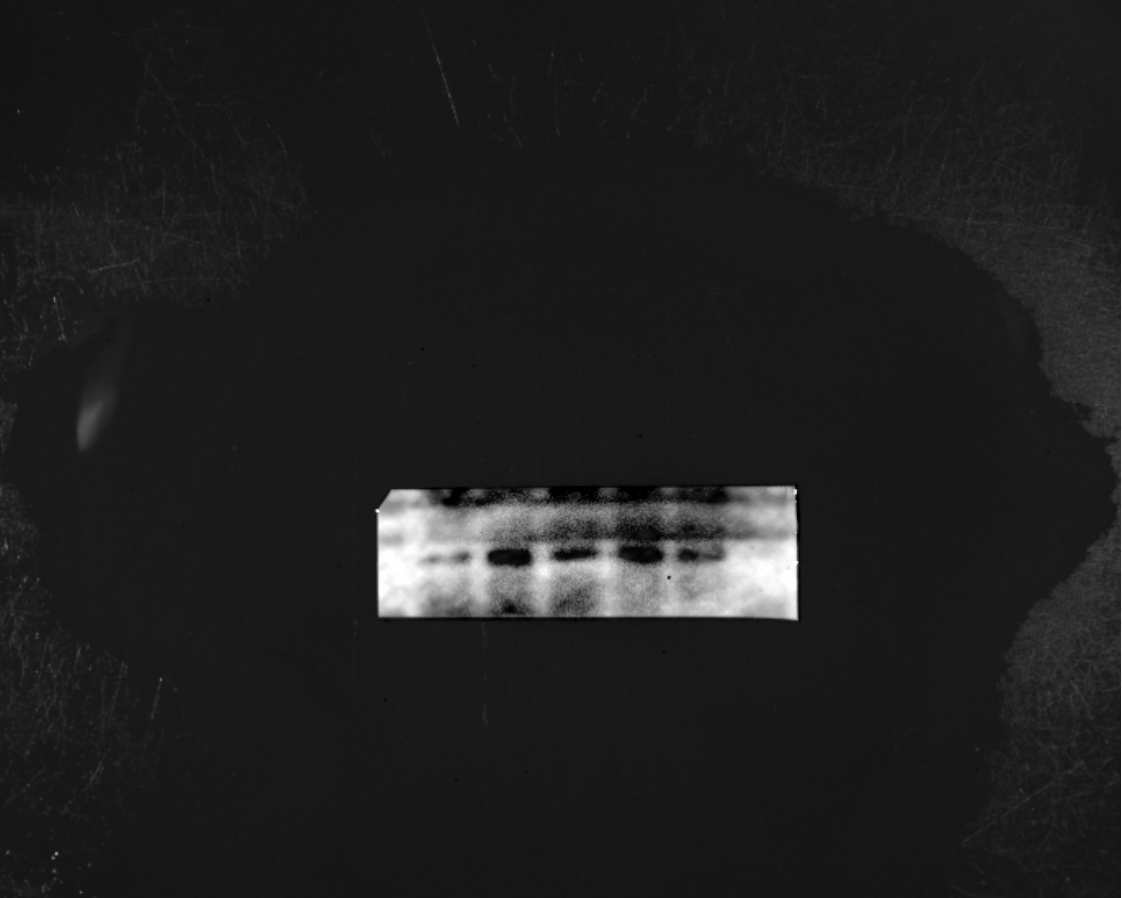

Supplement: Supplementary file 7 — original data [file 41420_2023_1589_MOESM7_ESM.zip › 2I-WB/revision/page2-6左 bad 210625 cxz 15% bad_2已用.tif]

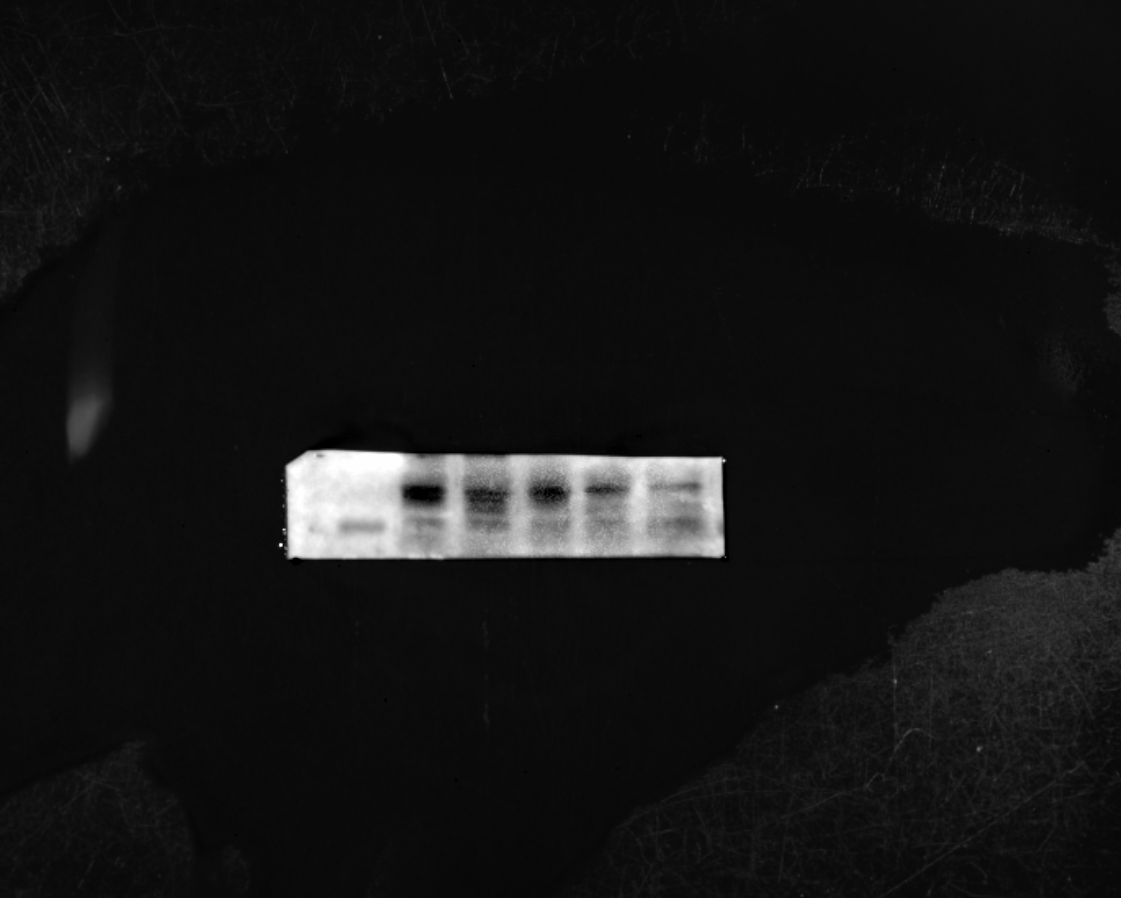

Supplement: Supplementary file 7 — original data [file 41420_2023_1589_MOESM7_ESM.zip › 2I-WB/revision/page2-7左 mcl-1 210624 cxz 12% mcl1_2已用.tif]

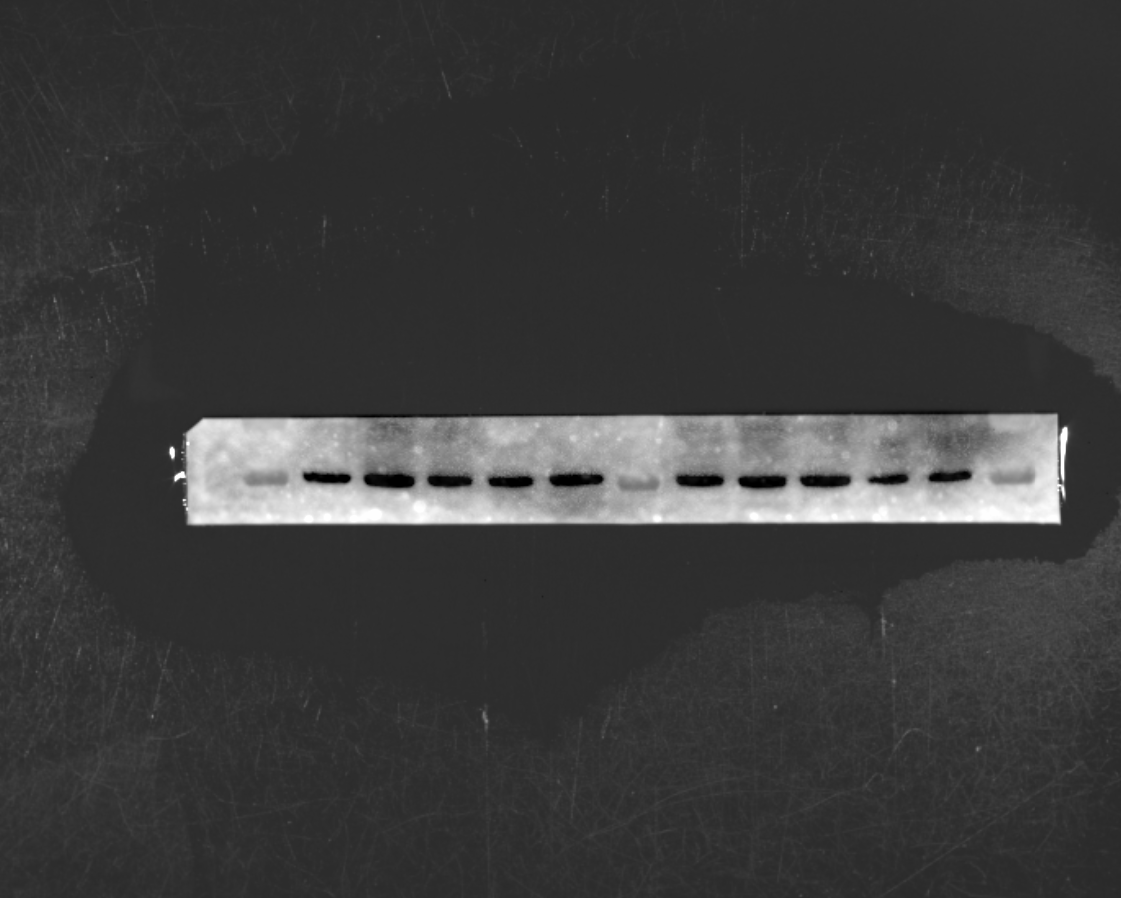

Supplement: Supplementary file 7 — original data [file 41420_2023_1589_MOESM7_ESM.zip › 2I-WB/revision/page2-8左 tubulin 210624 cxz 12% tubulin_2已用.tif]

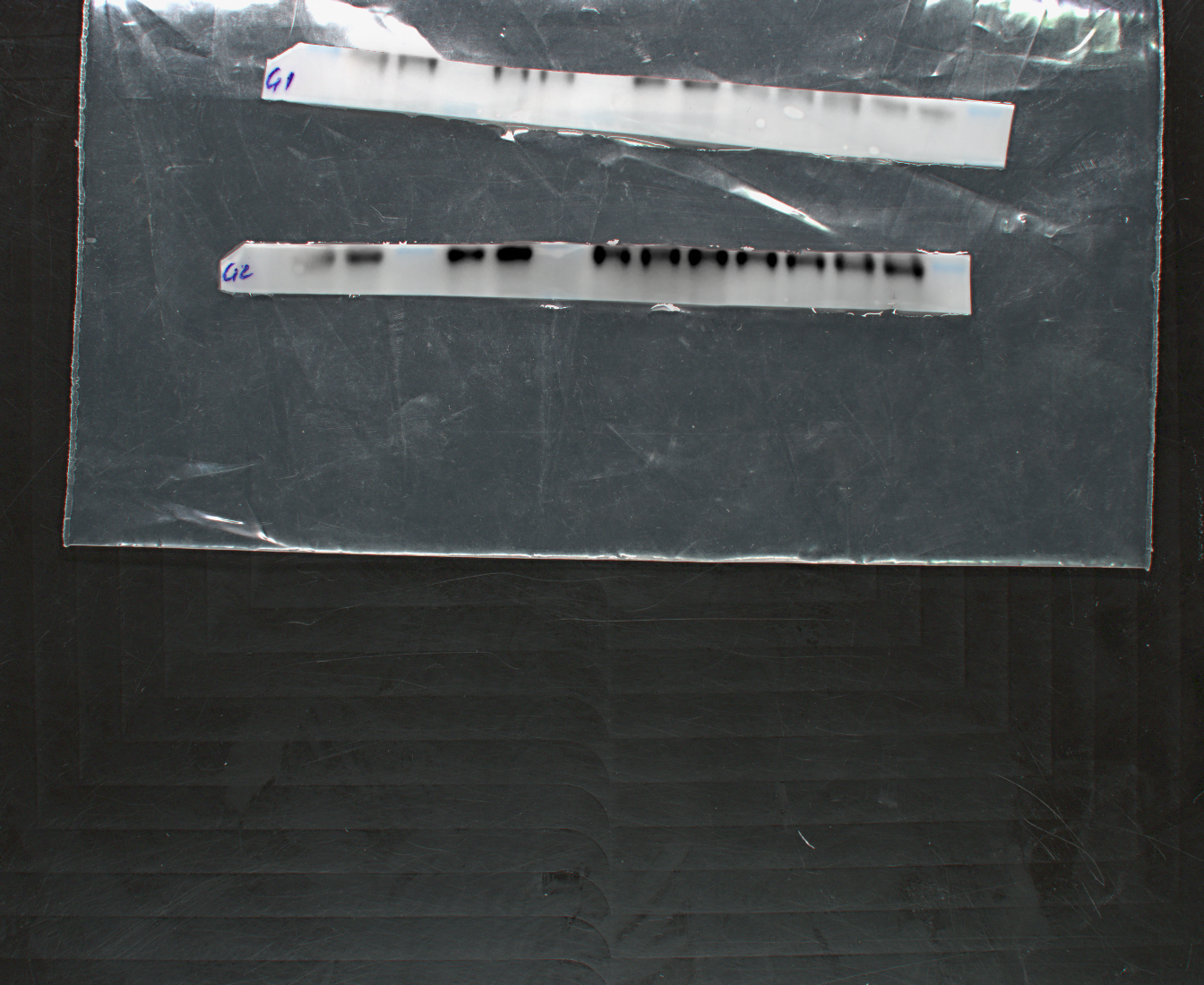

Supplement: Supplementary file 7 — original data [file 41420_2023_1589_MOESM7_ESM.zip › 3G-WB/GAP-2与K5OE对应 (1).Tif]

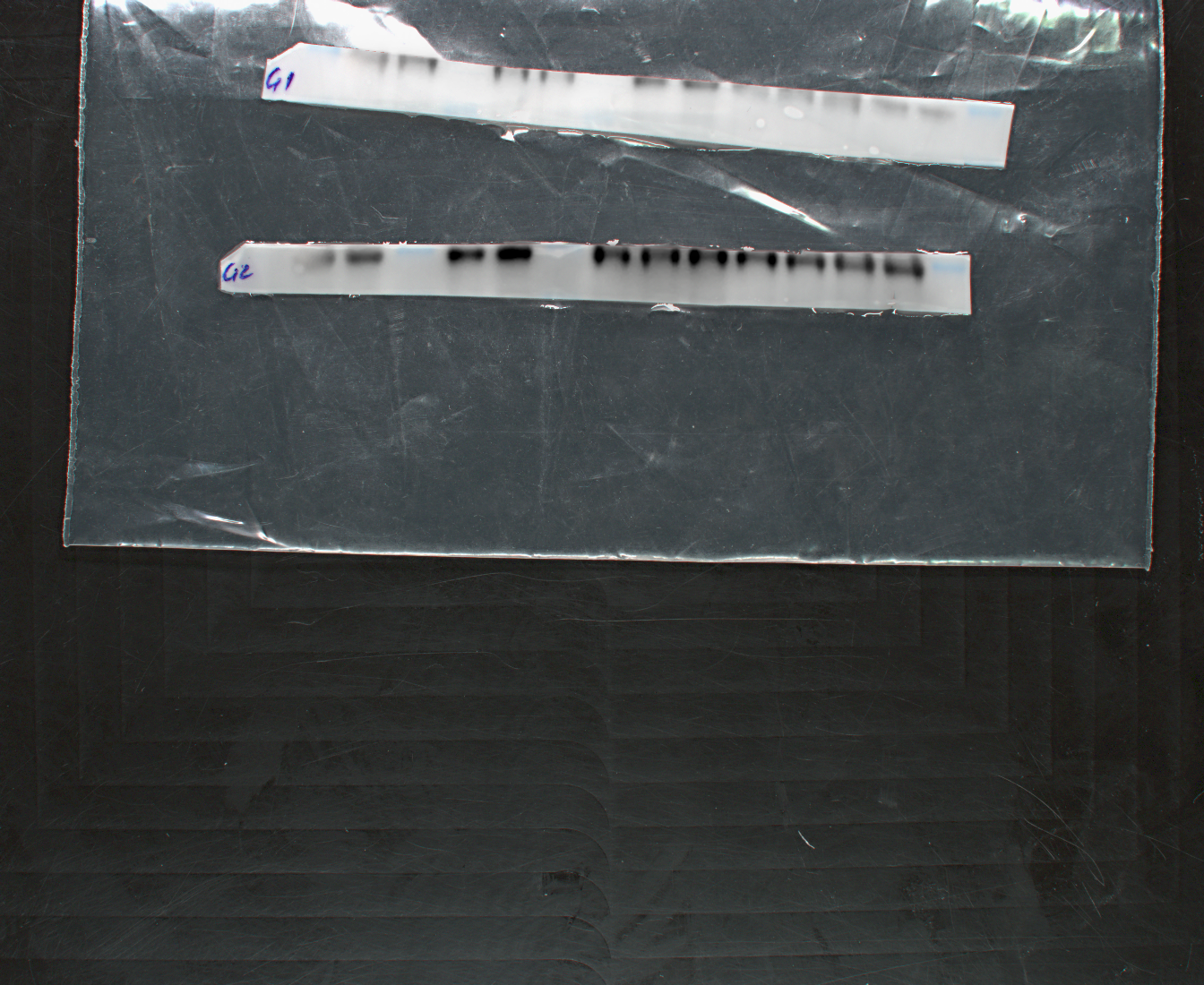

Supplement: Supplementary file 7 — original data [file 41420_2023_1589_MOESM7_ESM.zip › 3G-WB/GAP-2与K5OE对应 (2).Tif]

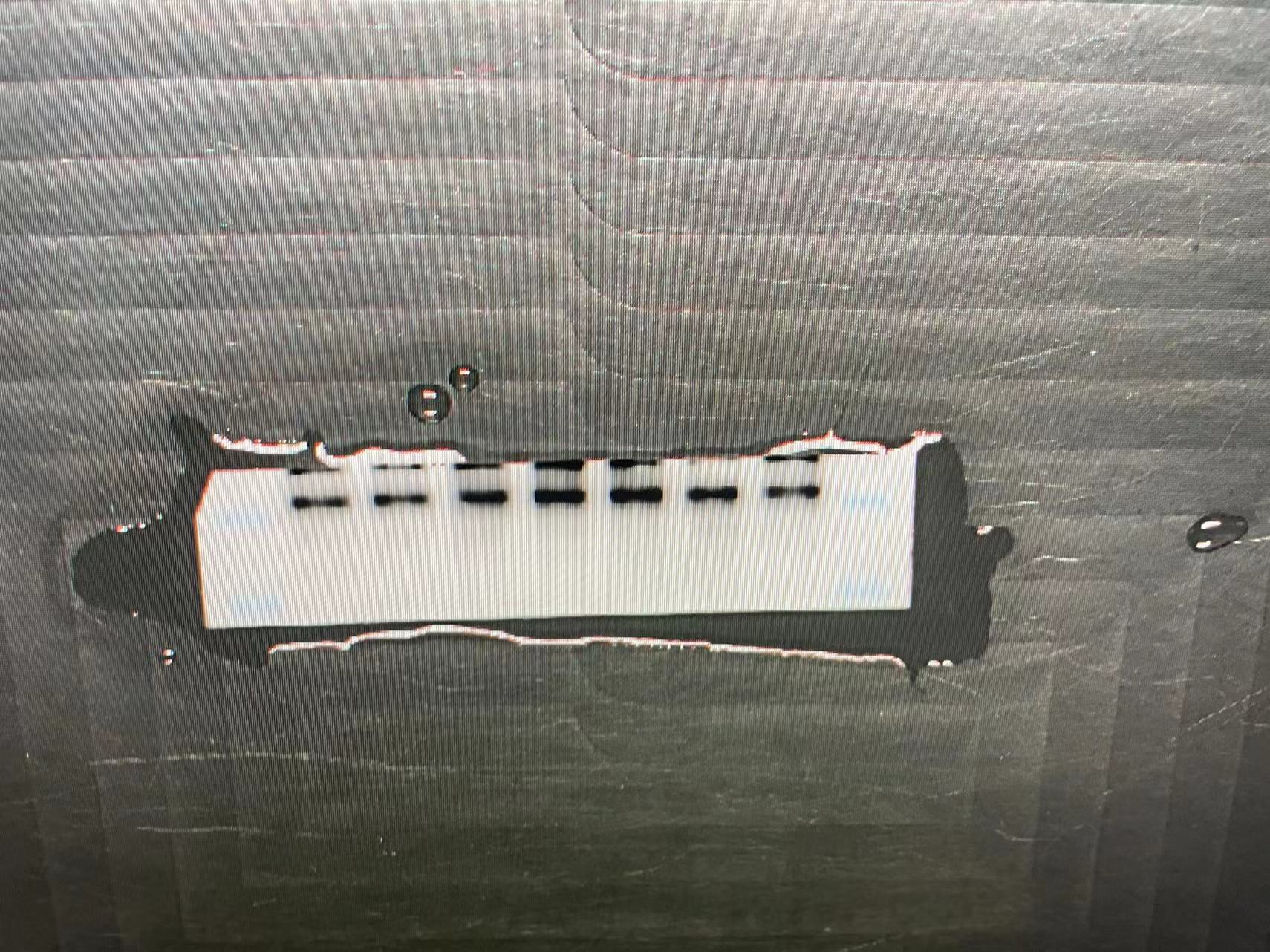

Supplement: Supplementary file 7 — original data [file 41420_2023_1589_MOESM7_ESM.zip › 3G-WB/GAPDH -与k5OE对应.jpg]

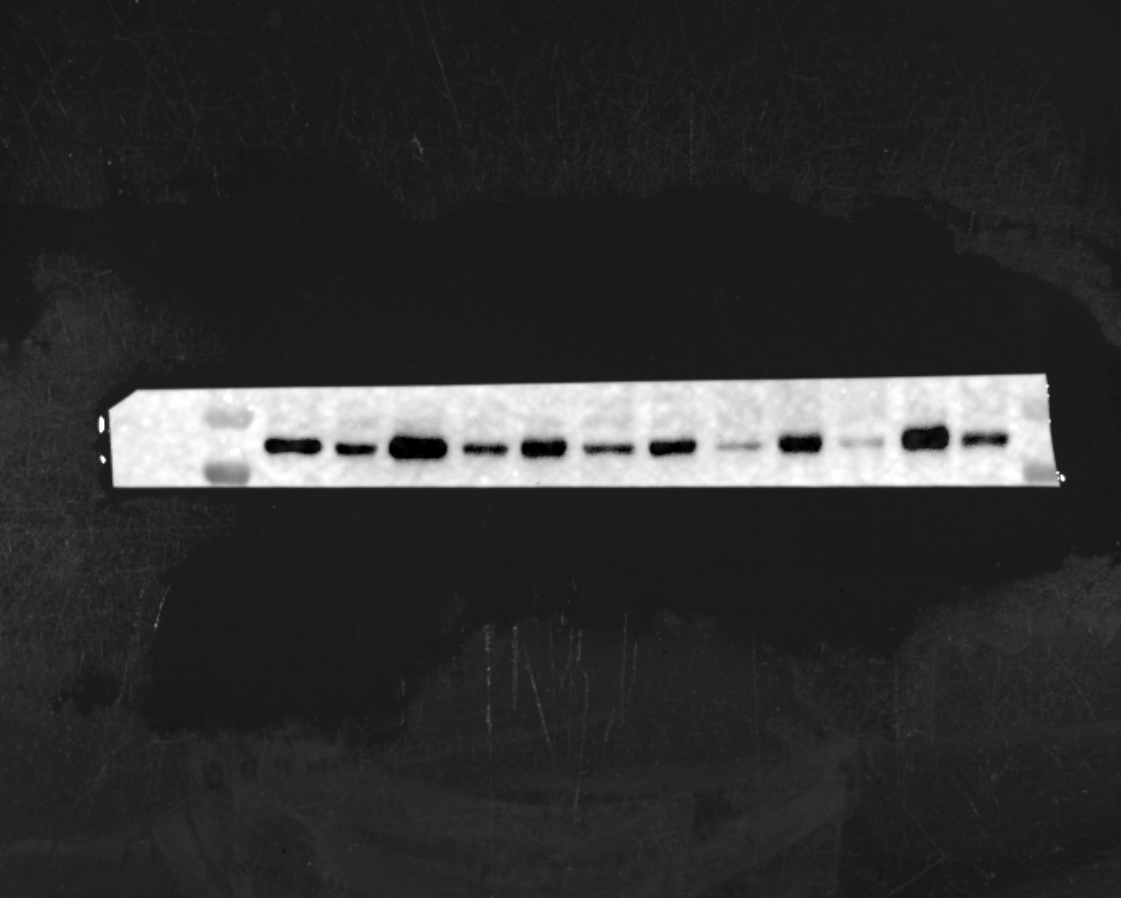

Supplement: Supplementary file 7 — original data [file 41420_2023_1589_MOESM7_ESM.zip › 3G-WB/page8-1=9-1 tlr2 211216 cxz 10% .tif]

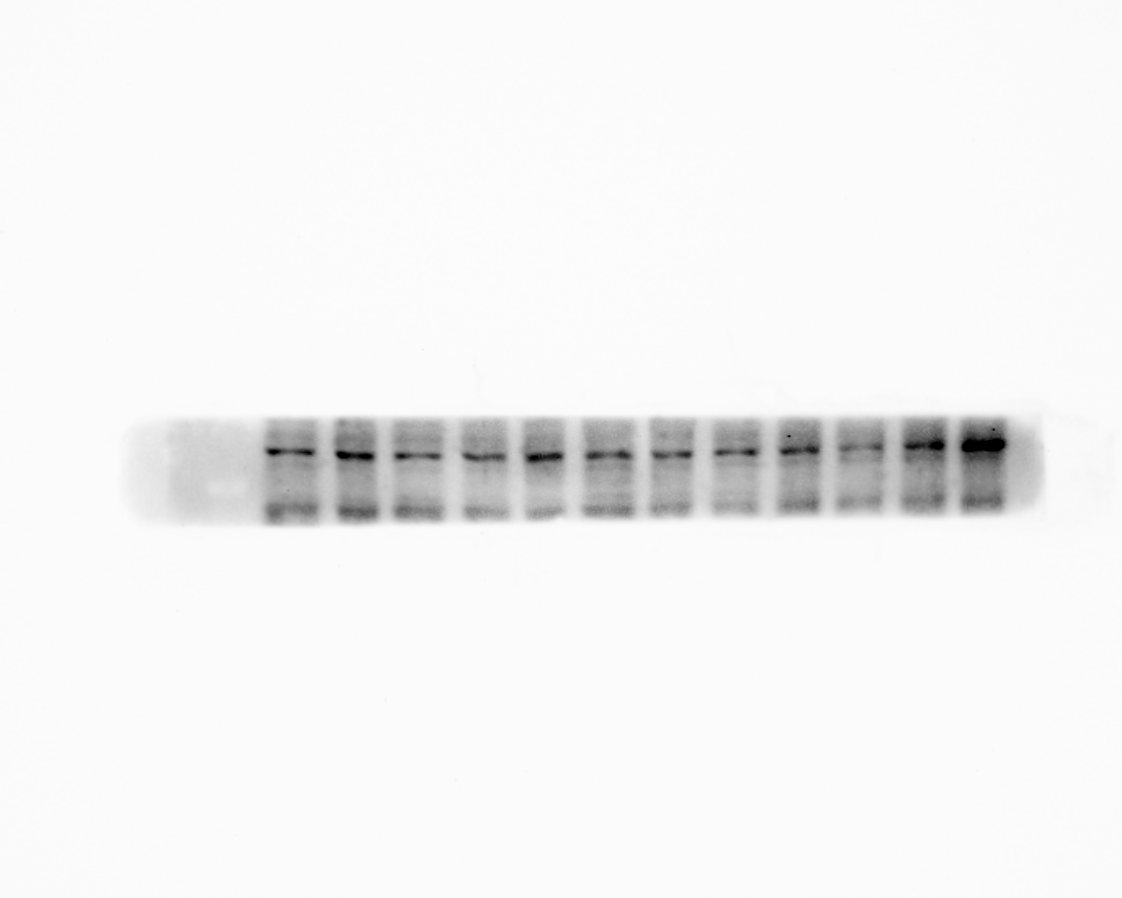

Supplement: Supplementary file 7 — original data [file 41420_2023_1589_MOESM7_ESM.zip › 3G-WB/page8-2 bactin 没有merge 211216 cxz 10% bactin已用.tif]

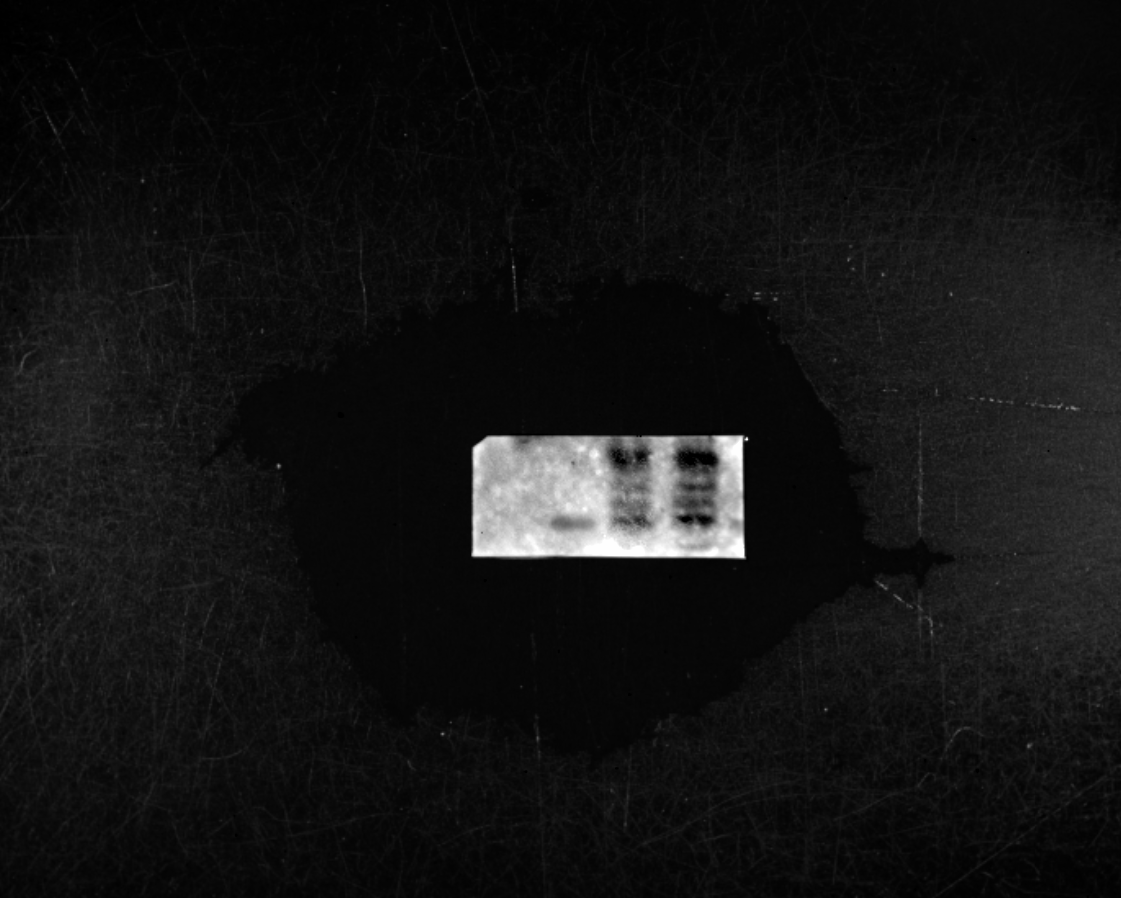

Supplement: Supplementary file 7 — original data [file 41420_2023_1589_MOESM7_ESM.zip › 2J-WB/page4-1 c-cas3 210701 cxz 15% c-casp3_2已用.tif]

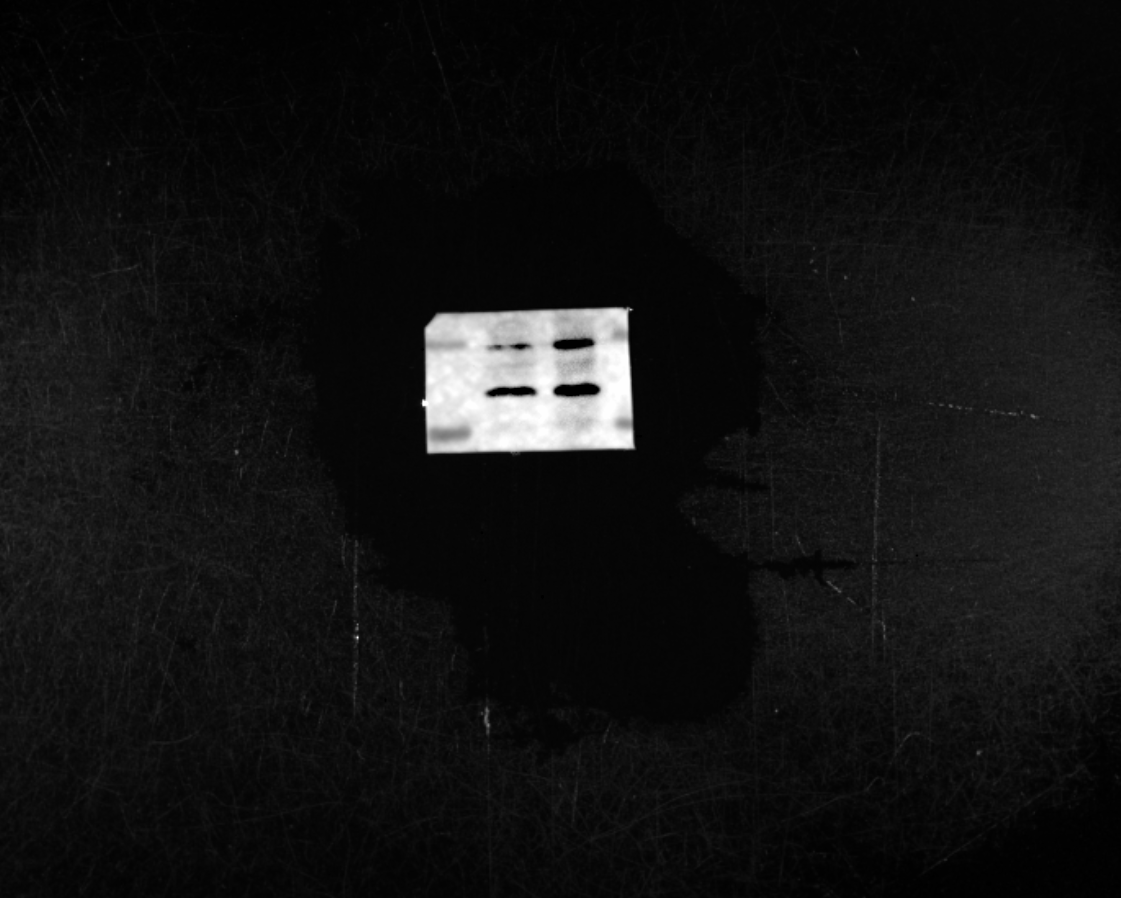

Supplement: Supplementary file 7 — original data [file 41420_2023_1589_MOESM7_ESM.zip › 2J-WB/page4-2 puma 210701 cxz 15% puma_2已用.tif]

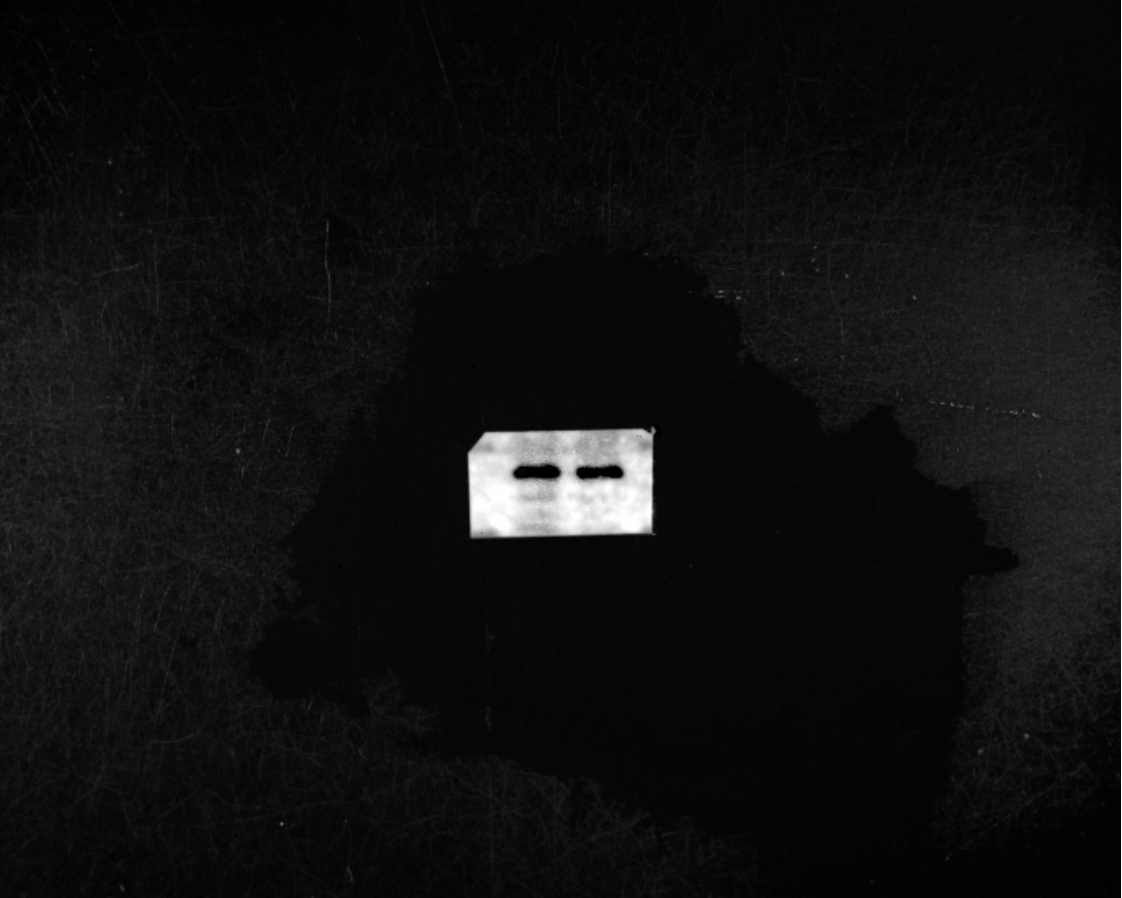

Supplement: Supplementary file 7 — original data [file 41420_2023_1589_MOESM7_ESM.zip › 2J-WB/page4-3 bad 210701 cxz 15% bad_2已用.tif]

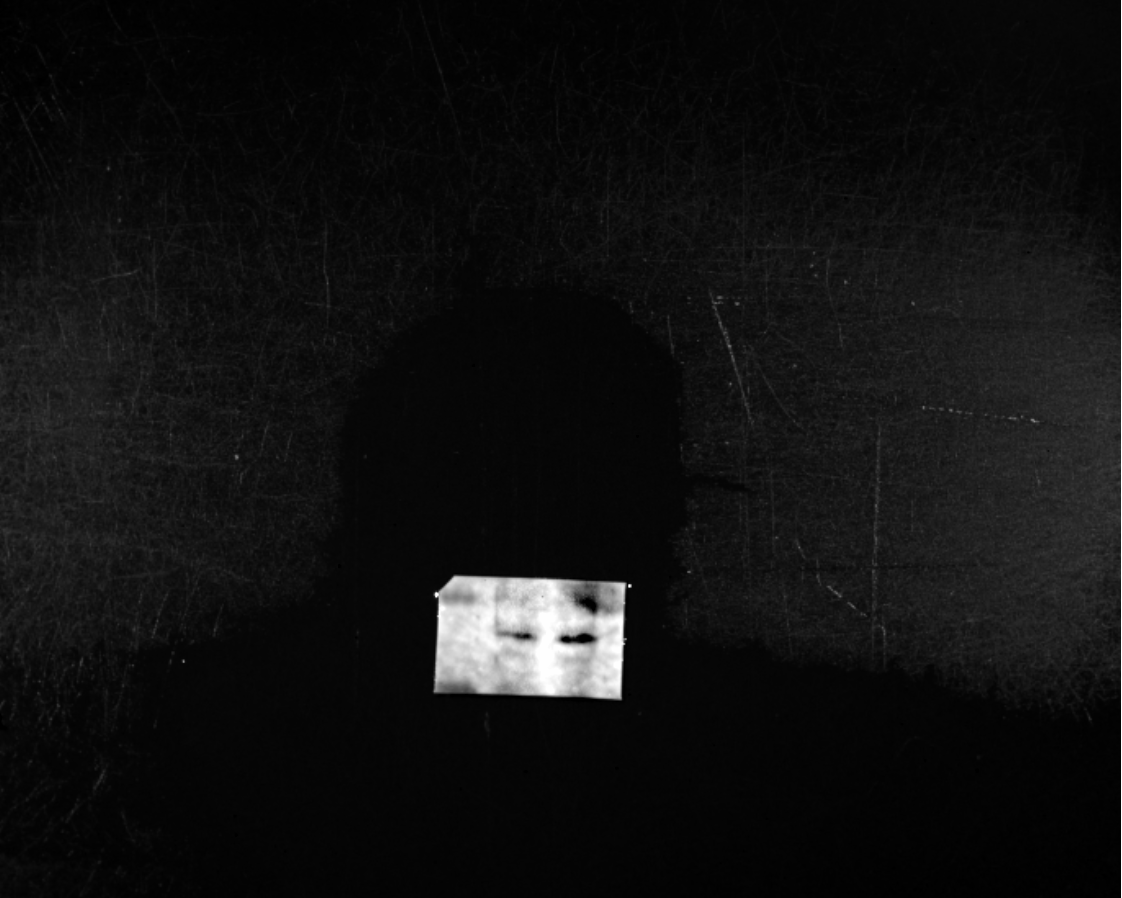

Supplement: Supplementary file 7 — original data [file 41420_2023_1589_MOESM7_ESM.zip › 2J-WB/page4-4 bax 210701 cxz 15% bax_2已用.tif]

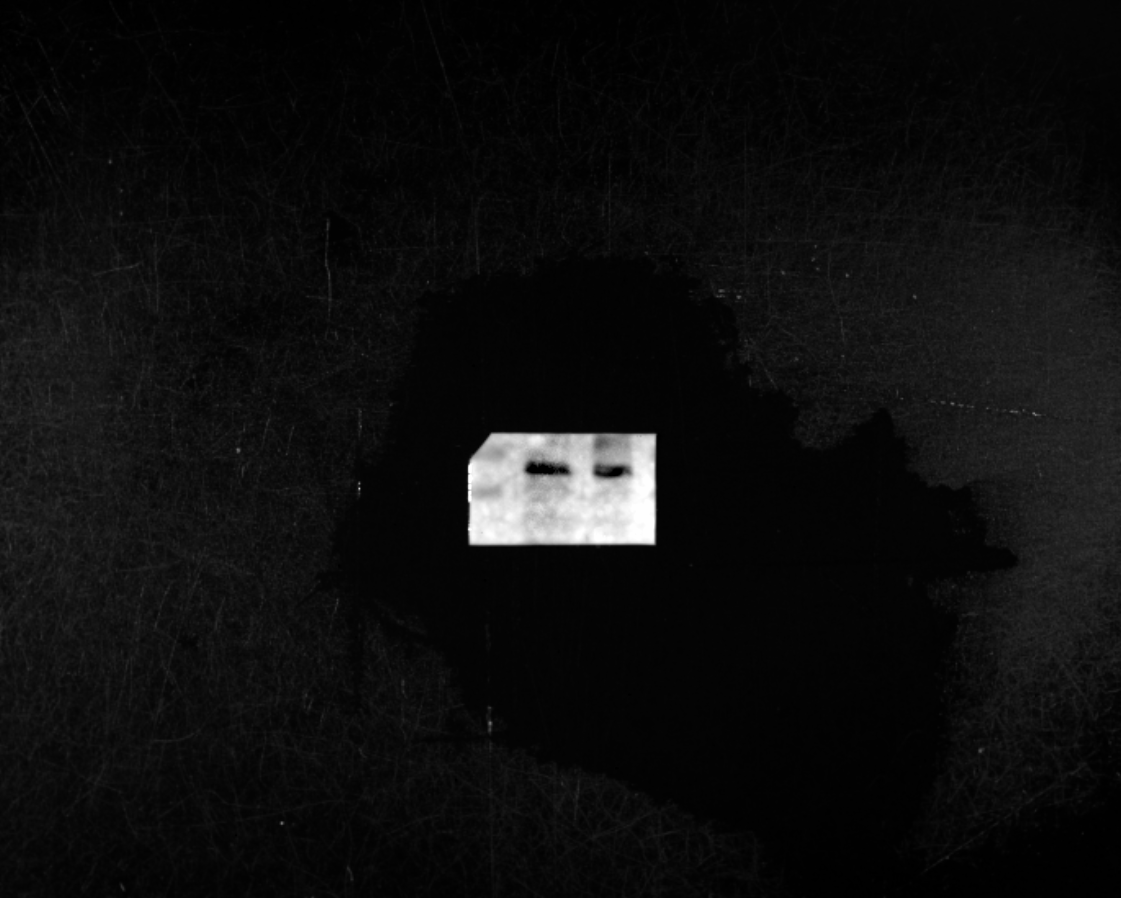

Supplement: Supplementary file 7 — original data [file 41420_2023_1589_MOESM7_ESM.zip › 2J-WB/page4-5 bcl-2 210701 cxz 15% bcl-xl_2已用.tif]

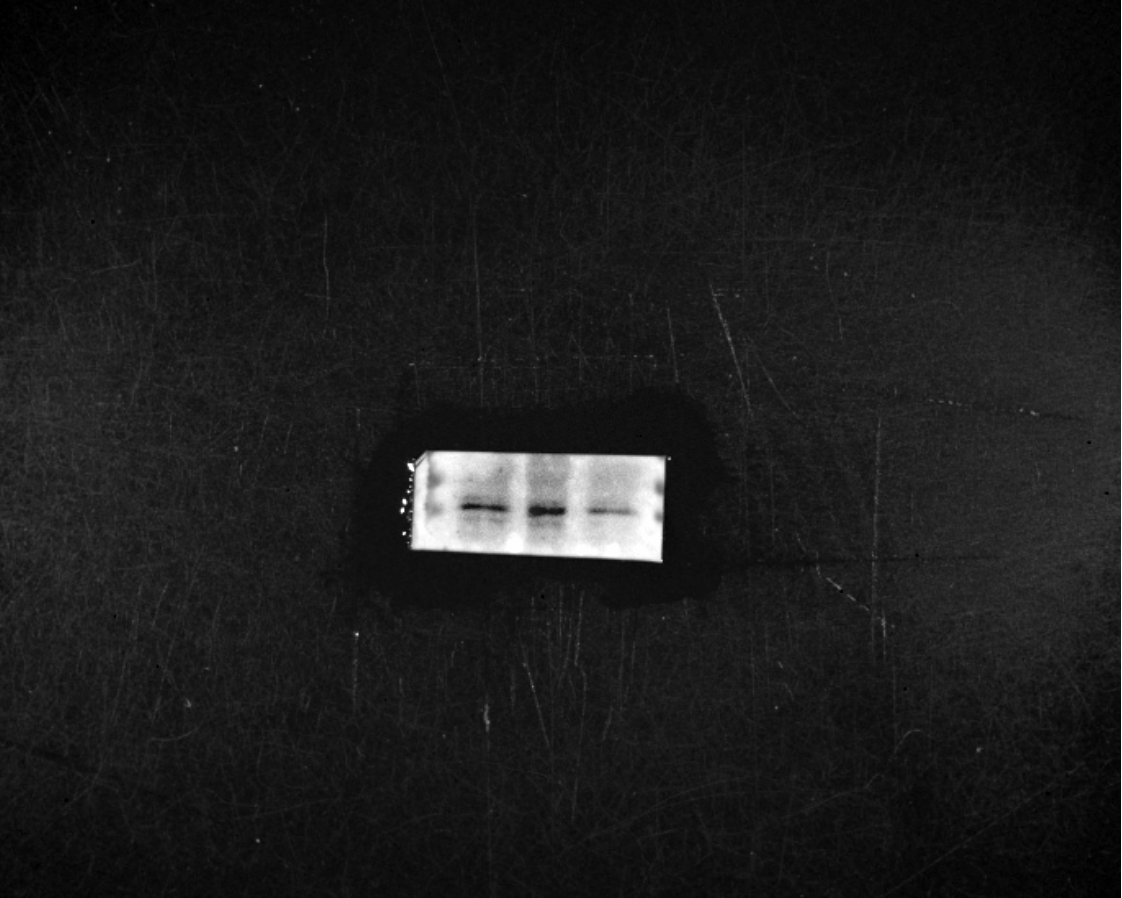

Supplement: Supplementary file 7 — original data [file 41420_2023_1589_MOESM7_ESM.zip › 2J-WB/page4-6 bcl-xl 210629 cxz 15% bcl2_2已用.tif]

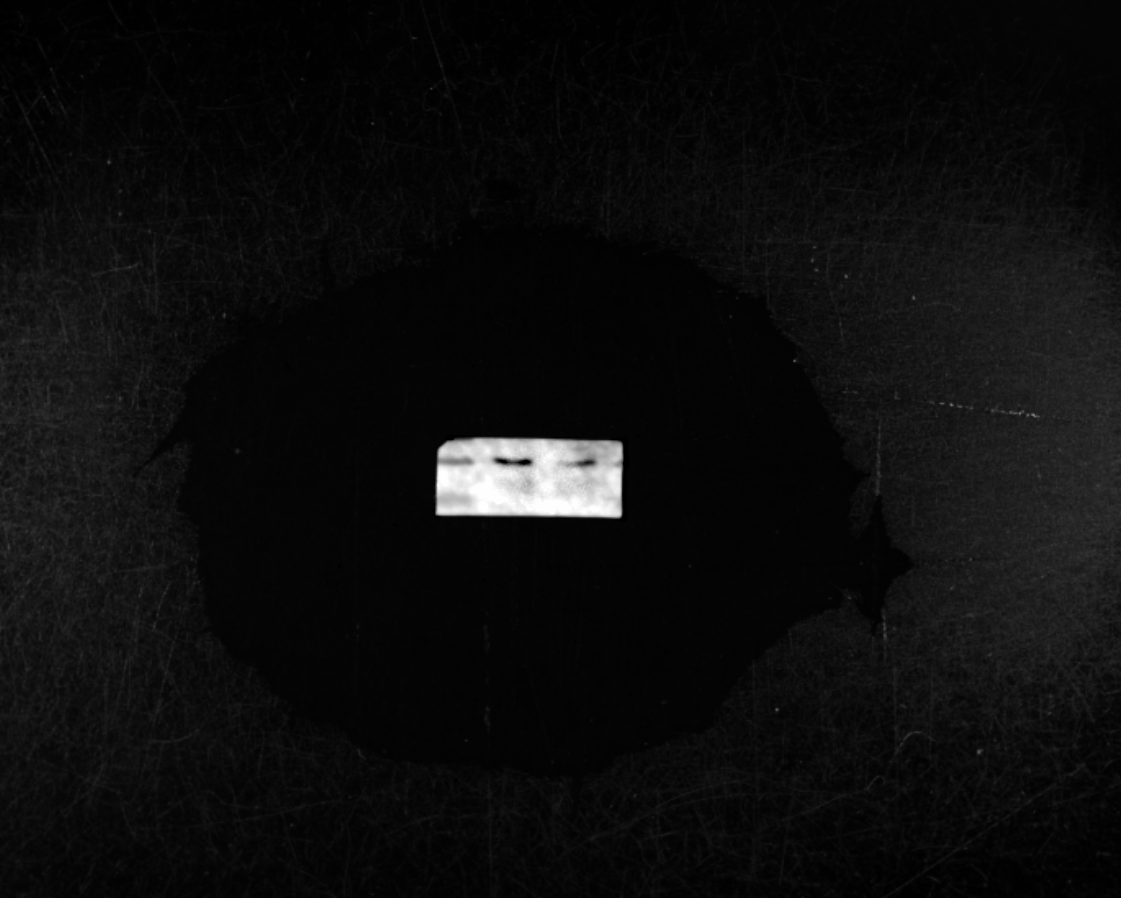

Supplement: Supplementary file 7 — original data [file 41420_2023_1589_MOESM7_ESM.zip › 2J-WB/page4-7 mcl-1 210701 cxz 15% mcl-1_2已用.tif]

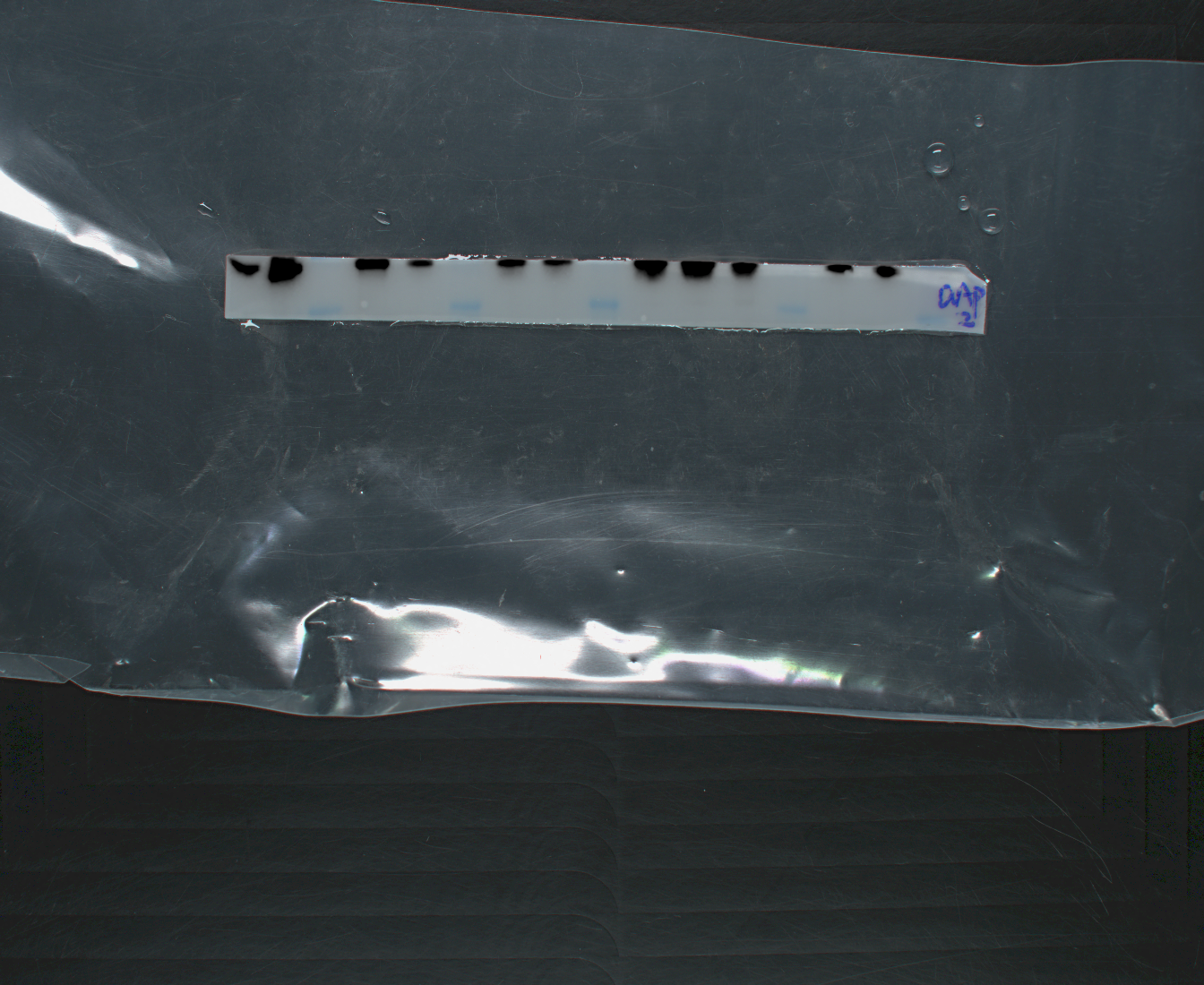

Supplement: Supplementary file 7 — original data [file 41420_2023_1589_MOESM7_ESM.zip › 2J-WB/2022-11-15 GAPDH merge.Tif]
